# Supplementary material for: Electrochemical C−C bond cleavage of cyclopropanes towards the synthesis of 1,3-difunctionalized molecules
Source: Nat Commun. 2021 May 24;12:3075. doi: 10.1038/s41467-021-23401-8 (PMC8144616; doi:10.1038/s41467-021-23401-8)
Supplement: Supplementary file 1 — Supplementary Information [file 41467_2021_23401_MOESM1_ESM.pdf]

Supplementary information

Electrochemical C-C Bond Cleavage of Cyclopropanes towards the Synthesis of 1,3-Difunctionalized Molecules

Pan Peng, Xingxiu Yan, Ke Zhang, Zhao Liu, Li Zeng, Yixuan Chen, Heng Zhang* and Aiwen Lei*

hengzhang@whu.edu.cn

aiwenlei@whu.edu.cn

Table of Contents

General information 3

Experimental section 3

1) General procedure for synthesis of arylcyclopropanes 3

General procedure A: Suzuki coupling[1] 3

General procedure B: Simmons-Smith cyclopropanation[6] 7

2) General procedure for synthesis of protected amide[11] 8

3) Optimization of Reaction Parameters for 1,3-difluorination 8

4) Procedures and analytical data of 1,3-difluorination compounds 10

5) Procedures and analytical data of 1,3-oxyfluorination compounds 22

6) Proposed mechanism of 1,3-oxyfluorination reaction using ether as nucleophiles 28

7) Procedures and analytical data of 1,3-dioxygenation compounds 28

8) Graphical guide and general procedure for gram scale reaction 31

9) Mechanistic study 33

10) General Computational Calculation Details 34

NMR Spectra of Products 36

Supplementary References 141

General information

Unless otherwise noted, materials were obtained from commercial suppliers and used without further purification. $PhCF_3$ was purchased from Aladdin. $Et_3N·3HF$ (95%) was purchased from Accela ChemBio Co., Ltd. Phenylcyclopropane, 1-bromo-4-cyclopropylbenzene and 1-fluoronaphthalene (98%) were purchased from Bidepharm. Other Arylcyclopropanes were prepared according to literature report. Carbon cloth was purchased from CeTech Co., Ltd. Nickel foam was purchased from KUNSHAN JIAYISHENG ELECTRONICS CO.,LTD. Thin layer chromatography (TLC) employed glass 0.25 mm silica gel plates. Flash chromatography columns were packed with 200-300 mesh silica gel. GC yields were recorded with a Shimadzu GC-2014. GC-MS was recorded by Shimadzu GCMS-QP2010 SE. All new compounds were characterized by ^{1}H NMR, ^{13}C NMR, ^{19}F NMR and HRMS. The known compounds were characterized by ^{1}H NMR, ^{13}C NMR and ^{19}F NMR. ^{1}H and ^{13}C NMR data were recorded with ADVANCE III 400 MHz or JNM-ECZ400 400 MHz with tetramethylsilane as an internal standard. All chemical shifts (δ) were reported in ppm and coupling constants (*J*) in Hz. All chemical shifts were reported relative to tetramethylsilane (0 ppm for ^{1}H), and $CDCl_3$ (77.16 ppm for ^{13}C), respectively. High resolution mass spectra (HRMS) were measured with Orbitrap Velos Pro，Thermo Scientific or Waters Micromass GCT Premier or Thermo Fisher Scientific LTQ FT Ultra, accurate masses are reported for the molecular ion + proton ($[M+H]^+$), molecular ion + NH_4^+ ($[M+NH_4]^+$) or molecular ion + Na^+ ($[M+Na]^+$).

Experimental section

1) General procedure for synthesis of arylcyclopropanes

General procedure A: Suzuki coupling[1]

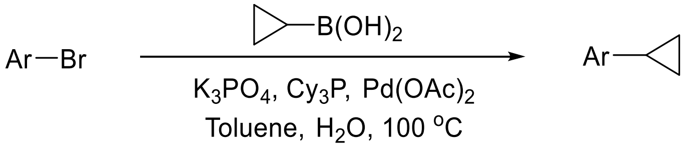

General procedure A: Aryl bromide (6 mmol, 1 equiv.), cyclopropylboronic acid (670 mg, 7.8 mmol, 1.3 equiv.), K_3PO_4 (4.46 g, 21.0 mmol, 3.5 equiv.), Cy_3P (168.2 mg, 0.6 mmol, 0.1 equiv.) and $Pd(OAc)_2$ (67.4 mg, 0.3 mmol, 0.05 equiv.) were added to a three-neck flask equipped with a magnetic stir bar, and the resultant mixture was degassed and filled with argon gas. Toluene (40 mL) and water (2 mL) were added. The resultant mixture was heated at 100 ℃ until aryl halide was consumed (more than 5 h, detected by GC-MS). The reaction was cooled to room temperature and diluted with water. The organic layer was separated and the aqueous layer was extracted with ethyl acetate, dried with anhydrous Na_2SO_4, filtered, and concentrated under reduced pressure. The residue was purified by flash column chromatography on silica gel.

The following were prepared according to general procedure A: **S6**[2], **S8**[1], **S14**[3], **S15**[2], **S17**[4], **S18**[2], **S19**[2], **S21**[1], **S22**[5] and **S24**[1] were previously reported.

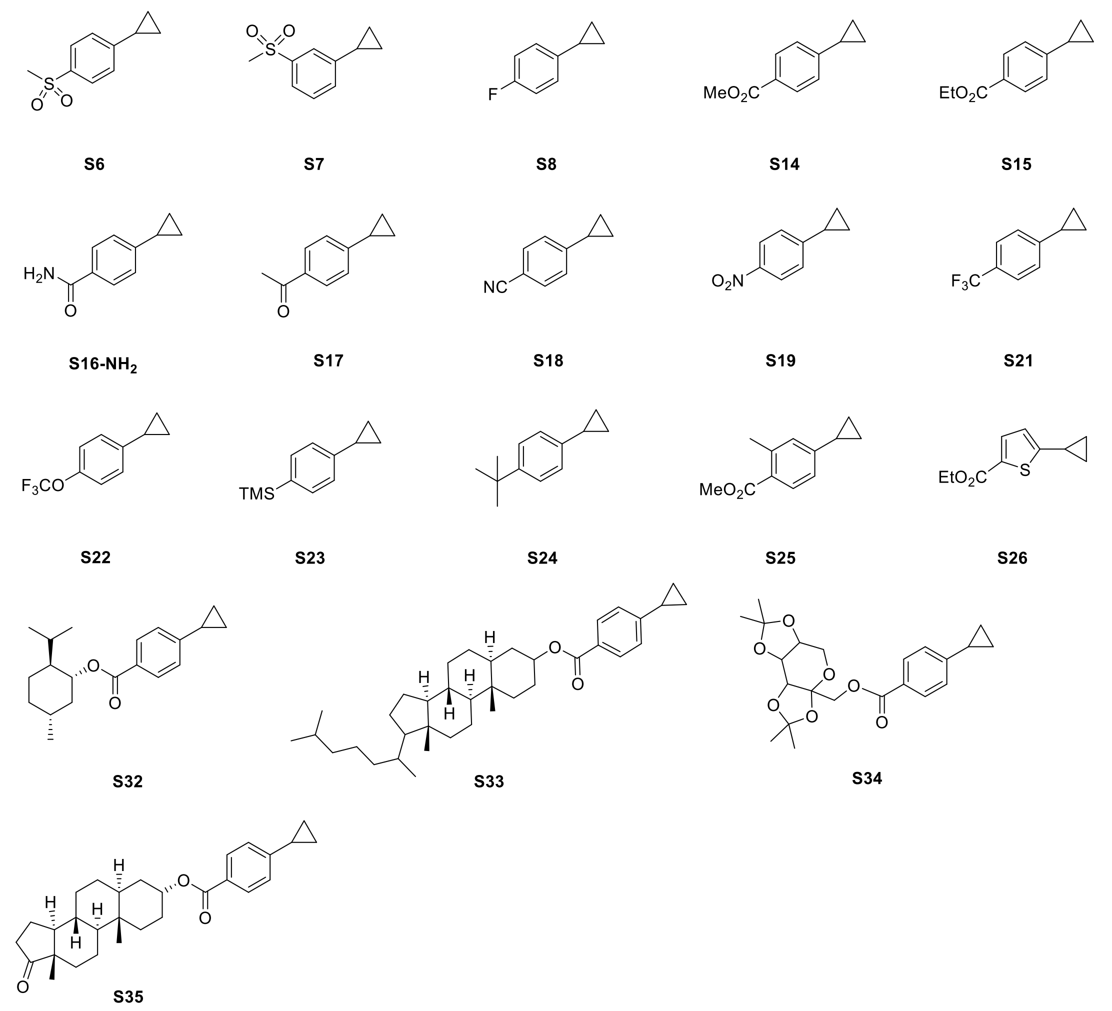

Supplementary Figure 1 Arylcyclopropanes prepared according to general procedure A

Characterization of unknown product:

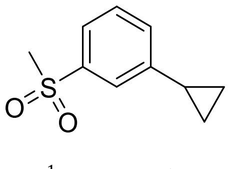

S7. ^{1}H NMR (400 MHz, $CDCl_3$) δ 7.71 (ddd, *J* = 7.7, 1.7, 1.2 Hz, 1H), 7.63 (t, *J* = 1.8 Hz, 1H), 7.45 (t, *J* = 7.7 Hz, 1H), 7.37 – 7.30 (m, 1H), 3.05 (s, 3H), 1.99 (tt, *J* = 8.4, 5.1 Hz, 1H), 1.07 (ddd, *J* = 8.4, 6.5, 4.8 Hz, 2H), 0.77 (dt, *J* = 6.6, 4.9 Hz, 2H). ^{13}C NMR (101 MHz, $CDCl_3$) δ 146.2, 140.5, 131.1, 129.3, 124.4, 124.3, 44.6, 15.5, 10.0. HRMS (ESI) m/z calculated for $C_{10}H_{13}O_2S$ (M+H) $^{+}$:197.06308, found: 197.06302.

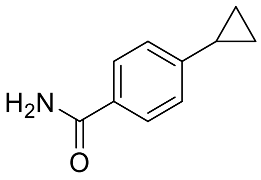

S16-NH₂. ^{1}H NMR (400 MHz, DMSO) δ 7.76 (d, *J* = 8.3 Hz, 2H), 7.13 (d, *J* = 8.3 Hz, 2H), 1.96 (tt, *J* = 8.4, 5.0 Hz, 1H), 1.06 – 0.95 (m, 2H), 0.78 – 0.67 (m, 2H). ^{13}C NMR (101 MHz, DMSO) δ 167.8, 147.6, 131.3, 127.6, 125.0, 15.2, 10.2. HRMS (ESI) m/z calculated for $C_{10}H_{12}NO$ $(M+H)^+$: 162.09134, found: 162.09075.

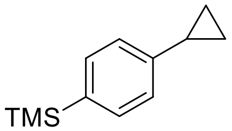

S23. ^{1}H NMR (400 MHz, $CDCl_3$) δ 7.41 (d, *J* = 8.0 Hz, 2H), 7.06 (d, *J* = 7.9 Hz, 2H), 1.94 – 1.81 (m, 1H), 0.99 – 0.92 (m, 2H), 0.74 – 0.68 (m, 2H), 0.24 (s, 9H). ^{13}C NMR (101 MHz, $CDCl_3$) δ 144.9, 137.0, 133.5, 125.2, 15.5, 9.5, -0.9. HRMS (EI) m/z calculated for $C_{12}H_{18}Si$ $(M)^+$: 190.1178, found: 190.1185.

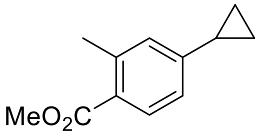

S25. ^{1}H NMR (400 MHz, $CDCl_3$) δ 7.82 (d, *J* = 8.1 Hz, 1H), 6.89 (dd, *J* = 11.6, 3.5 Hz, 2H), 3.86 (s, 3H), 2.57 (s, 3H), 1.87 (tt, *J* = 8.4, 5.0 Hz, 1H), 1.04 – 0.98 (m, 2H), 0.77 – 0.71 (m, 2H). ^{13}C NMR (101 MHz, $CDCl_3$) δ 168.0, 149.0, 140.6, 131.0, 129.90, 126.4, 122.6, 51.7, 22.1, 15.5, 10.1. HRMS (ESI) m/z calculated for $C_{12}H_{15}O_2$ $(M+H)^+$: 191.10666, found: 191.10670.

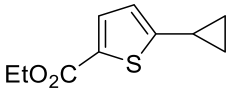

S26. ^{1}H NMR (400 MHz, $CDCl_3$) δ 7.58 (d, *J* = 3.8 Hz, 1H), 6.73 (dd, *J* = 3.8, 0.5 Hz, 1H), 4.31 (q, *J* = 7.1 Hz, 2H), 2.10 (dq, *J* = 8.4, 5.0 Hz, 1H), 1.35 (t, *J* = 7.1 Hz, 3H), 1.07 (ddd, *J* = 8.3, 6.6, 4.6 Hz, 2H), 0.78 (dt, *J* = 6.7, 4.7 Hz, 2H). ^{13}C NMR (101 MHz, $CDCl_3$) δ 162.2, 157.0, 133.6, 129.6, 123.3, 60.9, 14.4, 11.9, 11.0. HRMS (ESI) m/z calculated for $C_{10}H_{13}O_2S$ $(M+H)^+$: 197.06308, found: 197.06308.

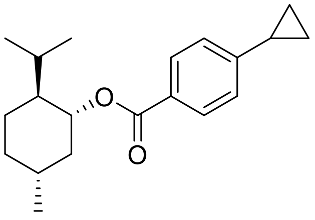

S32. ^{1}H NMR (400 MHz, $CDCl_3$) δ 7.97 – 7.87 (m, 2H), 7.15 – 7.04 (m, 2H), 4.91 (td, *J* = 10.9, 4.4 Hz, 1H), 2.16 – 2.07 (m, 1H), 2.00 – 1.89 (m, 2H), 1.77 – 1.67 (m, 2H), 1.60 – 1.48 (m, 2H), 1.16 – 1.01 (m, 4H), 0.96 – 0.85 (m, 7H), 0.80 – 0.73 (m, 5H). ^{13}C NMR (101 MHz, $CDCl_3$) δ 166.2, 149.9, 129.7, 128.0, 125.4, 74.6, 47.4, 41.1, 34.5, 31.6, 26.6, 23.8, 22.2, 20.9, 16.7, 15.8, 10.4. HRMS (ESI) m/z calculated for $C_{20}H_{28}O_2Na$ $(M+Na)^+$: 323.19815, found: 323.19800.

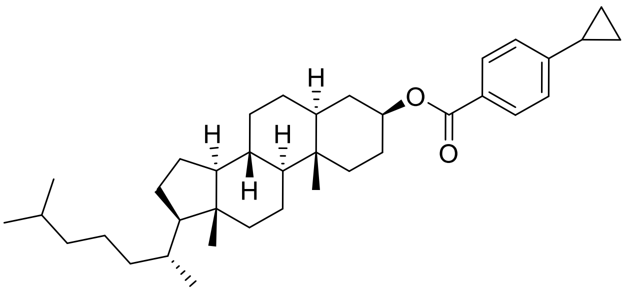

S33. ^{1}H NMR (400 MHz, $CDCl_3$) δ 7.91 (d, *J* = 8.3 Hz, 2H), 7.08 (d, *J* = 8.3 Hz, 2H), 5.05 – 4.77 (m, 1H), 2.01 – 1.88 (m, 3H), 1.87 – 0.80 (m, 42H), 0.78 – 0.73 (m, 2H), 0.71 – 0.61 (m, 4H). ^{13}C NMR (101 MHz, $CDCl_3$) δ 166.3, 149.8, 129.7, 128.1, 125.3, 74.2, 56.6, 56.3(8), 54.3(5), 44.8, 42.7, 40.1, 39.7, 36.9, 36.3, 36.0, 35.7, 35.6, 34.3, 32.2, 28.8, 28.4, 28.2, 27.7, 24.4, 24.0, 23.0, 22.7, 21.4, 18.8, 15.8, 12.5, 12.2, 10.4. HRMS (DART POSITIVE) m/z calculated for $C_{34}H_{57}O_2$ $(M+H)^+$: 533.4353, found: 533.4352.

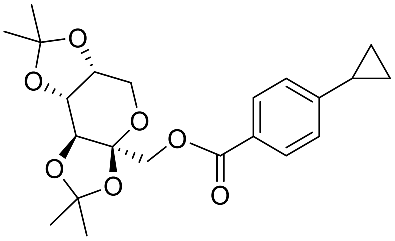

S34. ^{1}H NMR (400 MHz, $CDCl_3$) δ 7.95 (d, *J* = 8.4 Hz, 2H), 7.09 (d, *J* = 8.4 Hz, 2H), 4.69 – 4.63 (m, 2H), 4.47 (d, *J* = 2.6 Hz, 1H), 4.34 – 4.23 (m, 2H), 4.01 – 3.74 (m, 2H), 1.98 – 1.89 (m, 1H), 1.55 (s, 3H), 1.47 (s, 3H), 1.37 (s, 3H), 1.35 (s, 3H), 1.12 – 1.01 (m, 2H), 0.82 – 0.72 (m, 2H). ^{13}C NMR (101 MHz, $CDCl_3$) δ 166.1, 150.4, 130.0, 127.0, 125.4, 109.3, 109.0, 101.8, 70.9, 70.6, 70.2, 65.1, 61.5, 26.7, 26.0, 25.7, 24.2, 15.9, 10.5. HRMS (ESI) m/z calculated for $C_{22}H_{29}O_7$ $(M+H)^+$: 405.19078, found: 405.18947.

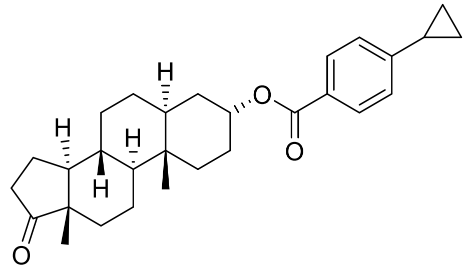

S35. ^{1}H NMR (400 MHz, $CDCl_3$) δ 7.95 – 7.87 (m, 2H), 7.09 (d, *J* = 8.3 Hz, 2H), 4.98 – 4.86 (m, 1H), 2.48 – 2.40 (m, 1H), 2.14 – 2.03 (m, 1H), 1.97 – 1.90 (m, 3H), 1.85 – 1.72 (m, 4H), 1.71 – 1.44 (m, 5H), 1.41 – 1.21 (m, 6H), 1.17 – 0.96 (m, 4H), 0.90 (s, 3H), 0.87 (s, 3H), 0.78 – 0.71 (m, 3H). ^{13}C NMR (101 MHz, $CDCl_3$) δ 221.6, 166.3, 149.9, 129.7, 128.0, 125.3, 74.0, 54.4, 51.5, 47.9, 44.8, 36.9, 36.0, 35.8, 35.2, 34.2, 31.7, 31.0, 28.4, 27.7, 21.9, 20.6, 15.8, 14.0, 12.4, 10.4. HRMS (ESI) m/z calculated for $C_{29}H_{39}O_3$ $(M+H)^+$: 435.28937, found: 435.28876.

General procedure B: Simmons-Smith cyclopropanation[6]

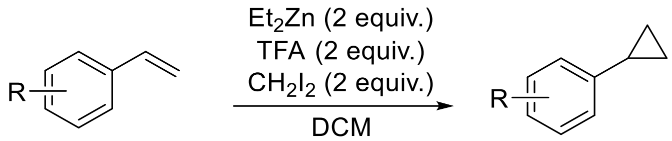

General procedure B: To 15 mL distilled DCM was added 10.0 mL Et_2Zn (10.0 mmol, 2 equiv., 1.0 M in hexanes) under N_2 atmosphere. The solution was cooled in an ice bath and a solution of trifluoroacetic acid (0.8 mL, 10.0 mmol, 2 equiv.) in 5 mL DCM was then dripped very slowly into the reaction mixture via syringe. Upon stirring for 20 min, a solution of CH_2I_2 (0.8 mL, 10.0 mmol, 2 equiv.) in 5 mL DCM was added. After an additional 20 min of stirring, a solution of the respective styrene derivative (5.0 mmol, 1 equiv.) in 5 mL DCM was added, and the ice bath was removed. After starting styrene derivative was consumed (more than 12 h, detected by GC-MS), the reaction mixture was quenched with 1M HCl (10 mL) and the phases were separated. The aqueous layer was extracted with DCM. The combined organic layers were washed with saturated $NaHCO_3$, and brine and then dried with Na_2SO_4, filtered and concentrated. The residue was purified by column chromatography to give the title compound.

The following were prepared according to general procedure B, **S9**[1], **S11[1]**, **S12[6]**, **S13**[7], **S27**[8], **S28**[8], **trans-S30**[9] and **S31[10]** were previously reported.

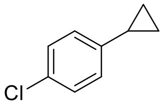

S9

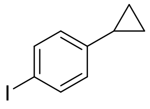

S11

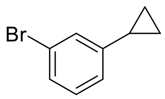

S12

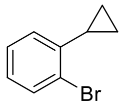

S13

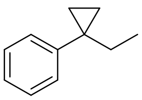

S27

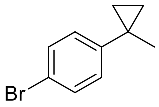

S28

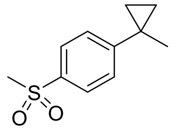

S29

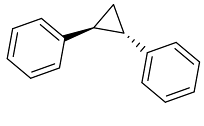

trans-S30

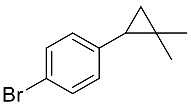

S31

Supplementary Figure 2 Arylcyclopropanes prepared according to general procedure B

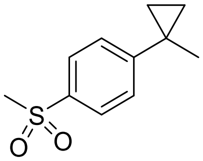

S29. ^1H NMR (400 MHz, $CDCl_3$) δ 7.90 – 7.79 (m, 2H), 7.44 – 7.34 (m, 2H), 3.04 (s, 3H), 1.45 (s, 3H), 0.99 – 0.79 (m, 4H). ^{13}C NMR (101 MHz, $CDCl_3$) δ 154.0, 137.4, 127.4, 127.2, 44.7, 24.9, 19.8, 17.1. HRMS (ESI) m/z calculated for $C_{11}H_{15}O_2S$ $(M+H)^+$: 211.07873, found: 211.07854.

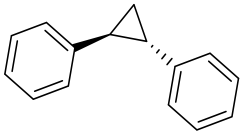

Trans-S30. ^{1}H NMR (400 MHz, $CDCl_3$) δ 7.32 – 7.26 (m, 4H), 7.22 – 7.11 (m, 6H), 2.21 – 2.12 (m, 2H), 1.47 – 1.43 (m, 2H). ^{13}C NMR (101 MHz, $CDCl_3$) δ 142.6, 128.5, 125.9, 28.2, 18.4. The observed spectral data are in agreement with the reported literature.

2) General procedure for synthesis of protected amide[11]

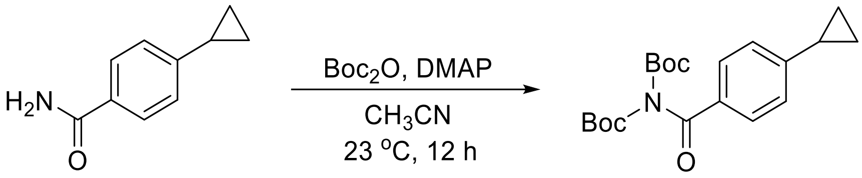

To a flask containing amide (4.6 mmol) was added DMAP (56.7mg, 0.46 mmol, 0.1 equiv.), followed by acetonitrile (23 mL, 0.2 M). Boc_2O (2.03 g, 9.28 mmol, 2.0 equiv.) was added in one portion and the reaction vessel was flushed with N_2. The reaction mixture was allowed to stir at 23 ℃ for 12 h. The mixture was washed with 1.0 M HCl (5 mL) and brine (5 mL). After drying over Na_2SO_4, filtered and the organics were concentrated under reduced pressure.

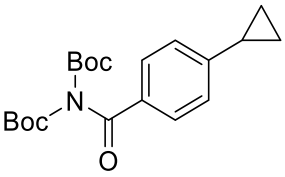

S16. ^{1}H NMR (400 MHz, $CDCl_3$) δ 7.77 – 7.70 (m, 2H), 7.12 (d, *J* = 8.4 Hz, 2H), 1.96 (ddd, *J* = 13.4, 8.4, 5.0 Hz, 1H), 1.38 (s, 18H), 1.12 – 1.06 (m, 2H), 0.80 (dt, *J* = 6.7, 4.8 Hz, 2H). ^{13}C NMR (101 MHz, $CDCl_3$) δ 169.0, 151.4, 150.0, 131.1, 129.6, 125.7, 84.2, 27. 8, 16.0, 10.8. HRMS (ESI) m/z calculated for $C_{20}H_{27}NO_5Na$ $(M+Na)^+$: 384.17814, found: 384.17697.

3) Optimization of Reaction Parameters for 1,3-difluorination

Supplementary Table 1. Evaluation of solvents

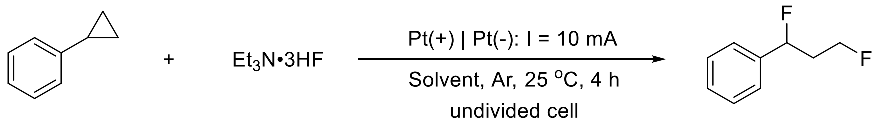

1 **2**

Entry	Solvent	Yield [%][a]
1	CH_3CN	11 %
2	DCE	55 %
3	CH_2Cl_2	27 %
4	Acetone	3 %
5	CH_3NO_2	1 %
6	EtOAc	11 %
7	THF	14 %
8	DME	23 %
9	TBME	9 %
10	PhMe	0 %
11	$PhCF_3$	72 %

Reaction conditions: undivided cell, Pt anode, Pt cathode, **1** (0.5 mmol), Et_3N•3HF (1.2 mL), solvent (4.8 mL), 10 mA. [a] GC Yield using biphenyl as internal standard.

Supplementary Table 2. Evaluation of current

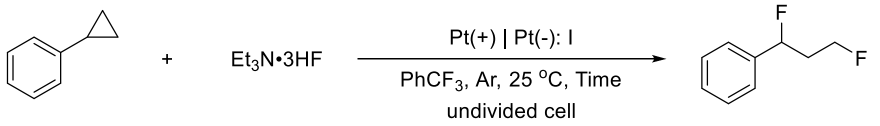

1 **2**

Entry	I, Time	Yield [%][a]
1	5 mA, 8 h	71 %
2	10 mA, 4 h	72 %
3	16 mA, 2 h	77 %
4	20 mA, 96 min	73 %

Reaction conditions: undivided cell, Pt anode, Pt cathode, **1** (0.5 mmol), Et_3N·3HF (1.2 mL), $PhCF_3$ (4.8 mL). [a] GC Yield using biphenyl as internal standard.

Supplementary Table 3. Evaluation of reaction temperature

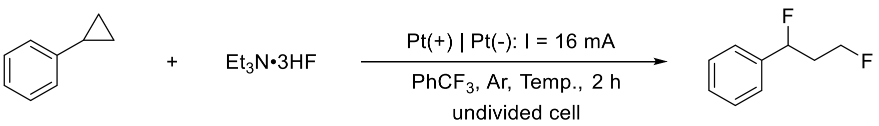

1 **2**

Entry	Temp.	Yield [%][a]
1	0 ^{o}C	49 %
2	25 ^{o}C	77 %
3	60 ^{o}C	55 %

Reaction conditions: undivided cell, Pt anode, Pt cathode, **1** (0.5 mmol), Et_3N•3HF (1.2 mL), $PhCF_3$ (4.8 mL). [a] GC Yield using biphenyl as internal standard.

Supplementary Table 4. Evaluation of electrode materials

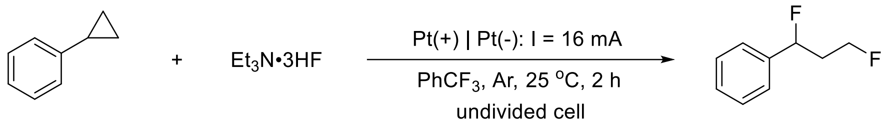

1 **2**

Entry	Electrode	Yield [%][a]
1	Pt(+) | Pt(-)	77 %
2	C cloth(+) | Pt(-)	69 %
3	C rod(+) | Pt(-)	63 %
4	Pt(+) | Ni plate(-)	71 %
5	Pt(+) | Ni foam(-)	74 %

Reaction conditions: undivided cell, Pt anode, Pt cathode, **1** (0.5 mmol), $Et_3N{\bullet}3HF$ (1.2 mL), $PhCF_3$ (4.8 mL). [a] GC Yield using biphenyl as internal standard.

Supplementary Table 5. Evaluation of amount of $Et_3N{\cdot}3HF$ and electricity

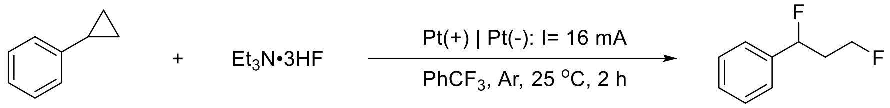

1 **2**

Entry	Variation from standard conditions	Yield [%][a]
9	0.5 mL $Et_3N{\bullet}3HF$, 5.5 mL $PhCF_3$	64%
10	5 mL $Et_3N{\bullet}3HF$	66%
11	no electric current	N.R.

Reaction conditions: undivided cell, Pt anode, Pt cathode, **1** (0.5 mmol), $Et_3N{\bullet}3HF$ (1.2 mL), $PhCF_3$ (4.8 mL). [a] GC Yield using biphenyl as internal standard.

4) Procedures and analytical data of 1,3-difluorination compounds

Graphical guide for the set-up: As experiment set-up, a platinum plate electrode anode (15 mm×15 mm×0.3 mm), a platinum plate electrode cathode (15 mm×15 mm×0.3 mm), rubber plugs, an undivided three-necked bottle and a dual display potentiostat (HJS-292B) (made in China) were used.

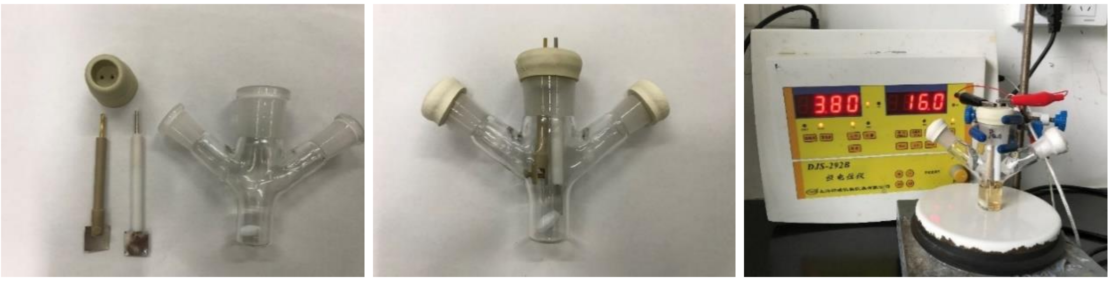

a) Platinum plate anode and cathode **b) Assembly of electrochemical cell** **c) Current control electrosis**

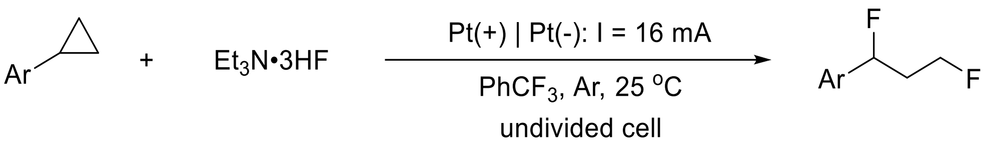

General procedure for the electrolysis: An oven-dried undivided three-necked bottle equipped with a stir bar. The bottle was equipped platinum plate (15 mm×15mm×0.3 mm) as the anode and platinum plate (15 mm×15 mm×0.3 mm) as the cathode and then charged with argon gas by glove box. Arylcyclopropane (0.1 mmol, 0.25 mmol or 0.5 mmol), $Et_3N·3HF$ (1.2 mL) and $PhCF_3$ (4.8 mL) were added. The reaction mixture was stirred and electrolyzed at a constant current of 16 mA under room temperature (monitored by TLC). The reaction was diluted with water. The organic layer was extracted with CH_2Cl_2, dried with anhydrous Na_2SO_4, filtered, and concentrated under reduced pressure. The pure product was obtained by flash column chromatography on silica gel.

Analytical data of compounds:

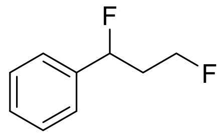

(1,3-Difluoropropyl)benzene (2). 0.5 mmol scale, 2 h. After work-up, the crude residue was purified by flash column chromatography on silica gel (petroleum ether: ethyl acetate = 200:1) to give **3** as a colorless oil. NMR Yield = 75% using 1-fluoronaphthalene as internal standard, electricity = 2.4 F mol^{-1}. ^1H NMR (400 MHz, $CDCl_3$) δ 7.42 – 7.32 (m, 5H), 5.64 (ddd, *J* = 47.8, 9.1, 4.1 Hz, 1H), 4.78 – 4.41 (m, 2H), 2.40 – 2.08 (m, 2H). ^{13}C NMR (101 MHz, $CDCl_3$) δ 139.5 (d, *J* = 19.5 Hz), 128.7(2), 128.6(9) (d, *J* = 2.0 Hz), 125.6 (d, *J* = 6.7 Hz), 90.7 (dd, *J* = 170.7, 4.8 Hz), 80.1 (dd, *J* = 165.0, 4.7 Hz), 38.3 (dd, *J* = 24.1, 19.7 Hz). ^{19}F NMR (377 MHz, $CDCl_3$) δ -178.62 (ddd, *J* = 46.5, 31.0, 14.7 Hz, 1F), -222.50 (tdd, *J* = 46.8, 30.4, 20.4 Hz, 1F). HRMS (EI) m/z calculated for $C_9H_{10}F_2$ $(M)^+$: 156.0751, found: 156.0752.

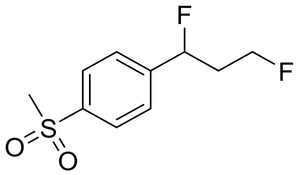

1-(1,3-Difluoropropyl)-4-(methylsulfonyl)benzene (6).[2] 0.5 mmol scale, 200 min. After work-up, the crude residue was purified by flash column chromatography on silica gel (petroleum ether: ethyl acetate = 3:1) to give **6** as a yellow solid**.** Yield = 75%, electricity = 4.0 F mol^{-1}. ^1H NMR (400 MHz, $CDCl_3$) δ 7.98 (d, *J* = 8.1 Hz, 2H), 7.57 (d, *J* = 8.6 Hz, 2H), 5.84 – 5.68 (m, 1H), 4.81 – 4.48 (m, 2H), 3.08 (s, 3H), 2.34 – 2.17 (m, 2H). ^{13}C NMR (101 MHz, $CDCl_3$) δ 145.7 (d, *J* = 19.9 Hz), 140.5 (d, *J* = 1.4 Hz), 127.8, 126.2 (d, *J* = 7.6 Hz), 89.6 (dd, *J* = 173.9, 4.4 Hz), 79.5 (dd, *J* = 165.8, 4.4 Hz), 44. 5, 38.2 (dd, *J* = 23.3, 19.8 Hz). ^{19}F NMR (377 MHz, $CDCl_3$) δ -183.63 (ddd, *J* = 47.3, 28.9, 19.1 Hz, 1F), -222.13 – -222.64 (m, 1F). HRMS (ESI) m/z calculated for $C_{10}H_{13}F_2O_2S$ $(M+H)^+$: 235.05988, found: 235.05965.

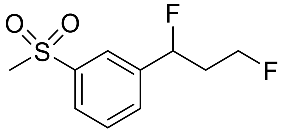

1-(1,3-difluoropropyl)-3-(methylsulfonyl)benzene (7). 0.5 mmol scale, 200 min. After work-up, the crude residue was purified by flash column chromatography on silica gel (petroleum ether: ethyl acetate = 3:1) to give **7** as a yellow oil. Yield = 75%, electricity = 4.0 F mol^{-1}. ^{1}H NMR (400 MHz, $CDCl_3$) δ 8.03 – 7.90 (m, 2H), 7.69 – 7.60 (m, 2H), 5.86 – 5.66 (m, 1H), 4.86 – 4.45 (m, 2H), 3.09 (s, 3H), 2.38 – 2.17 (m, 2H). ^{13}C NMR (101 MHz, $CDCl_3$) δ 141.5 (d, *J* = 20.4 Hz), 141.2, 130.7 (d, *J* = 6.9 Hz), 129.9, 127.5 (d, *J* = 1.4 Hz), 124.3 (d, *J* = 7.6 Hz), 89. 7 (dd, *J* = 173.6, 4.4 Hz), 79.6 (dd, *J* = 165.9, 4.4 Hz), 44.5, 38.1 (dd, *J* = 23.5, 19.8 Hz). ^{19}F NMR (377 MHz, $CDCl_3$) δ -181.37 – -181.68 (m, 1F), -222.23 – -222.62 (m, 1F). HRMS (ESI) m/z calculated for $C_{10}H_{13}F_2O_2S$ $(M+H)^+$:235.05988, found: 235.05975.

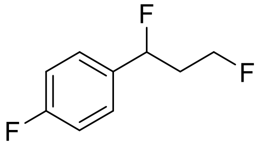

1-(1,3-Difluoropropyl)-4-fluorobenzene (8). 0.5 mmol scale, 2 h. After work-up, the crude residue was purified by flash column chromatography on silica gel (petroleum ether: ethyl acetate = 200:1) to give **8** as a colorless oil. Yield = 52%, electricity = 2.4 F mol^{-1}. ^{1}H NMR (400 MHz, $CDCl_3$) δ 7.51 – 7.30 (m, 2H), 7.17 – 7.03 (m, 2H), 5.63 (ddd, *J* = 47.6, 9.1, 4.1 Hz, 1H), 4.82 – 4.41 (m, 2H), 2.41 – 2.07 (m, 2H). ^{13}C NMR (101 MHz, $CDCl_3$) δ 162.9 (dd, *J* = 247.2, 2.1 Hz), 135.3 (dd, *J* = 20.0, 3.2 Hz), 127.6 (dd, *J* = 8.2, 6.5 Hz), 115.8 (d, *J* = 21.7 Hz), 90.2 (dd, *J* = 170.8, 4.6 Hz), 80.0 (dd, *J* = 165.2, 4.7 Hz), 38.3 (dd, *J* = 24.5, 19.8 Hz). ^{19}F NMR (377 MHz, $CDCl_3$) δ -113.13 – 113.22 (m, 1F), -176.28 – -176.61 (m, 1F), -222.59 (tdd, *J* = 46.9, 30.8, 20.4 Hz, 1F). HRMS (EI) m/z calculated for $C_9H_9F_3$ $(M)^+$: 174.0656, found: 174.0659.

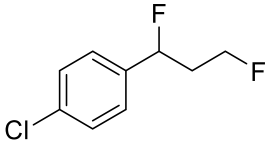

1-chloro-4-(1,3-difluoropropyl)benzene (9). 0.5 mmol scale, 2 h. After work-up, the crude residue was purified by flash column chromatography on silica gel (petroleum ether: ethyl acetate = 200:1) to give **9** as a pale yellow oil**.** Yield = 66%, electricity = 2.4 F mol^{-1}. NMR (400 MHz, $CDCl_3$) δ 7.36 (d, *J* = 8.3 Hz, 2H), 7.28 (d, *J* = 8.3 Hz, 2H), 5.62 (ddd, *J* = 47.6, 9.0, 4.1 Hz, 1H), 4.78 – 4.41 (m, 2H), 2.38 – 2.05 (m, 2H). ^{13}C NMR (101 MHz, $CDCl_3$) δ 138.1 (d, *J* = 19.9 Hz), 134.5 (d, *J* = 2.3 Hz), 129.0, 127.0 (d, *J* = 6.8 Hz), 90.0 (dd, *J* = 171.6, 4.7 Hz), 79.9 (dd, *J* = 165.4, 4.7 Hz), 38.2 (dd, *J* = 24.1, 19.8 Hz). ^{19}F NMR (565 MHz, $CDCl_3$) δ -178.98 (ddd, *J* = 47.1, 31.5, 14.6 Hz, 1F), -222.40 – -222.73 (m, 1F). HRMS (EI) m/z calculated for $C_9H_9ClF_2$ $(M)^+$: 190.0361, found: 190.0368.

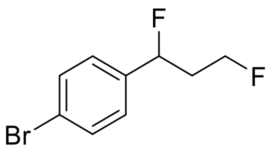

1-Bromo-4-(1,3-difluoropropyl)benzene (10). 0.5 mmol scale, 2 h. After work-up, the crude residue was purified by flash column chromatography on silica gel (petroleum ether: ethyl acetate

= 200:1) to give **10** as a colorless oil. Yield = 67%, electricity = 2.4 F mol^{-1}. ^{1}H NMR (400 MHz, $CDCl_3$) δ 7.52 (d, *J* = 8.0 Hz, 2H), 7.22 (d, *J* = 8.2 Hz, 2H), 5.60 (ddd, *J* = 47.6, 9.0, 4.1 Hz, 1H), 4.78 – 4.41 (m, 2H), 2.37 – 2.05 (m, 2H). ^{13}C NMR (101 MHz, $CDCl_3$) δ 138.6 (d, *J* = 20.0 Hz), 131.9, 127.3 (d, *J* = 6.8 Hz), 122.6 (d, *J* = 2.4 Hz), 90.1 (dd, *J* = 171.8, 4.6 Hz), 79.8 (dd, *J* = 165.5, 4.6 Hz), 38.2 (dd, *J* = 24.0, 19.7 Hz). ^{19}F NMR (377 MHz, $CDCl_3$) δ -179.35 (ddd, *J* = 46.8, 30.9, 14.9 Hz, 1F), -222.40 (tdd, *J* = 46.9, 30.6, 20.4 Hz, 1F). HRMS (EI) m/z calculated for $C_9H_9BrF_2$ $(M)^+$: 233.9856, found: 233.9860.

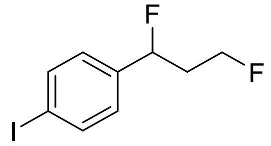

1-(1,3-Difluoropropyl)-4-iodobenzene (11). 0.5 mmol, 140 min. After work-up, the crude residue was purified by flash column chromatography on silica gel (petroleum ether: ethyl acetate = 200:1) to give **11** as a colorless oil. Yield = 52%, electricity = 2.8 F mol^{-1}. ^{1}H NMR (400 MHz, $CDCl_3$) δ 7.72 (d, *J* = 7.8 Hz, 2H), 7.09 (d, *J* = 8.1 Hz, 2H), 5.70 – 5.48 (m, 1H), 4.79 – 4.40 (m, 2H), 2.36 – 2.05 (m, 2H). ^{13}C NMR (101 MHz, $CDCl_3$) δ 139.2 (d, *J* = 19.9 Hz), 137.8, 127.4 (d, *J* = 6.8 Hz), 94.3 (d, *J* = 2.5 Hz), 90.1 (dd, *J* = 171.9, 4.6 Hz), 79.8 (dd, *J* = 165.5, 4.6 Hz), 38.2 (dd, *J* = 23.9, 19.8 Hz). ^{19}F NMR (377 MHz, $CDCl_3$) δ -180.13 (ddd, *J* = 46.8, 31.0, 15.0 Hz, 1F), -222.54 (tdd, *J* = 46.7, 30.7, 20.4 Hz, 1F). HRMS (EI) m/z calculated for $C_9H_9F_2I$ $(M)^+$: 281.9717, found: 281.9723.

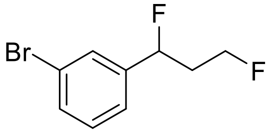

1-bromo-3-(1,3-difluoropropyl)benzene (12). 0.5 mmol scale, add 0.5 mL DCE, 2 h. After work-up, the crude residue was purified by flash column chromatography on silica gel (petroleum ether: ethyl acetate = 200:1) to give **12** as a colorless oil. Yield = 63%, electricity = 2.4 F·mol^{-1}. ^{1}H NMR (400 MHz, $CDCl_3$) δ 7.63 – 7.46 (m, 2H), 7.36 – 7.27 (m, 2H), 5.65 (ddd, *J* = 47.7, 8.8, 4.3 Hz, 1H), 4.83 – 4.47 (m, 2H), 2.42 – 2.10 (m, 2H). ^{13}C NMR (101 MHz, $CDCl_3$) δ 141.8 (d, *J* = 19.9 Hz), 131.7 (d, *J* = 1.7 Hz), 130.4, 128.6 (d, *J* = 7.5 Hz), 124.1 (d, *J* = 6.9 Hz), 122.8, 89.8 (dd, *J* = 172.8, 4.6 Hz), 79.8 (dd, *J* = 165.6, 4.6 Hz), 38.2 (dd, *J* = 23.8, 19.8 Hz). ^{19}F NMR (377 MHz, $CDCl_3$) δ -180.45 (ddd, *J* = 46.8, 30.9, 15.6 Hz, 1F), -222.31 – -222.75 (m, 1F). HRMS (EI) m/z calculated for $C_9H_9BrF_2$ $(M)^+$: 233.9850, found: 233.9853.

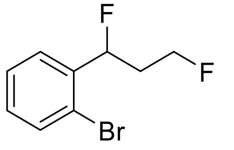

1-Bromo-2-(1,3-difluoropropyl)benzene (13). 0.5 mmol scale, 2 h. After work-up, the crude residue was purified by flash column chromatography on silica gel (petroleum ether: ethyl acetate = 200:1) to give **13** as a colorless oil. Yield = 52%, electricity = 2.4 F mol^{-1}. ^{1}H NMR (400 MHz, $CDCl_3$) δ 7.61 – 7.45 (m, 2H), 7.39 – 7.35 (m, 1H), 7.21 – 7.17 (m, 1H), 5.95 (ddd, *J* = 47.1, 9.5, 2.7 Hz, 1H), 4.85 – 4.48 (m, 2H), 2.51 – 1.97 (m, 2H). ^{13}C NMR (101 MHz, $CDCl_3$) δ 139.2 (d, *J* = 21.2 Hz), 132.9, 129.9 (d, *J* = 1.4 Hz), 128.0, 126.7 (d, *J* = 10.3 Hz), 120.6 (d, *J* = 5.9 Hz), 89.8 (dd, *J* = 173.1, 5.2 Hz), 80.0 (dd, *J* = 166.1, 2.9 Hz), 37.1 (dd, *J* = 23.6, 20.0 Hz). ^{19}F NMR (565 MHz, $CDCl_3$) δ -185.11 (ddd, *J* = 47.2, 34.8, 16.8 Hz, 1F), -222.33 (tdd, *J* = 46.9, 31.3, 17.1 Hz, 1F). HRMS (EI) m/z calculated for $C_9H_9BrF_2$ $(M)^+$: 233.9856, found: 233.9858.

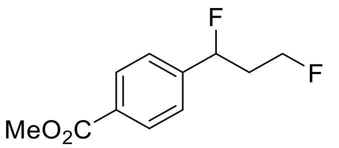

Methyl 4-(1,3-difluoropropyl)benzoate (14). 0.5 mmol scale, 6 h. After work-up, the crude residue was purified by flash column chromatography on silica gel (petroleum ether: ethyl acetate = 100:1) to give **14** as a pale yellow oil. Yield = 56%, electricity = 7.2 F mol^{-1}. ^{1}H NMR (400 MHz, $CDCl_3$) δ 8.07 (d, *J* = 8.0 Hz, 2H), 7.42 (d, *J* = 8.3 Hz, 2H), 5.81 – 5.61 (m, 1H), 4.81 – 4.44 (m, 2H), 3.92 (s, 3H), 2.35 – 2.14 (m, 2H). ^{13}C NMR (101 MHz, $CDCl_3$) δ 166.7, 144.5 (d, *J* = 19.6 Hz), 130.3 (d, *J* = 1.6 Hz), 130.0, 125.3 (d, *J* = 7.3 Hz), 90.1 (dd, *J* = 172.7, 4.6 Hz), 79.7 (dd, *J* = 165.6, 4.5 Hz), 52.3, 38.3 (dd, *J* = 23.6, 19.8 Hz). ^{19}F NMR (377 MHz, $CDCl_3$) δ -182.45 (ddd, *J* = 47.6, 30.5, 16.9 Hz, 1F), -222.51 (tdd, *J* = 46.7, 30.6, 21.4 Hz, 1F). HRMS (ESI) m/z calculated for $C_{11}H_{13}F_2O_2$ $(M+H)^+$: 215.08781, found : 215.08720.

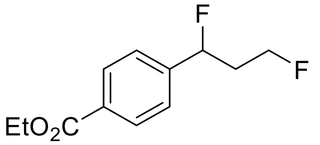

Ethyl 4-(1,3-difluoropropyl)benzoate (15).[2] 0.5 mmol scale, 6 h. After work-up, the crude residue was purified by flash column chromatography on silica gel (petroleum ether: ethyl acetate = 100:1) to give **15** as a pale yellow oil. Yield = 56%, electricity = 7.2 F mol^{-1}. ^{1}H NMR (400 MHz, $CDCl_3$) δ 8.07 (d, *J* = 8.1 Hz, 2H), 7.42 (d, *J* = 8.2 Hz, 2H), 5.84 – 5.60 (m, 1H), 4.82 – 4.43 (m, 2H), 4.39 (q, *J* = 7.1 Hz, 2H), 2.39 – 2.10 (m, 2H), 1.40 (t, *J* = 7.1 Hz, 3H). ^{13}C NMR (101 MHz, $CDCl_3$) δ 166.2 (s), 144.4 (d, *J* = 19.5 Hz), 130.7 (d, *J* = 1.5 Hz), 130.0, 125.2 (d, *J* = 7.3 Hz), 90.1 (dd, *J* = 172.7, 4.6 Hz), 79.7 (dd, *J* = 165.6, 4.5 Hz), 61.2, 38.3 (dd, *J* = 23.6, 19.8 Hz), 14.4. ^{19}F NMR (377 MHz, $CDCl_3$) δ -182.16 – -182.42 (m, 1F), -222.29 – -222.69 (m, 1F). HRMS (ESI) m/z calculated for $C_{12}H_{15}F_2O_2$ $(M+H)^+$: 229.10346, found: 229.10329.

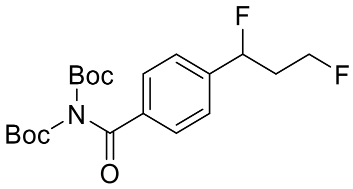

(**16**). 0.25 mmol scale, add 0.5 mL DCE, 10 h. After work-up, the crude residue was purified by flash column chromatography on silica gel (petroleum ether: ethyl acetate = 15:1) to give **16** as a white solid**.** Isolated yield = 52%, 27% starting material was recovered, electricity = 23.9 F mol^{-1}. ^{1}H NMR (400 MHz, $CDCl_3$) δ 7.86 (d, *J* = 8.1 Hz, 2H), 7.47 (d, *J* = 8.3 Hz, 2H), 5.89 – 5.58 (m, 1H), 4.87 – 4.44 (m, 2H), 2.37 – 2.11 (m, 2H), 1.39 (s, 18H). ^{13}C NMR (101 MHz, $CDCl_3$) δ 168.9, 149.8, 145.2 (d, *J* = 19.7 Hz), 134.3 (d, *J* = 1.5 Hz), 129.5, 125.5 (d, *J* = 7.4 Hz), 89.9 (dd, *J* = 173.4, 4.5 Hz), 84.5, 79.6 (dd, *J* = 165.9, 4.4 Hz), 38.3 (dd, *J* = 23.5, 19.8 Hz), 27.7. ^{19}F NMR (377 MHz, $CDCl_3$) δ -182.93 – -183.24 (m, 1F), -222.18 – -222.62 (m, 1F). HRMS (ESI) m/z calculated for $C_{20}H_{27}F_2NO_5Na$ $(M+Na)^+$: 422.17495, found: 422.17328.

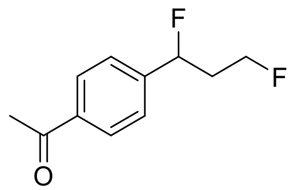

1-(4-(1,3-difluoropropyl)phenyl)ethan-1-one (17).[2] 0.5 mmol scale, 14 h. After work-up, the crude residue was purified by flash column chromatography on silica gel (petroleum ether: ethyl acetate = 30:1) to give **17** as a pale yellow oil**.** Isolated yield = 42%, electricity = 16.7 F mol^{-1}. ^{1}H NMR (400 MHz, $CDCl_3$) δ 7.99 (d, *J* = 8.0 Hz, 2H), 7.45 (d, *J* = 8.4 Hz, 2H), 5.81 – 5.62 (m, 1H), 4.82 – 4.44 (m, 2H), 2.62 (s, 3H), 2.35 – 2.15 (m, 2H). ^{13}C NMR (101 MHz, $CDCl_3$) δ 197.7, 144.7 (d, *J* = 19.6 Hz), 137.2 (d, *J* = 1.4 Hz), 128.8, 125.5 (d, *J* = 7.3 Hz), 90.0 (dd, *J* = 172.8, 4.6 Hz), 79.7 (dd, *J* = 165.6, 4.5 Hz), 38.2 (dd, *J* = 23.5, 19.8 Hz), 26.7. ^{19}F NMR (377 MHz, $CDCl_3$) δ -182.51 (ddd, *J* = 47.1, 30.3, 17.1 Hz, 1F), -222.42 (tdd, *J* = 46.9, 30.4, 20.8 Hz, 1F). HRMS (ESI) m/z calculated for $C_{11}H_{13}F_2O$ $(M+H)^+$: 199.09290, found: 199.09268.

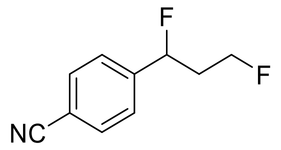

4-(1,3-difluoropropyl)benzonitrile (18).[2] 0.5 mmol scale, 20 h. After work-up, the crude residue was purified by flash column chromatography on silica gel (petroleum ether: ethyl acetate = 30:1) to give **18** as a pale yellow oil**.** Isolated yield = 49%, electricity = 23.9 F mol^{-1}. ^{1}H NMR (400 MHz, $CDCl_3$) δ 7.70 (d, *J* = 8.1 Hz, 2H), 7.47 (d, *J* = 8.4 Hz, 2H), 5.72 (ddd, *J* = 47.7, 7.9, 5.1 Hz, 1H), 4.82 – 4.46 (m, 2H), 2.33 – 2.15 (m, 2H). ^{13}C NMR (101 MHz, $CDCl_3$) δ 144.8 (d, *J* = 20.0 Hz), 132.6, 126.0 (d, *J* = 7.7 Hz), 118.5, 112.4 (d, *J* = 1.6 Hz), 89.6 (dd, *J* = 174.1, 4.4 Hz), 79.5 (dd, *J* = 166.0, 4.4 Hz), 38.2 (dd, *J* = 23.3, 19.8 Hz). ^{19}F NMR (377 MHz, $CDCl_3$) δ -183.81 – -184.07 (m, 1F), -222.27 – -222.67 (m, 1F). HRMS (ESI) m/z calculated for $C_{10}H_{10}F_2N$ $(M+H)^+$: 182.07758, found: 182.07724.

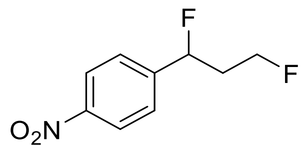

1-(1,3-Difluoropropyl)-4-nitrobenzene (19).[2] 0.5 mmol scale, 3 h. After work-up, the crude residue was purified by flash column chromatography on silica gel (petroleum ether: ethyl acetate = 30:1) to give **19** as a yellow oil**.** Yield = 49%, electricity = 3.6 F·mol^{-1}. ^{1}H NMR (400 MHz, $CDCl_3$) δ 8.26 (d, *J* = 8.3 Hz, 2H), 7.54 (d, *J* = 8.9 Hz, 2H), 5.94 – 5.64 (m, 1H), 4.83 – 4.48 (m, 2H), 2.38 – 2.17 (m, 2H). ^{13}C NMR (101 MHz, $CDCl_3$) δ 147.92, 146.72 (d, *J* = 19.9 Hz), 126.1 (d, *J* = 7.7 Hz), 124.0, 89.5 (dd, *J* = 174.4, 4.4 Hz), 79.5 (dd, *J* = 166.0, 4.4 Hz), 38.2 (dd, *J* = 23.2, 19.8 Hz). ^{19}F NMR (377 MHz, $CDCl_3$) δ -183.83 – -184.09 (m, 1F), -222.26 – -222.66 (m, 1F). HRMS (ESI) m/z calculated for $C_9H_{10}F_2NO_2$ $(M+H)^+$: 202.06741, found: 202.06697.

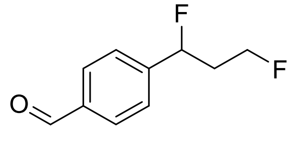

4-(1,3-Difluoropropyl)benzaldehyde (20). 0.5 mmol, 16 h. After work-up, the crude residue was purified by flash column chromatography on silica gel (petroleum ether: ethyl acetate = 100:1) to give **20** as a yellow oil. ^{1}H NMR (400 MHz, $CDCl_3$) δ 10.03 (s, 1H), 7.92 (d, *J* = 8.0 Hz, 2H), 7.53 (d, *J* = 8.1 Hz, 2H), 5.84 – 5.65 (m, 1H), 4.82 – 4.47 (m, 2H), 2.36 – 2.17 (m, 2H). ^{13}C NMR (101 MHz, $CDCl_3$) δ 191.9, 146.2 (d, *J* = 19.6 Hz), 136.5, 130.2, 125.9 (d, *J* = 7.5 Hz), 90.0 (dd, *J* = 173.4, 4.5 Hz), 79.7 (dd, *J* = 165.8, 4.4 Hz), 38.3 (dd, *J* = 23.5, 19.8 Hz). ^{19}F NMR (377 MHz, $CDCl_3$) δ -183.12 – -183.44 (m, 1F), -222.23 – -222.71 (m, 1F). HRMS (ESI) m/z calculated for $C_{10}H_{11}F_2O$ $(M+H)^+$: 185.07725, found: 185.07729.

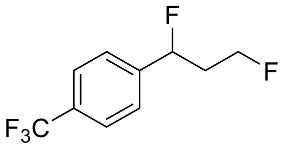

1-(1,3-Difluoropropyl)-4-(trifluoromethyl)benzene (21). 0.5 mmol scale, 2 h. After work-up, the crude residue was purified by flash column chromatography on silica gel (petroleum ether: ethyl acetate = 200:1) to give **21** as a colorless oil. Yield = 49%, electricity = 2.4 F mol^{-1}. ^{1}H NMR (400 MHz, $CDCl_3$) δ 7.66 (d, *J* = 8.3 Hz, 2H), 7.47 (d, *J* = 8.0 Hz, 2H), 5.82 – 5.60 (m, 1H), 4.83 – 4.44 (m, 2H), 2.36 – 2.14 (m, 2H). ^{13}C NMR (101 MHz, $CDCl_3$) δ 143.6 (d, *J* = 21.0 Hz),130.8 (q, *J* = 32.4 Hz), 125.8 (q, *J* = 3.6 Hz), 125.7 (d, *J* = 7.5 Hz), 124.1 (q, *J* = 272.1 Hz), 89.9 (dd, *J* = 173.0, 4.6 Hz), 79.7 (dd, *J* = 165.7, 4.5 Hz), 38.4 (dd, *J* = 23.5, 19.8 Hz). ^{19}F NMR (377 MHz, $CDCl_3$) δ -62.68 (s, 3F), -182.56 (ddd, *J* = 47.3, 30.7, 16.9 Hz, 1F), -222.59 (tdd, *J* = 46.8, 30.7, 20.9 Hz, 1F). HRMS (EI) m/z calculated for $C_{10}H_9F_5$ $(M)^+$: 224.0624, found: 224.0616.

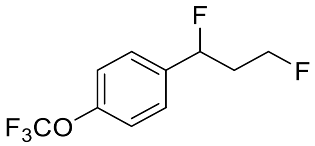

1-(1,3-Difluoropropyl)-4-(trifluoromethoxy)benzene (22). 0.25 mmol scale, add 0.5 mL DCE, 1 h. After work-up, the crude residue was purified by flash column chromatography on silica gel (30-60 °C petroleum ether: ethyl acetate = 200:1) to give **22** as a colorless oil. Isolated yield = 53%, electricity = 2.4 F mol^{-1}. ^{1}H NMR (400 MHz, $CDCl_3$) δ 7.39 (d, *J* = 8.5 Hz, 2H), 7.25 (d, *J* = 8.8 Hz, 2H), 5.67 (ddd, *J* = 47.6, 9.0, 4.1 Hz, 1H), 4.83 – 4.40 (m, 2H), 2.43 – 2.08 (m, 2H). ^{13}C NMR (101 MHz, $CDCl_3$) δ 149.4, 138.3 (d, *J* = 20.1 Hz), 127.1 (d, *J* = 6.8 Hz), 121.3, 120.6 (q, *J* =258.4 Hz), 89.9 (dd, *J* = 171.8, 4.6 Hz), 79.9 (dd, *J* = 165.5, 4.6 Hz), 38.3 (dd, *J* = 24.0, 19.8 Hz). ^{19}F NMR (377 MHz, $CDCl_3$) δ -58.03 (s, 3F), -179.09 (ddd, *J* = 46.8, 31.0, 15.0 Hz, 1F), -222.72 (tdd, *J* = 46.9, 30.6, 20.4 Hz, 1F). HRMS (EI) m/z calculated for $C_{10}H_9F_5O$ $(M)^+$: 240.0574, found: 240.0581.

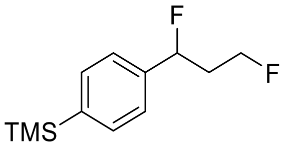

(4-(1,3-Difluoropropyl)phenyl)trimethylsilane (23). 0.25 mmol scale, add 0.5 mL DCE, 110 min. After work-up, the crude residue was purified by flash column chromatography on silica gel (petroleum ether: ethyl acetate = 200:1) to give **23** as a colorless oil. Yield = 53%, electricity = 4.4 F mol^{-1}. ^{1}H NMR (400 MHz, $CDCl_3$) δ 7.55 (d, *J* = 7.7 Hz, 2H), 7.33 (d, *J* = 7.5 Hz, 2H), 5.63 (ddd, *J* = 47.9, 9.1, 4.1 Hz, 1H), 4.84 – 4.39 (m, 2H), 2.41 – 2.07 (m, 2H), 0.27 (s, 9H). ^{13}C NMR (101 MHz, $CDCl_3$) δ 141.3 (d, *J* = 1.7 Hz), 140.0 (d, *J* = 19.4 Hz), 133.8, 124.9 (d, *J* = 6.7 Hz), 90.7 (dd, *J* = 170.7, 4.8 Hz), 80.1 (dd, *J* = 165.1, 4.6 Hz, 1F), 38.2 (dd, *J* = 24.1, 19.7 Hz, 1F), -1.0. ^{19}F NMR

(377 MHz, $CDCl_3$) δ -179.31 (ddd, *J* = 46.9, 31.3, 14.7 Hz, 1F), -222.45 (tdd, *J* = 47.0, 30.7, 20.3 Hz, 1F). HRMS (EI) m/z calculated for $C_{12}H_{18}F_2Si$ $(M)^+$: 228.1146, found: 228.1150.

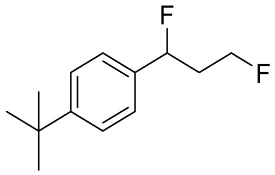

1-(Tert-butyl)-4-(1,3-difluoropropyl)benzene (24). 0.25 mmol, 40 min. After work-up, the crude residue was purified by flash column chromatography on silica gel (petroleum ether: ethyl acetate = 200:1) to give **24** as a colorless oil**.** Isolated yield = 40%, electricity = 1.6 F mol^{-1}. ^1H NMR (400 MHz, $CDCl_3$) δ 7.41 (d, *J* = 8.2 Hz, 2H), 7.29 (d, *J* = 7.4 Hz, 2H), 5.62 (ddd, *J* = 47.9, 9.2, 4.0 Hz, 1H), 4.81 – 4.40 (m, 2H), 2.43 – 2.08 (m, 2H), 1.32 (s, 9H). ^{13}C NMR (101 MHz, $CDCl_3$) δ 151.8 (d, *J* = 2.2 Hz), 136.5 (d, *J* = 19.5 Hz), 125.7, 125.5 (d, *J* = 6.3 Hz), 90.6 (dd, *J* = 169.7, 4.9 Hz), 80.2 (dd, *J* = 165.0, 4.6 Hz), 38.1 (dd, *J* = 24.3, 19.7 Hz), 34.8, 31.4. ^{19}F NMR (377 MHz, $CDCl_3$) δ -176.65 (ddd, *J* = 46.6, 31.3, 14.2 Hz, 1F), -222.49 (tdd, *J* = 46.9, 30.4, 20.0 Hz, 1F). HRMS (EI) m/z calculated for $C_{13}H_{18}F_2$ $(M)^+$: 212.1377, found: 212.1376.

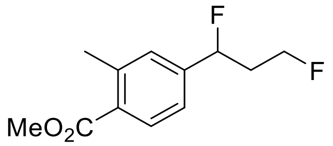

Methyl 4-(1,3-difluoropropyl)-2-methylbenzoate (25). 0.5 mmol scale, 6 h. After work-up, the crude residue was purified by flash column chromatography on silica gel (petroleum ether: ethyl acetate = 100:1) to give **25** as a colorless oil**.** Yield = 54%, electricity = 7.2 F mol^{-1}. ^1H NMR (400 MHz, $CDCl_3$) δ 7.94 (d, *J* = 8.6 Hz, 1H), 7.23 – 7.21 (m, 2H), 5.66 (ddd, *J* = 47.9, 8.5, 4.5 Hz, 1H), 4.82 – 4.43 (m, 2H), 3.90 (s, 3H), 2.62 (s, 3H), 2.34 – 2.13 (m, 2H). ^{13}C NMR (101 MHz, $CDCl_3$) δ 167.7, 143.4 (d, *J* = 19.5 Hz), 140. 9, 131.2, 129.7, 128.5 (d, *J* = 7.1 Hz), 122.6 (d, *J* = 7.1 Hz), 90.1 (dd, *J* = 172.4, 4.7 Hz), 79.9 (dd, *J* = 165.5, 4.5 Hz), 52.1, 38.3 (dd, *J* = 23.7, 19.8 Hz), 22.0. ^{19}F NMR (377 MHz, $CDCl_3$) δ -182.27 (ddd, *J* = 47.1, 30.3, 16.4 Hz, 1F), -222.51 (tdd, *J* = 46.8, 30.6, 21.0 Hz, 1F). HRMS (ESI) m/z calculated for $C_{12}H_{15}F_2O_2$ $(M+H)^+$: 229.10346, found: 229.10347.

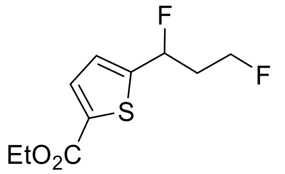

Ethyl 5-(1,3-difluoropropyl)thiophene-2-carboxylate (**26**). 0.25 mmol scale, 80 min. After work-up, the crude residue was purified by flash column chromatography on silica gel (petroleum ether: ethyl acetate = 100:1) to give **26** as a yellow oil**.** Isolated yield = 56%, electricity = 3.2 F mol^{-1}. ^1H NMR (400 MHz, $CDCl_3$) δ 7.71 – 7.69 (m, 1H), 7.13 – 6.96 (m, 1H), 5.85 (ddd, *J* = 48.0, 8.9, 4.4 Hz, 1H), 4.84 – 4.45 (m, 2H), 4.36 (q, *J* = 7.1 Hz, 2H), 2.78 – 2.20 (m, 2H), 1.38 (t, *J* = 7.1 Hz, 3H). ^{13}C NMR (101 MHz, $CDCl_3$) δ 162.0, 148.5 (d, *J* = 21.3 Hz), 134.6 (d, *J* = 2.6 Hz), 133.1 (d, *J* = 1.4 Hz), 126.2 (d, *J* = 5.8 Hz), 86.2 (dd, *J* = 171.5, 4.8 Hz), 79.5 (dd, *J* = 166.1, 4.4 Hz), 61.5, 38.0 (dd, *J* = 23.8, 19.9 Hz), 14.4. ^{19}F NMR (377 MHz, $CDCl_3$) δ -165.87 (ddd, *J* = 47.3, 29.4, 14.3 Hz, 1F), -222.88 (tdd, *J* = 46.8, 30.5, 20.7 Hz, 1F). HRMS (ESI) m/z calculated for $C_{10}H_{13}F_2O_2S$ $(M+H)^+$: 235.05988, found: 235.05981.

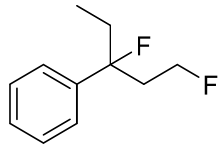

(1,3-Difluoropentan-3-yl)benzene (27).[8] 0.5 mmol scale, 2 h. After work-up, the crude residue was purified by flash column chromatography on silica gel (petroleum ether: ethyl acetate = 200:1) to give **27** as a colorless oil**.** Yield = 67%, electricity = 2.4 F mol^{-1}. ^{1}H NMR (400 MHz, $CDCl_3$) δ 7.63 – 7.24 (m, 5H), 4.64 – 4.23 (m, 2H), 2.52 – 2.21 (m, 2H), 2.12 – 1.85 (m, 2H), 0.79 (t, *J* = 7.4 Hz, 3H). ^{13}C NMR (101 MHz, $CDCl_3$) δ 141.9 (d, *J* = 22.1 Hz), 128.4 (d, *J* = 1.9 Hz), 127.3 (d, *J* = 0.9 Hz), 124.4 (d, *J* = 10.5 Hz), 98.5 (dd, *J* = 176.3, 7.1 Hz), 80.1 (dd, *J* = 163.8, 5.1 Hz), 40.8 (dd, *J* = 23.1, 19.7 Hz), 33.8 (d, *J* = 23.9 Hz), 7.5 (d, *J* = 4.9 Hz). ^{19}F NMR (377 MHz, $CDCl_3$) δ -162.42 (tt, *J* = 30.3, 16.0 Hz, 1F), -220.33 (tt, *J* = 47.0, 20.3 Hz, 1F). HRMS (EI) m/z calculated for $C_{11}H_{14}F_2$ $(M)^+$: 184.1058, found: 184.1057.

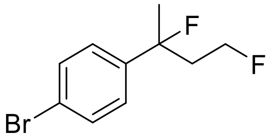

1-Bromo-4-(2,4-difluorobutan-2-yl)benzene (28).[8] 0.5 mmol scale, 2 h. After work-up, the crude residue was purified by flash column chromatography on silica gel (petroleum ether: ethyl acetate = 200:1) to give **28** as a colorless oil. Yield = 65%, electricity = 2.4 F mol^{-1}. ^{1}H NMR (400 MHz, $CDCl_3$) δ 7.50 (d, *J* = 8.3 Hz, 2H), 7.23 (d, *J* = 8.5 Hz, 2H), 4.67 – 4.30 (m, 2H), 2.45 – 2.20 (m, 2H), 1.70 (d, *J* = 22.7 Hz, 3H). ^{13}C NMR (101 MHz, $CDCl_3$) δ 143.1 (d, *J* = 22.2 Hz), 131.7 (d, *J* = 1.4 Hz), 125.9 (d, *J* = 9.8 Hz), 121.7 (d, *J* = 1.6 Hz), 96.0 (dd, *J* = 173.7, 5.4 Hz), 79.8 (dd, *J* = 165.0, 5.2 Hz), 42.3 (dd, *J* = 23.8, 19.6 Hz), 27.8 (d, *J* = 24.9 Hz). ^{19}F NMR (377 MHz, $CDCl_3$) δ -148.18 (d, *J* = 2.3 Hz), -219.84 (d, *J* = 2.3 Hz). ^{19}F NMR (565 MHz, Chloroform-*d*) δ -148.13 (tq, *J* = 22.4, 22.4 Hz, 1F), -219.83 (tt, *J* = 46.2, 22.8 Hz, 1F). HRMS (EI) m/z calculated for $C_{10}H_{11}BrF_2$ $(M)^+$: 248.0007, found: 248.0011.

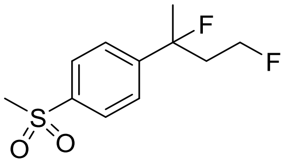

1-(2,4-Difluorobutan-2-yl)-4-(methylsulfonyl)benzene (**29**). 0.25 mmol scale, 100 min. After work-up, the crude residue was purified by flash column chromatography on silica gel (petroleum ether: ethyl acetate = 3:1) to give **29** as a yellow oil**.** Yield = 81%, electricity = 4 F mol^{-1}. ^{1}H NMR (400 MHz, $CDCl_3$) δ 7.96 (d, *J* = 8.2 Hz, 2H), 7.57 (d, *J* = 8.5 Hz, 2H), 4.70 – 4.33 (m, 2H), 3.08 (s, 3H), 2.54 – 2.22 (m, 2H), 1.75 (d, *J* = 22.7 Hz, 3H). ^{13}C NMR (101 MHz, $CDCl_3$) δ 150.2 (d, *J* = 22.0 Hz), 139.8, 127.7 (d, *J* = 1.7 Hz), 125.1 (d, *J* = 10.1 Hz), 95.9 (dd, *J* = 175.7, 4.6 Hz), 79.5 (dd, *J* = 165.7, 5.1 Hz), 44.5, 42.1 (dd, *J* = 23.5, 19.7 Hz), 27.8 (d, *J* = 24.9 Hz). ^{19}F NMR (377 MHz, $CDCl_3$) δ -149.45 (tq, *J* = 22.6, 22.6 Hz, 1F), -219.40 (tt, *J* = 46.9, 23.7 Hz, 1F). HRMS (ESI) m/z calculated for $C_{11}H_{15}F_2O_2S$ $(M+H)^+$: 249.07553, found: 249.07550.

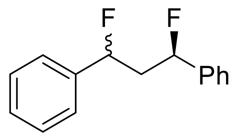

1,3-Difluoro-1,3-diphenylpropane (**30**). 0.5 mmol scale, 2 h. After work-up, the crude residue was purified by flash column chromatography on silica gel (petroleum ether: ethyl acetate = 200:1) to give **30** as a colorless oil with 1.3:1 diastereoselectivity. Isolated yield = 61%, electricity = 2.4 F mol^{-1}. ^{1}H, ^{13}C, ^{19}F spectra are obtained from diastereoisomer mixture. ^{1}H NMR (400 MHz, $CDCl_3$) δ 7.41 – 7.30 (m, 23.2 H), 5.89 – 5.71 (m, 2.6 H), 5.43 (ddd, *J* = 47.1, 7.8, 6.2 Hz, 2H), 2.90 – 2.75 (m, 1H), 2.44 – 2.15 (m, 3.8 H). ^{13}C NMR (101 MHz, $CDCl_3$) δ 139.7 (d, *J* = 19.4 Hz), 139.1 (d, *J* = 19.5 Hz), 128.9 (d, *J* = 2.1 Hz), 128.8, 128.7, 128.7 (d, *J* = 1.0 Hz), 126.0 (d, *J* = 6.5 Hz), 125.6 (d, *J* = 6.9 Hz), 91.7 (dd, J = 170.2, 6.5 Hz), 90.6 (dd, *J* = 171.1, 2.3 Hz), 45.6 (t, *J* = 23.4 Hz) 44.4 (t, J = 24.4 Hz). ^{19}F NMR (377 MHz, $CDCl_3$) δ -174.37 (ddd, *J* = 47.2, 25.4, 11.6 Hz, 2F), -178.11 – -178.40 (m, 2.6F). HRMS (EI) m/z calculated for $C_{15}H_{14}F_2$ $(M)^+$: 232.1064, found: 232.1059.

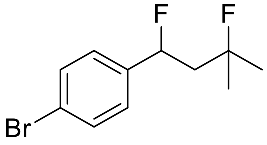

1-Bromo-4-(1,3-difluoro-3-methylbutyl)benzene (31). 0.25 mmol scale, 1 h. After After work-up, the crude residue was purified by flash column chromatography on silica gel (petroleum ether : ethyl acetate = 200:1) to give **31** as a colorless oil. Isolated **y**ield = 81%, electricity = 2.4 F mol^{-1}. ^{1}H NMR (400 MHz, $CDCl_3$) δ 7.58 – 7.49 (m, 2H), 7.22 (d, *J* = 8.3 Hz, 2H), 5.68 (ddd, *J* = 48.4, 9.5, 2.0 Hz, 1H), 2.59 – 1.93 (m, 2H), 1.52 (dd, *J* = 21.7, 1.3 Hz, 3H), 1.43 (d, *J* = 21.7 Hz, 3H). ^{13}C NMR (101 MHz, $CDCl_3$) δ 139.7 (d, *J* = 20.4 Hz), 131.8 (d, *J* = 5.0 Hz), 127.2 (d, *J* = 6.9 Hz), 122.4 (d, *J* = 2.4 Hz), 94.4 (d, *J* = 166.5 Hz), 90.4 (dd, *J* = 171.8, 5.8 Hz), 48.7 (t, *J* = 22.8 Hz), 28.6 (dd, *J* = 24.0, 1.3 Hz), 26.4 (dd, *J* = 24.8, 3.0 Hz). ^{19}F NMR (377 MHz, $CDCl_3$) δ -136.53 – -137.06 (m, 1F), -173.30 (ddd, *J* = 48.2, 37.5, 18.3 Hz, 1F). HRMS (EI) m/z calculated for $C_{11}H_{13}BrF_2$ $(M)^+$: 262.0163, found: 262.0159.

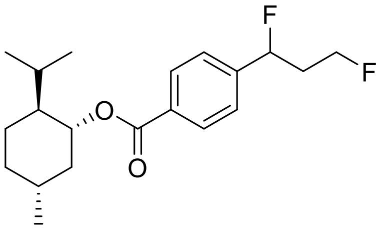

(1R, 2S, 5R)-2-isopropyl-5-methylcyclohexyl 4-(1,3-difluoropropyl)benzoate (**32**). 0.1 mmol scale, add 0.5 ml DCE, 2 h. After work-up, the crude residue was purified by flash column chromatography on silica gel (petroleum ether : ethyl acetate = 150:1) to give **32** as yellow oil with 1:1 diastereoselectivity. Isolated yield = 86%, electricity = 11.9 F mol^{-1}. ^{1}H, ^{13}C, ^{19}F spectra are obtained from diastereoisomer mixture. ^{1}H NMR (400 MHz, $CDCl_3$) δ 8.08 (d, *J* = 8.0 Hz, 2H), 7.42 (d, *J* = 8.3 Hz, 2H), 5.71 (ddd, *J* = 47.8, 8.5, 4.5 Hz, 1H), 4.94 (td, *J* = 10.9, 4.4 Hz, 1H), 4.81 – 4.44 (m, 2H), 2.40 – 2.15 (m, 2H), 2.15 – 2.09 (m, 1H), 1.99 – 1.91 (m, 1H), 1.79 – 1.70 (m, 2H), 1.63 – 1.50 (m, 2H), 1.18 – 1.05 (m, 2H), 0.94 – 0.91 (m, 7H), 0.80 (d, *J* = 7.0 Hz, 3H). ^{13}C NMR (101 MHz, $CDCl_3$) δ 165.7, 144.3 (d, *J* = 19.6 Hz), 131.1, 130.0, 125.3 (d, *J* = 7.2 Hz), 90.1 (dd, *J* = 172.7, 4.6 Hz), 79.8 (dd, *J* = 165.6, 4.5 Hz), 75.1, 47.4, 41.1, 38.3 (dd, *J* = 23.6, 19.8 Hz), 34.4,

31.6, 26.6, 23.7, 22.2, 20.9, 16.6. ^{19}F NMR (377 MHz, $CDCl_3$) δ -182.04 – -182.36 (m, 1F), -222.24 – -222.68 (m, 1F). HRMS (ESI) m/z calculated for $C_{20}H_{38}F_2O_2Na$ $(M+Na)^+$: 361.19496, found: 361.19437.

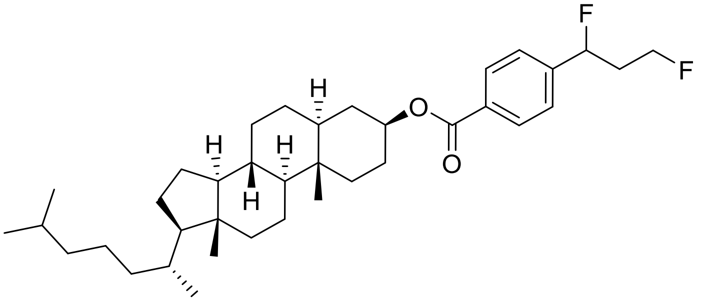

(**33**). 0.1 mmol scale, add 0.5 mL DCE, 4 h. After work-up, the crude residue was purified by flash column chromatography on silica gel (petroleum ether: ethyl acetate = 100:1) to give **33** as white solid with 1:1 diastereoselectivity. Isolated yield = 65%, 10% starting material was recovered, electricity = 23.8 F mol^{-1}. ^{1}H, ^{13}C, ^{19}F spectra are obtained from diastereoisomer mixture. ^{1}H NMR (400 MHz, $CDCl_3$) δ 8.06 (d, *J* = 8.1 Hz, 2H), 7.40 (d, *J* = 8.3 Hz, 2H), 5.71 (ddd, *J* = 47.7, 8.3, 4.5 Hz, 1H), 5.03 – 4.87 (m, 1H), 4.81 – 4.42 (m, 2H), 2.38 – 2.09 (m, 2H), 2.02 – 1.89 (m, 2H), 1.85 – 0.99 (m, 28H), 0.92 – 0.84 (m, 12H), 0.73 – 0.63 (m, 4H). ^{13}C NMR (101 MHz, $CDCl_3$) δ 165.7, 144.2 (d, *J* = 19.5 Hz), 131.2, 130.0, 125.2 (d, *J* = 7.2 Hz), 90.1 (dd, *J* = 172.7, 4.5 Hz), 79.8 (dd, *J* = 165.7, 4.5 Hz), 74.7, 56.5, 56.4, 54.3, 44.8, 42.7, 40.1, 39.6, 38.4 (dd, *J* = 23.6, 19.8 Hz), 36.9, 36.3, 35.9, 35.6(3), 35.5(9), 34.2, 32.1, 28.8, 28.4, 28.1, 27.7, 24.3, 24.0, 23.0, 22.7, 21.4, 18.8, 12.4, 12.2. ^{19}F NMR (377 MHz, $CDCl_3$) δ -182.22 (ddd, *J* = 47.1, 30.0, 16.5 Hz, 1F), -222.45 (tdd, *J* = 46.8, 30.3, 21.0 Hz, 1F). HRMS (DART POSITIVE) m/z calculated for $C_{37}H_{60}F_2NO_2$ $(M+NH_4)^+$: 588.4587, found: 588.4589.

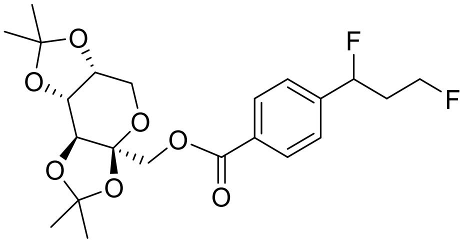

(34). 0.1 mmol scale, add 0.5 mL DCE, 6 h. After work-up, the crude residue was purified by flash column chromatography on silica gel (petroleum ether: ethyl acetate = 8:1) to give **34** as colorless oil with 1:1 diastereoselectivity. Isolated yield = 82%, electricity = 35.8 F mol^{-1}. ^{1}H, ^{13}C, ^{19}F spectra are obtained from diastereoisomer mixture. ^{1}H, ^{13}C, ^{19}F spectra are obtained from diastereoisomer mixture. ^{1}H NMR (400 MHz, $CDCl_3$) δ 8.11 (d, *J* = 8.1 Hz, 2H), 7.43 (d, *J* = 8.3 Hz, 2H), 5.83 – 5.60 (m, 1H), 4.81 – 4.44 (m, 5H), 4.36 – 4.26 (m, 2H), 3.99 – 3.79 (m, 2H), 2.40 – 2.12 (m, 2H), 1.55 (s, 3H), 1.47 (s, 3H), 1.38 (s, 3H), 1.35 (s, 3H). ^{13}C NMR (101 MHz, $CDCl_3$) δ 165.6, 144.7 (d, *J* = 19.6 Hz), 130.2, 130.1, 125.3 (d, *J* = 7.3 Hz), 109.2, 108.9, 101.7, 90.0 (dd, J = 172.9, 4.6 Hz), 79.7 (dd, J = 165.7, 4.5 Hz), 70.8, 70.6, 70.1, 65.5, 61.4, 38.3 (dd, *J* = 23.5, 19.8 Hz), 26.6, 25.9, 25.6, 24.1. ^{19}F NMR (377 MHz, $CDCl_3$) δ -182.43 – -182.69 (m, 1F), -222.43 (tdd, *J* = 47.0, 29.8, 21.5 Hz, 1F). HRMS (ESI) m/z calculated for $C_{22}H_{29}F_2O_7$ $(M+H)^+$: 443.18759, found: 443.18588.

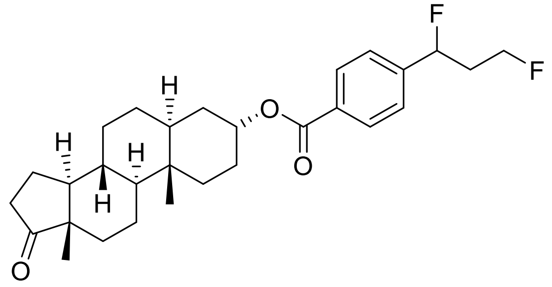

(3R, 5S, 8R, 9S, 10S, 13S, 14S)-10,13-dimethyl-17-oxohexadecahydro-1H-cyclopenta[a]phenanthren-3-yl 4-(1,3-difluoropropyl)benzoate (35). 0.1 mmol scale, add 0.5 mL DCE, 140 min. After work-up, the crude residue was purified by flash column chromatography on silica gel (petroleum ether: ethyl acetate = 15:1) to give **35** as white solid with 1:1 diastereoselectivity. Yield = 30%, 36% starting material was recovered, electricity = 13.9 F mol^{-1}. ^{1}H, ^{13}C, ^{19}F spectra are obtained from diastereoisomer mixture. ^{1}H, ^{13}C, ^{19}F spectra are obtained from diastereoisomer mixture. ^{1}H NMR (400 MHz, $CDCl_3$) δ 8.06 (d, *J* = 8.1 Hz, 2H), 7.41 (d, *J* = 8.2 Hz, 2H), 5.71 (ddd, *J* = 47.7, 8.3, 4.5 Hz, 1H), 5.01 – 4.89 (m, 1H), 4.82 – 4.43 (m, 2H), 2.48 – 2.41 (m, 1H), 2.35 – 2.14 (m, 2H), 2.13 – 2.02 (m, 1H), 2.00 – 1.90 (m, 2H), 1.85 – 1.73 (m, 4H), 1.72 – 1.47 (m, 5H), 1.36 – 1.23 (m, 6H), 1.17 – 0.98 (m, 2H), 0.91 (s, 3H), 0.87 (s, 3H), 0.79 – 0.71 (m, 1H). ^{13}C NMR (101 MHz, $CDCl_3$) δ 221.4, 165.7, 144.3 (d, *J* = 19.6 Hz), 131.1, 130.0, 125.2 (d, *J* = 7.2 Hz), 90.1 (dd, *J* = 172.7, 4.6 Hz), 79.8 (dd, *J* = 165.6, 4.5 Hz), 74.4, 54.4, 51. 5, 47.9, 44.8, 38.3 (dd, *J* = 23.6, 19.7 Hz), 36.8, 36.0, 35.8, 35.1, 34.1, 31.6, 30.9, 28.4, 27.6, 21.9, 20.6, 13.9, 12.4. ^{19}F NMR (377 MHz, $CDCl_3$) δ -182.08 – -182.40 (m, 1F), -222.47 (tdd, *J* = 46.9, 30.6, 20.9 Hz, 1F). HRMS (ESI) m/z calculated for $C_{29}H_{39}F_2O_3$ $(M+H)^+$: 473.28618, found: 473.28513.

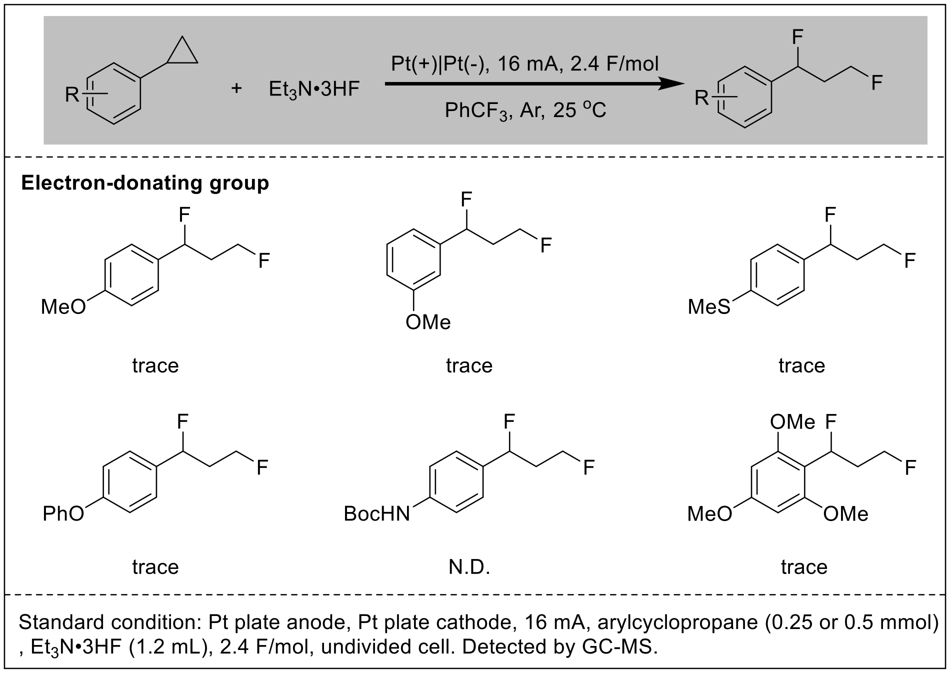

Supplementary Figure 3 unsuccessful substrates

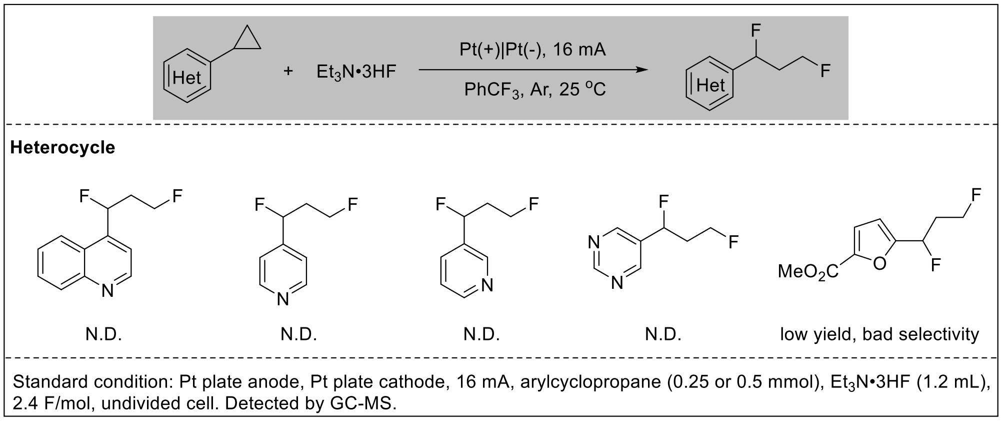

Supplementary Figure 4 unsuccessful substrates

5) Procedures and analytical data of 1,3-oxyfluorination compounds

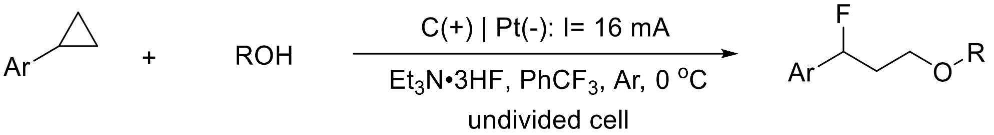

An oven-dried undivided three-necked bottle equipped with a stir bar. The bottle was equipped carbon cloth (15 mm×15 mm) as the anode and platinum plate (15 mm×15 mm×0.3 mm) as the cathode and then charged with argon gas by glove box. Arylcyclopropane (0.25 mmol), ROH (0.2-0.75 mL), $Et_3N{\cdot}3HF$ (0.8 mL) and $PhCF_3$ (4.8 mL) were added. The reaction mixture was stirred and electrolyzed at a constant current of 16 mA under 0 °C. The reaction was diluted with water. The organic layer was extracted with CH_2Cl_2, dried with anhydrous Na_2SO_4, filtered, and concentrated under reduced pressure. The pure product was obtained by flash column chromatography on silica gel.

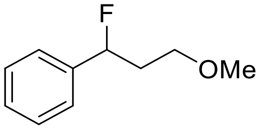

(1-fluoro-3-methoxypropyl)benzene (**3**). 0.25 mmol scale, 0.2 mL MeOH, 1 h. After work-up, the crude residue was purified by flash column chromatography on silica gel (petroleum ether: ethyl acetate = 150:1) to give **33** as colorless oil**.** Yield = 76%, electricity = 2.4 F mol^{-1}. ^{1}H NMR (400 MHz, $CDCl_3$) δ 7.44 – 7.28 (m, 5H), 5.62 (ddd, *J* = 47.9, 9.0, 4.3 Hz, 1H), 3.63 – 3.39 (m, 2H), 3.37 (s, 3H), 2.30 – 1.96 (m, 2H). ^{13}C NMR (101 MHz, $CDCl_3$) δ 140.2 (d, *J* = 19.6 Hz), 128.6, 128.5 (d, *J* = 2.0 Hz), 125.7 (d, *J* = 6.8 Hz), 91. 8 (d, *J* = 169.7 Hz), 68.5 (d, *J* = 4.7 Hz), 58.9, 37.6 (d, *J* = 23.9 Hz). ^{19}F NMR (377 MHz, $CDCl_3$) δ -177.57 (ddd, *J* = 47.4, 31.6, 14.6 Hz, 1F). HRMS (ESI) m/z calculated for $C_{10}H_{13}FONa$ $(M+Na)^+$:191.08426, found: 191.08354.

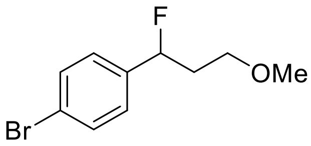

1-Bromo-4-(1-fluoro-3-methoxypropyl)benzene (36). 0.25 mmol scale, 0.2 mL MeOH, 100 min. After work-up, the crude residue was purified by flash column chromatography on silica gel (petroleum ether: ethyl acetate = 150:1) to give **36** as colorless oil**.** Yield = 50%, electricity = 4.0 F mol^{-1}. ^{1}H NMR (400 MHz, $CDCl_3$) δ 7.54 (d, *J* = 8.1 Hz, 2H), 7.25 (d, *J* = 8.2 Hz, 2H), 5.62 (ddd, *J* = 47.7, 8.9, 4.3 Hz, 1H), 3.68 – 3.36 (m, 5H), 2.29 – 1.94 (m, 2H). ^{13}C NMR (101 MHz, $CDCl_3$) δ 139.3 (d, *J* = 20.0 Hz), 131.8, 127.3 (d, *J* = 6.9 Hz), 122.3 (d, *J* = 2.4 Hz), 91.1 (d, *J* = 170.7 Hz), 68.2 (d, *J* = 4.8 Hz), 58.9, 37.5 (d, *J* = 23.8 Hz). ^{19}F NMR (377 MHz, $CDCl_3$) δ -178.50 (ddd, *J* = 46.8, 31.2, 14.6 Hz). HRMS (ESI) m/z calculated for $C_{10}H_{12}BrFONa$ $(M+Na)^+$: 268.99478, found: 268.99551.

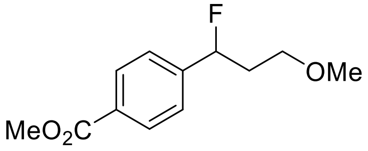

Methyl 4-(1-fluoro-3-methoxypropyl)benzoate (37). 0.25 mmol scale, 0.2 mL MeOH, 1 h. After work-up, the crude residue was purified by flash column chromatography on silica gel (petroleum ether : ethyl acetate = 30:1) to give **37** as colorless oil**.** Yield = 69%, electricity = 2.4 F mol^{-1}. ^{1}H NMR (400 MHz, $CDCl_3$) δ 8.07 (d, *J* = 8.1 Hz, 2H), 7.43 (d, *J* = 8.3 Hz, 2H), 5.70 (ddd, *J* = 47.9, 8.9, 4.2 Hz, 1H), 3.94 (s, 3H), 3.67 – 3.34 (m, 5H), 2.33 – 1.95 (m, 2H). ^{13}C NMR (101 MHz, $CDCl_3$) δ 166.8, 145.3 (d, *J* = 19.7 Hz), 130.0 (d, *J* = 1.5 Hz), 129.9, 125.3 (d, *J* = 7.3 Hz), 91.1 (d, *J* = 171.6 Hz), 68.1 (d, *J* = 4.6 Hz), 58.9, 52.3, 37.6 (d, *J* = 23.4 Hz). ^{19}F NMR (377 MHz, $CDCl_3$) δ -181.46 (ddd, *J* = 47.2, 31.5, 15.2 Hz, 1F). HRMS (ESI) m/z calculated for $C_{12}H_{15}FO_3Na$ $(M+Na)^+$: 249.08974, found: 249.08956.

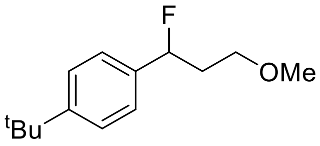

1-(Tert-butyl)-4-(1-fluoro-3-methoxypropyl)benzene (38). 0.25 mmol scale, 0.2 mL MeOH, 1 h. After work-up, the crude residue was purified by flash column chromatography on silica gel (petroleum ether : ethyl acetate = 150:1) to give **38** as colorless oil**.** Yield = 49%, electricity = 2.4 F mol^{-1}. ^{1}H NMR (400 MHz, $CDCl_3$) δ 7.40 (d, *J* = 8.5 Hz, 2H), 7.31 – 7.26 (m, 2H), 5.59 (ddd, *J* = 48.0, 9.1, 4.2 Hz, 1H), 3.64 – 3.42 (m, 2H), 3.37 (s, 3H), 2.32 – 1.95 (m, 2H), 1.32 (s, 9H). ^{13}C NMR (101 MHz, $CDCl_3$) δ 151.5 (d, *J* = 2.1 Hz), 137.2 (d, *J* = 19.6 Hz), 125.6 (d, *J* = 3.2 Hz), 125.5 (d, *J* = 3.2 Hz), 91.7 (d, *J* = 168.6 Hz), 68.6 (d, *J* = 4.5 Hz), 58.9, 37.4 (d, *J* = 24.1 Hz), 34.7, 31.5. ^{19}F NMR (376 MHz, $CDCl_3$) δ -175.43 – -175.76 (m, 1F). HRMS (ESI) m/z calculated for $C_{14}H_{21}FONa$ $(M+Na)^+$: 247.14686, found: 247.14703.

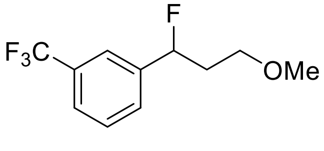

1-(1-Fluoro-3-methoxypropyl)-3-(trifluoromethyl)benzene (39). 0.25 mmol scale, 0.2 mL MeOH, 1 h. After work-up, the crude residue was purified by flash column chromatography on silica gel (petroleum ether : ethyl acetate = 100:1) to give **39** as colorless oil**.** Yield = 53%, electricity = 2.4 F mol^{-1}. ^{1}H NMR (600 MHz, $CDCl_3$) δ 7.53 (s, 1H), 7.50 (d, *J* = 7.5 Hz, 1H), 7.45

– 7.39 (m, 2H), 5.60 (ddd, *J* = 47.6, 9.0, 4.2 Hz, 1H), 3.52 (ddd, *J* = 9.3, 8.3, 4.8 Hz, 1H), 3.34 (dt, *J* = 9.4, 5.4 Hz, 1H), 3.28 (s, 3H), 2.16 – 2.08 (m, 1H), 2.03 – 1.90 (m, 1H). ^{13}C NMR (151 MHz, $CDCl_3$) δ 141.42 (d, *J* = 20.2 Hz), 131.08 (q, *J* = 32.5 Hz), 129.16, 128.87 (d, *J* = 6.9 Hz), 125.19 (q, *J* = 4.1 Hz), 124.13 (q, *J* = 272.3 Hz), 122.39 (dq, *J* = 7.6, 3.8 Hz), 90.99 (d, *J* = 171.7 Hz), 68.09 (d, *J* = 4.5 Hz), 58.90, 37.69 (d, *J* = 23.4 Hz). ^{19}F NMR (565 MHz, $CDCl_3$) δ -62.74, -180.35 (ddd, *J* = 47.1, 31.7, 14.6 Hz). HRMS (ESI) m/z calculated for $C_{11}H_{12}F_4ONa$ $(M+Na)^+$: 259.07165, found: 259.07111.

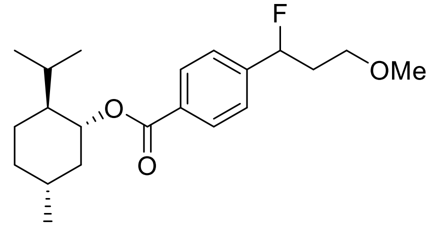

(1R,2S,5R)-2-isopropyl-5-methylcyclohexyl 4-(1-fluoro-3-methoxypropyl)benzoate (40). 0.25 mmol scale, 0.2 mL MeOH, 2 h. After work-up, the crude residue was purified by flash column chromatography on silica gel (petroleum ether: ethyl acetate = 30:1) to give **40** with 1:1 diastereoselectivity. Yield = 56%, electricity = 2.4 F mol^{-1}. ^{1}H NMR (400 MHz, $CDCl_3$) δ 8.06 (d, *J* = 8.0 Hz, 2H), 7.41 (d, *J* = 8.1 Hz, 2H), 5.69 (ddd, *J* = 47.9, 8.9, 4.1 Hz, 1H), 4.94 (td, *J* = 10.9, 4.3 Hz, 1H), 3.69 – 3.14 (m, 5H), 2.27 – 1.91 (m, 4H), 1.73 (d, *J* = 12.9 Hz, 2H), 1.61 – 1.49 (m, 2H), 1.13 (dd, *J* = 24.6, 12.4 Hz, 2H), 0.97 – 0.86 (m, 7H), 0.79 (d, *J* = 6.9 Hz, 3H). ^{13}C NMR (101 MHz, $CDCl_3$) δ 165.8, 145.1 (d, *J* = 19.7 Hz), 130.8 (d, *J* = 0.9 Hz), 129.9, 125.3 (dd, *J* = 7.3, 1.6 Hz), 91.2 (d, *J* = 171.6 Hz), 75.0, 68.2 (d, *J* = 4.6 Hz), 58.9, 47.4, 41.1, 37.7 (d, *J* = 23.5 Hz), 34.4, 31.6, 26.6, 23.7, 22.2, 20.9, 16.6. ^{19}F NMR (377 MHz, $CDCl_3$) δ -181.10 – 181.44 (m, 1F). HRMS (ESI) m/z calculated for $C_{21}H_{31}FO_3Na$ $(M+Na)^+$: 373.21494, found: 373.21467.

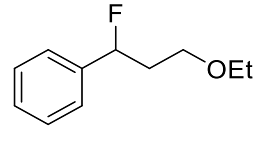

(3-Ethoxy-1-fluoropropyl)benzene (41). 0.25 mmol scale, 0.3 mL EtOH, 1 h. After work-up, the crude residue was purified by flash column chromatography on silica gel (petroleum ether: ethyl acetate = 150:1) to give **41** as colorless oil. Yield = 50%, electricity = 2.4 F mol^{-1}. ^{1}H NMR (400 MHz, $CDCl_3$) δ 7.46 – 7.27 (m, 5H), 5.63 (ddd, *J* = 47.9, 8.9, 4.4 Hz, 1H), 3.67 – 3.43 (m, 4H), 2.31 – 1.95 (m, 2H), 1.21 (t, *J* = 7.0 Hz, 3H). ^{13}C NMR (101 MHz, $CDCl_3$) δ 140.3 (d, *J* = 19.5 Hz), 128.6, 128.4 (d, *J* = 1.9 Hz), 125.7 (d, *J* = 6.8 Hz), 91.9 (d, *J* = 169.5 Hz), 66.5, 66.2 (d, *J* = 4.9 Hz), 37.7 (d, *J* = 23.8 Hz), 15.3. ^{19}F NMR (377 MHz, $CDCl_3$) δ -177.45 (ddd, *J* = 46.7, 31.1, 14.6 Hz, 1F). HRMS (ESI) m/z calculated for $C_{11}H_{15}FONa$ $(M+Na)^+$: 205.09991, found: 205.09956.

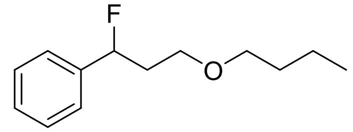

(3-Butoxy-1-fluoropropyl)benzene (42). 0.25 mmol scale, 0.45 mL *n*-BuOH, 1 h. After work-up, the crude residue was purified by flash column chromatography on silica gel (petroleum ether: ethyl acetate = 150:1) to give **42** as colorless oil. Yield = 63%, electricity = 2.4 F mol^{-1}. ^{1}H NMR (400

MHz, $CDCl_3$) δ 7.47 – 7.32 (m, 5H), 5.67 (ddd, *J* = 47.9, 8.8, 4.4 Hz, 1H), 3.69 – 3.45 (m, 4H), 2.36 – 1.99 (m, 2H), 1.65 – 1.57 (m, 2H), 1.50 – 1.38 (m, 2H), 0.97 (t, *J* = 7.3 Hz, 3H). ^{13}C NMR (101 MHz, $CDCl_3$) δ 140.2 (d, *J* = 19.6 Hz), 128.5, 128.3 (d, *J* = 1.9 Hz), 125.6 (d, *J* = 6.7 Hz), 91.8 (d, *J* = 169.4 Hz), 71.0, 66.3 (d, *J* = 5.0 Hz), 37.6 (d, *J* = 23.8 Hz), 31.8, 19.4, 14.0. ^{19}F NMR (377 MHz, $CDCl_3$) δ -177.33 (ddd, *J* = 46.6, 31.1, 14.6 Hz, 1F). HRMS (ESI) m/z calculated for $C_{13}H_{19}FONa$ $(M+Na)^+$: 233.13121, found: 233.13198.

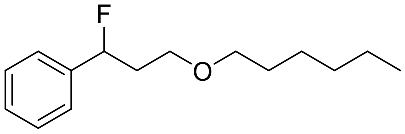

(1-Fluoro-3-(hexyloxy)propyl)benzene (43). 0.25 mmol scale, 0.6 mL 1-hexanol, 1 h. After work-up, the crude residue was purified by flash column chromatography on silica gel (petroleum ether : ethyl acetate = 150:1) to give **43** as colorless oil. Yield = 69%, electricity = 2.4 F mol^{-1}. ^{1}H NMR (400 MHz, $CDCl_3$) δ 7.44 – 7.28 (m, 5H), 5.62 (ddd, *J* = 47.9, 8.8, 4.4 Hz, 1H), 3.65 – 3.39 (m, 4H), 2.31 – 1.95 (m, 2H), 1.63 – 1.53 (m, 2H), 1.36 – 1.26 (m, 6H), 0.93 – 0.87 (m, 3H). ^{13}C NMR (101 MHz, $CDCl_3$) δ 140.3 (d, *J* = 19.6 Hz), 128.6, 128.4 (d, *J* = 1.9 Hz), 125.7 (d, *J* = 6.7 Hz), 91.9 (d, *J* = 169.4 Hz), 71.4, 66.4 (d, *J* = 5.0 Hz), 37.7 (d, *J* = 23.8 Hz), 31.8, 29.8, 26.0, 22.8, 14.2. ^{19}F NMR (377 MHz, $CDCl_3$) δ -177.27 (ddd, *J* = 46.4, 30.8, 14.5 Hz, 1F). HRMS (ESI) m/z calculated for $C_{15}H_{23}FONa$ $(M+Na)^+$: 261.16251, found: 261.16197.

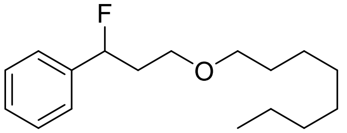

(1-Fluoro-3-(octyloxy)propyl)benzene (44). 0.25 mmol scale, 0.75 mL octanol, 1 h. After work-up, the crude residue was purified by flash column chromatography on silica gel (petroleum ether : ethyl acetate = 150:1) to give **44** as colorless oil. Yield = 59%, electricity = 2.4 F mol^{-1}. ^{1}H NMR (400 MHz, $CDCl_3$) δ 7.48 – 7.31 (m, 5H), 5.68 (ddd, *J* = 47.9, 8.8, 4.4 Hz, 1H), 3.73 – 3.43 (m, 4H), 2.38 – 2.00 (m, 2H), 1.67 – 1.58 (m, 2H), 1.39 – 1.27 (m, 10H), 0.96 – 0.92 (m, *J* = 9.0, 4.8 Hz, 3H). ^{13}C NMR (101 MHz, $CDCl_3$) δ 140.3 (d, *J* = 19.5 Hz), 128.6, 128.4 (d, *J* = 1.9 Hz), 125.7 (d, *J* = 6.7 Hz), 91.9 (d, *J* = 169.5 Hz), 71.4, 66.4 (d, *J* = 5.0 Hz), 37.7 (d, *J* = 23.8 Hz), 32.0, 29.8, 29.6, 29.4, 26.3, 22.8, 14.3. ^{19}F NMR (377 MHz, $CDCl_3$) δ -177.29 (ddd, *J* = 46.5, 31.0, 14.6 Hz, 1F). HRMS (ESI) m/z calculated for $C_{17}H_{27}FONa$ $(M+Na)^+$: 289.19381, found: 289.19352.

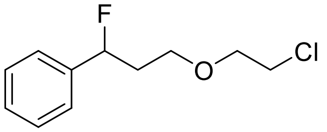

(3-(2-Chloroethoxy)-1-fluoropropyl)benzene (45). 0.25 mmol scale, 0.3 mL $ClCH_2CH_2OH$, 1 h. After work-up, the crude residue was purified by flash column chromatography on silica gel (petroleum ether : ethyl acetate = 150:1) to give **45** as colorless oil. Yield = 40%, electricity = 2.4 F mol^{-1}. ^{1}H NMR (400 MHz, $CDCl_3$) δ 7.49 – 7.33 (m, 5H), 5.70 (ddd, *J* = 47.9, 8.9, 4.4 Hz, 1H), 3.81 – 3.55 (m, 6H), 2.37 – 2.03 (m, 2H). ^{13}C NMR (101 MHz, $CDCl_3$) δ 140.0 (d, *J* = 19.5 Hz), 128.5, 128.4 (d, *J* = 2.0 Hz), 125.6 (d, *J* = 6.8 Hz), 91.6 (d, *J* = 169.6 Hz), 71.1, 66.8 (d, *J* = 5.0 Hz), 42.9, 37.4 (d, *J* = 24.0 Hz). ^{19}F NMR (377 MHz, $CDCl_3$) δ -177.65 (ddd, *J* = 46.6, 30.9, 14.4 Hz, 1F). HRMS (ESI) m/z calculated for $C_{11}H_{14}FClONa$ $(M+Na)^+$: 239.06094, found: 239.06167.

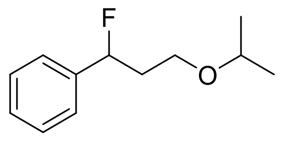

(1-Fluoro-3-isopropoxypropyl)benzene (46). 0.25 mmol scale, 0.3 mL *i*-PrOH, 1 h. After work-up, the crude residue was purified by flash column chromatography on silica gel (petroleum ether : ethyl acetate = 150:1) to give **46** as colorless oil. Yield = 47%, electricity = 2.4 F mol^{-1}. ^{1}H NMR (400 MHz, $CDCl_3$) δ 7.44 – 7.27 (m, 5H), 5.63 (ddd, *J* = 48.0, 8.9, 4.4 Hz, 1H), 3.65 – 3.43 (m, 3H), 2.29 – 1.94 (m, 2H), 1.17 (t, *J* = 6.1 Hz, 6H). ^{13}C NMR (101 MHz, $CDCl_3$) δ 140.3 (d, *J* = 19.5 Hz), 128.6, 128.4 (d, *J* = 2.0 Hz), 125.7 (d, *J* = 6.8 Hz), 91.9 (d, *J* = 169.2 Hz), 71.9, 63.8 (d, *J* = 5.0 Hz), 38.0 (d, *J* = 23.8 Hz), 22.2(4), 22.2(1). ^{19}F NMR (377 MHz, $CDCl_3$) δ -177.25 – -177.51 (m, 1F). HRMS (ESI) m/z calculated for $C_{12}H_{17}FONa$ $(M+Na)^+$: 219.11556, found: 219.11487.

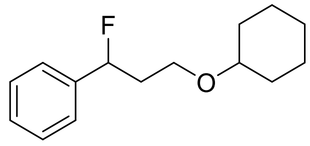

(3-(Cyclohexyloxy)-1-fluoropropyl)benzene (47). 0.25 mmol scale, 0.5 mL cyclohexanol, 1 h. After work-up, the crude residue was purified by flash column chromatography on silica gel (petroleum ether : ethyl acetate = 150:1) to give **47** as colorless oil. Yield = 62%, electricity = 2.4 F mol^{-1}. ^{1}H NMR (400 MHz, $CDCl_3$) δ 7.42 – 7.28 (m, 5H), 5.63 (ddd, *J* = 48.0, 8.8, 4.4 Hz, 1H), 3.69 – 3.45 (m, 2H), 3.25 – 3.19 (m, 1H), 2.31 – 1.97 (m, 2H), 1.94 – 1.88 (m, 2H), 1.75 – 1.73 (m, 2H), 1.55 – 1.52 (m, 1H), 1.36 – 1.07 (m, 5H). ^{13}C NMR (101 MHz, $CDCl_3$) δ 140.4 (d, *J* = 19.5 Hz), 128.6, 128.4 (d, *J* = 2.0 Hz), 125.7 (d, *J* = 6.8 Hz), 92.0 (d, *J* = 169.1 Hz), 77.9, 63.5 (d, *J* = 5.0 Hz), 38.1 (d, *J* = 23.7 Hz), 32.5, 32.3, 26.0, 24.3. ^{19}F NMR (377 MHz, $CDCl_3$) δ -177.20 (ddd, *J* = 46.3, 30.9, 14.2 Hz, 1F). HRMS (ESI) m/z calculated for $C_{15}H_{21}FONa$ $(M+Na)^+$: 259.14686, found: 259.14675.

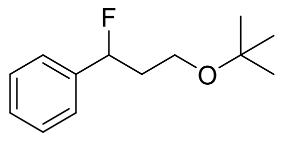

(3-(Tert-butoxy)-1-fluoropropyl)benzene (48). 0.25 mmol scale, 0.45 mL *t*-BuOH, 1 h. After work-up, the crude residue was purified by flash column chromatography on silica gel (petroleum ether: ethyl acetate = 150:1) to give **48** as colorless oil. Yield = 50%, electricity = 2.4 F mol^{-1}. ^{1}H NMR (400 MHz, $CDCl_3$) δ 7.42 – 7.28 (m, 5H), 5.63 (ddd, *J* = 48.1, 9.0, 4.2 Hz, 1H), 3.61 – 3.39 (m, 2H), 2.26 – 1.92 (m, 2H), 1.20 (s, 9H). ^{13}C NMR (101 MHz, $CDCl_3$) δ 140.46 (d, *J* = 19.5 Hz), 128.5, 128.3 (d, *J* = 2.0 Hz), 125.8 (d, *J* = 6.8 Hz), 91.9 (d, *J* = 168.9 Hz), 73.0, 57.3 (d, *J* = 4.8 Hz), 38.3 (d, *J* = 23.6 Hz), 27.7. ^{19}F NMR (377 MHz, $CDCl_3$) δ -177.32 (ddd, *J* = 47.1, 32.1, 14.3 Hz, 1F). HRMS (ESI) m/z calculated for $C_{13}H_{19}FONa$ $(M+Na)^+$: 233.13121, found: 233.13113.

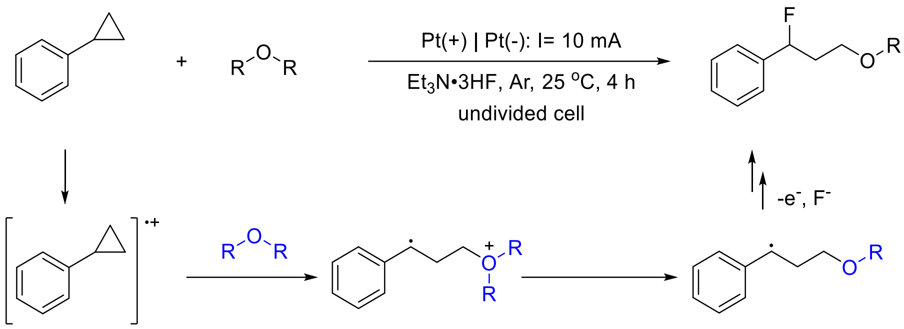

An oven-dried undivided three-necked bottle equipped with a stir bar. The bottle was equipped platinum plate (15 mm×15 mm×0.3 mm) as the anode and platinum plate (15 mm×15 mm×0.3 mm) as the cathode and then charged with argon gas by glove box. Cyclopropylbenzene (0.5 mmol), $Et_3N{\cdot}3HF$ (1.2 mL) and ether (4.8 mL) were added. The reaction mixture was stirred and electrolyzed at a constant current of 10 mA under room temperature for 4 h. The reaction was diluted with water. The organic layer was extracted with CH_2Cl_2, dried with anhydrous Na_2SO_4, filtered, and concentrated under reduced pressure. The pure product was obtained by flash column chromatography on silica gel.

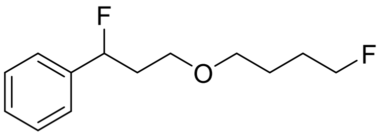

(1-fluoro-3-(4-fluorobutoxy)propyl)benzene (49). 0.5 mmol scale, 4 h. After work-up, the crude residue was purified by flash column chromatography on silica gel (petroleum ether : ethyl acetate = 50:1) to give **49** as colorless oil. Yield = 43%, electricity = 2.9 F mol^{-1}. ^{1}H NMR (400 MHz, $CDCl_3$) δ 7.40 – 7.29 (m, 5H), 5.60 (ddd, *J* = 47.9, 8.8, 4.4 Hz, 1H), 4.46 (dt, *J* = 47.2, 5.9 Hz, 2H), 3.64 – 3.42 (m, 4H), 2.28 – 1.96 (m, 2H), 1.84 – 1.65 (m, 4H). ^{13}C NMR (101 MHz, $CDCl_3$) δ 140.2 (d, *J* = 19.6 Hz), 128.6, 128.4 (d, *J* = 1.9 Hz), 125.6 (d, *J* = 6.7 Hz), 91.8 (d, *J* = 169.6 Hz), 84.0 (d, *J* = 164.4 Hz), 70.5, 66.4 (d, *J* = 4.9 Hz), 37.6 (d, *J* = 23.9 Hz), 27.4 (d, *J* = 19.8 Hz), 25.6 (d, *J* = 5.2 Hz). ^{19}F NMR (377 MHz, $CDCl_3$) δ -177.31 (ddd, *J* = 46.6, 31.0, 14.6 Hz, 1F), -217.88 – -218.33 (m, 1F). HRMS (ESI) m/z calculated for $C_{13}H_{18}F_2ONa$ $(M+Na)^+$: 251.12179, found: 251.12178.

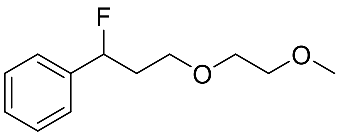

(1-fluoro-3-(2-methoxyethoxy)propyl)benzene (**50**). 0.5 mmol scale, 4 h. After work-up, the crude residue was purified by flash column chromatography on silica gel (petroleum ether : ethyl acetate = 10:1) to give **50** as colorless oil**.** Yield = 44%, electricity = 2.9 F mol^{-1}. ^{1}H NMR (400 MHz, $CDCl_3$) δ 7.41 – 7.30 (m, 5H), 5.64 (ddd, *J* = 47.9, 8.9, 4.3 Hz, 1H), 3.71 – 3.50 (m, 6H), 3.40 (s, 3H), 2.31 – 2.00 (m, 2H). ^{13}C NMR (101 MHz, $CDCl_3$) δ 140.2 (d, *J* = 19.5 Hz), 128.6, 128.4 (d, *J* = 1.9 Hz), 125.7 (d, *J* = 6.7 Hz), 91.7 (d, *J* = 169.5 Hz), 72.0, 70.4, 67.1 (d, *J* = 4.9 Hz), 59.2, 37.4 (d, *J* = 23.8 Hz). ^{19}F NMR (377 MHz, $CDCl_3$) δ -177.50 (ddd, *J* = 46.5, 31.2, 14.7 Hz, 1F). HRMS (ESI) m/z calculated for $C_{12}H_{17}FO_2Na$ $(M+Na)^+$: 235.11048, found: 235.11037.

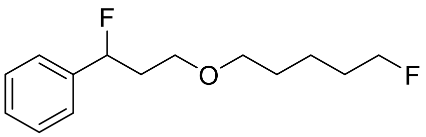

(1-Fluoro-3-((5-fluoropentyl)oxy)propyl)benzene (**51**). 0.5 mmol scale, add 1 mL DCE, 4 h. After work-up, the crude residue was purified by flash column chromatography on silica gel (petroleum ether: ethyl acetate = 50:1) to give **51** as colorless oil. Yield = 36%, electricity = 2.9 F mol^{-1}. ^{1}H NMR (400 MHz, $CDCl_3$) δ 7.42 – 7.26 (m, 5H), 5.61 (ddd, *J* = 47.9, 8.8, 4.4 Hz, 1H), 4.44 (dt, *J* = 47.3, 6.1 Hz, 2H), 3.63 – 3.41 (m, 4H), 2.28 – 1.96 (m, 2H), 1.79 – 1.57 (m, 4H), 1.53 – 1.43 (m, 2H). ^{13}C NMR (101 MHz, $CDCl_3$) δ 140.2 (d, *J* = 19.5 Hz), 128.5, 128.4 (d, *J* = 2.0 Hz), 125.6 (d, *J* = 6.7 Hz), 91.8 (d, *J* = 169.5 Hz), 84.1 (d, *J* = 164.3 Hz), 70.9, 66.4 (d, *J* = 5.0 Hz), 37.6 (d, *J* = 23.9 Hz), 30.3 (d, *J* = 19.5 Hz), 29.4, 22.0 (d, *J* = 5.5 Hz). ^{19}F NMR (377 MHz, $CDCl_3$) δ -177.25 (ddd, *J* = 46.5, 30.8, 14.6 Hz, 1F), -218.24 (tt, *J* = 47.1, 25.1 Hz, 1F). HRMS (ESI) m/z calculated for $C_{14}H_{20}F_2ONa$ $(M+Na)^+$: 265.13744, found: 265.13684.

6) Proposed mechanism of 1,3-oxyfluorination reaction using ether as nucleophiles

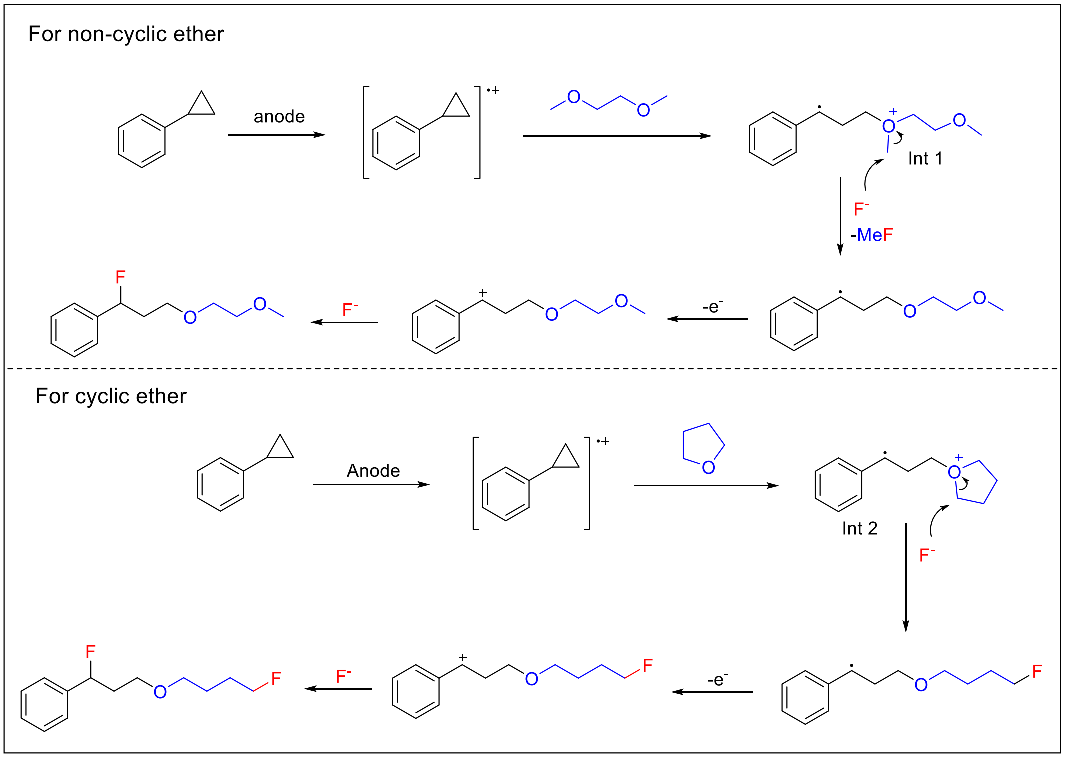

Supplementary Figure 5 Proposed mechanism of 1,3-oxyfluorination reaction

7) Procedures and analytical data of 1,3-dioxygenation compounds

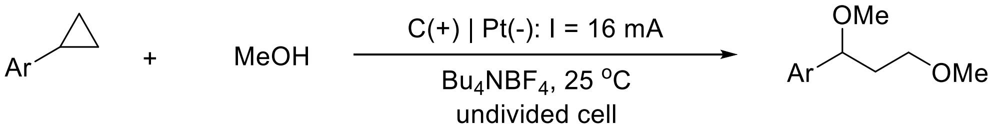

An oven-dried undivided three-necked bottle equipped with a stir bar. The bottle was equipped carbon cloth (15 mm×15 mm) as the anode and platinum plate (15 mm×15 mm×0.3 mm) as the cathode and then charged with argon gas by glove box. Arylcyclopropane (0.25 mmol), Bu_4NBF_4 (0.25 mmol) and MeOH (6 mL) were added. The reaction mixture was stirred and electrolyzed at a constant current of 16 mA for 1 h under room temperature. The reaction mixture was concentrated under reduced pressure. The pure product was obtained by flash column chromatography on silica gel.

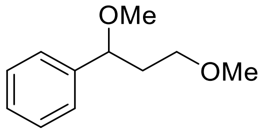

(1,3-Dimethoxypropyl)benzene (**5**). GC yield = 95%, Electricity = 2.4 F mol^{-1}. ^{1}H NMR (400 MHz, Chloroform-*d*) δ 7.39 – 7.19 (m, 5H), 4.28 (dd, *J* = 8.1, 5.7 Hz, 1H), 3.53 – 3.42 (m, 1H), 3.37 – 3.26 (m, 4H), 3.20 (s, 3H), 2.11 – 1.80 (m, 2H). ^{13}C NMR (101 MHz, Chloroform-*d*) δ 142.1, 128.5, 127.6, 126.7, 80.7, 69.2, 58.6, 56.7, 38.2. HRMS (ESI) m/z calculated for $C_{11}H_{17}O_2$ $(M+H)^+$: 181.12231, found: 181.12160.

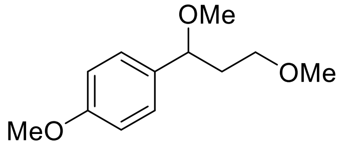

1-(1,3-Dimethoxypropyl)-4-methoxybenzene (52). The crude residue was purified by flash column chromatography on silica gel (petroleum ether: ethyl acetate = 20:1) to give **52** as colorless oil. Isolated yield = 74%, Electricity = 2.4 F mol^{-1}. ^{1}H NMR (400 MHz, $CDCl_3$) δ 7.23 – 7.17 (m, 2H), 6.91 – 6.84 (m, 2H), 4.21 (dd, *J* = 7.9, 6.0 Hz, 1H), 3.79 (s, 3H), 3.47 – 3.42 (m, 1H), 3.33 – 3.24 (m, 4H), 3.17 (s, 3H), 2.14 – 1.74 (m, 2H). ^{13}C NMR (101 MHz, $CDCl_3$) δ 159.2, 134.0, 128.0, 113.9, 80.3, 69.4, 58.7, 56.5, 55.4, 38.2. HRMS (ESI) m/z calculated for $C_{12}H_{19}O_3$ $(M+H)^+$: 211.13287, found: 211.13254.

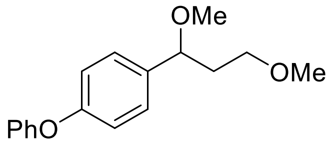

1-(1,3-Dimethoxypropyl)-4-phenoxybenzene (53). The crude residue was purified by flash column chromatography on silica gel (petroleum ether: ethyl acetate = 20:1) to give **53** as colorless oil. Isolated yield = 93%, reaction time 50 min, Electricity = 2 F mol^{-1}. ^{1}H NMR (400 MHz, $CDCl_3$) δ 7.37 – 7.30 (m, 2H), 7.28 – 7.21 (m, 2H), 7.13 – 7.06 (m, 1H), 7.04 – 6.95 (m, 4H), 4.26 (dd, *J* = 8.0, 5.8 Hz, 1H), 3.52 – 3.45 (m, 1H), 3.38 – 3.27 (m, 4H), 3.21 (s, 3H), 2.14 – 1.78 (m, 2H). ^{13}C NMR (101 MHz, $CDCl_3$) δ 157.2, 156.8, 136.8, 129.8, 128.1, 123.4, 119.1, 118.8, 80.3, 69.3, 58.7, 56.7, 38.2. HRMS (ESI) m/z calculated for $C_{17}H_{20}O_3Na$ $(M+Na)^+$: 295.13047, found: 295.13041.

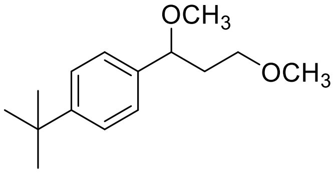

1-(Tert-butyl)-4-(1,3-dimethoxypropyl)benzene (54). The crude residue was purified by flash column chromatography on silica gel (petroleum ether: ethyl acetate = 30:1) to give **54** as colorless oil. Isolated yield = 85%, Electricity = 2.4 F mol^{-1}. ^{1}H NMR (400 MHz, $CDCl_3$) δ 7.35 (d, *J* = 8.4 Hz, 2H), 7.21 (d, *J* = 8.3 Hz, 2H), 4.25 (dd, *J* = 8.1, 5.6 Hz, 1H), 3.56 – 3.46 (m, 1H), 3.36 – 3.28 (m, 4H), 3.20 (s, 3H), 2.14 – 1.70 (m, 2H), 1.31 (s, 9H). ^{13}C NMR (101 MHz, $CDCl_3$) δ 150.5, 139.0, 126.4, 125.4, 80.5, 69.4, 58.7, 56.8, 38.2, 34.6, 31.5. HRMS (ESI) m/z calculated for $C_{15}H_{24}O_2Na$ $(M+Na)^+$: 259.16685, found: 259.16656.

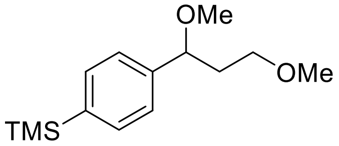

(4-(1,3-Dimethoxypropyl)phenyl)trimethylsilane (55). The crude residue was purified by flash column chromatography on silica gel (petroleum ether: ethyl acetate = 30:1) to give **55** as colorless oil. Isolated yield = 85%, Electricity = 2.4 F mol^{-1}. ^{1}H NMR (400 MHz, $CDCl_3$) δ 7.53 – 7.46 (m, 2H), 7.31 – 7.25 (m, 2H), 4.27 (dd, *J* = 8.2, 5.5 Hz, 1H), 3.55 – 3.45 (m, 1H), 3.40 – 3.28 (m, 4H), 3.21 (s, 3H), 2.12 – 1.79 (m, 2H), 0.26 (s, 9H). ^{13}C NMR (101 MHz, $CDCl_3$) δ 142.7, 139.7, 133.6, 126.1, 80.8, 69.3, 58.7, 56.9, 38.3, -1.0. HRMS (ESI) m/z calculated for $C_{14}H_{24}O_2SiNa$ $(M+Na)^+$: 275.14378, found: 275.14359.

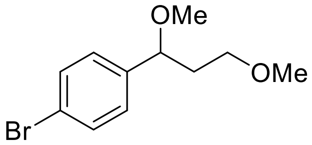

1-Bromo-4-(1,3-dimethoxypropyl)benzene (56). The crude residue was purified by flash column chromatography on silica gel (petroleum ether: ethyl acetate = 30:1) to give **56** as colorless oil. Isolated yield = 95%, Electricity = 2.4 F mol^{-1}. ^{1}H NMR (400 MHz, $CDCl_3$) δ 7.46 (dd, *J* = 8.1, 1.5 Hz, 2H), 7.16 (dd, *J* = 8.2, 1.5 Hz, 2H), 4.24 (dd, *J* = 7.8, 6.1 Hz, 1H), 3.50 – 3.41 (m, 1H), 3.38 – 3.21 (m, 4H), 3.18 (s, 3H), 2.08 – 1.72 (m, 2H). ^{13}C NMR (101 MHz, $CDCl_3$) δ 141.2, 131.7, 128.5, 121.4, 80.2, 69.0, 58.7, 56.8, 38.2. HRMS (ESI) m/z calculated for $C_{11}H_{26}BrO_2$ $(M+H)^+$: 259.03282, found: 259.03278.

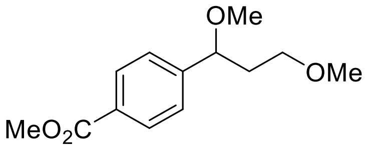

Methyl 4-(1,3-dimethoxypropyl)benzoate (**57**). The crude residue was purified by flash column chromatography on silica gel (petroleum ether: ethyl acetate = 20:1) to give **57** as colorless oil. Isolated yield = 85%, Electricity = 2.4 F mol^{-1}. ^{1}H NMR (400 MHz, $CDCl_3$) δ 7.95 (d, *J* = 8.3 Hz, 2H), 7.30 (d, *J* = 8.3 Hz, 2H), 4.28 (dd, *J* = 8.1, 5.6 Hz, 1H), 3.84 (s, 3H), 3.45 – 3.39 (m, 1H), 3.26 – 3.17 (m, 4H), 3.15 (s, 3H), 2.11 – 1.67 (m, 2H). ^{13}C NMR (101 MHz, $CDCl_3$) δ 167.1, 147.610, 129.9, 129.6, 126.7, 80.4, 68.9, 58.7, 57.0, 52.2, 38.2. HRMS (ESI) m/z calculated for $C_{13}H_{18}O_4Na$ $(M+Na)^+$: 261.10973, found: 261.10965.

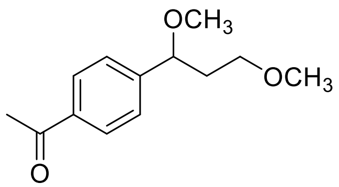

1-(4-(1,3-Dimethoxypropyl)phenyl)ethan-1-one (58). The crude residue was purified by flash column chromatography on silica gel (petroleum ether: ethyl acetate = 20:1) to give **58** as colorless oil. Isolated yield = 84%, Electricity = 2.4 F mol^{-1}. ^{1}H NMR (400 MHz, $CDCl_3$) δ 7.93 – 7.86 (m, 2H), 7.39 – 7.36 (m, 2H), 4.33 (dd, *J* = 8.2, 5.5 Hz, 1H), 3.50 – 3.44 (m, 1H), 3.34 – 3.23 (m, 4H), 3.19 (s, 3H), 2.57 (s, 3H), 2.08 – 1.94 (m, 1H), 1.84 – 1.76 (m, 1H). ^{13}C NMR (101 MHz, $CDCl_3$) δ 197.9, 147.9, 136.7, 128.7, 126.8, 80.3, 68.9, 58.7, 57.1, 38.1, 26.7. HRMS (ESI) m/z calculated for $C_{13}H_{19}O_3$ $(M+H)^+$: 223.13287, found: 223.13293.

8) Graphical guide and general procedure for gram scale reaction

Graphical guide for the gram set-up: As experiment set-up, a carbon cloth anode (20 mm×23 mm), a nickel foam cathode (20 mm×23 mm), rubber plugs, an undivided three-necked bottle and a dual display potentiostat (HJS-292B) (made in China) were used.

a) Carbon cloth anode and nickel foam cathode b) Assembly of electrochemical cell

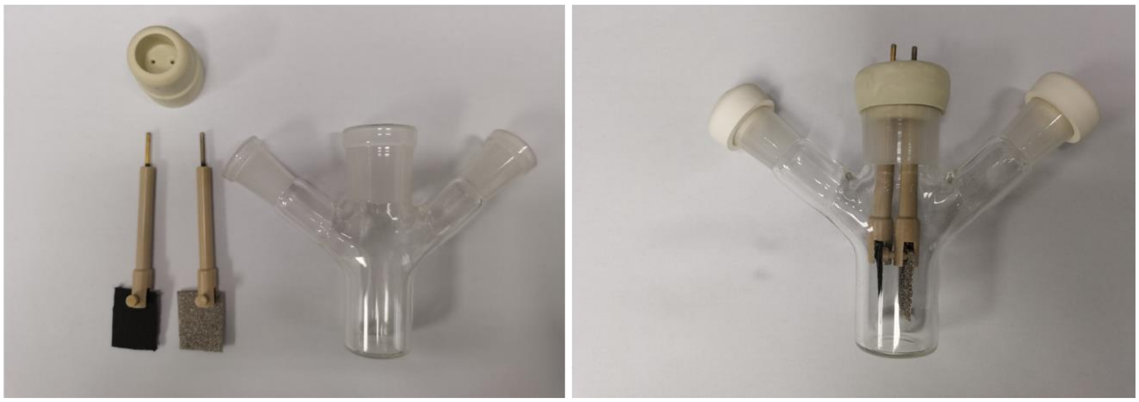

c) Compared with milligram scale set-up　　　　d) Current control electrolysis

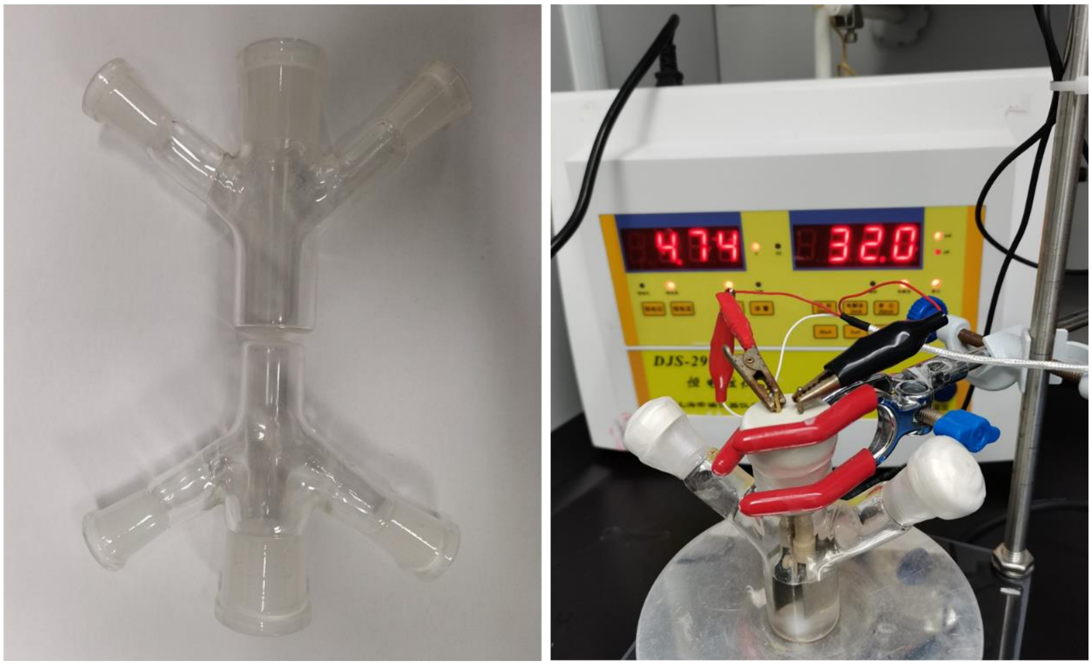

An oven-dried undivided three-necked bottle equipped with a stir bar. The bottle was equipped carbon cloth (20 mm×25mm) as the anode and nickel foam (20 mm×25 mm) as the cathode and then charged with argon gas by glove box. Arylcyclopropane (6 or 8 mmol), $Et_3N·3HF$ (14.5 mmol, 2.4 mL) and $PhCF_3$ (9.6 mL) were added. The reaction mixture was stirred and electrolyzed at a constant current of 32 mA under room temperature. Then, the reaction was diluted with water. The organic layer was extracted with CH_2Cl_2, dried with anhydrous Na_2SO_4, filtered, and concentrated under reduced pressure. The pure product was obtained by flash column chromatography on silica gel.

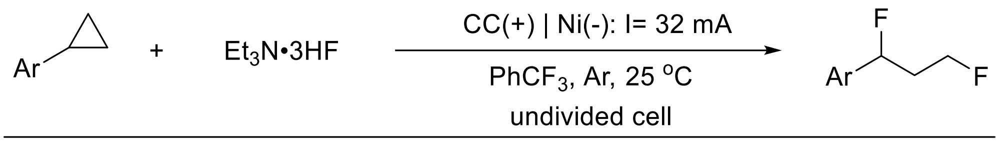

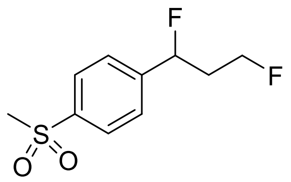

6, 6 mmol,1,26 g, 90%

Reaction time : 32 h

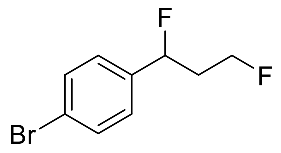

10, 8 mmol, 1.18 g, 63%

Reaction time : 25 h

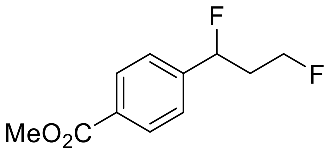

14, 8 mmol, 1.11g, 65%

Reaction time : 75 h

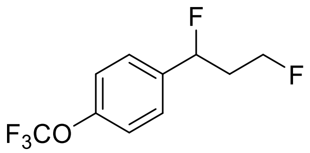

22, 8 mmol, 1.21g, 63%

Reaction time : 25 h

9) Mechanistic study

Cyclic voltammetry studies:

Cyclic voltammetry was performed in a three-electrode cell. The working electrode was a Pt electrode, the counter electrode was a graphite rod. The reference was an Ag/AgCl electrode submerged in saturated aqueous KCl solution. CH_3CN (10 mL) containing 0.5 mmol $^{n}Bu_4NBF_4$ was tested as blank background. The cyclic voltammogram of cyclopropanes (10 mM) were recorded at room temperature in an electrolyte of $^{n}Bu_4NBF_4$ (50 mM) in MeCN. The scan rate is 100 mV/s.

Trap of benzyl carbonium intermediate:

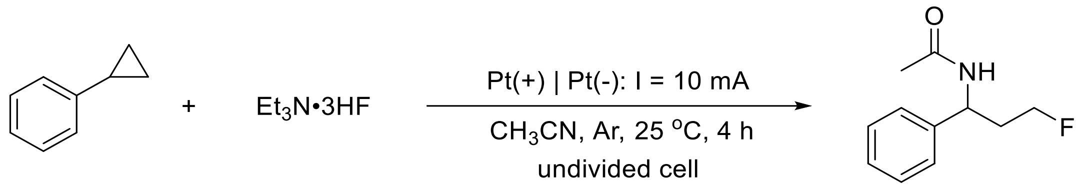

An oven-dried undivided three-necked bottle equipped with a stir bar. The bottle was equipped platinum plate (15 mm×15 mm×0.3 mm) as the anode and platinum plate (15 mm×15 mm×0.3 mm) as the cathode and then charged with argon gas by glove box. Phenylcyclopropane (0.5 mmol), $Et_3N·3HF$ (1.2 mL) and CH_3CN (4.8 mL) were added. The reaction mixture was stirred and electrolyzed at a constant current of 10 mA under room temperature for 4 h. The reaction was diluted with water. The organic layer was extracted with CH_2Cl_2, dried with anhydrous Na_2SO_4, filtered, and concentrated under reduced pressure. The pure product was obtained by flash column chromatography on silica gel.

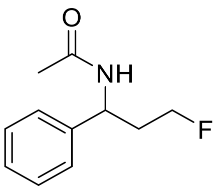

N-(3-fluoro-1-phenylpropyl)acetamide (59). After work-up, the crude residue was purified by flash column chromatography on silica gel (petroleum ether: ethyl acetate: Et_3N = 50:100:0.15) to give product as yellow solid**.** Yield = 43%, electricity = 2.9 F mol^{-1}. ^1H NMR (400 MHz, $CDCl_3$) δ 7.34 – 7.22 (m, 5H), 7.00 (d, *J* = 8.0 Hz, 1H), 5.14 (td, *J* = 7.6, 7.2 Hz, 1H), 4.54 – 4.26 (m, 2H), 2.19 – 2.05 (m, 2H), 1.92 (s, 3H). ^{13}C NMR (101 MHz, $CDCl_3$) δ 169.8, 141.5, 128.7, 127.5, 126.5, 81.4 (d, *J* = 164.3 Hz), 50.4 (d, *J* = 4.5 Hz), 36.6 (d, *J* = 19.4 Hz), 23.2. ^{19}F NMR (377 MHz, $CDCl_3$) δ -219.44 (tt, *J* = 47.4, 25.7 Hz, 1F). HRMS (ESI) m/z calculated for $C_{11}H_{15}FNO$ $(M+H)^+$: 196.11322, found: 196.11252.

10) General Computational Calculation Details

DFT calculations were performed using the M06-2x method[12] with the Gaussian09 program[13]. The 6-311G(d,p) basis set was used for all the elements and $PhCF_3$ was employed as the solvent during the geometry optimization by using SMD model[14]. For the integration grid in the calculations, the parameter int = ultrafine was used. Frequency calculations at the same level of theory have been performed to identify all of the stationary points as minima (zero imaginary frequencies). Grimme's dispersion correction[15] was used during the calculations.

The electrostatic potential surface was generated using Multiwfn[16] and VMD[17].

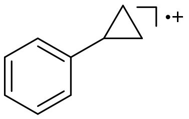

Thermal correction to Gibbs Free Energy= 0.129230

Sum of electronic and thermal Free Energies= -348.535613

C	1.61947800	1.37490300	0.00005000
C	0.28484300	1.07432600	0.00050600
C	-0.14375600	-0.29413800	0.00052100
C	0.84908300	-1.32790600	0.00031600
C	2.18095900	-1.01290300	-0.00017700

C	2.57627800	0.33858700	-0.00038100
H	1.94814200	2.40616400	0.00000100
H	-0.44652700	1.87386500	0.00085100
H	0.51957000	-2.36003500	0.00049100
H	2.93191100	-1.79277600	-0.00040500
H	3.63070700	0.58715800	-0.00084800
C	-1.51210600	-0.67278800	0.00039600
C	-2.58575300	0.22935600	0.72172500
C	-2.58499700	0.22902900	-0.72264200
H	-1.73075600	-1.73159200	0.00047600
H	-2.17595700	1.07541000	1.25681100
H	-3.30404000	-0.38161800	1.25365300
H	-3.30264600	-0.38216900	-1.25518300
H	-2.17456500	1.07478500	-1.25773400

NMR Spectra of Products

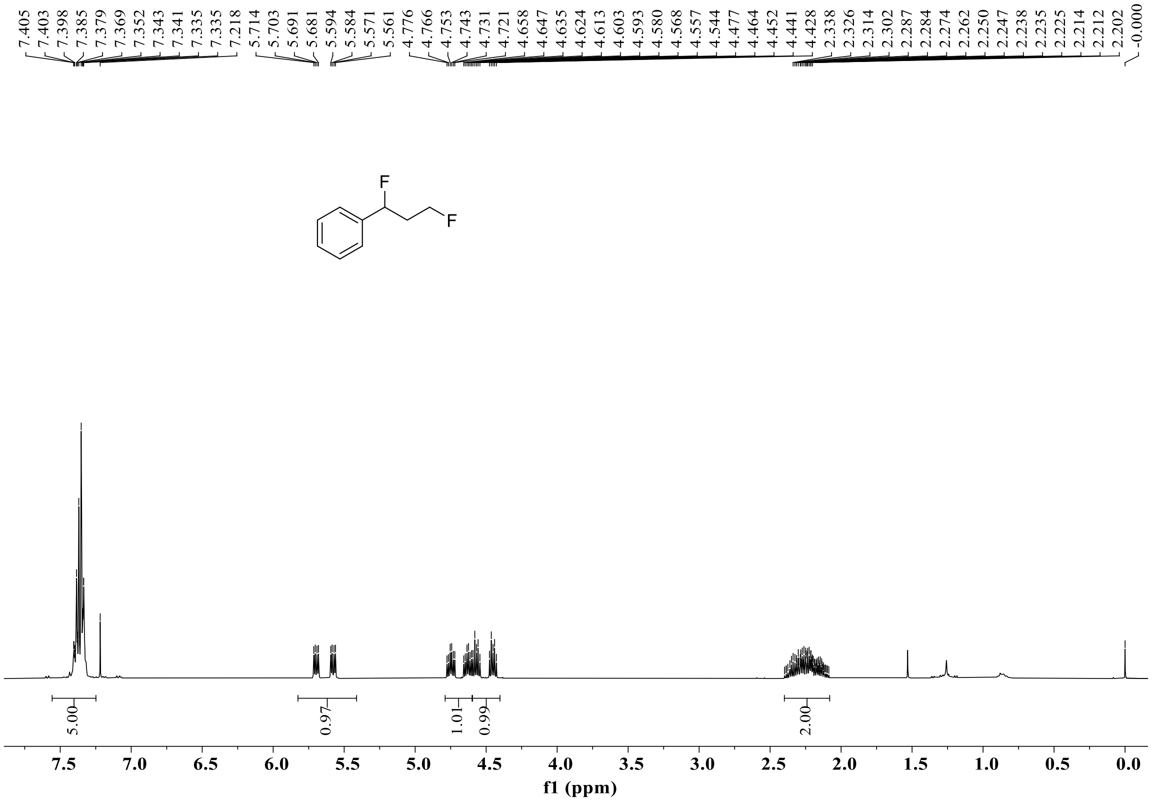

Supplementary Figure 6 ^{1}H NMR spectra of compound **2**

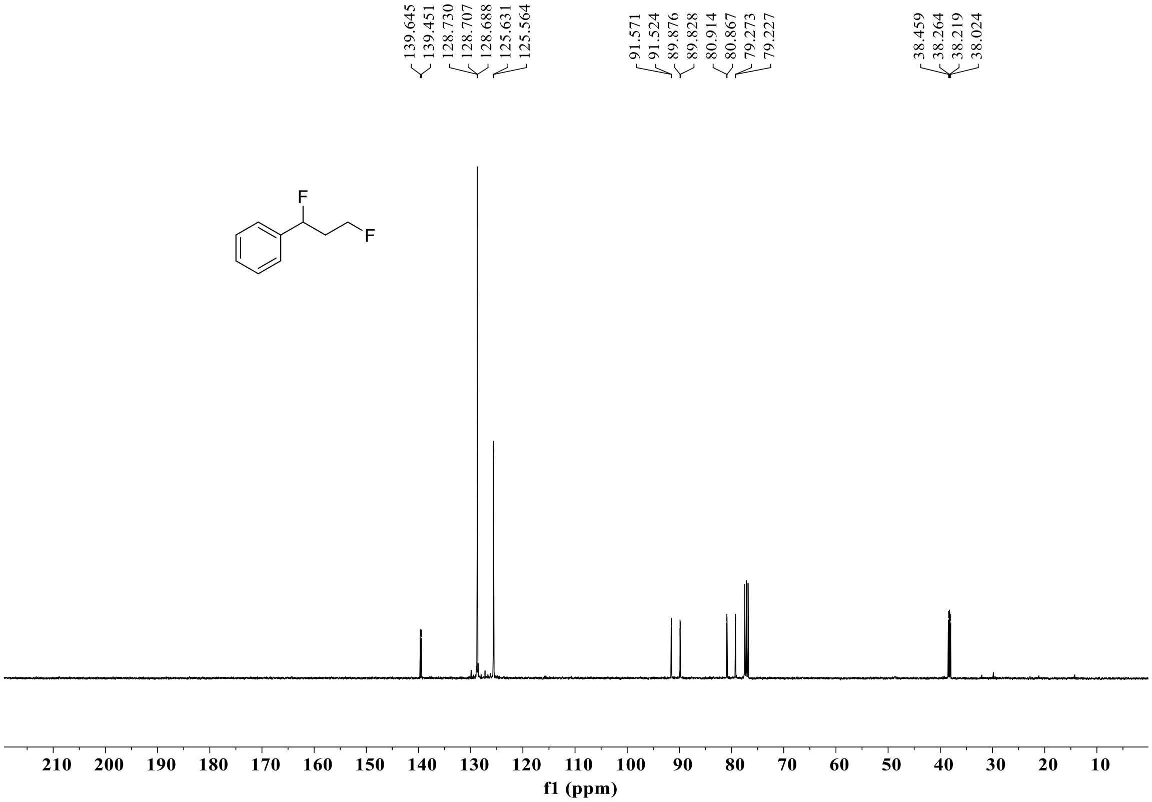

Supplementary Figure 7 ^{13}C NMR spectra of compound **2**

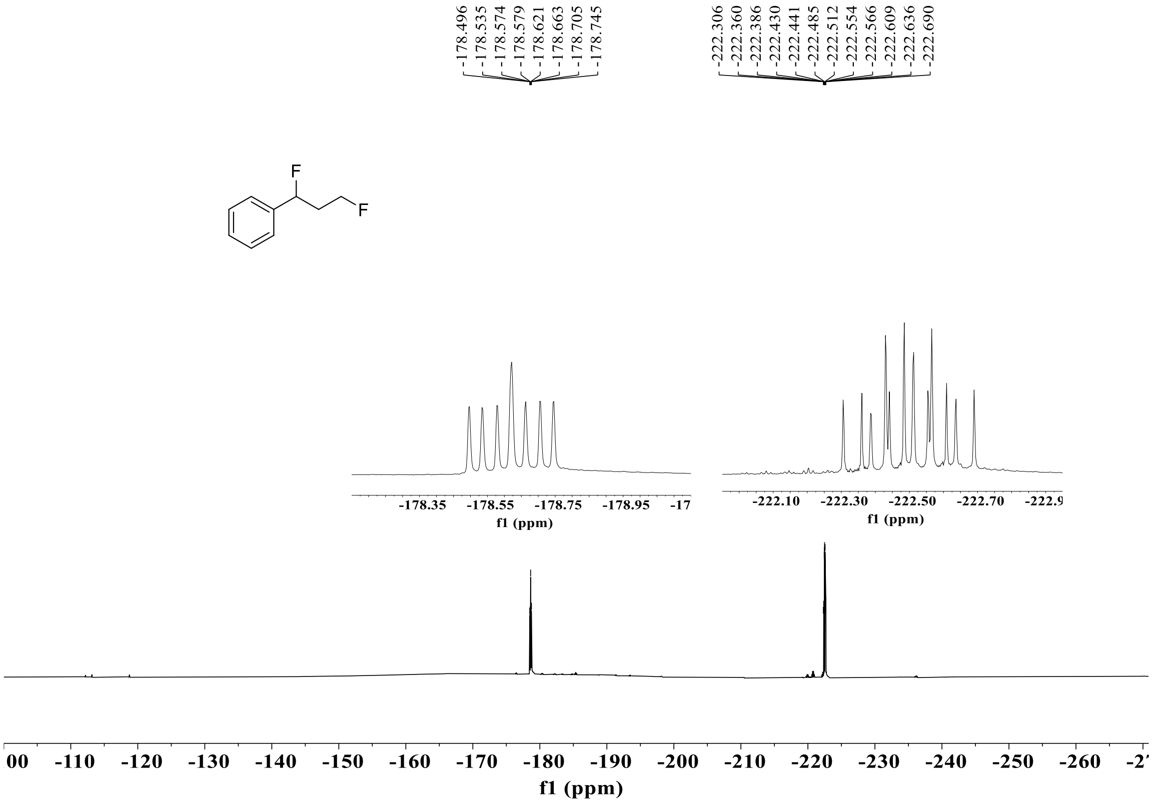

Supplementary Figure 8 ^{19}F NMR spectra of compound **2**

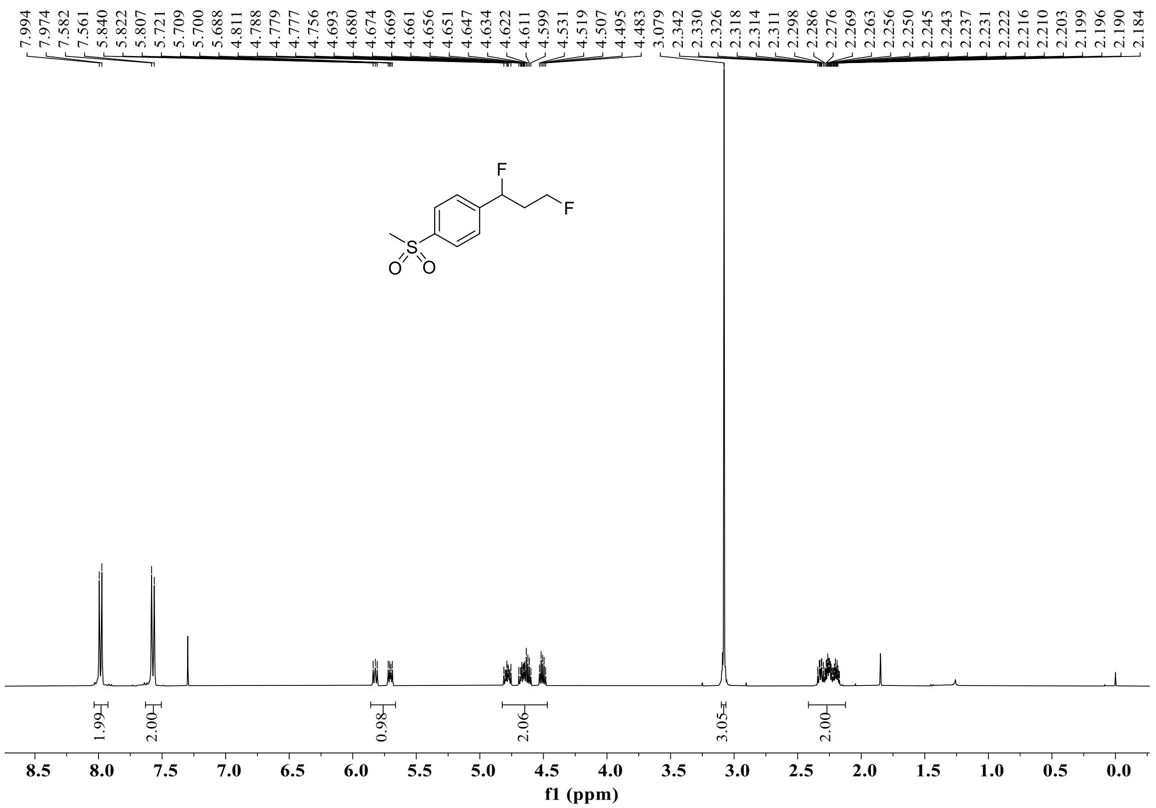

Supplementary Figure 9 ^{1}H NMR spectra of compound **6**

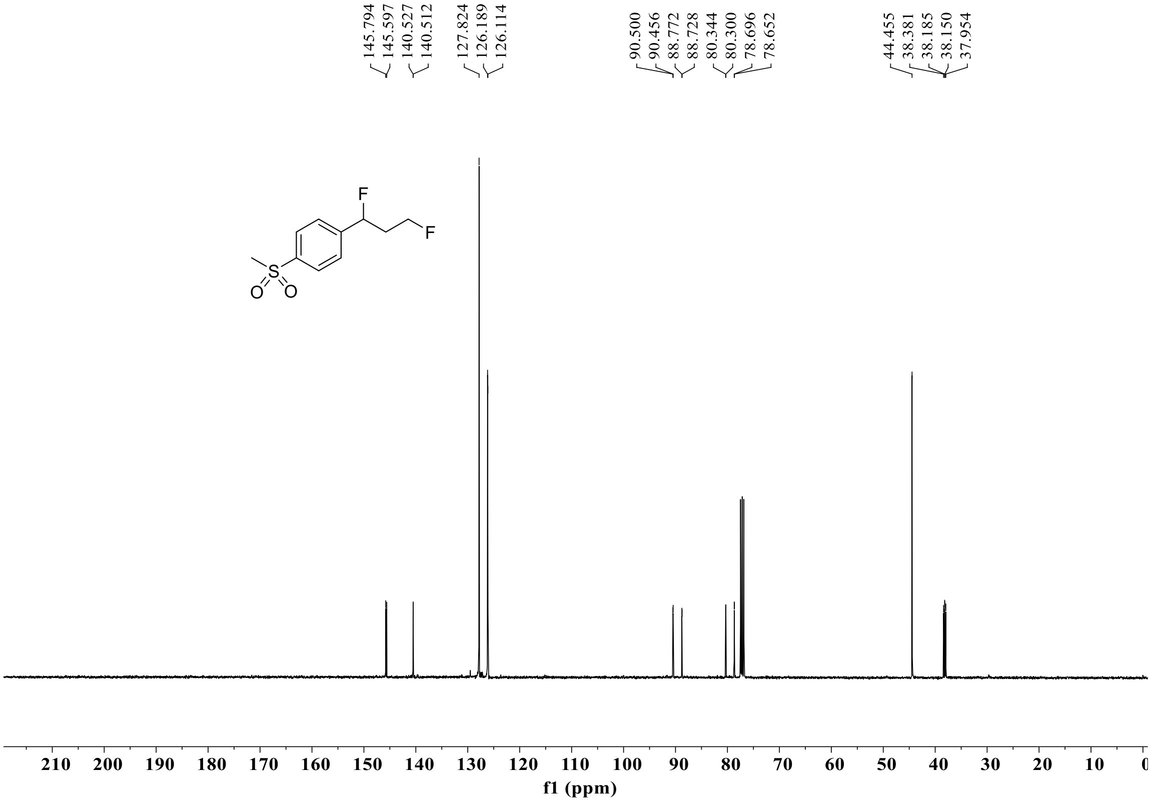

Supplementary Figure 10 ^{13}C NMR spectra of compound **6**

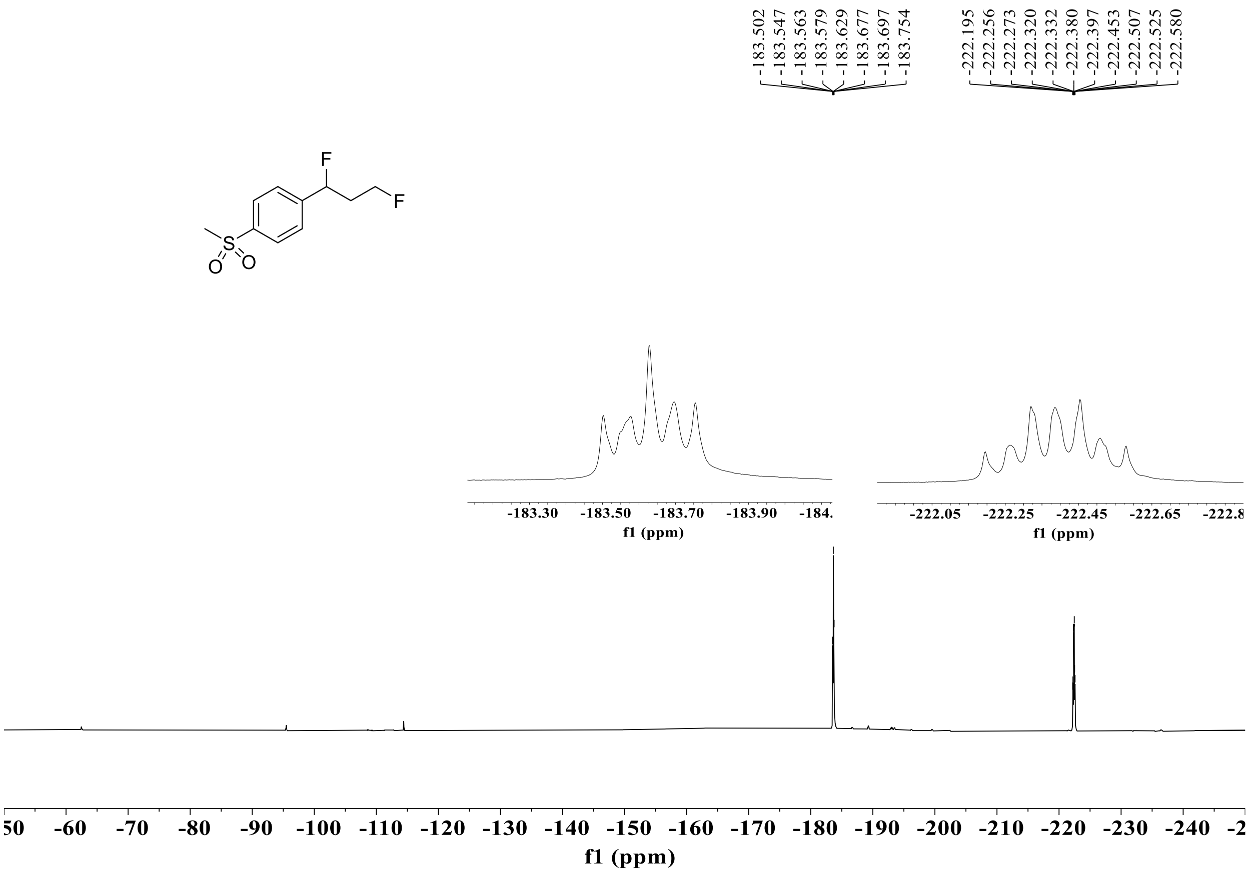

Supplementary Figure 11 ^{19}F NMR spectra of compound **6**

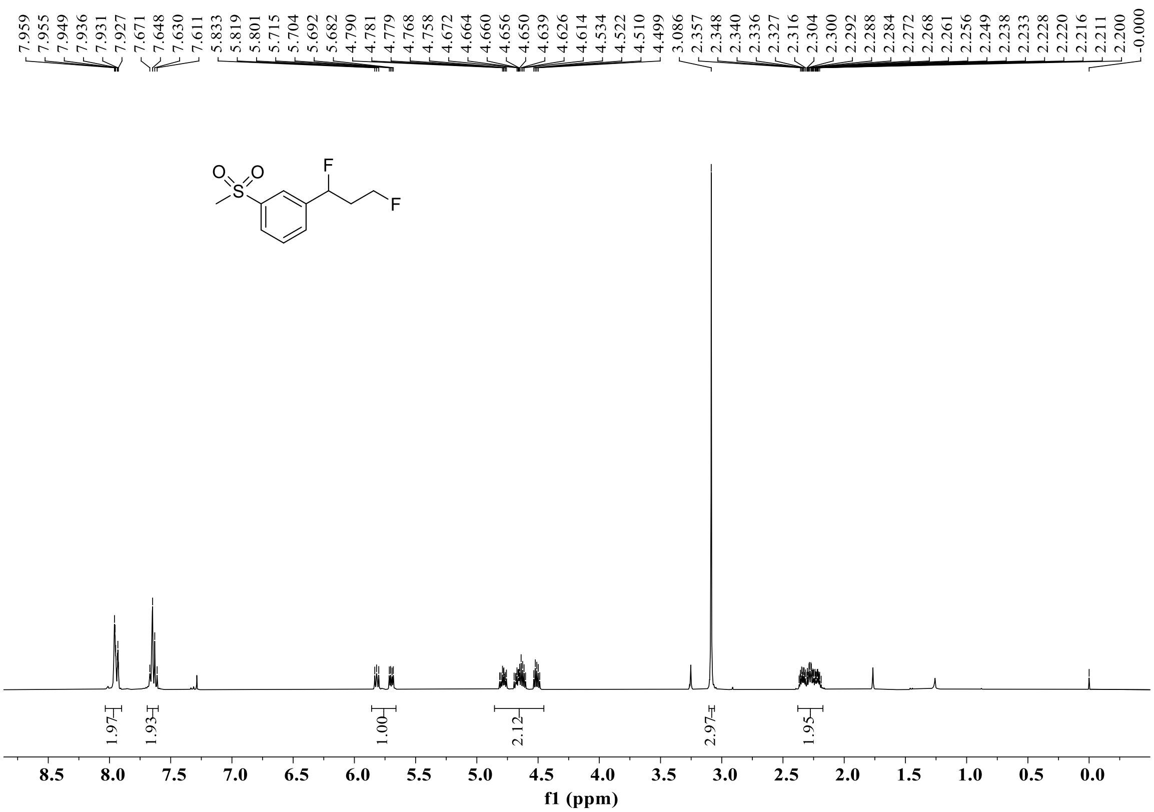

Supplementary Figure 12 ^{1}H NMR spectra of compound **7**

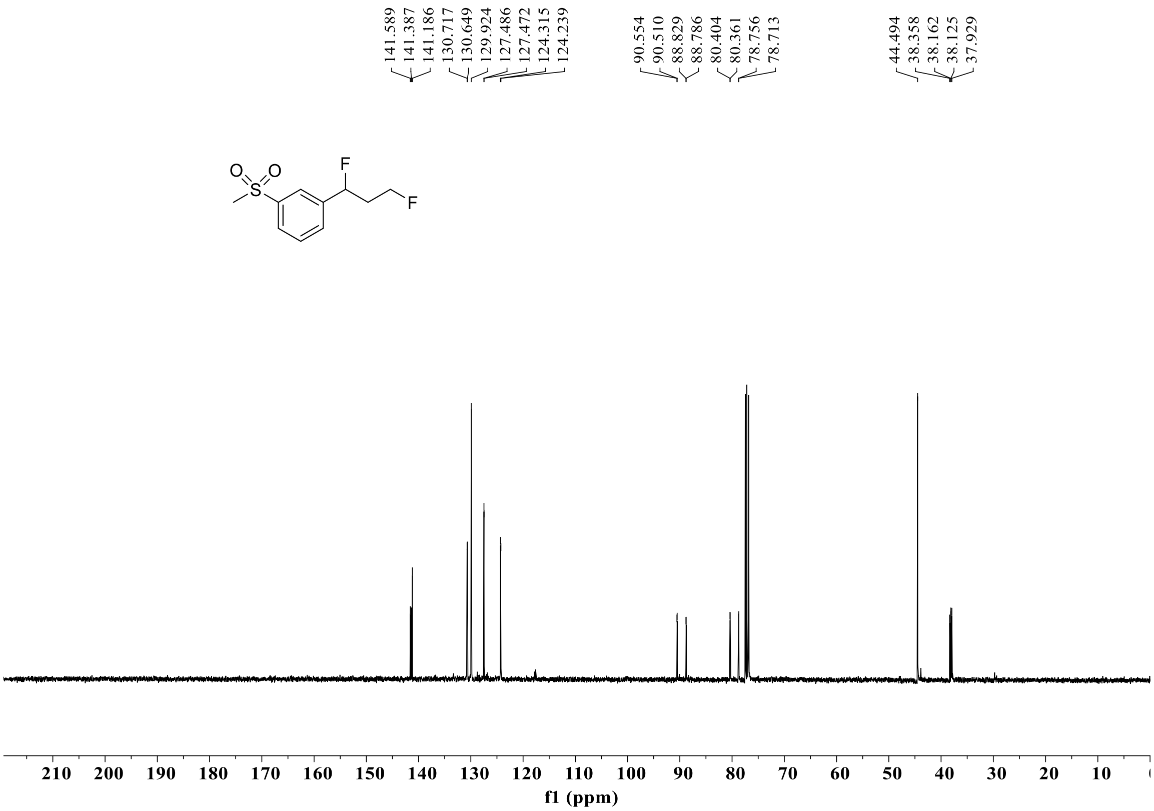

Supplementary Figure 13 ^{13}C NMR spectra of compound **7**

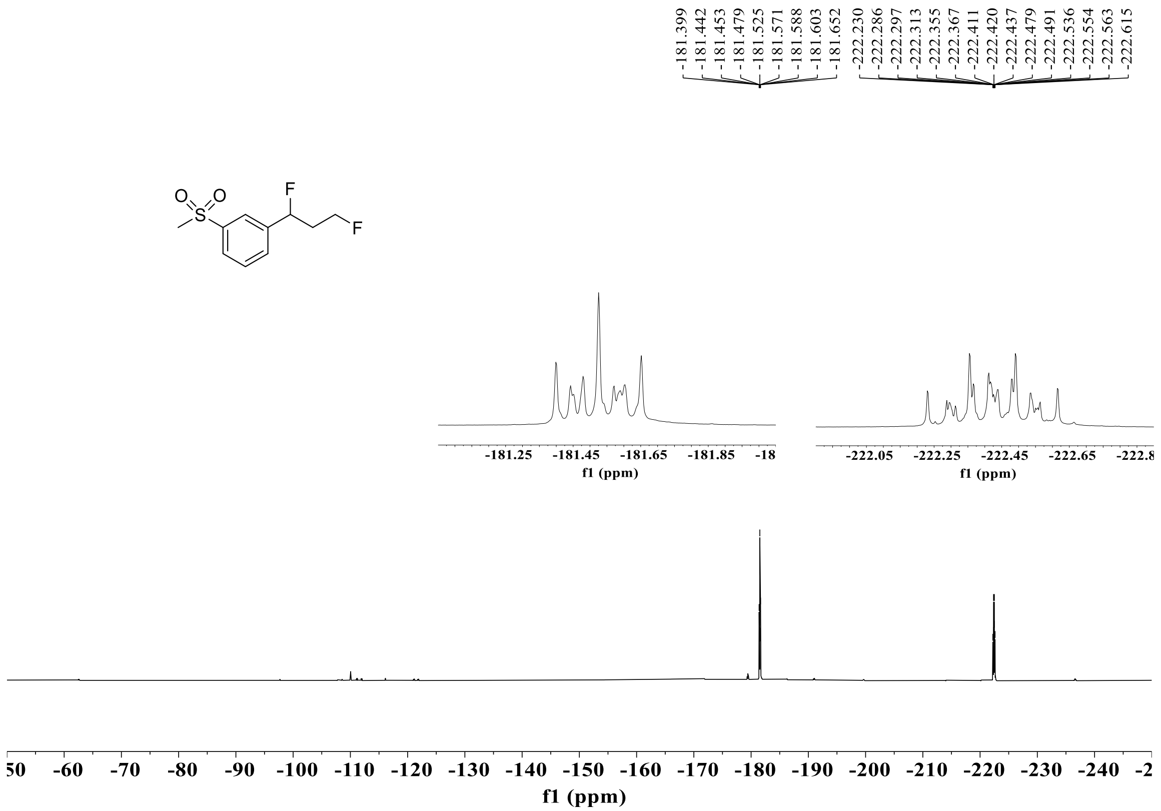

Supplementary Figure 14 ^{19}F NMR spectra of compound **7**

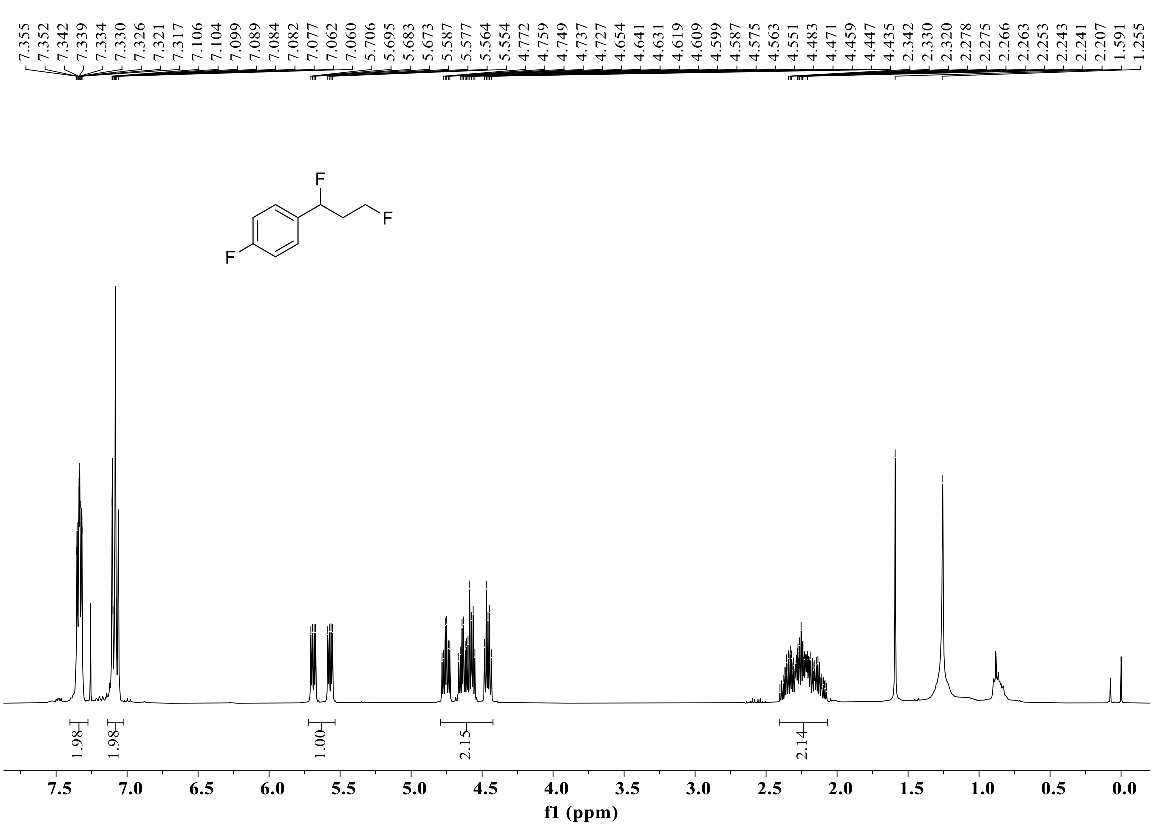

Supplementary Figure 15 ^{1}H NMR spectra of compound **8**

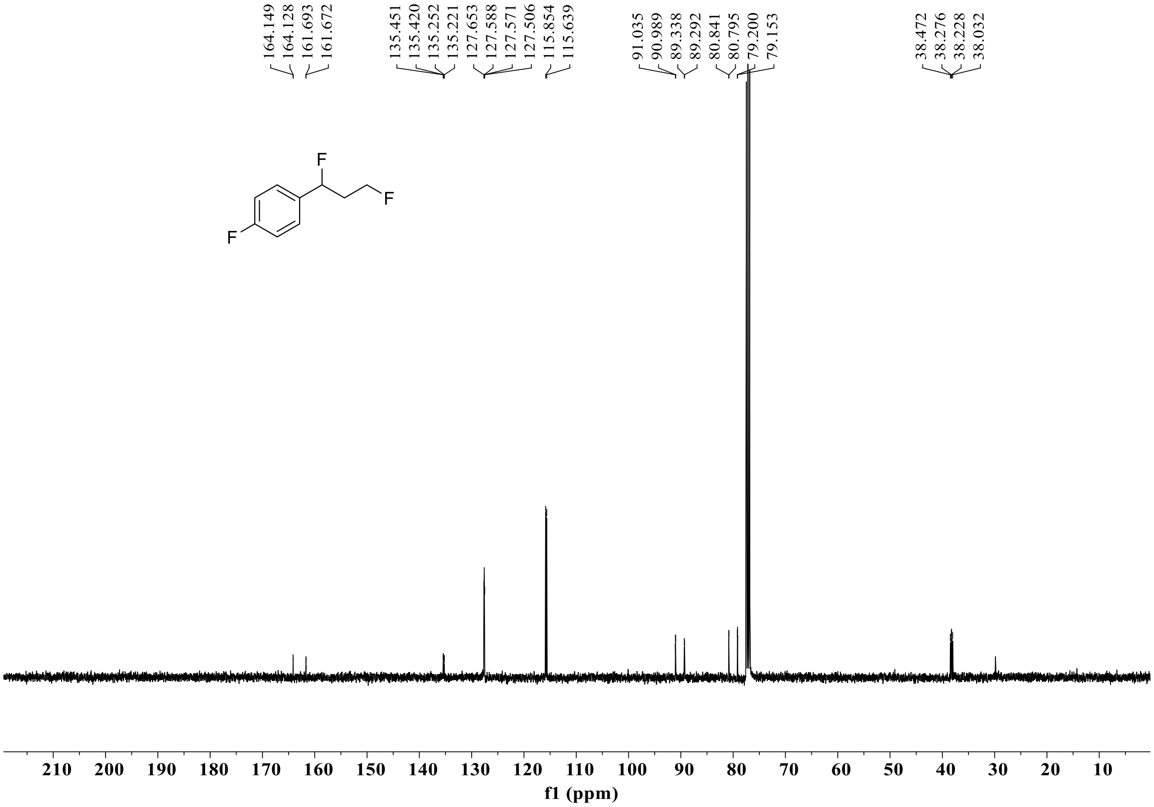

Supplementary Figure 16 ^{13}C NMR spectra of compound **8**

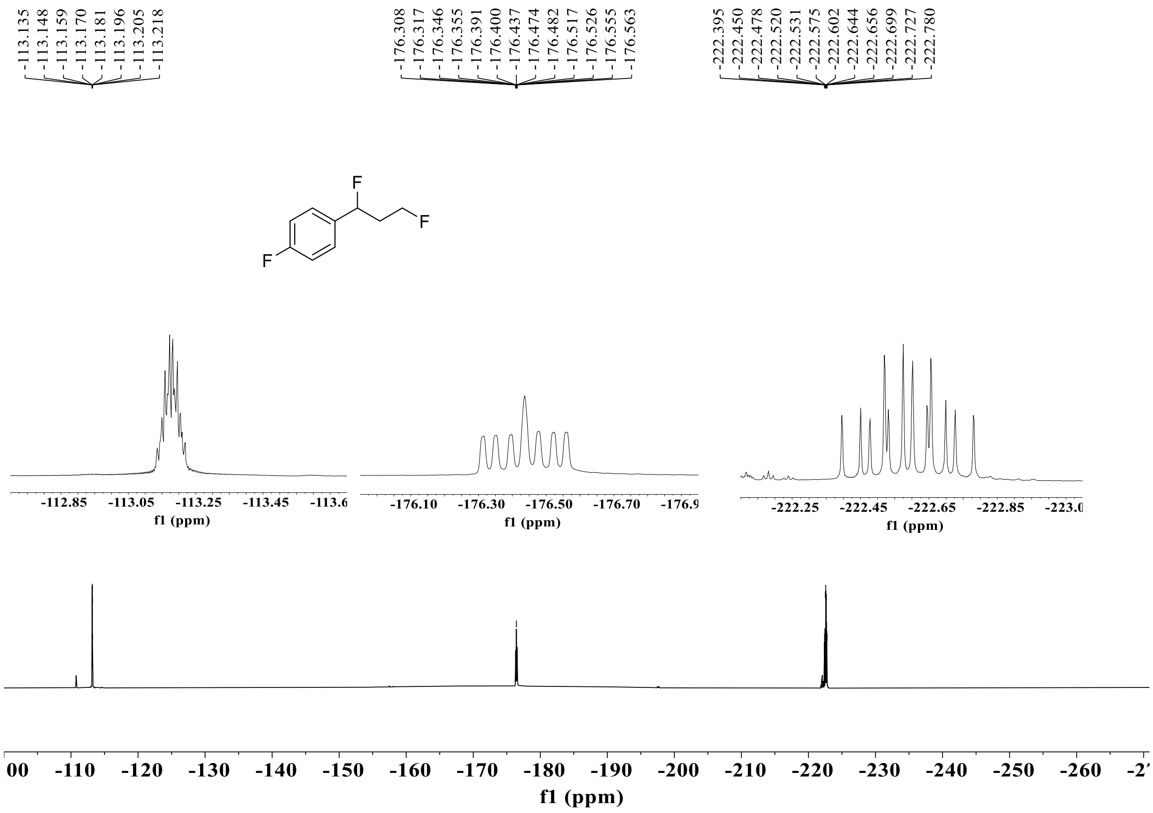

Supplementary Figure 17 ^{19}F NMR spectra of compound **8**

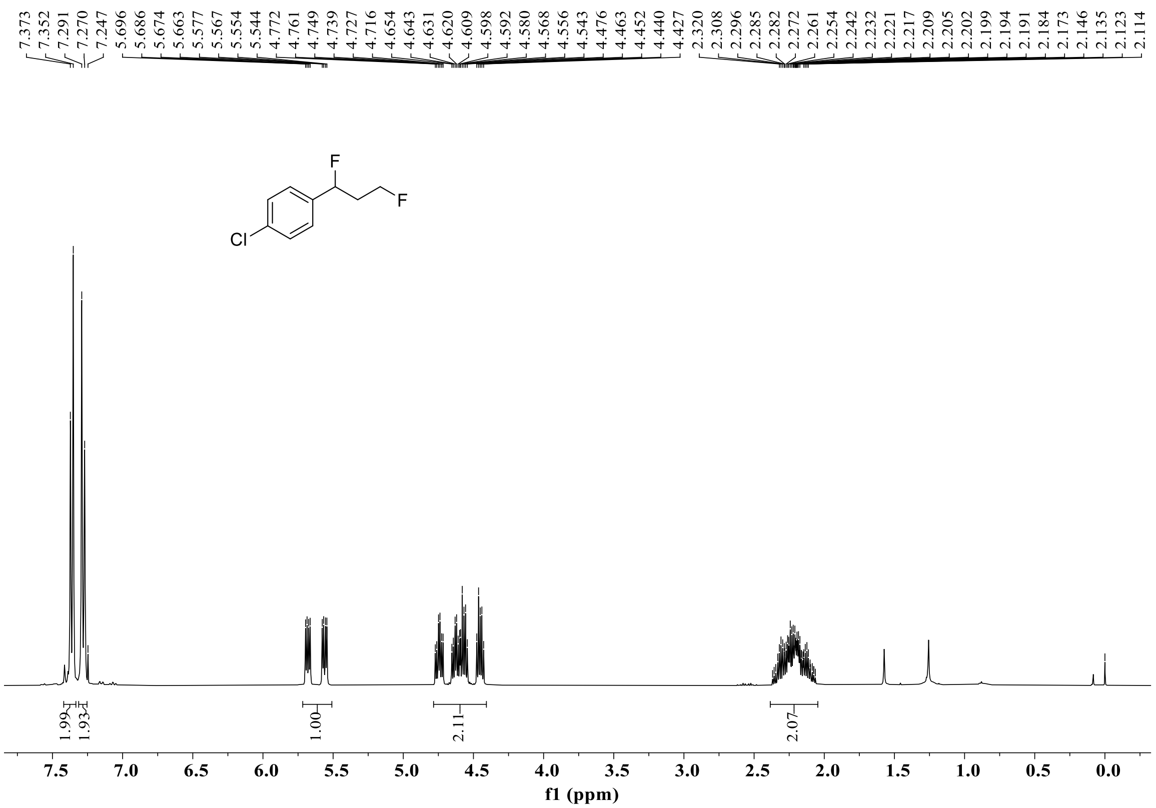

Supplementary Figure 18 ^{1}H NMR spectra of compound **9**

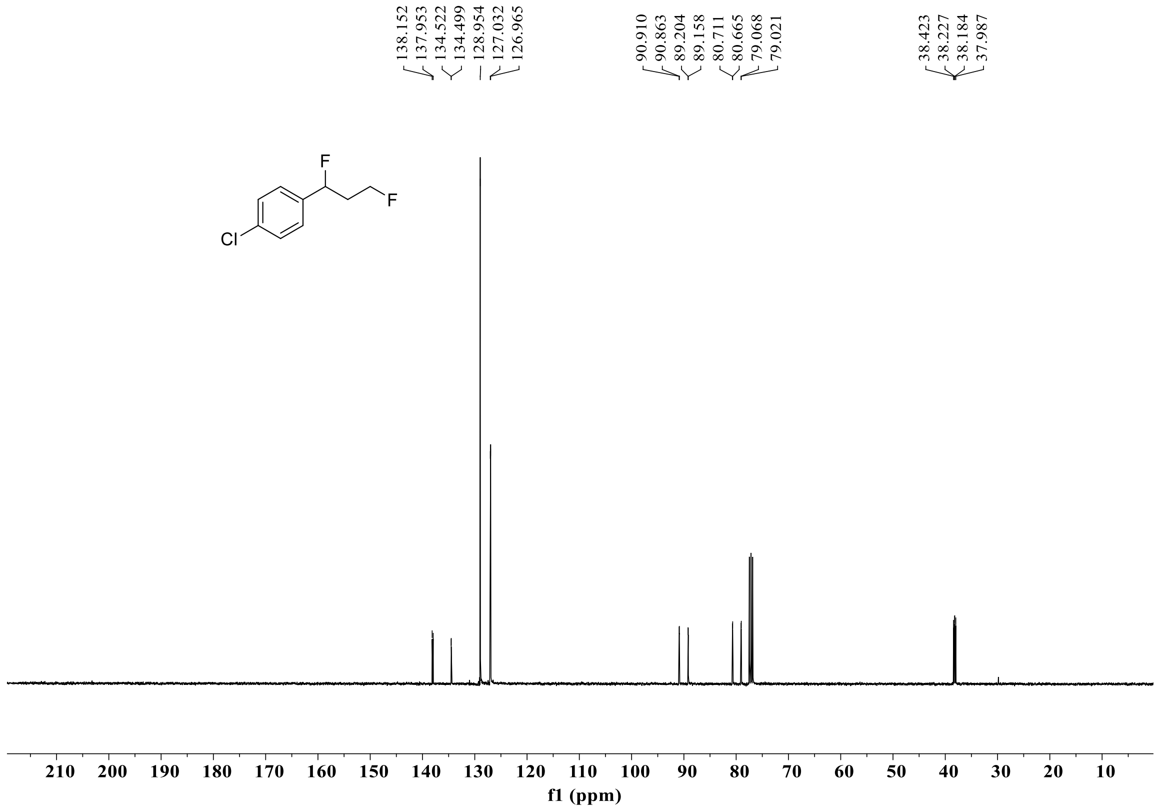

Supplementary Figure 19 ^{13}C NMR spectra of compound **9**

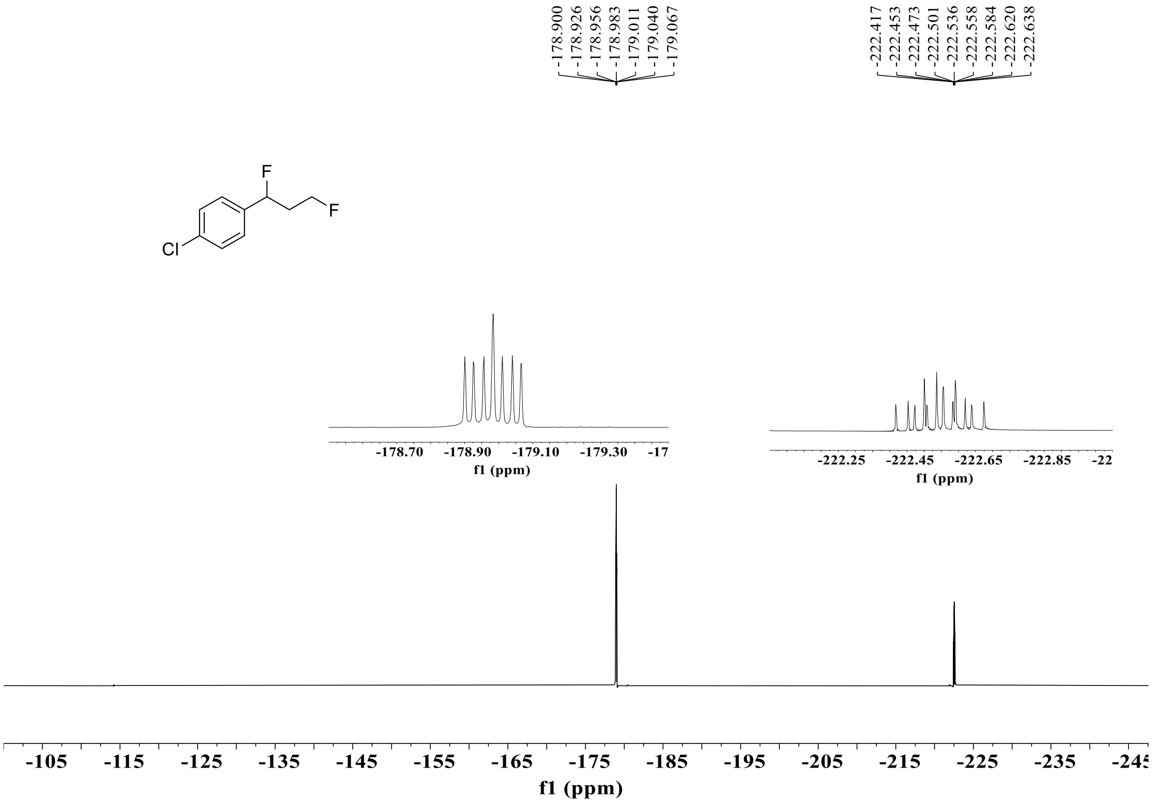

Supplementary Figure 20 ^{19}F NMR spectra of compound **9**

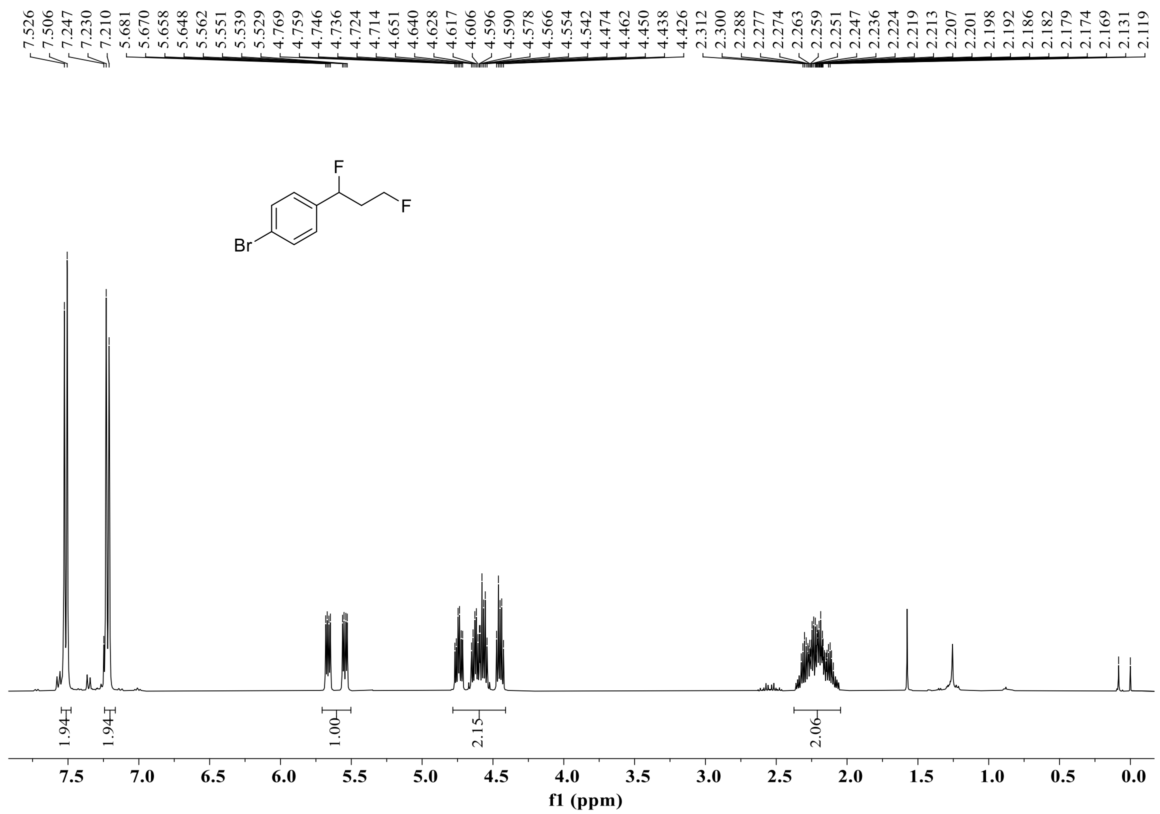

Supplementary Figure 21 ^{1}H NMR spectra of compound **10**

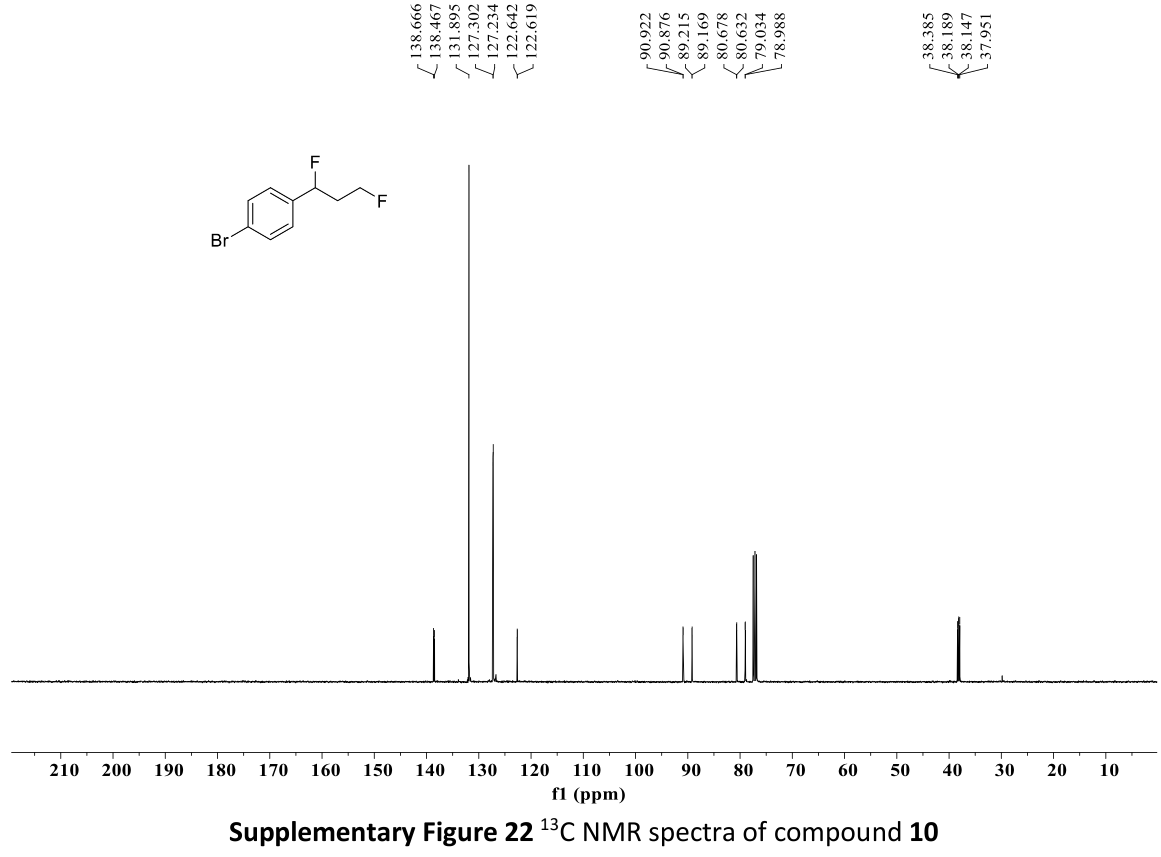

Supplementary Figure 22 ^{13}C NMR spectra of compound **10**

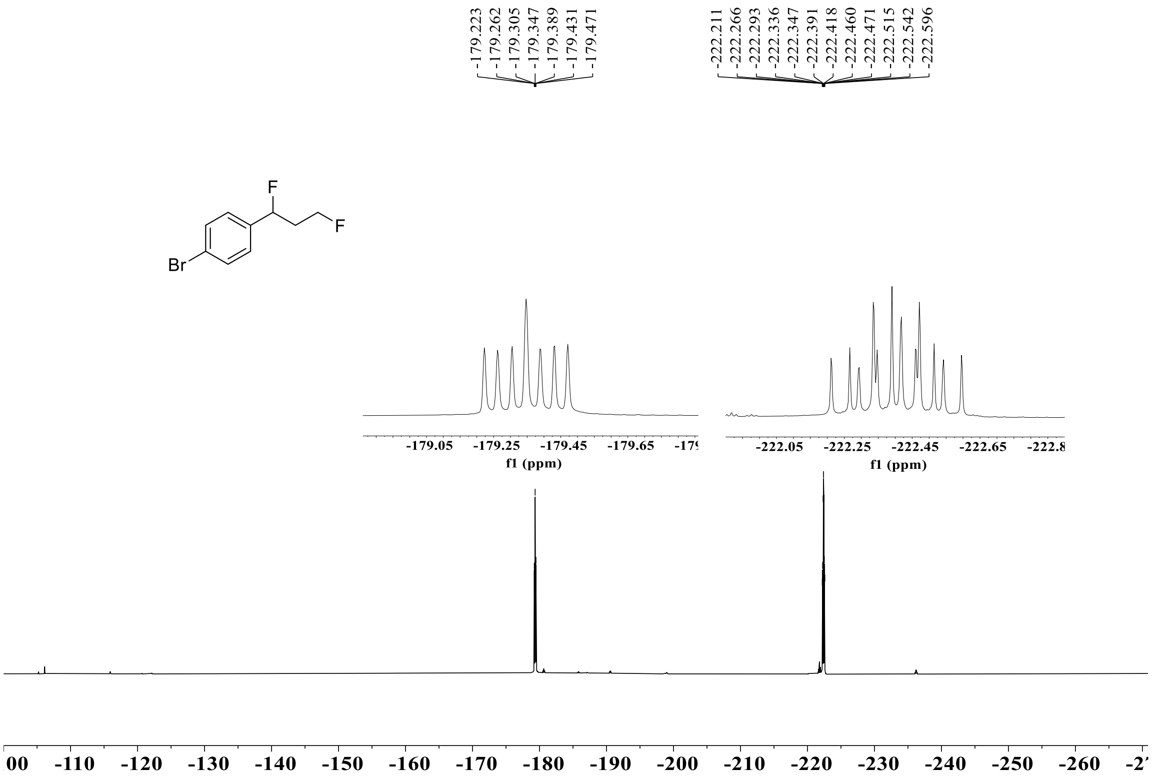

Supplementary Figure 23 ^{19}F NMR spectra of compound **10**

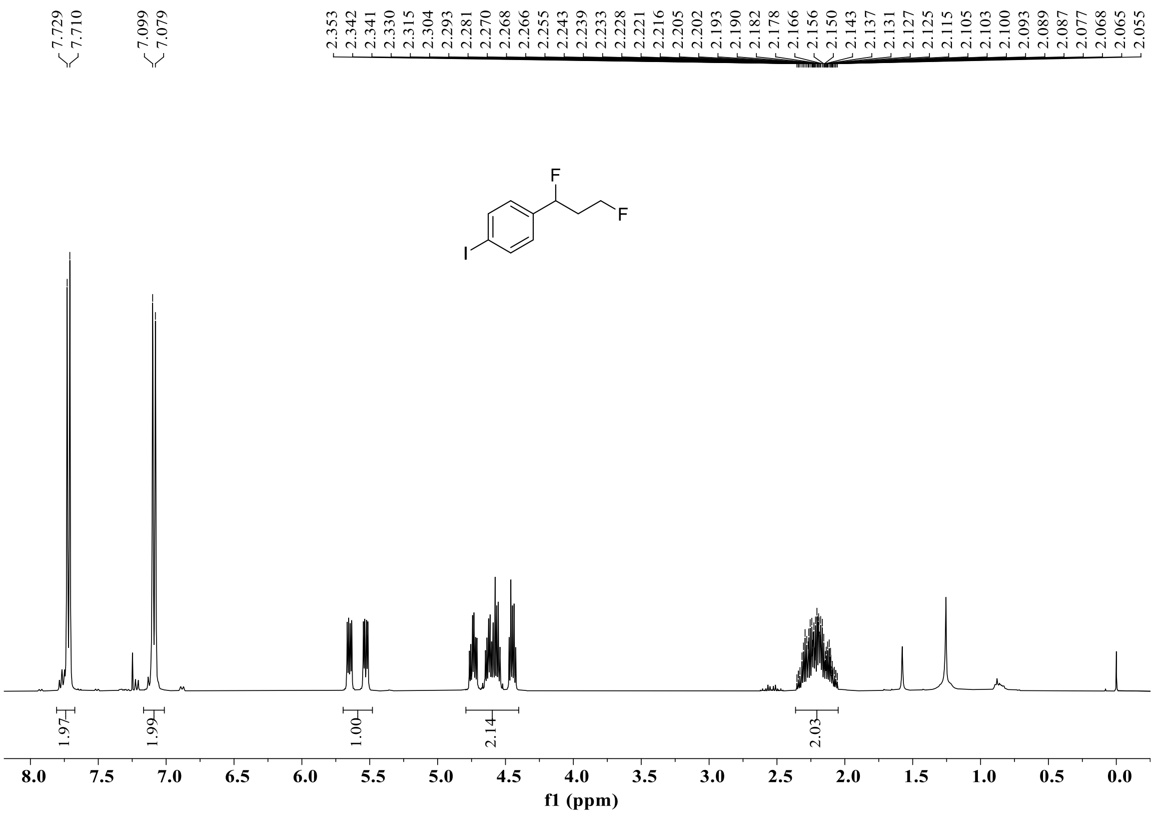

Supplementary Figure 24 ^{1}H NMR spectra of compound **11**

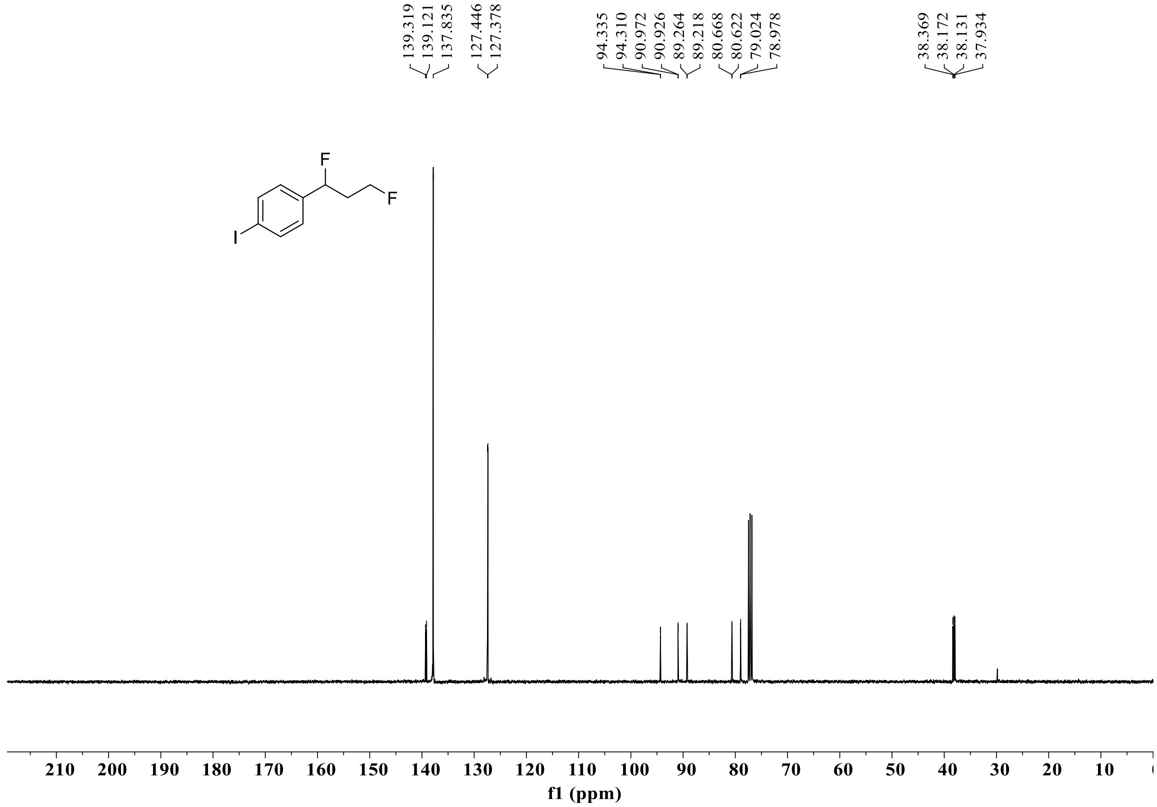

Supplementary Figure 25 ^{13}C NMR spectra of compound **11**

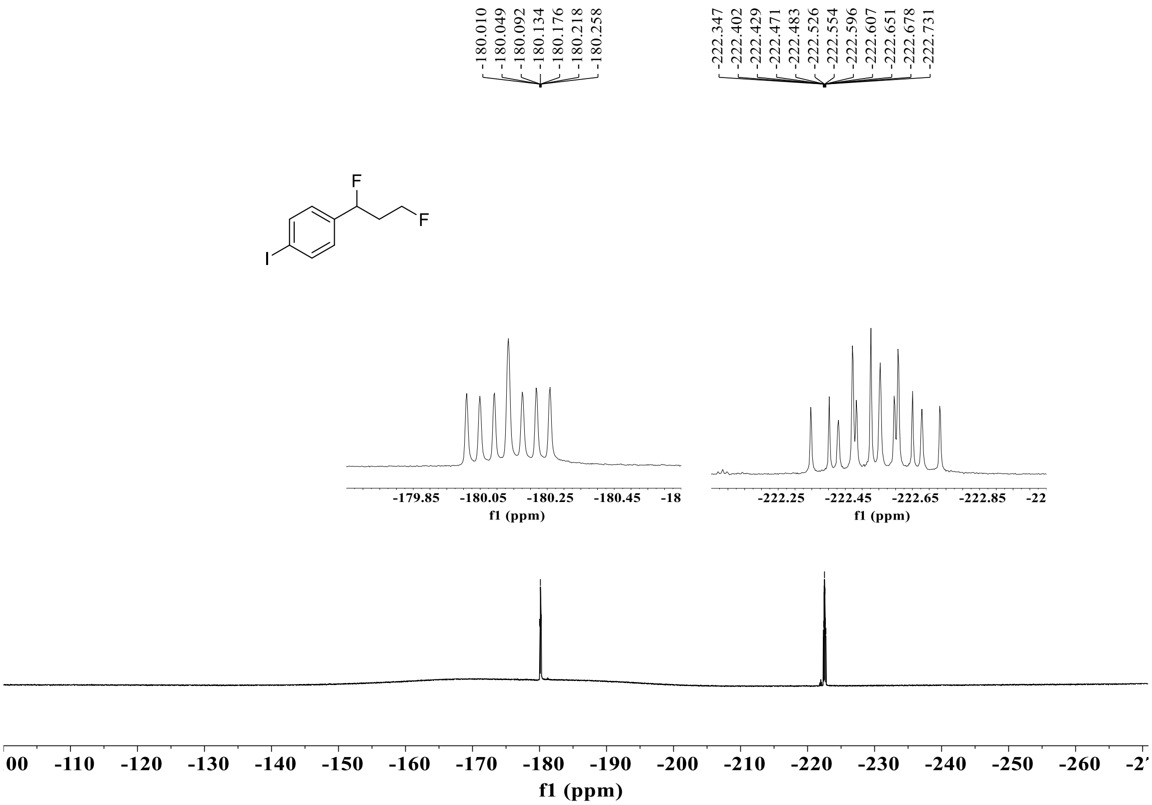

Supplementary Figure 26 ^{19}F NMR spectra of compound **11**

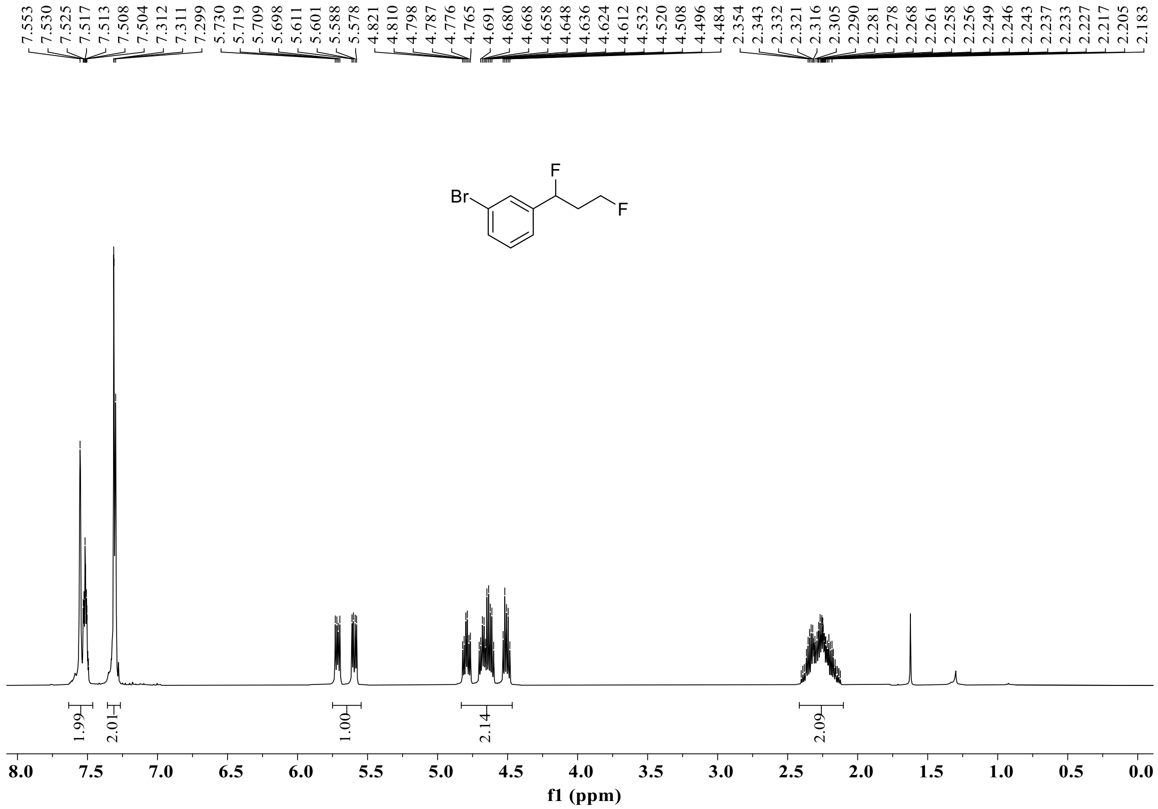

Supplementary Figure 27 ^{1}H NMR spectra of compound **12**

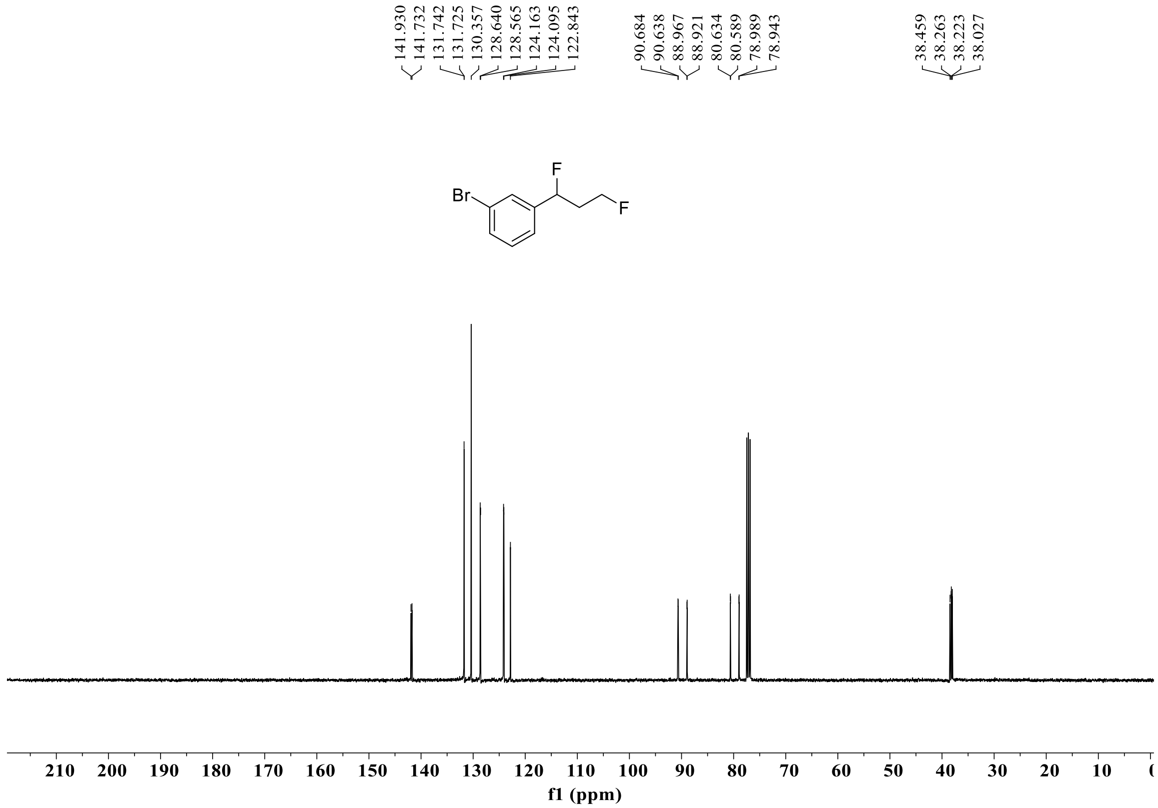

Supplementary Figure 28 ^{13}C NMR spectra of compound **12**

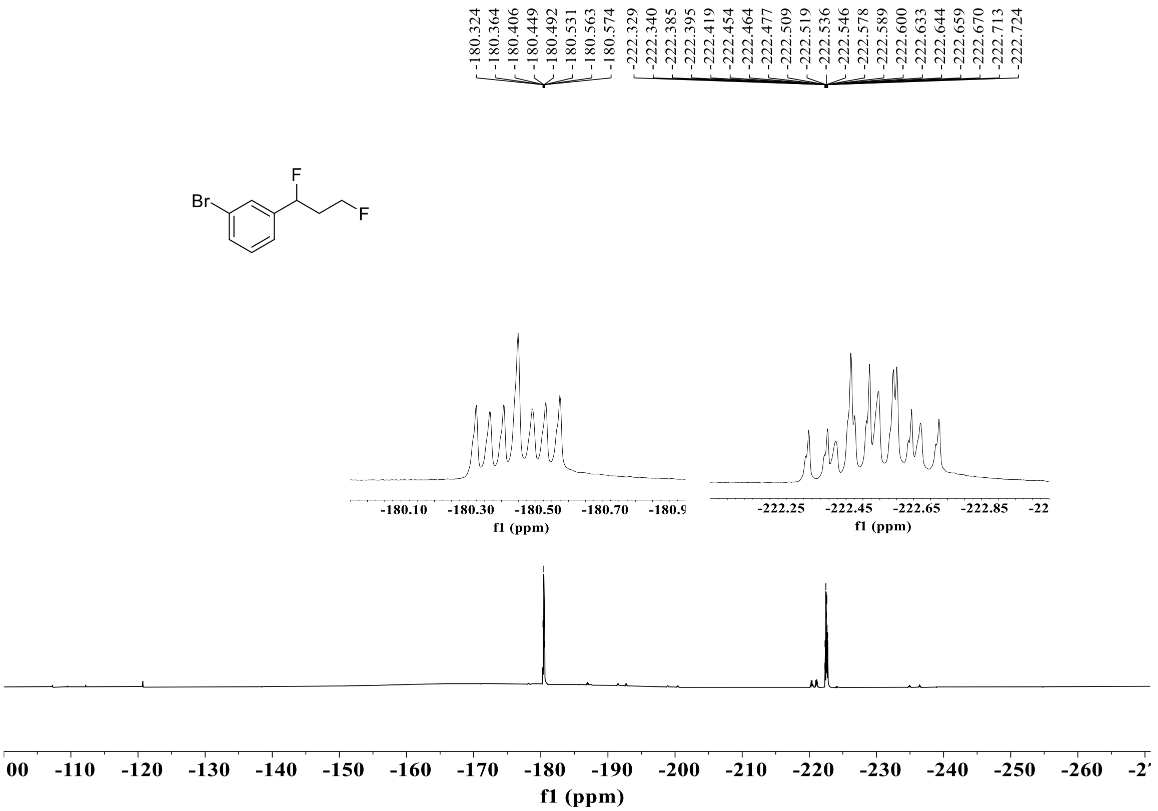

Supplementary Figure 29 ^{19}F NMR spectra of compound **12**

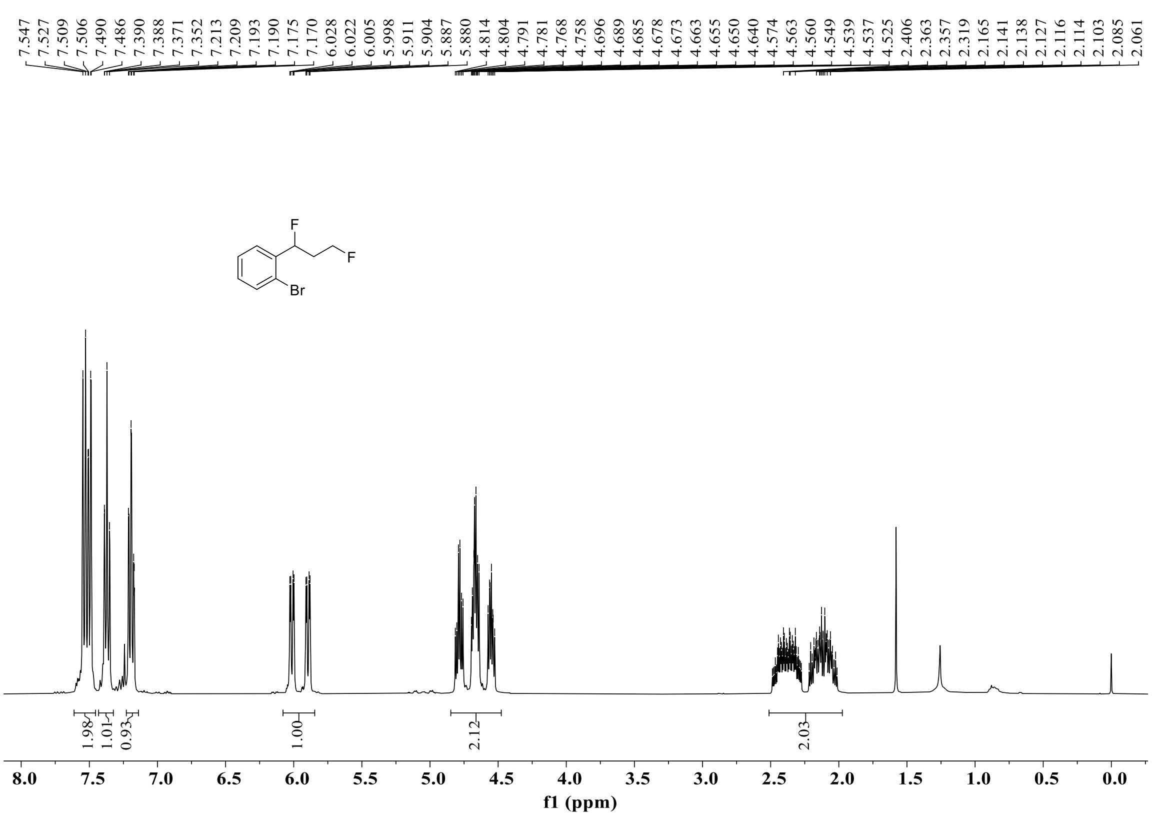

Supplementary Figure 30 ^{1}H NMR spectra of compound **13**

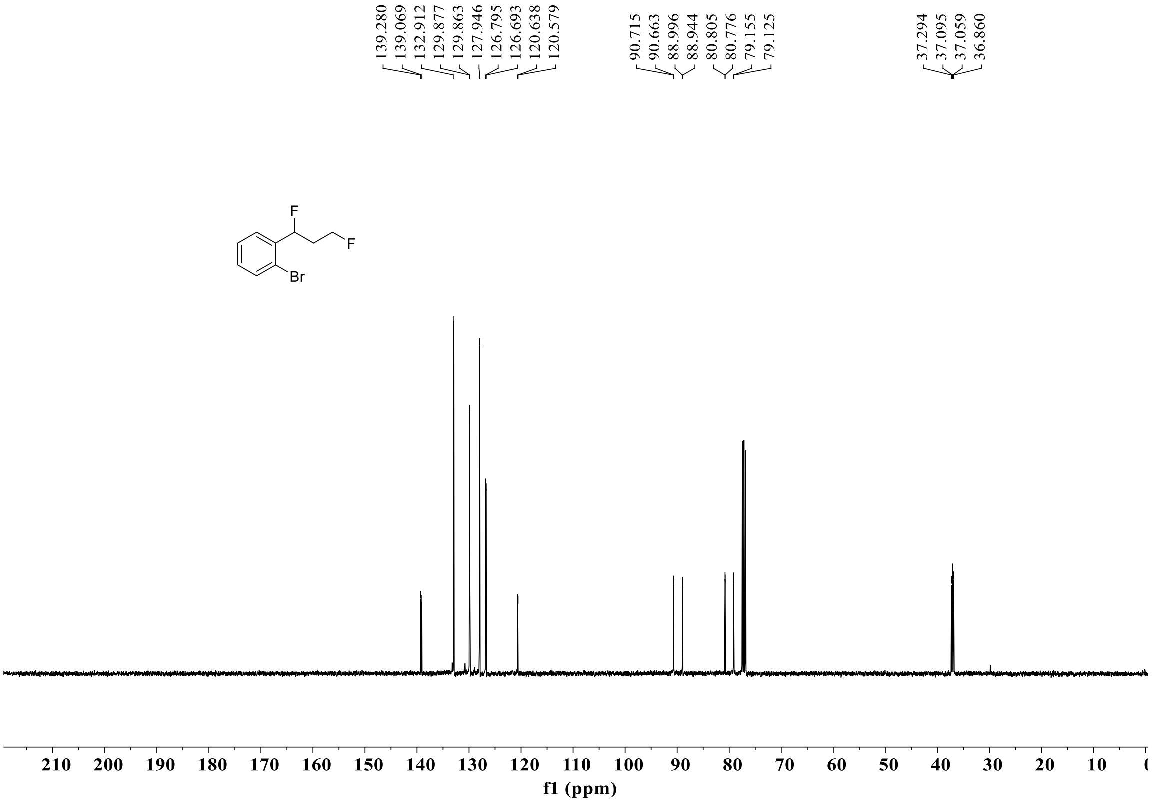

Supplementary Figure 31 ^{13}C NMR spectra of compound **13**

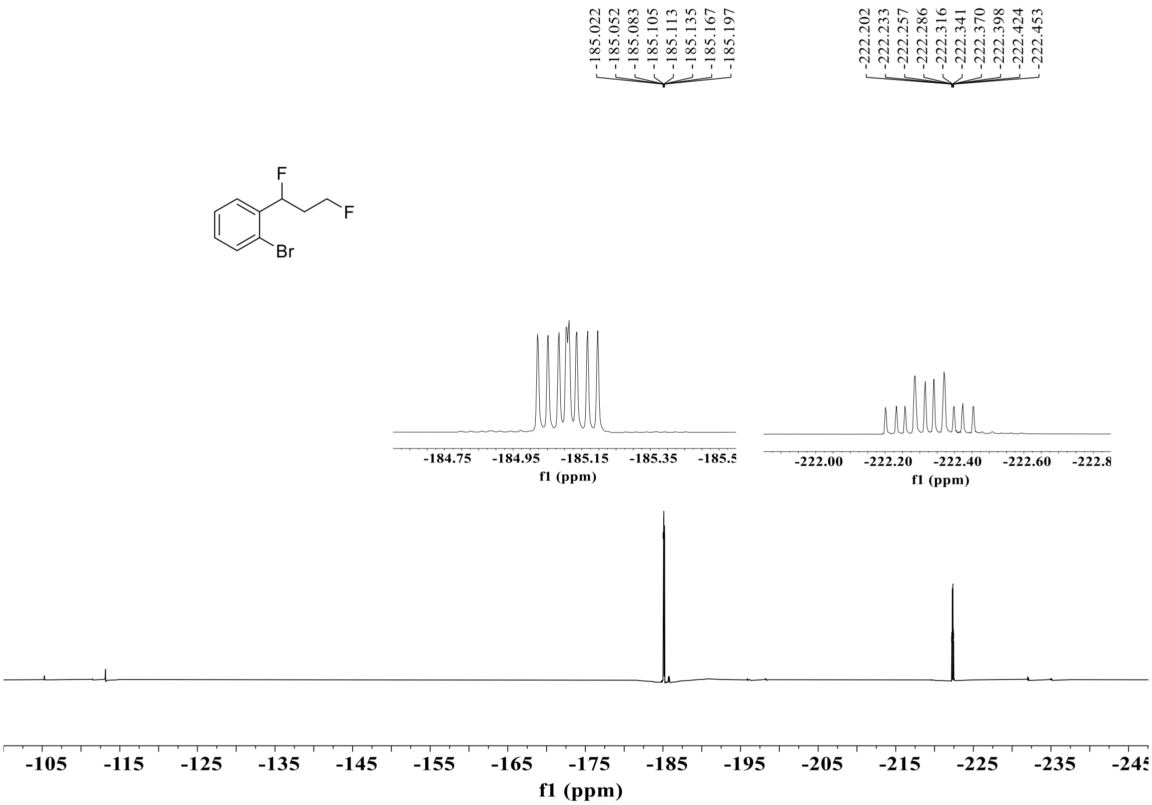

Supplementary Figure 32 ^{19}F NMR spectra of compound **13**

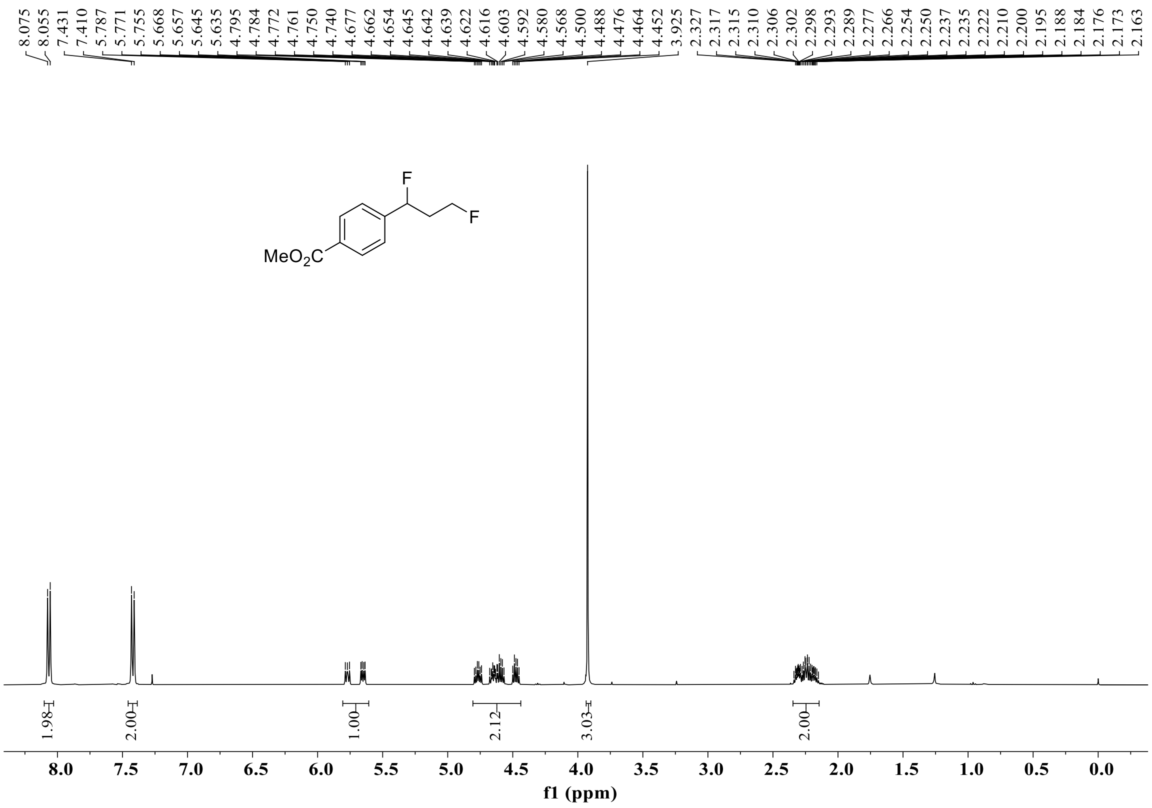

Supplementary Figure 33 ^{1}H NMR spectra of compound **14**

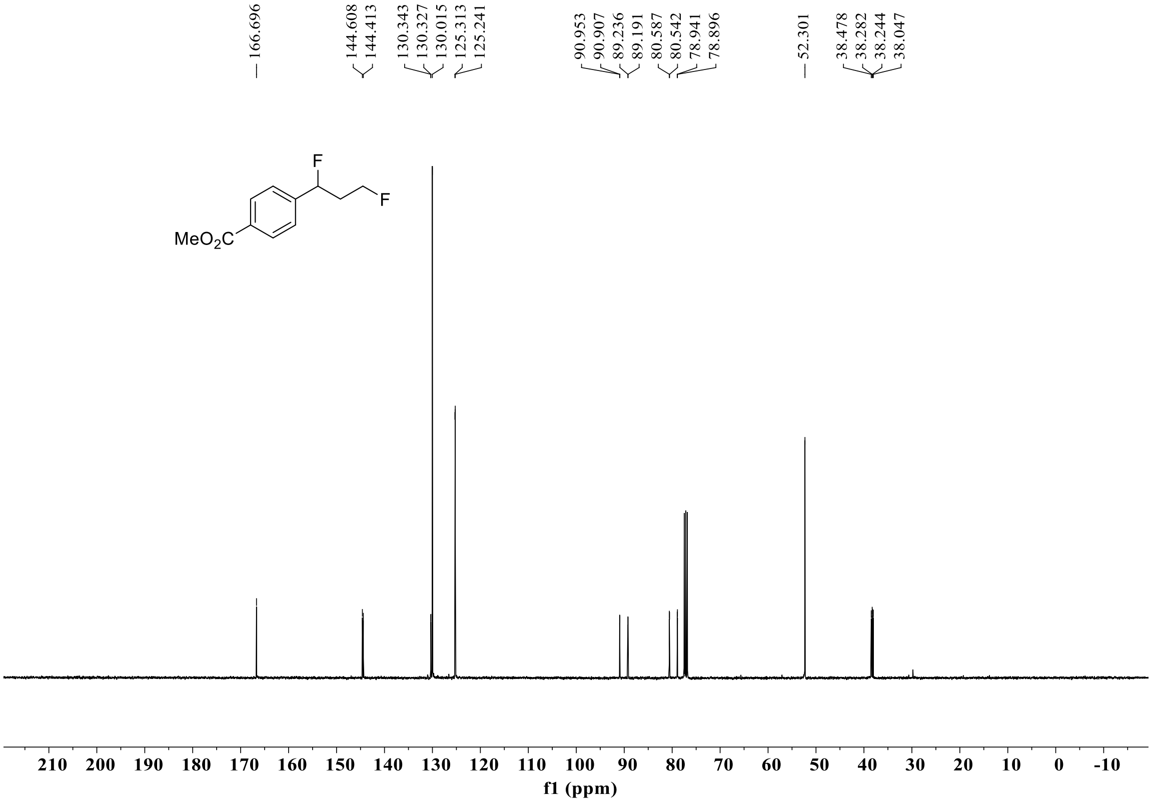

Supplementary Figure 34 ^{13}C NMR spectra of compound **14**

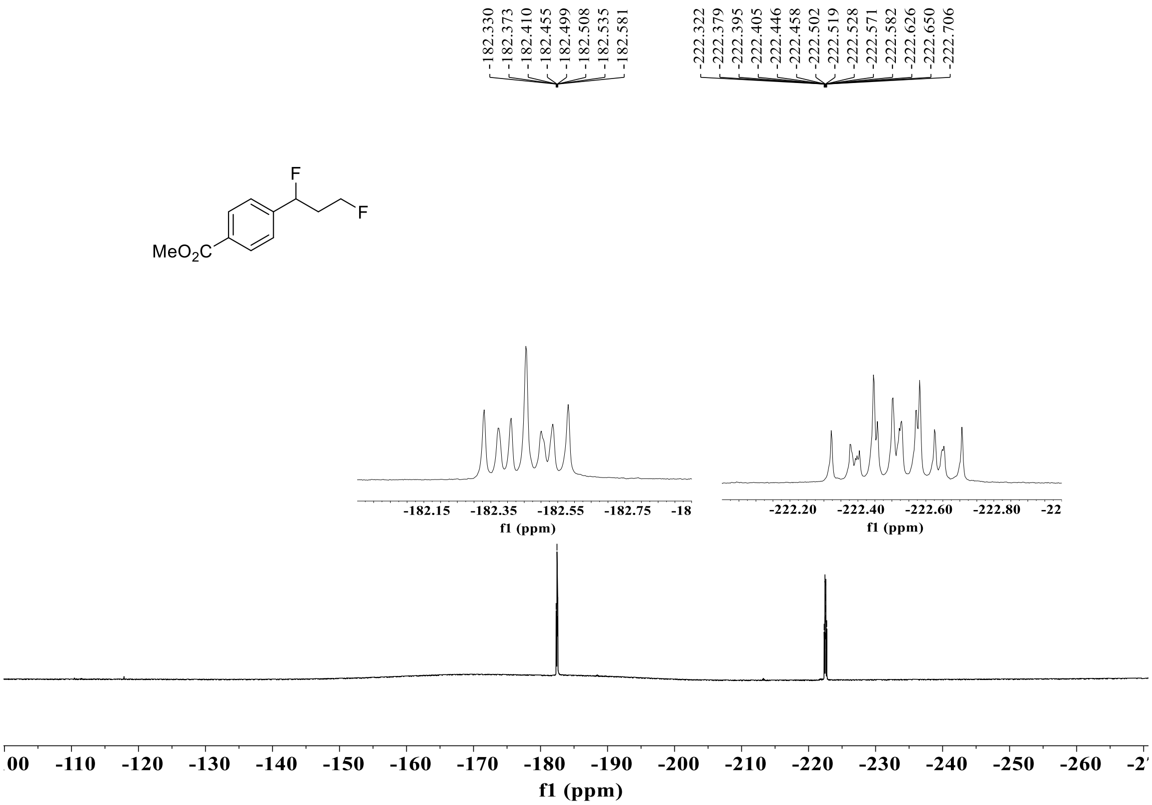

Supplementary Figure 35 ^{19}F NMR spectra of compound **14**

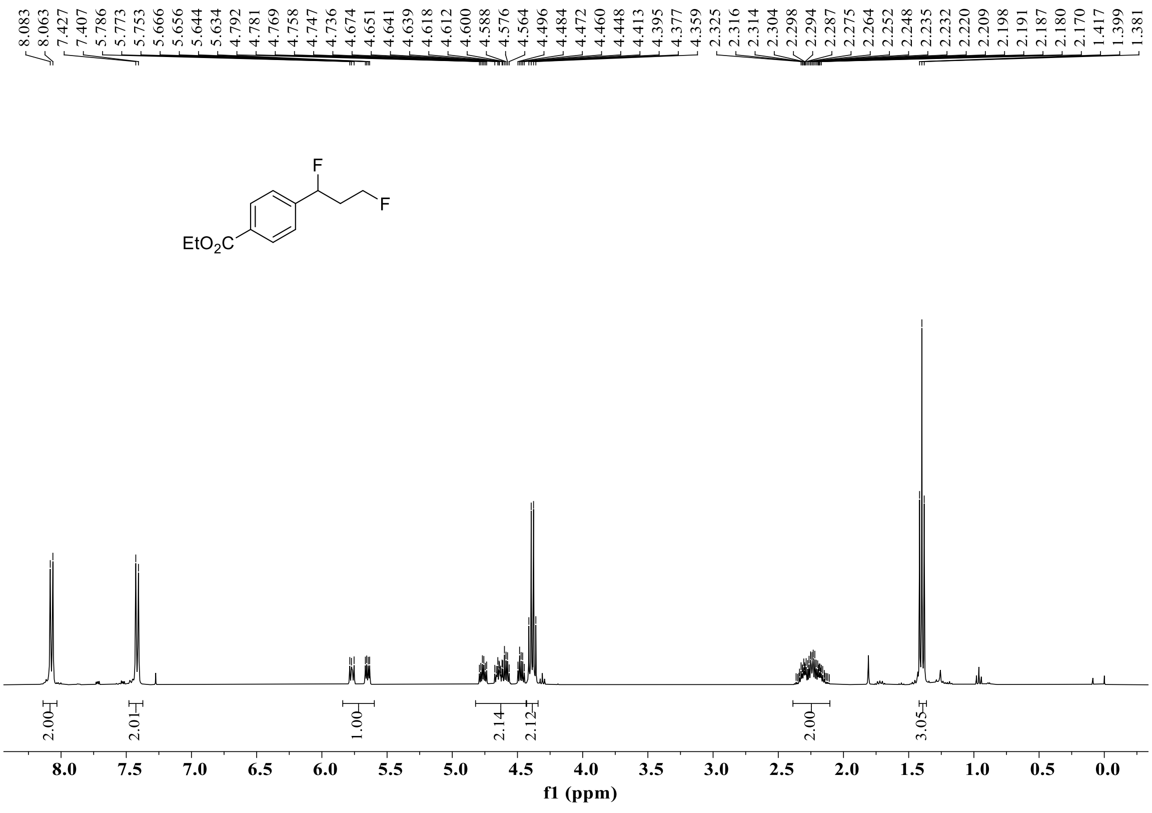

Supplementary Figure 36 ^{1}H NMR spectra of compound **15**

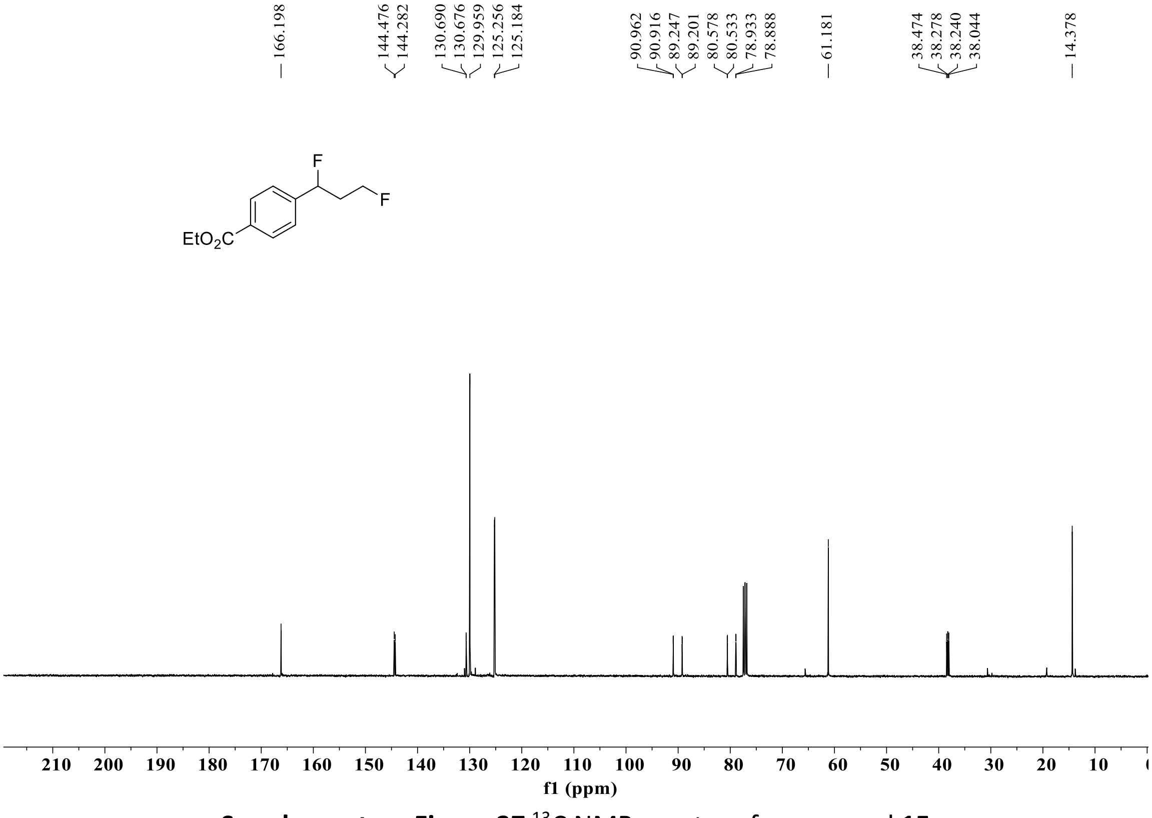

Supplementary Figure 37 ^{13}C NMR spectra of compound **15**

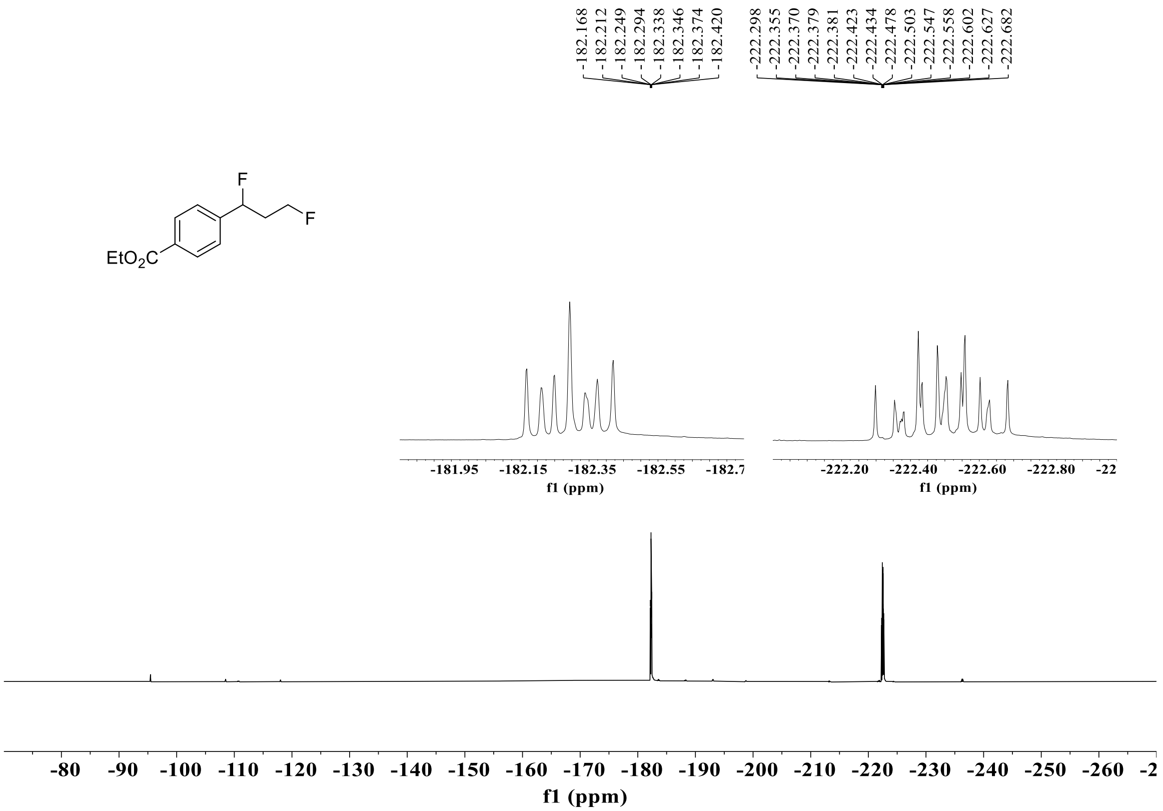

Supplementary Figure 38 ^{19}F NMR spectra of compound **15**

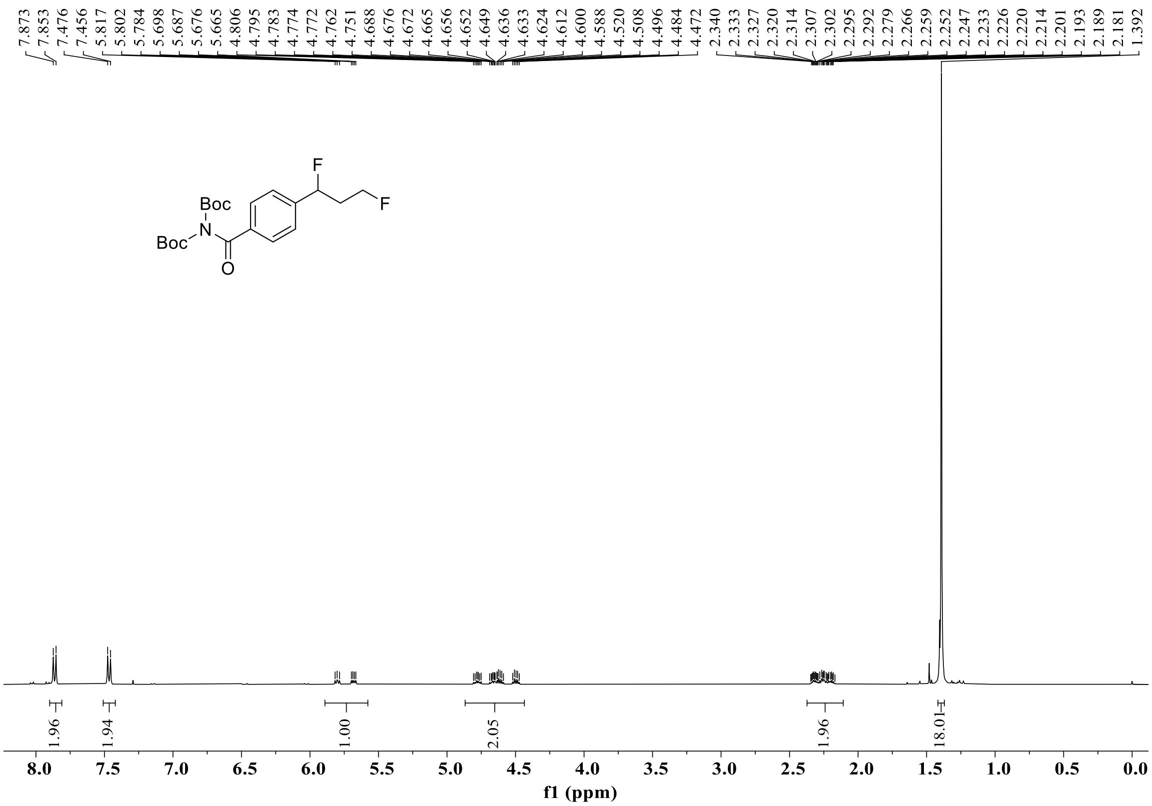

Supplementary Figure 39 ^{1}H NMR spectra of compound **16**

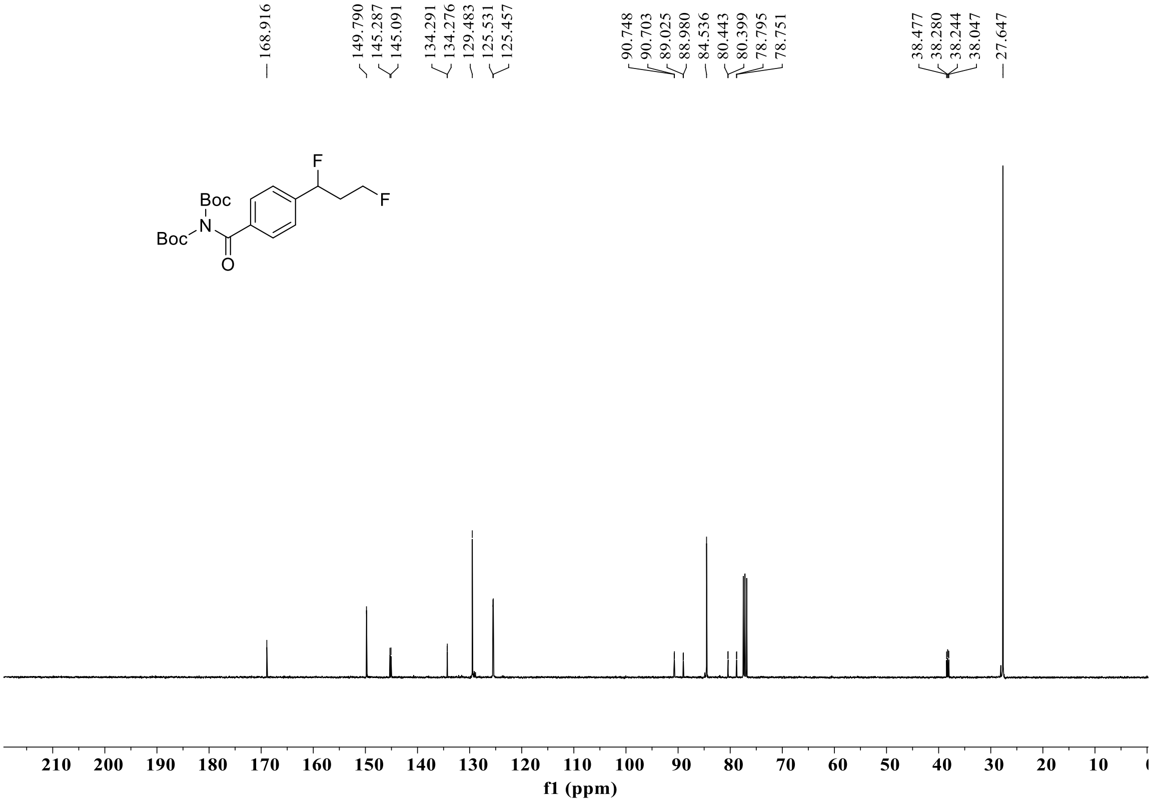

Supplementary Figure 40 ^{13}C NMR spectra of compound **16**

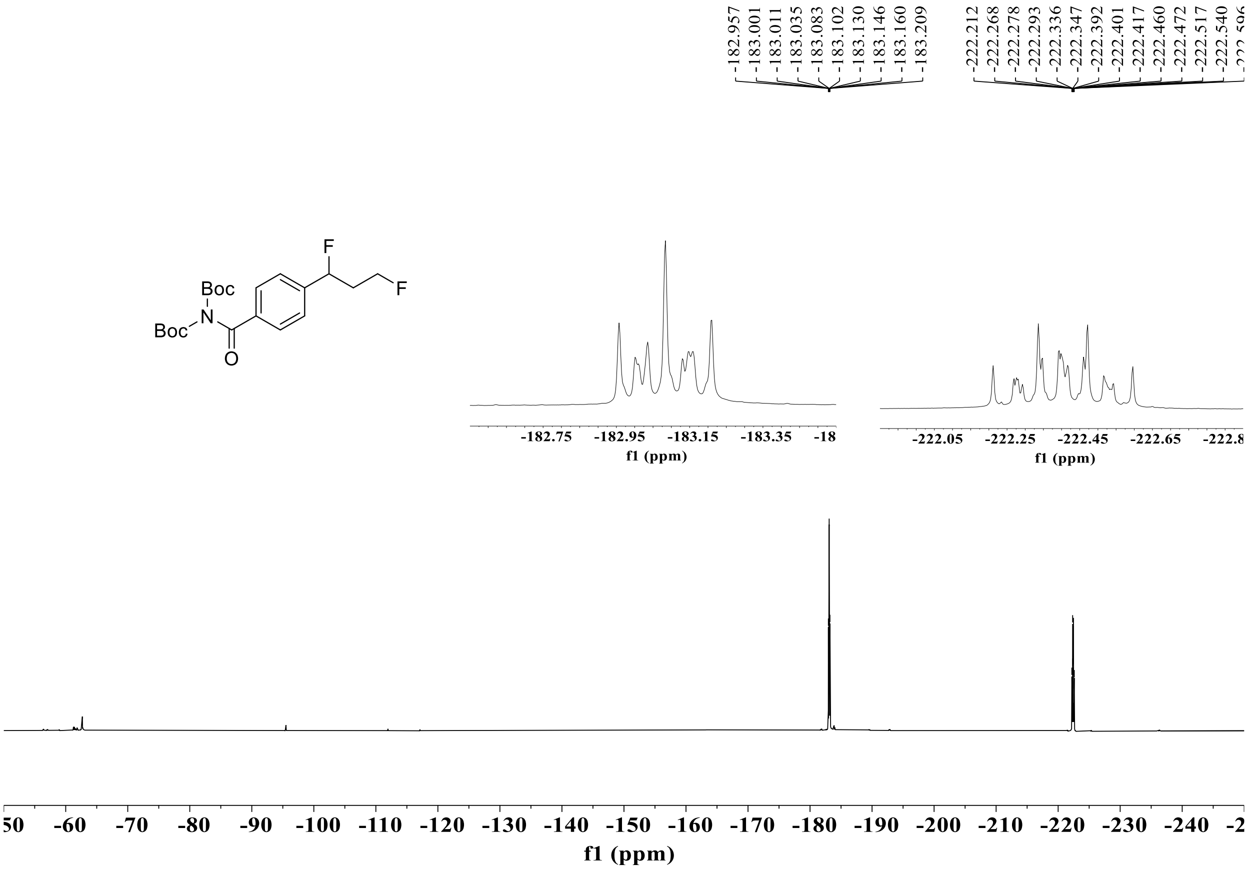

Supplementary Figure 41 ^{19}F NMR spectra of compound **16**

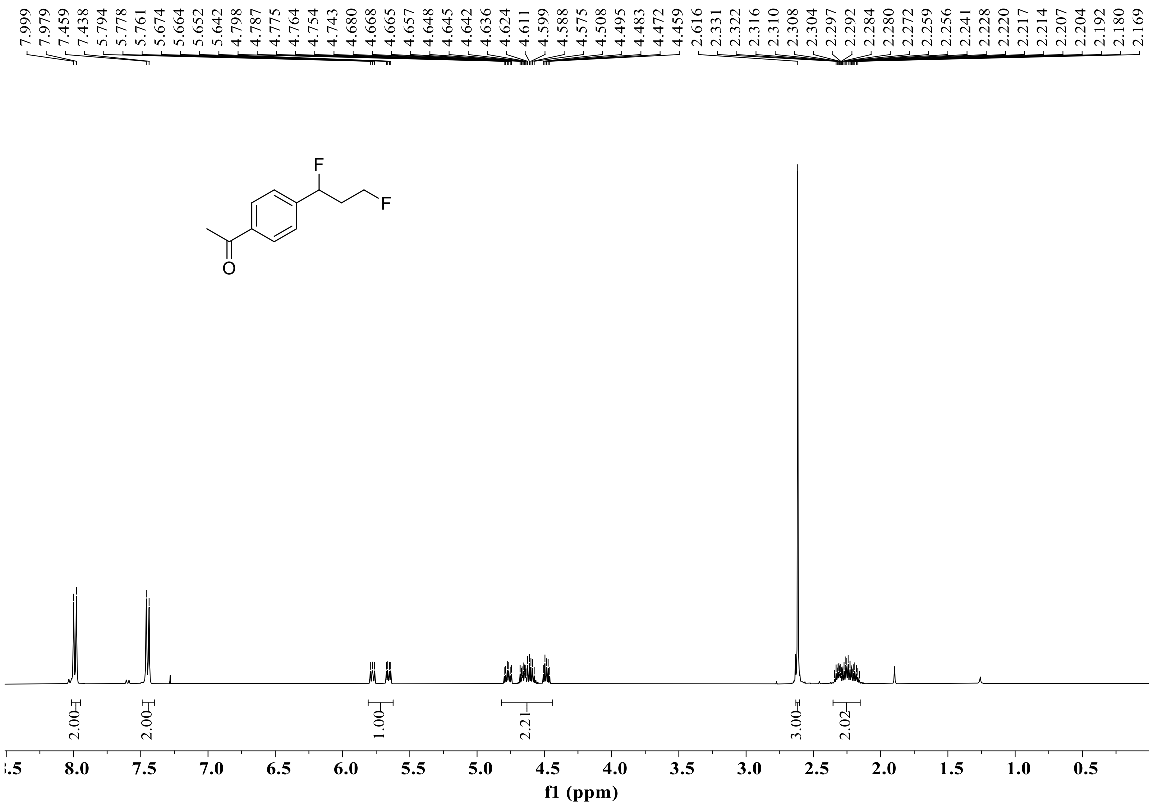

Supplementary Figure 42 ^{1}H NMR spectra of compound **17**

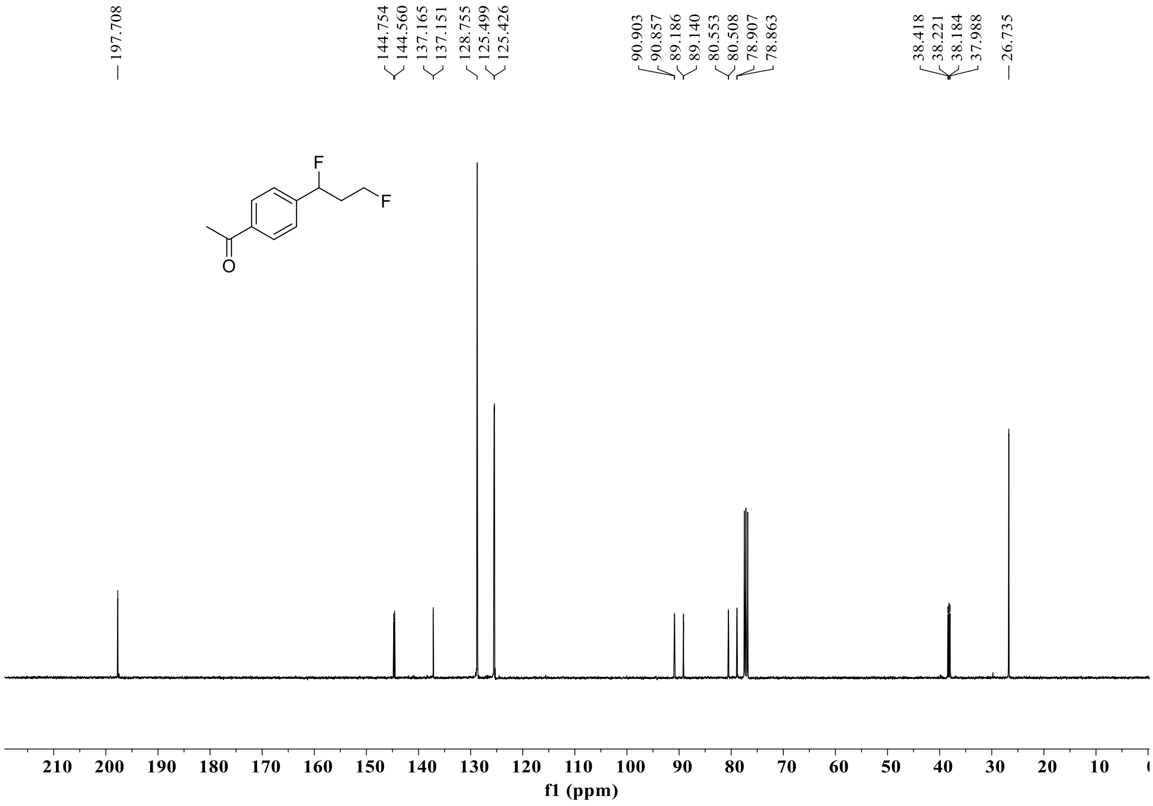

Supplementary Figure 43 ^{13}C NMR spectra of compound **17**

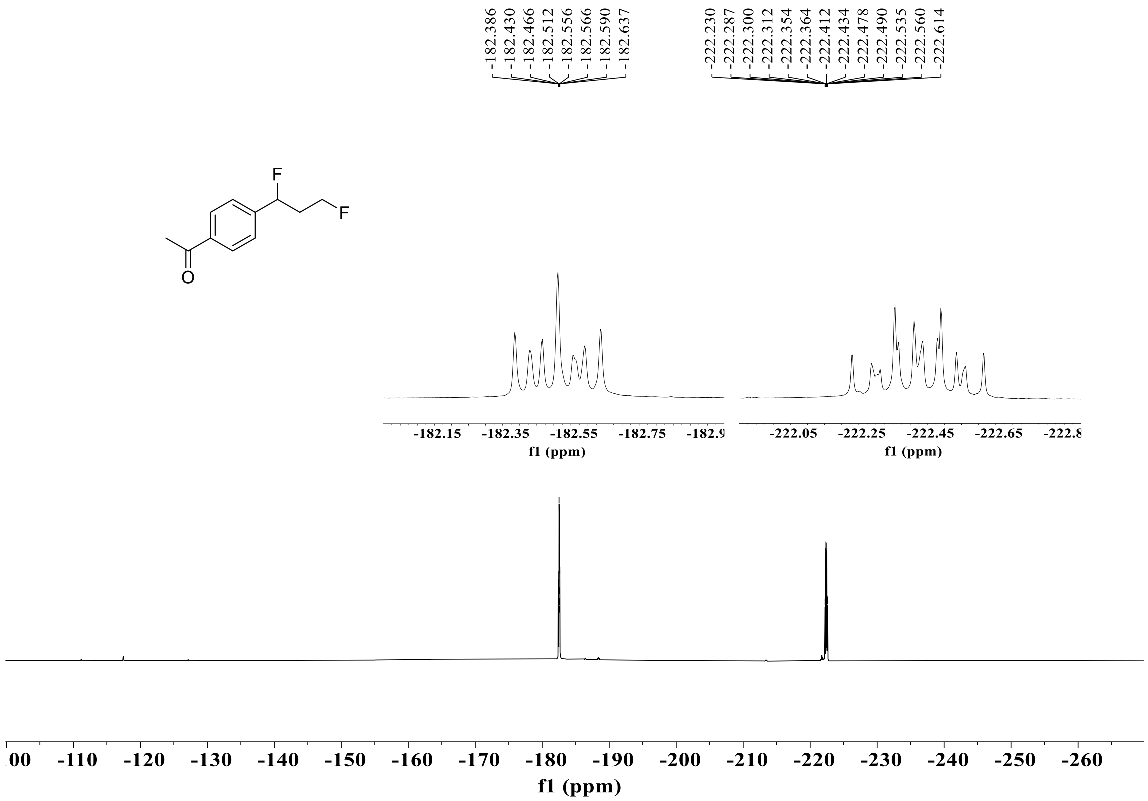

Supplementary Figure 44 ^{19}F NMR spectra of compound **17**

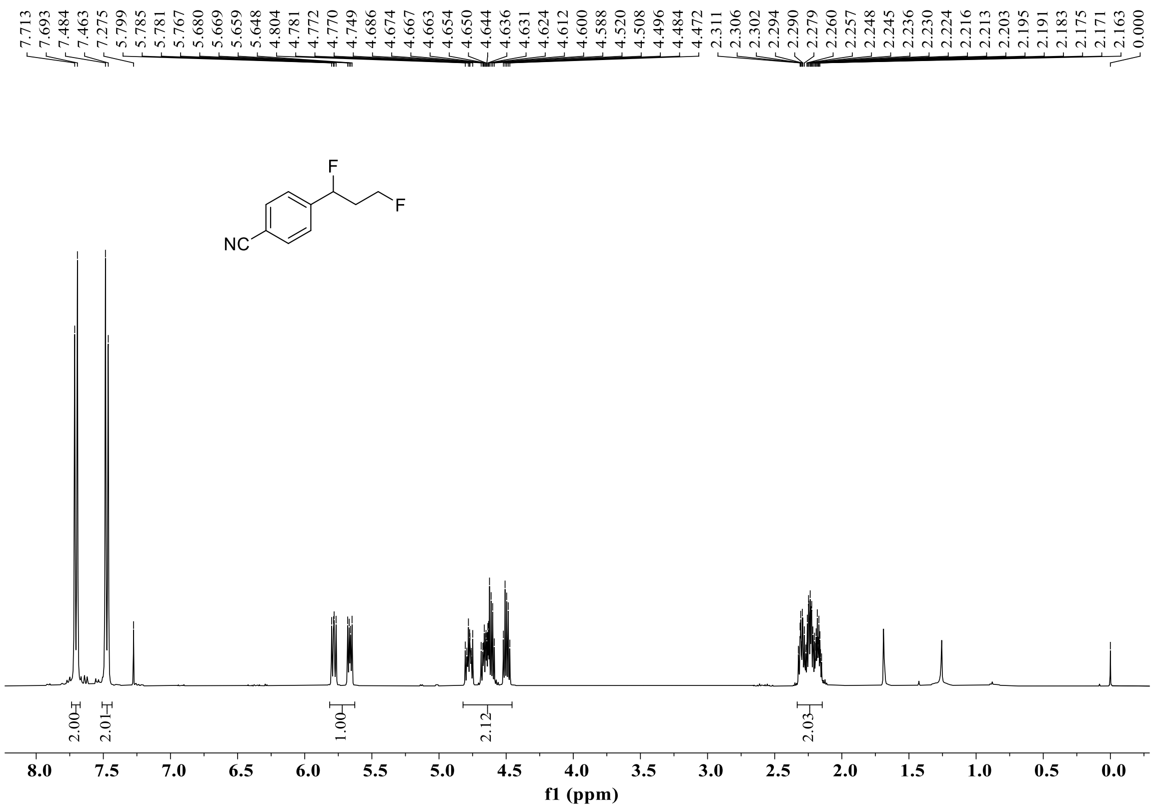

Supplementary Figure 45 ^{1}H NMR spectra of compound **18**

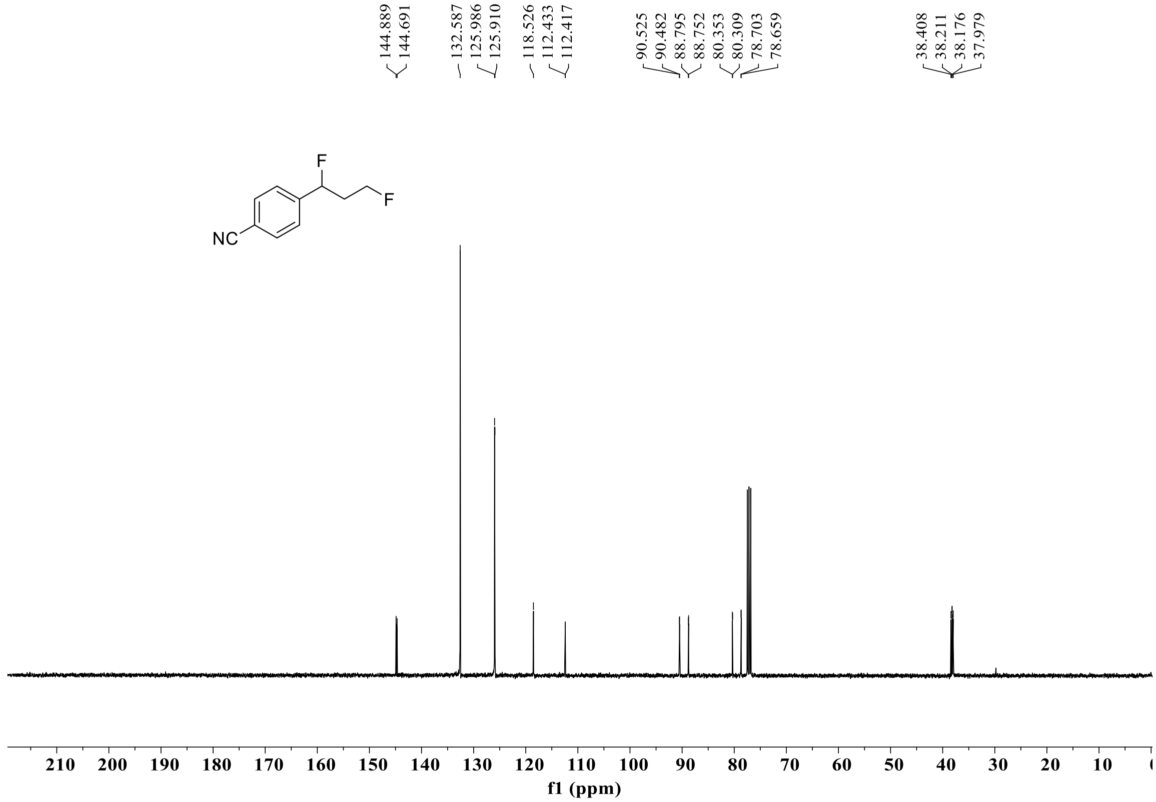

Supplementary Figure 46 ^{13}C NMR spectra of compound **18**

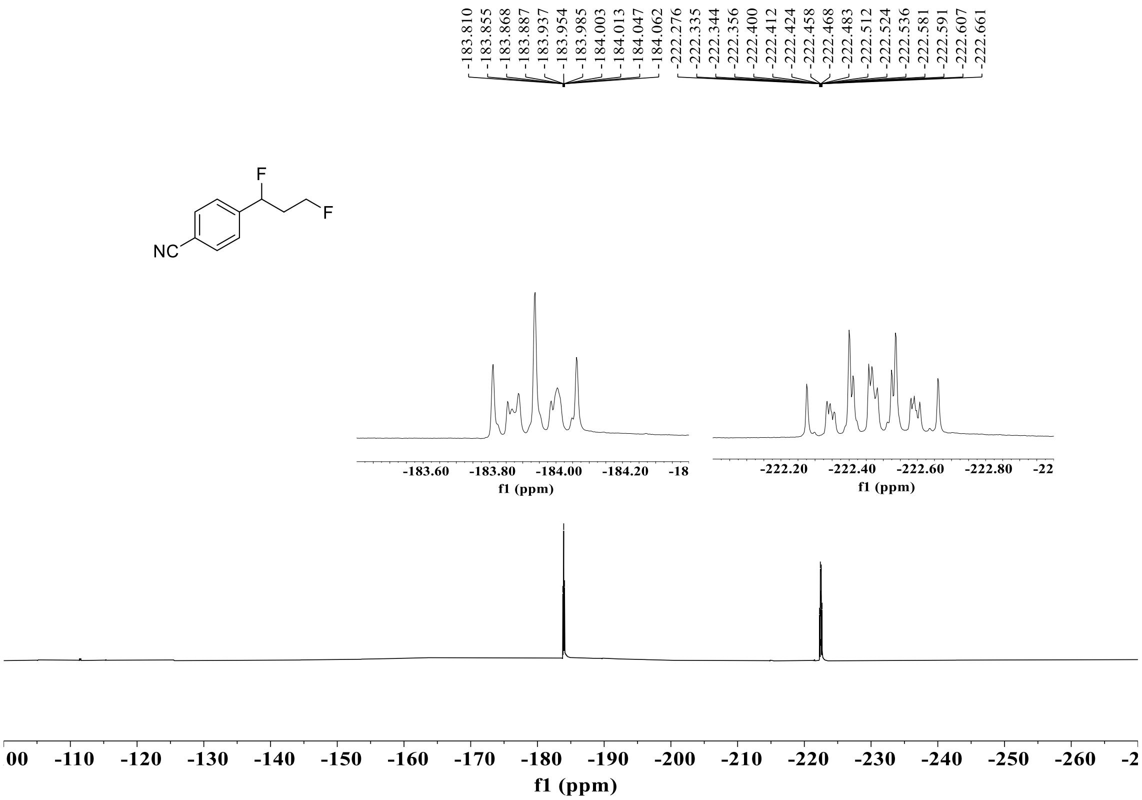

Supplementary Figure 47 ^{19}F NMR spectra of compound **18**

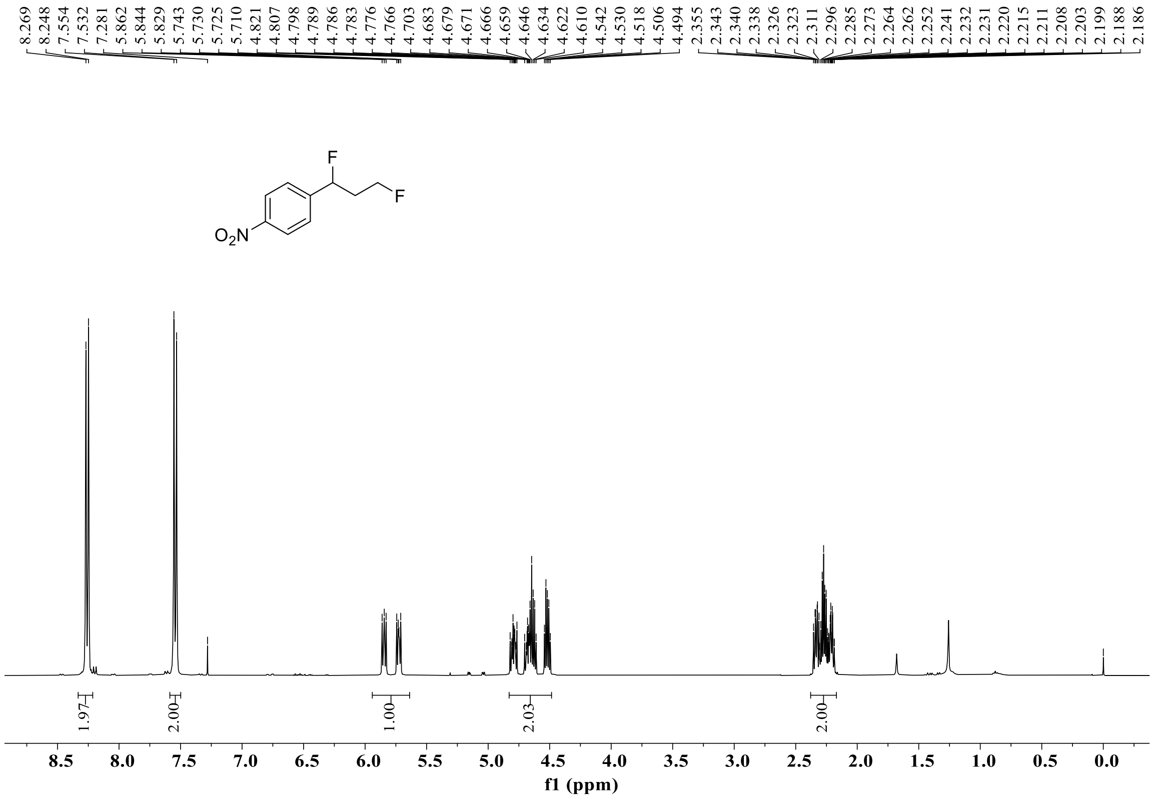

Supplementary Figure 48 ^{1}H NMR spectra of compound **19**

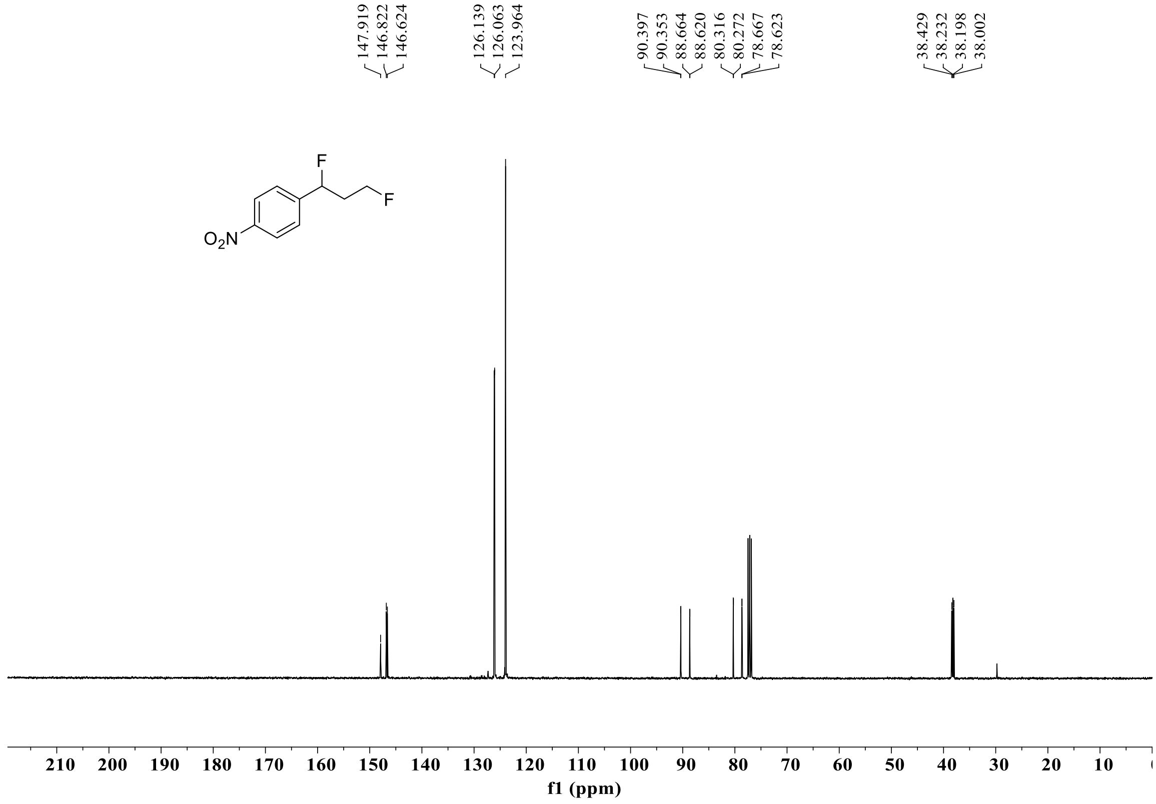

Supplementary Figure 49 ^{13}C NMR spectra of compound **19**

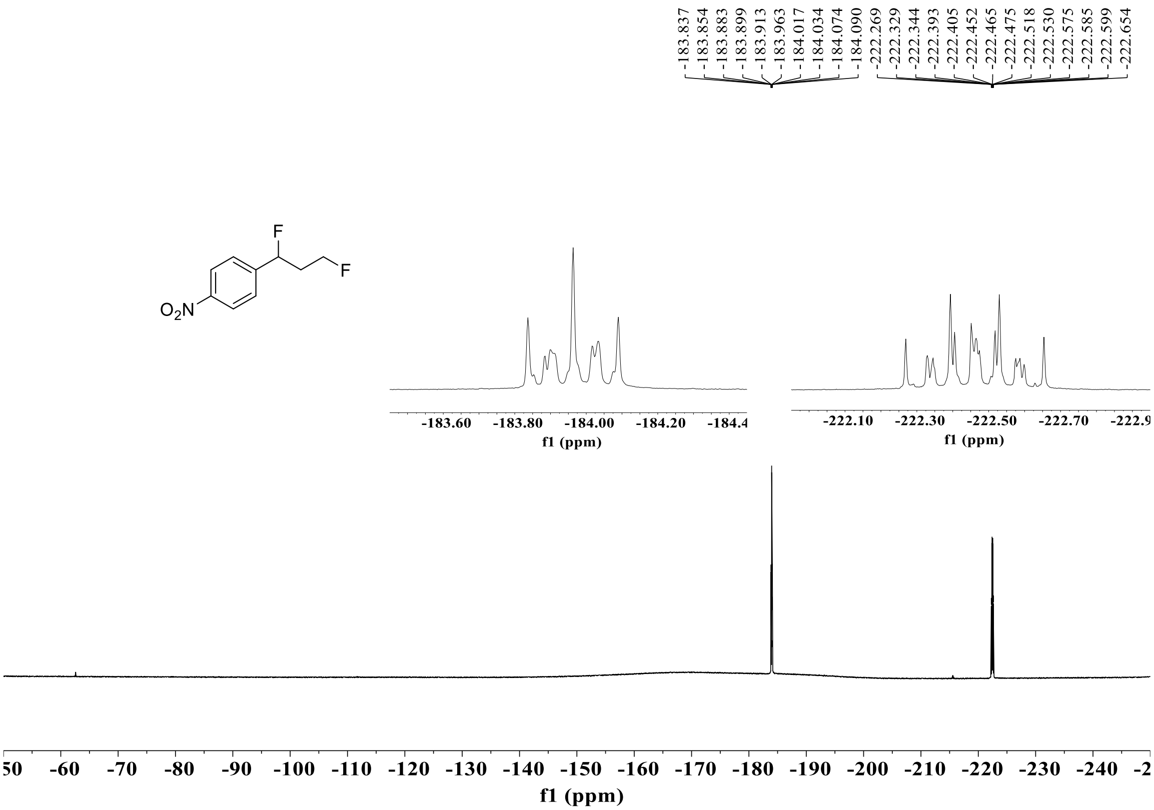

Supplementary Figure 50 ^{19}F NMR spectra of compound **19**

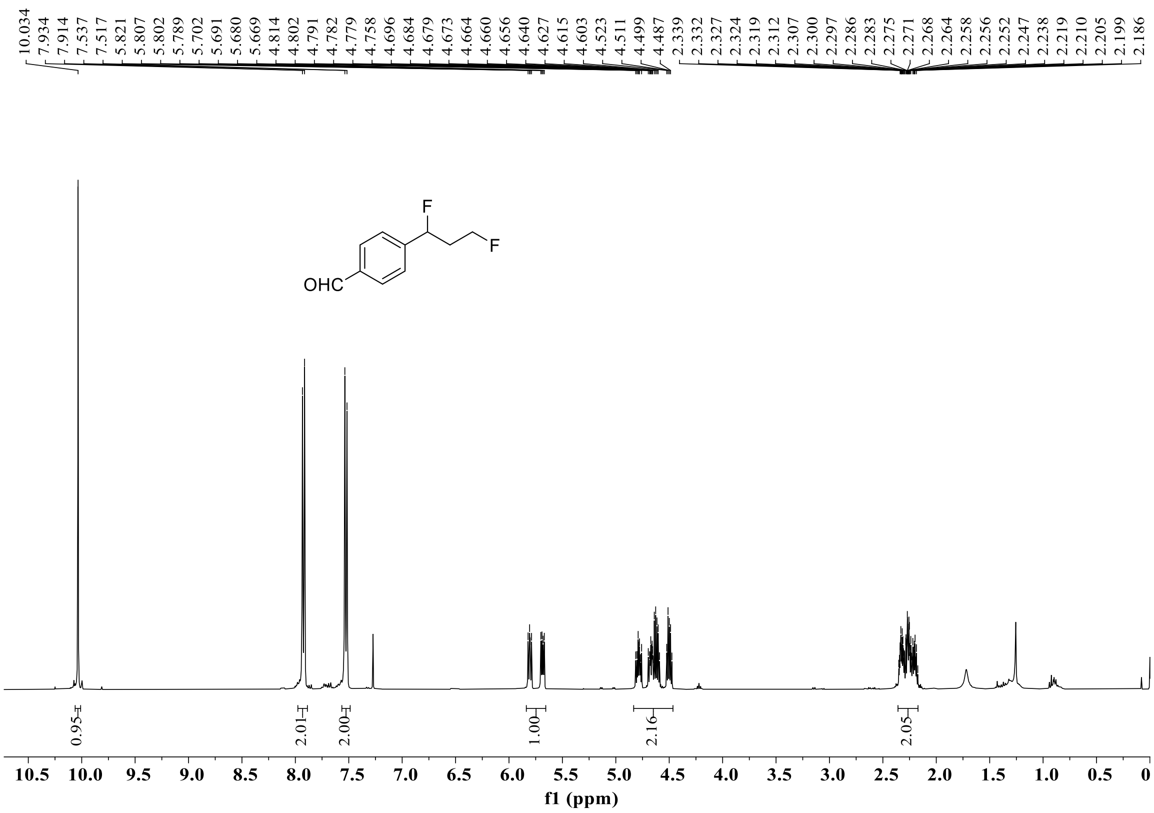

Supplementary Figure 51 ^{1}H NMR spectra of compound **20**

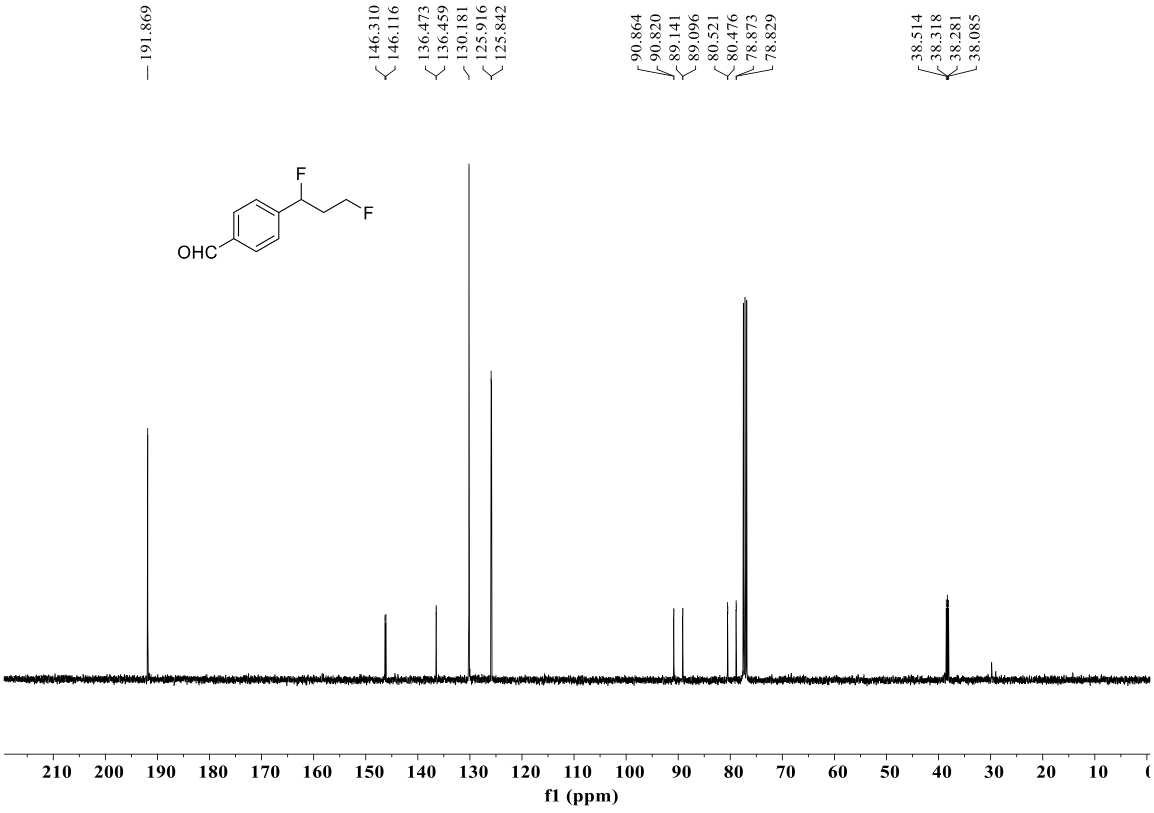

Supplementary Figure 52 ^{13}C NMR spectra of compound **20**

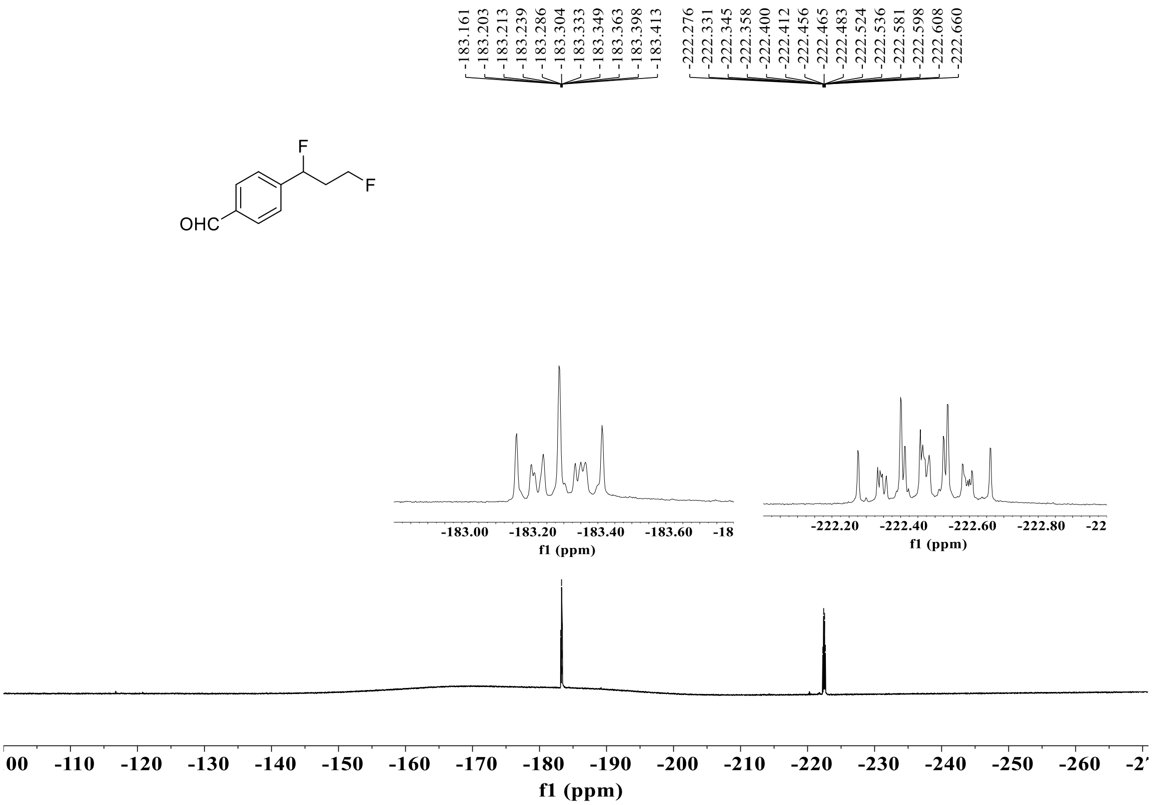

Supplementary Figure 53 ^{19}F NMR spectra of compound **20**

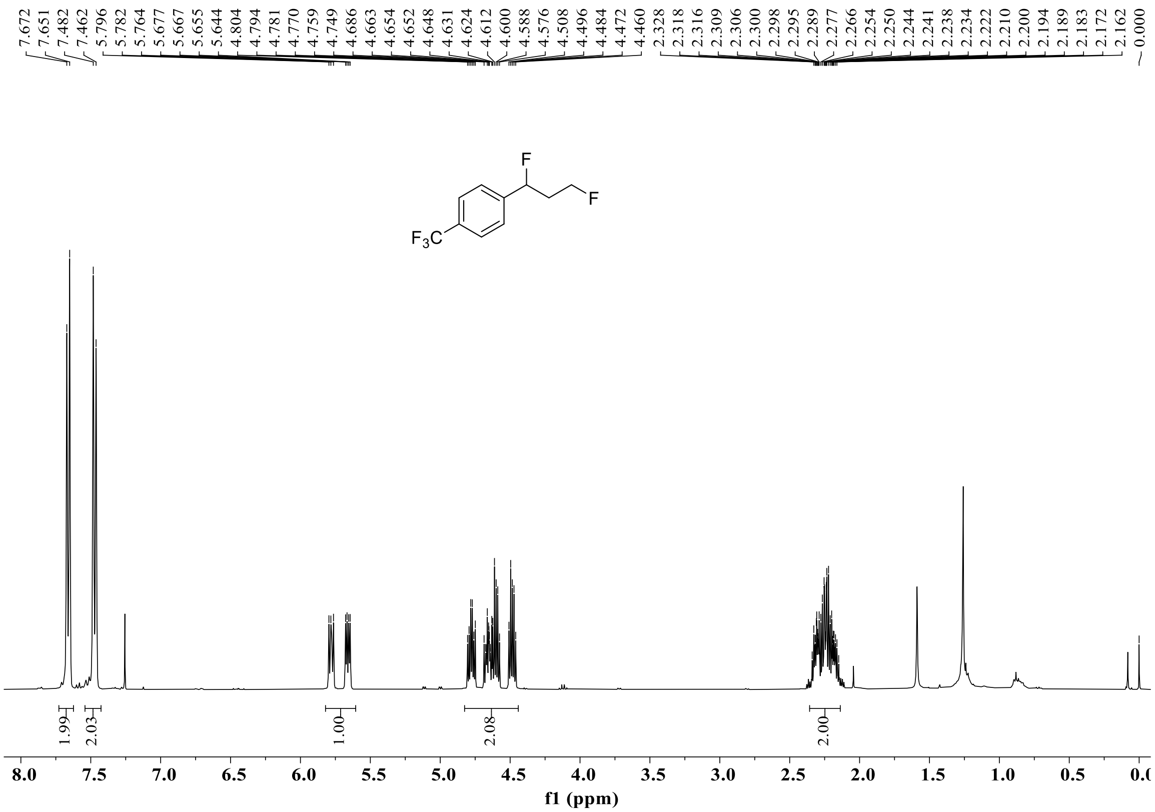

Supplementary Figure 54 ^{1}H NMR spectra of compound **21**

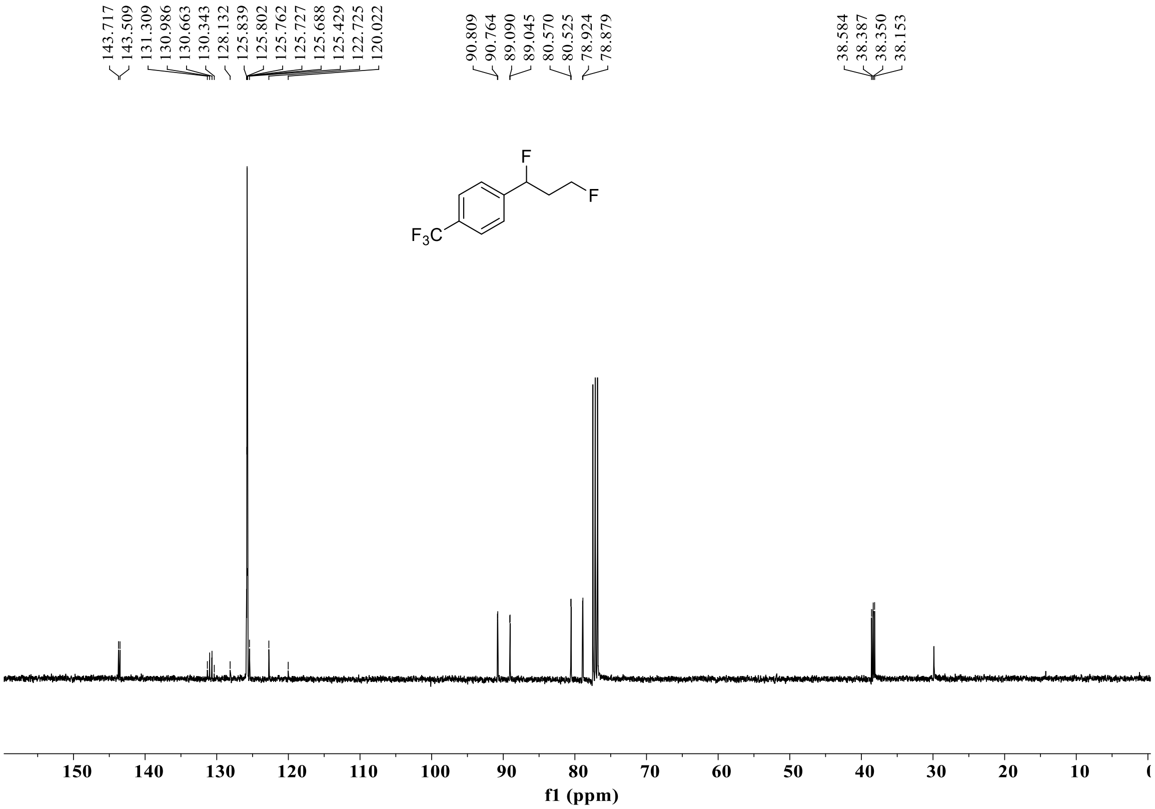

Supplementary Figure 55 ^{13}C NMR spectra of compound **21**

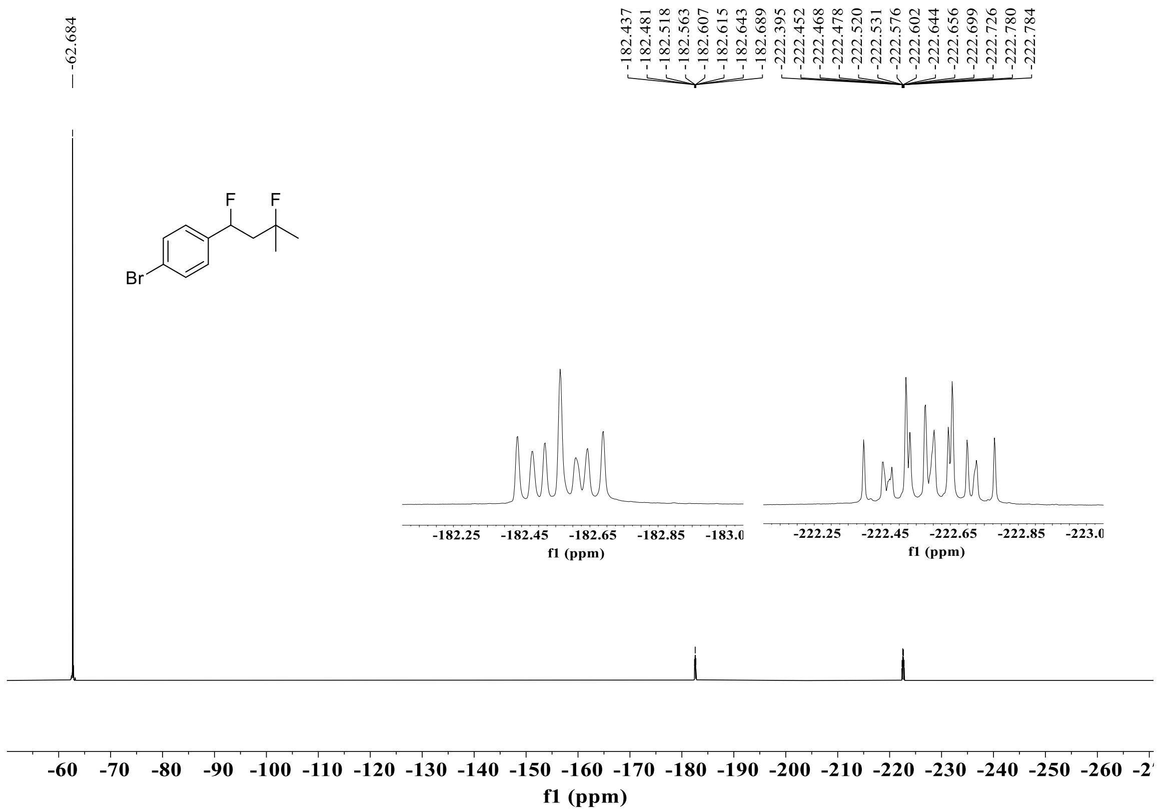

Supplementary Figure 56 ^{19}F NMR spectra of compound **21**

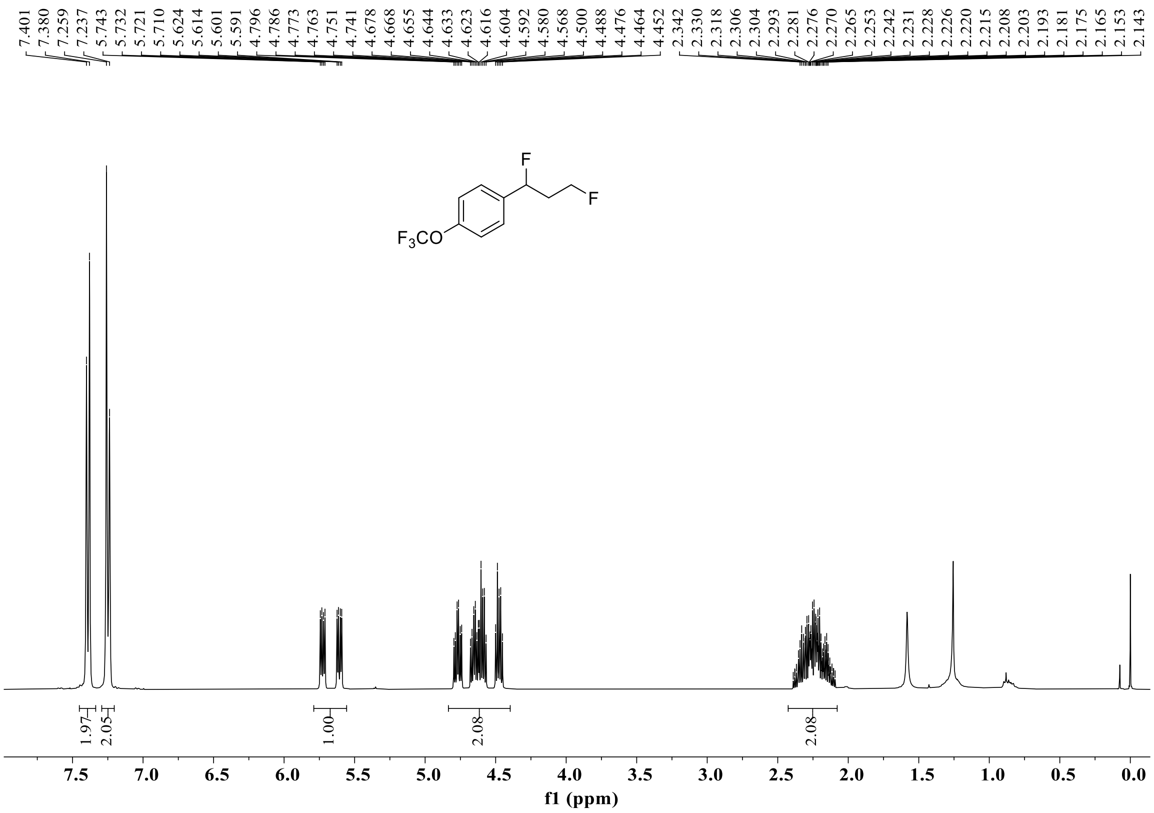

Supplementary Figure 57 ^{1}H NMR spectra of compound **22**

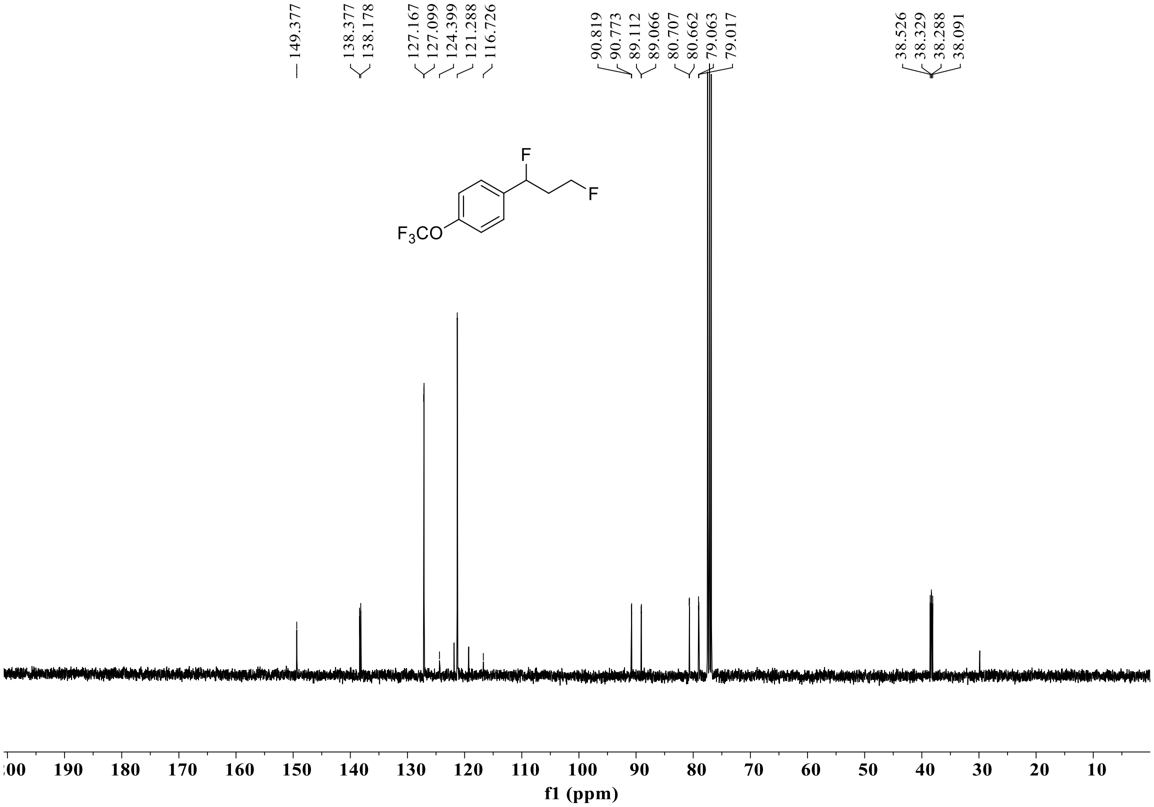

Supplementary Figure 58 ^{13}C NMR spectra of compound **22**

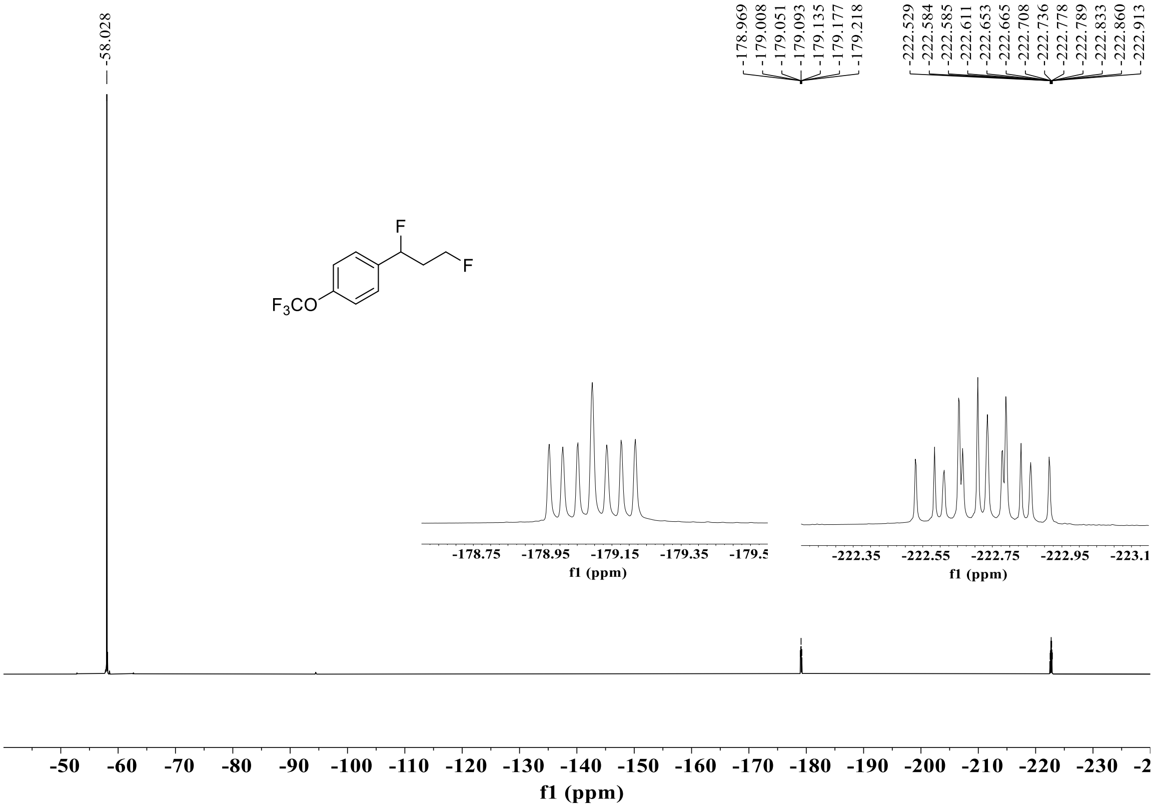

Supplementary Figure 59 ^{19}F NMR spectra of compound **22**

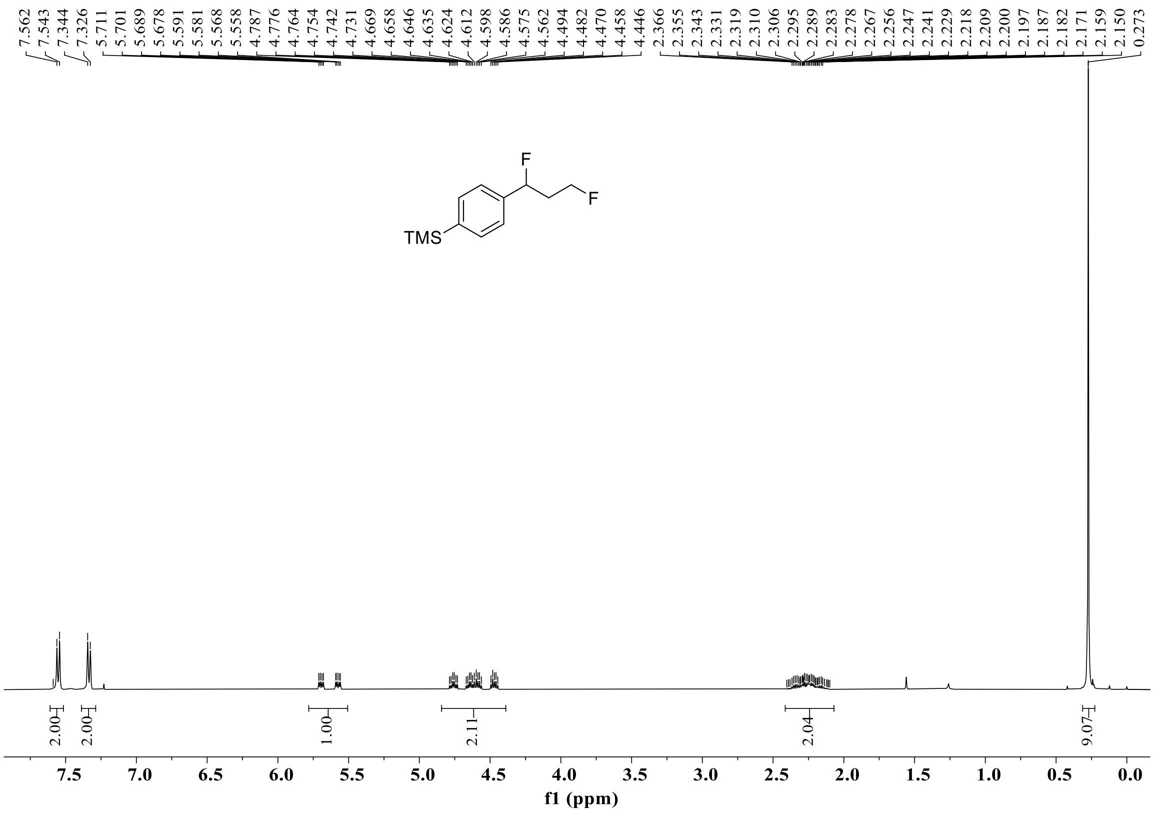

Supplementary Figure 60 ^{1}H NMR spectra of compound **23**

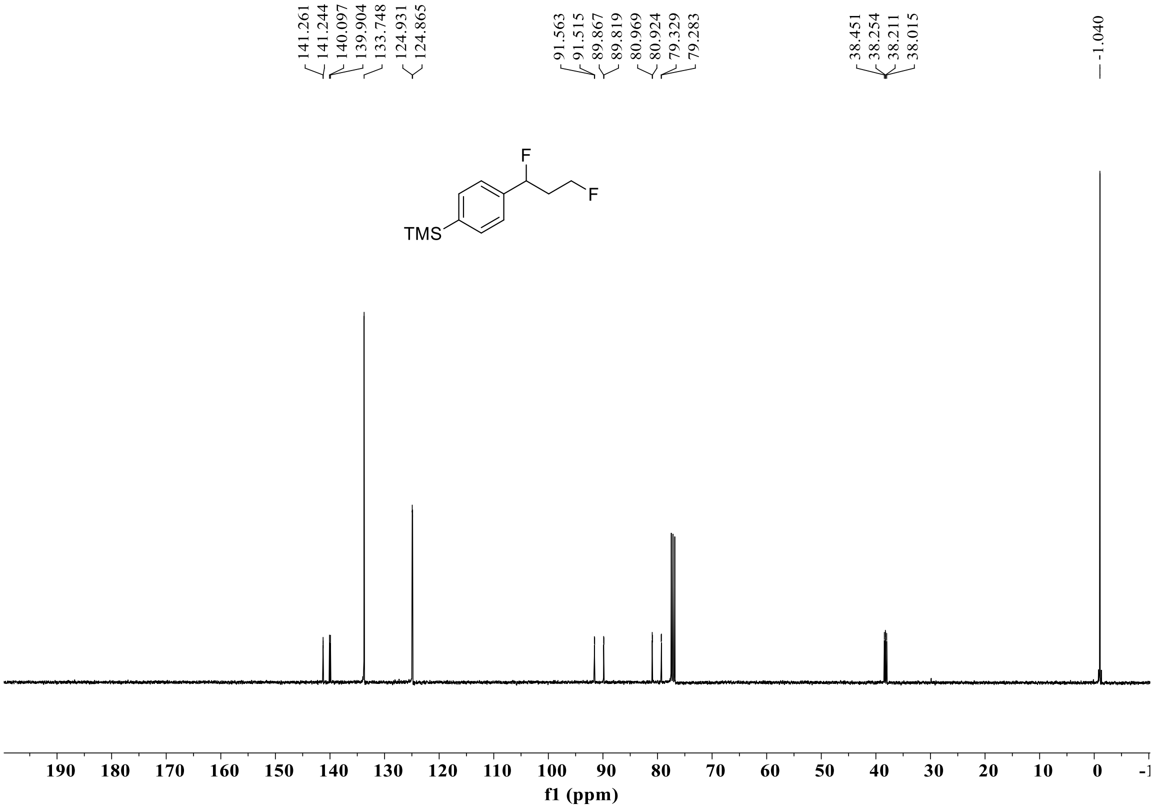

Supplementary Figure 61 ^{13}C NMR spectra of compound **23**

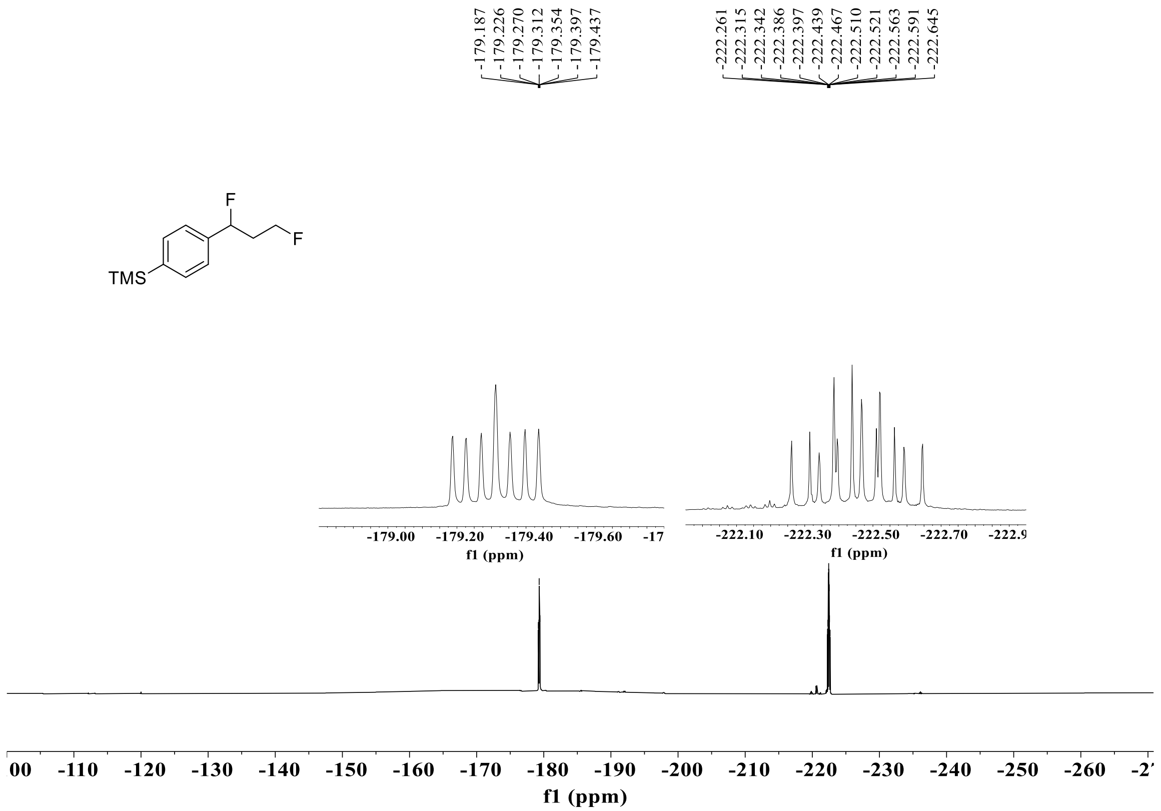

Supplementary Figure 62 ^{19}F NMR spectra of compound **23**

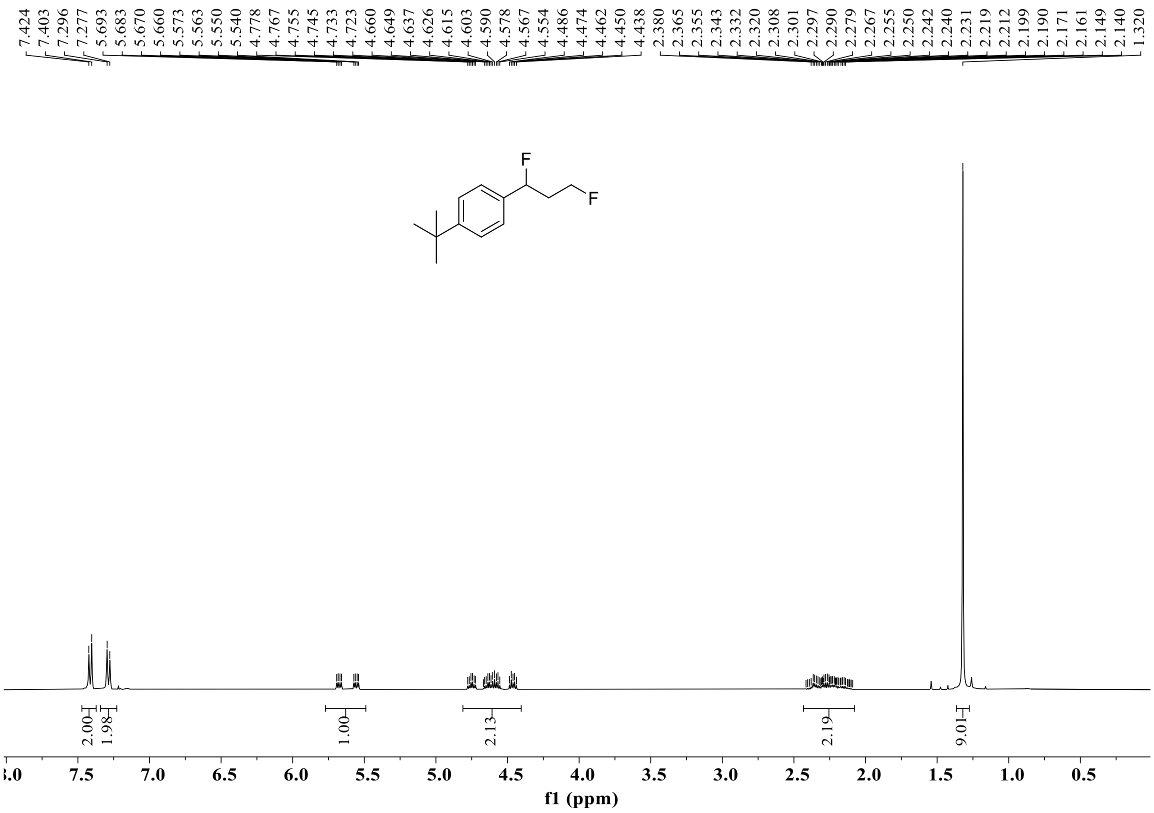

Supplementary Figure 63 ^{1}H NMR spectra of compound **24**

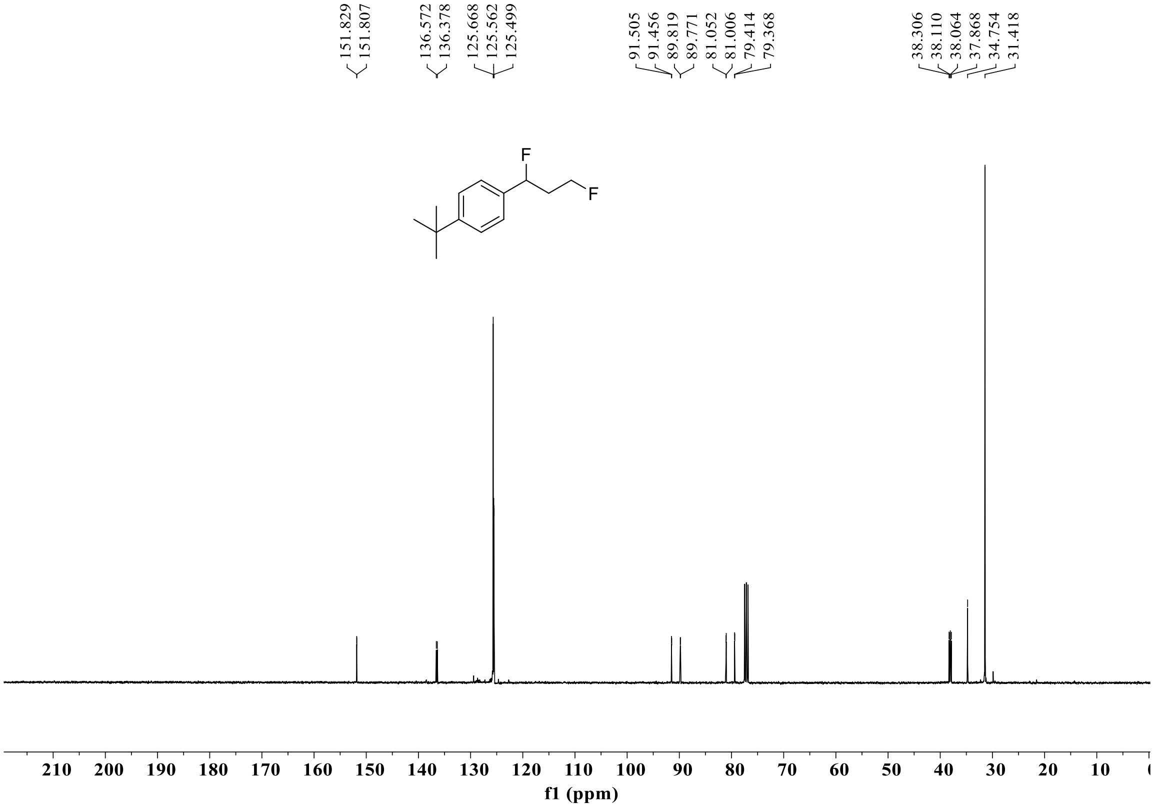

Supplementary Figure 64 ^{13}C NMR spectra of compound **24**

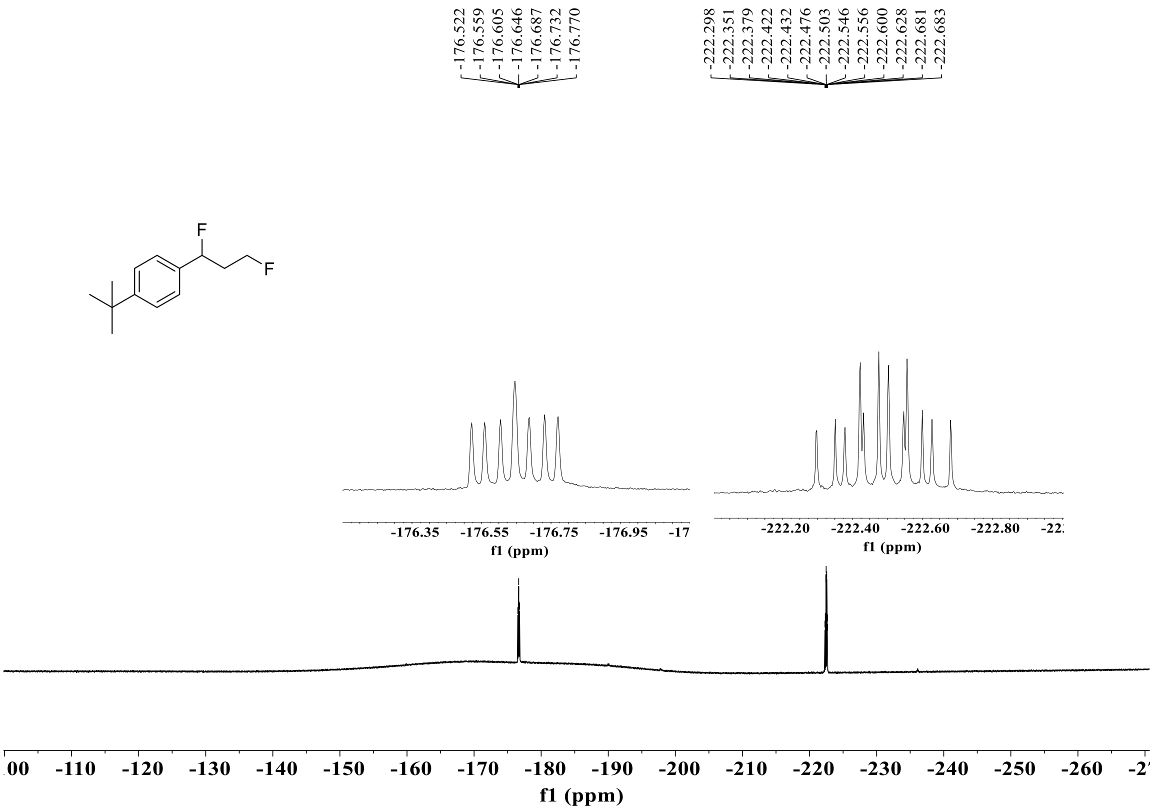

Supplementary Figure 65 ^{19}F NMR spectra of compound **24**

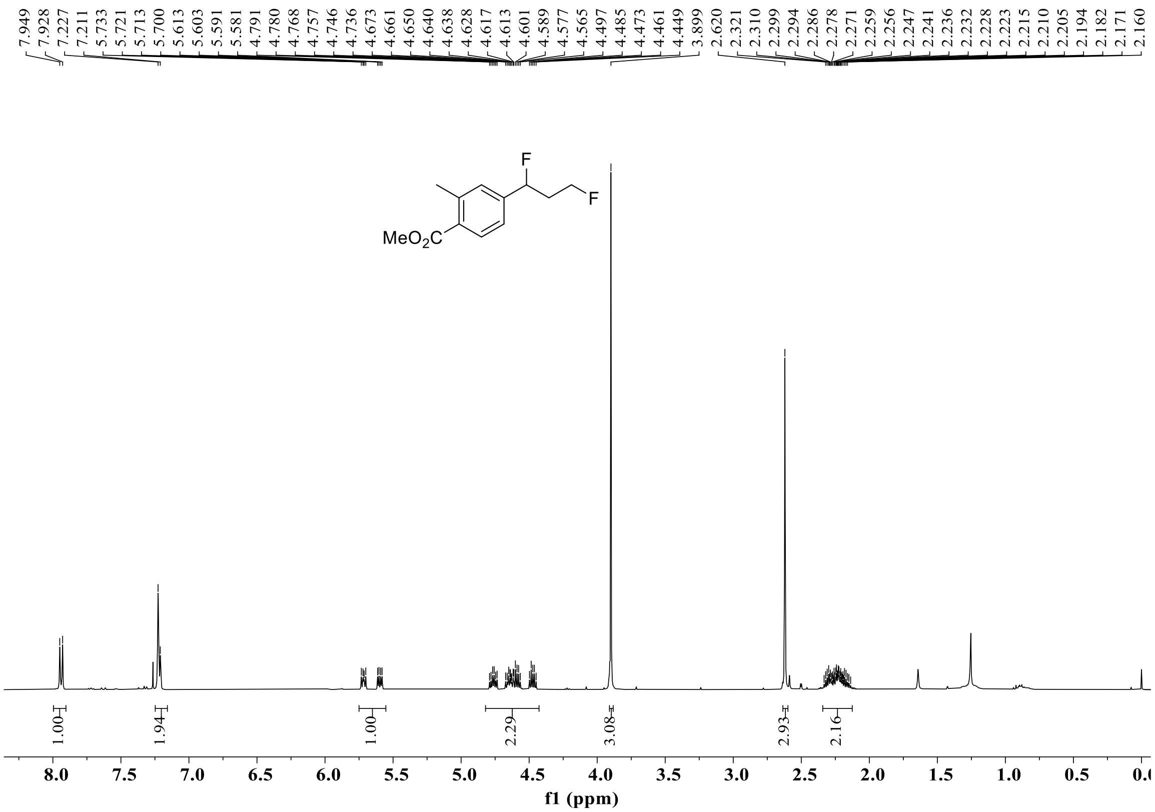

Supplementary Figure 66 ^{1}H NMR spectra of compound **25**

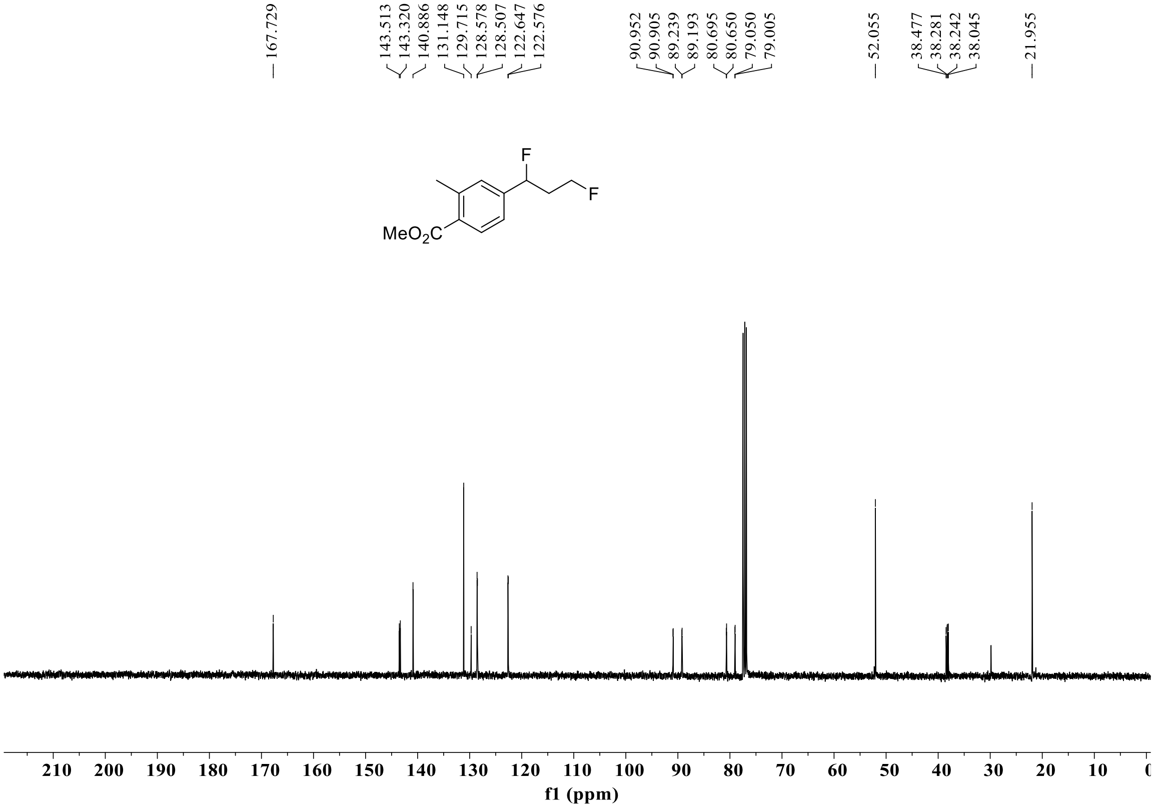

Supplementary Figure 67 ^{13}C NMR spectra of compound **25**

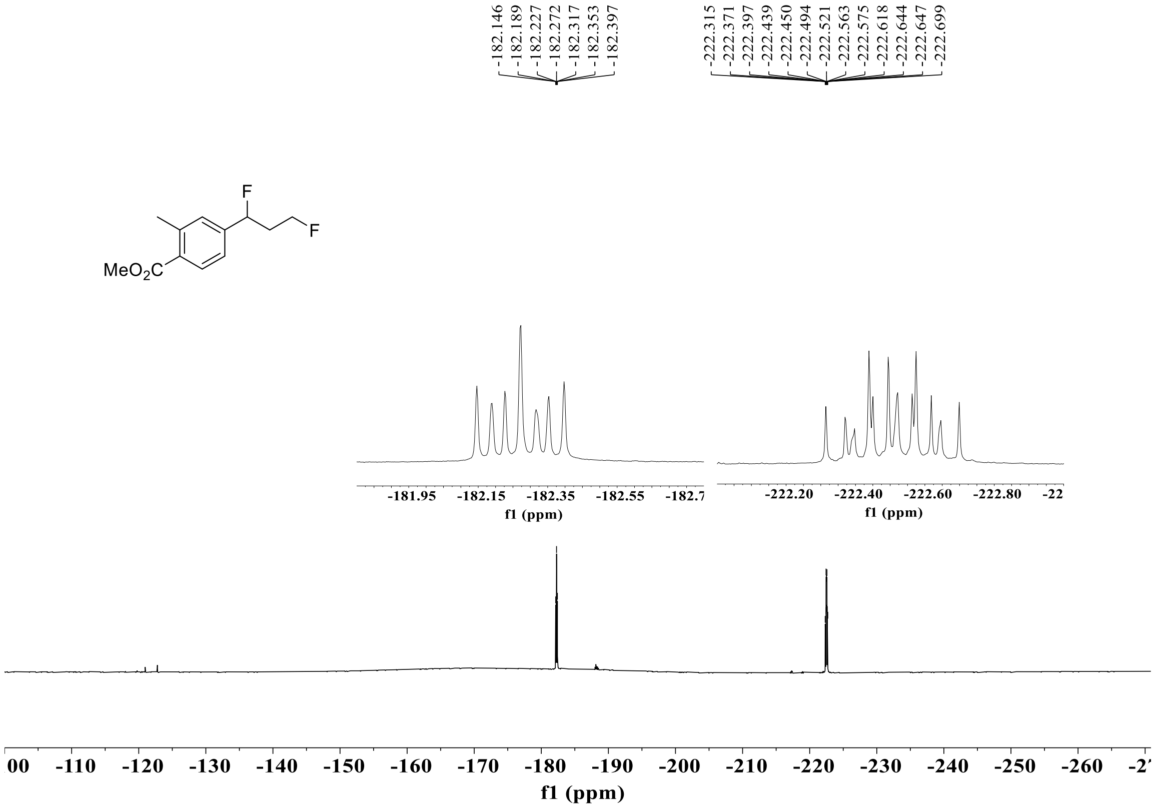

Supplementary Figure 68 ^{19}F NMR spectra of compound **25**

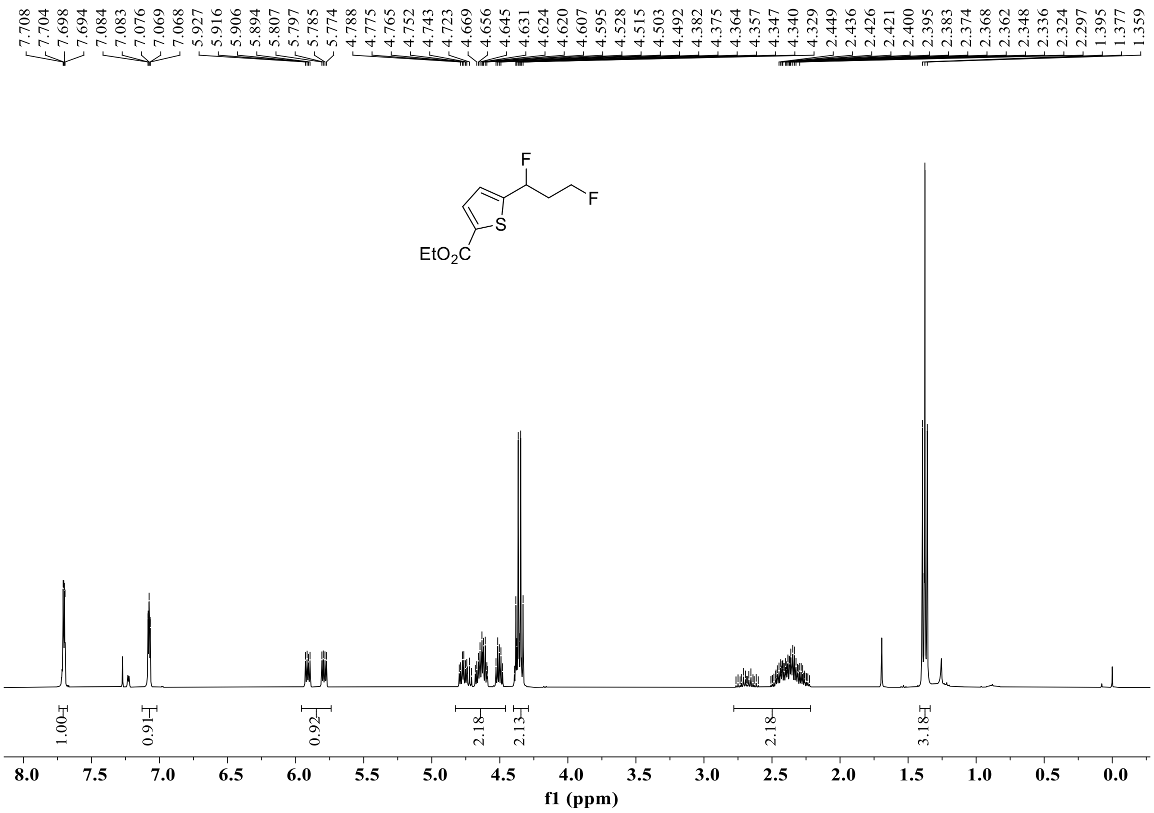

Supplementary Figure 69 ^{19}F NMR spectra of compound **26**

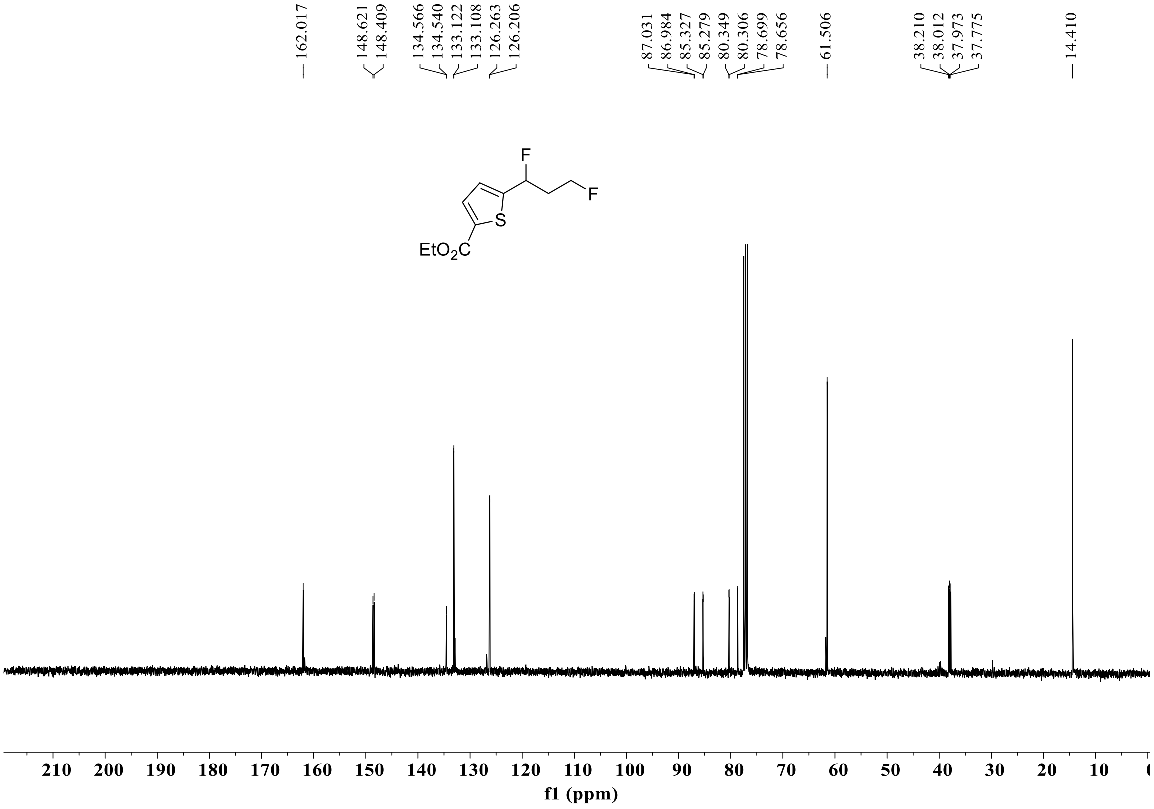

Supplementary Figure 70 ^{13}C NMR spectra of compound **26**

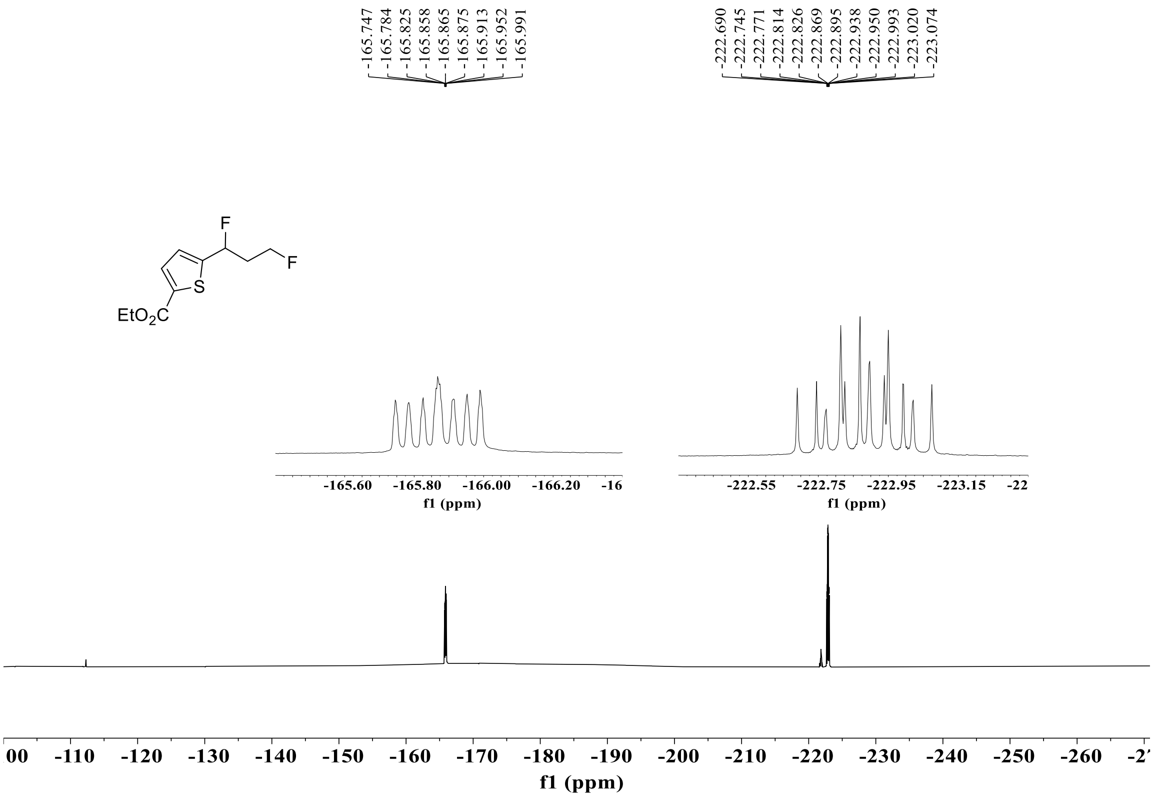

Supplementary Figure 71 ^{19}F NMR spectra of compound **26**

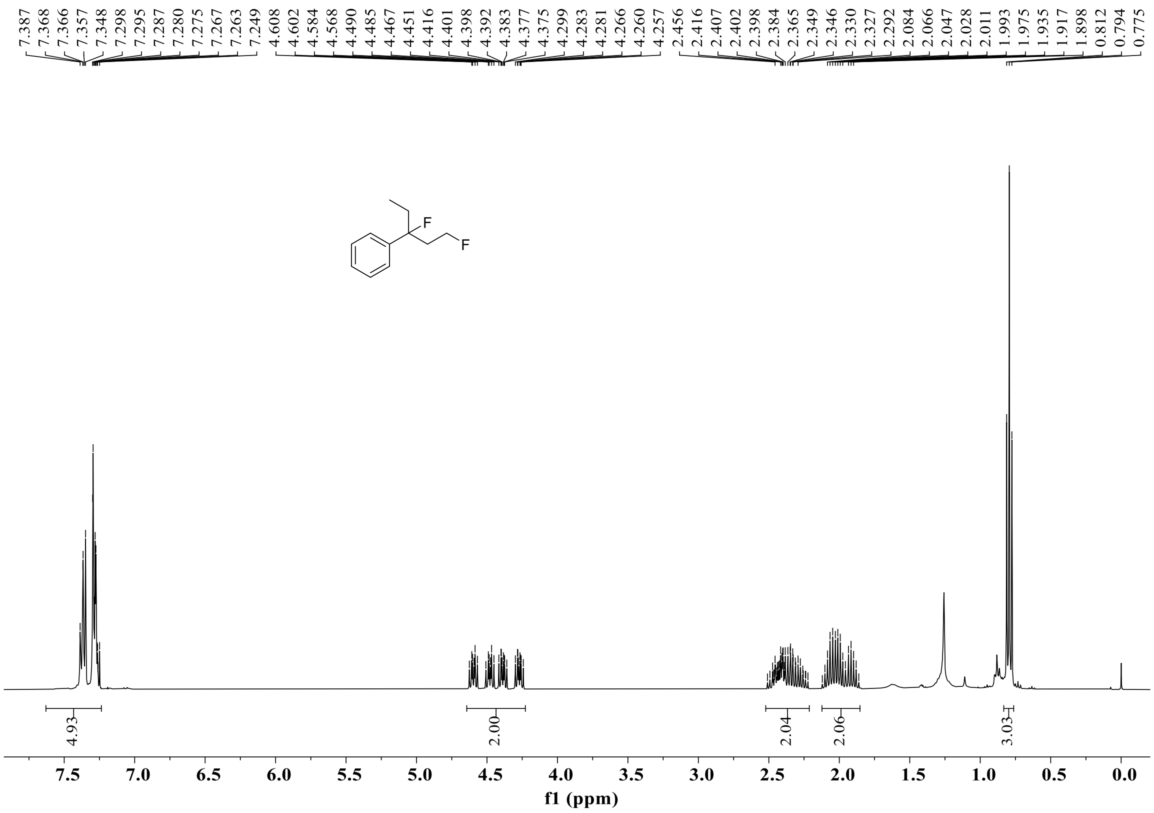

Supplementary Figure 72 ^{1}H NMR spectra of compound **27**

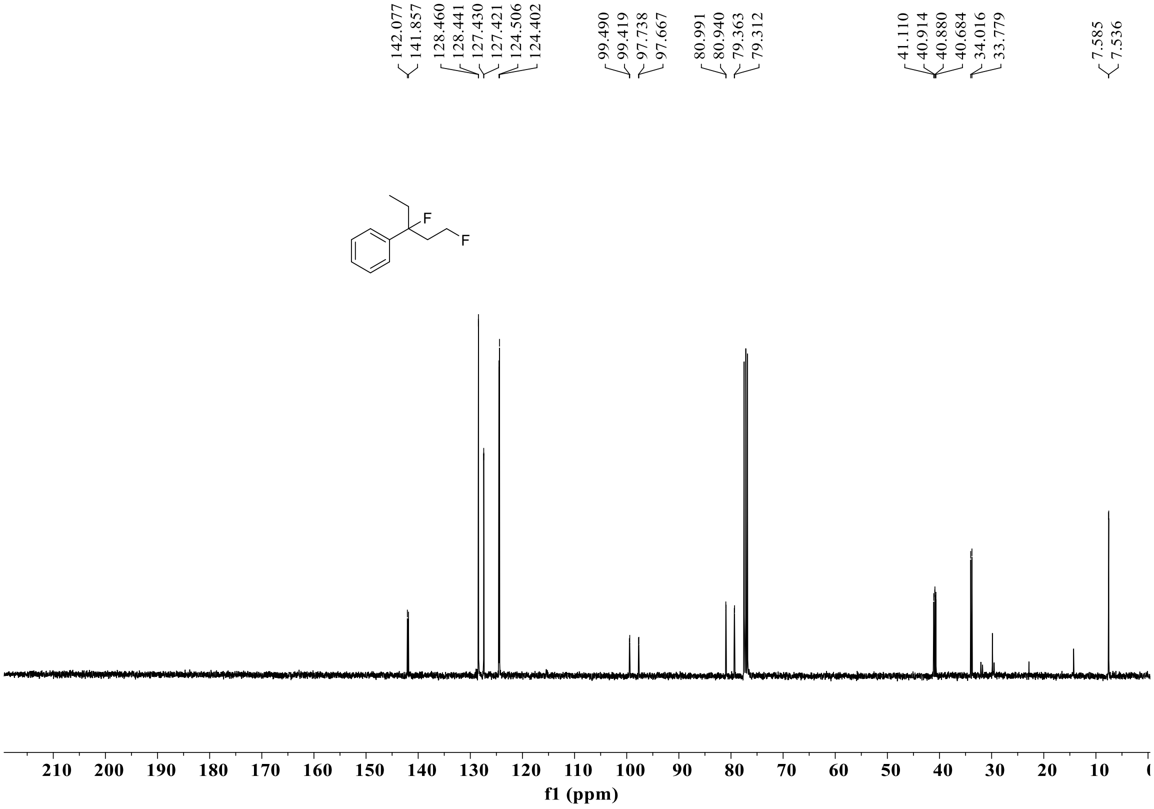

Supplementary Figure 73 ^{13}C NMR spectra of compound **27**

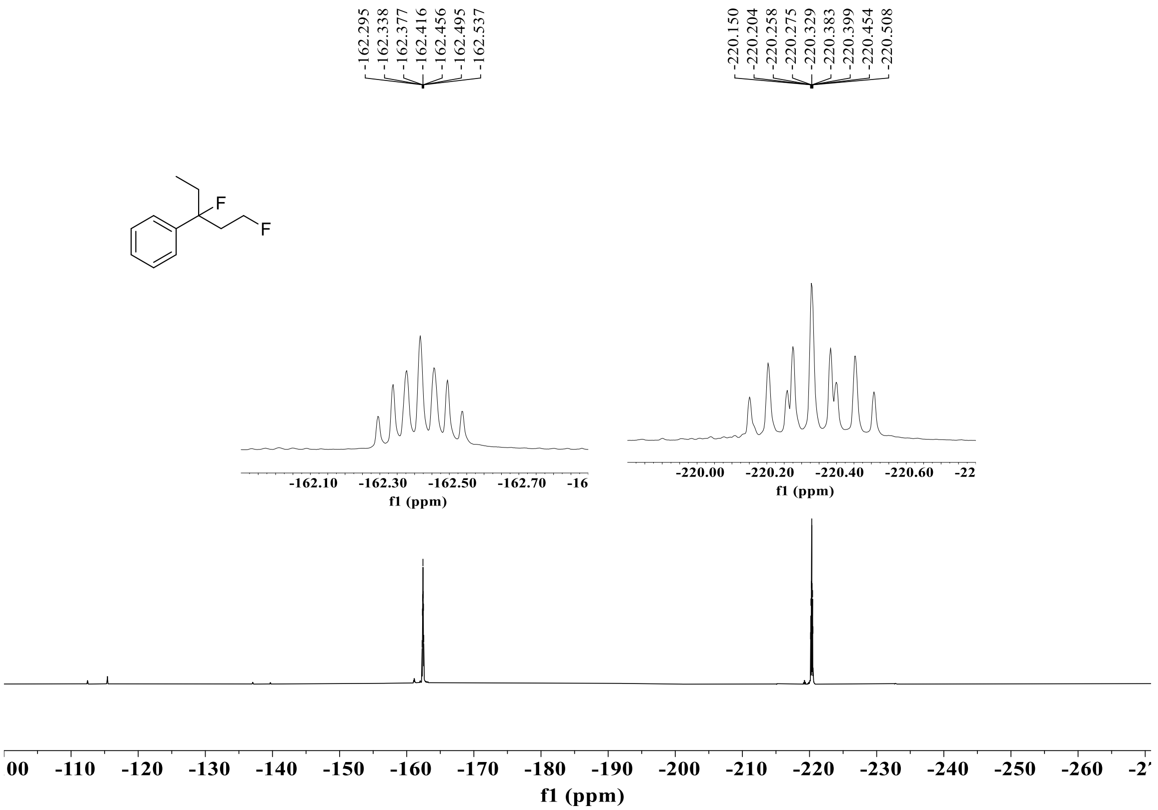

Supplementary Figure 74 ^{19}F NMR spectra of compound **27**

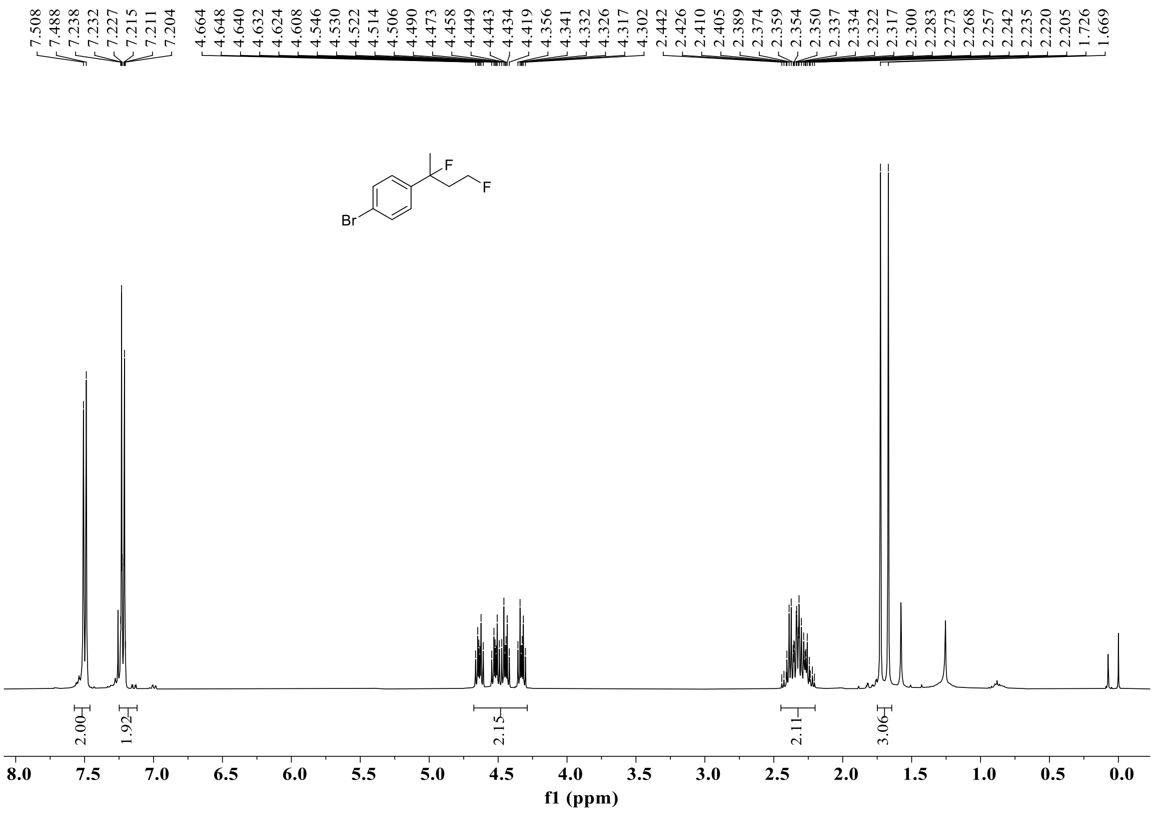

Supplementary Figure 75 ^{1}H NMR spectra of compound **28**

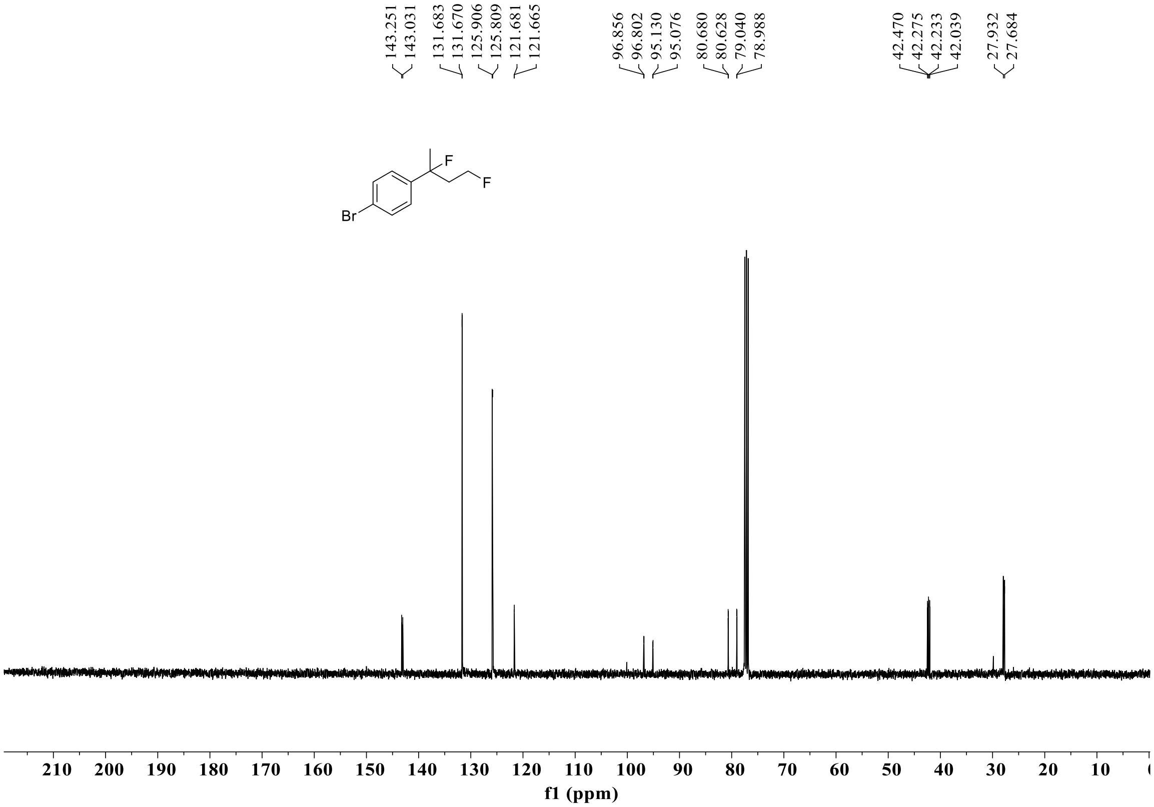

Supplementary Figure 76 ^{1}H NMR spectra of compound **28**

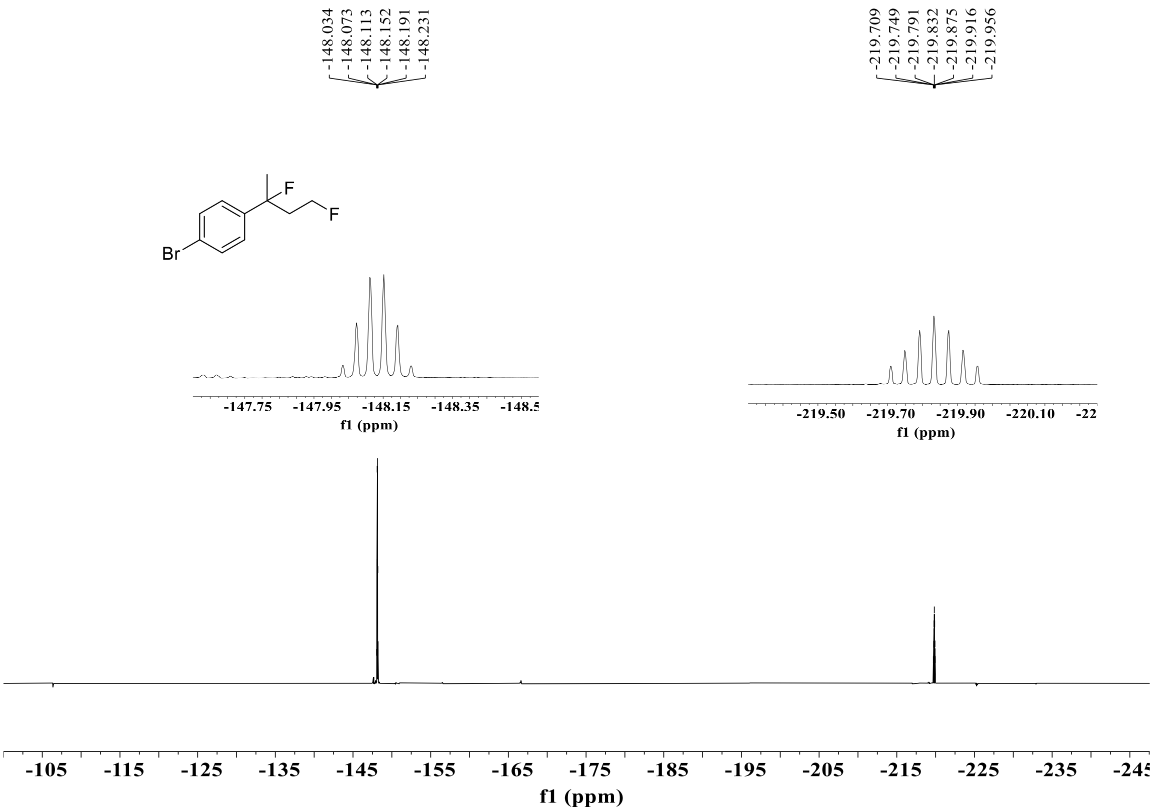

Supplementary Figure 77 ^{1}H NMR spectra of compound **28**

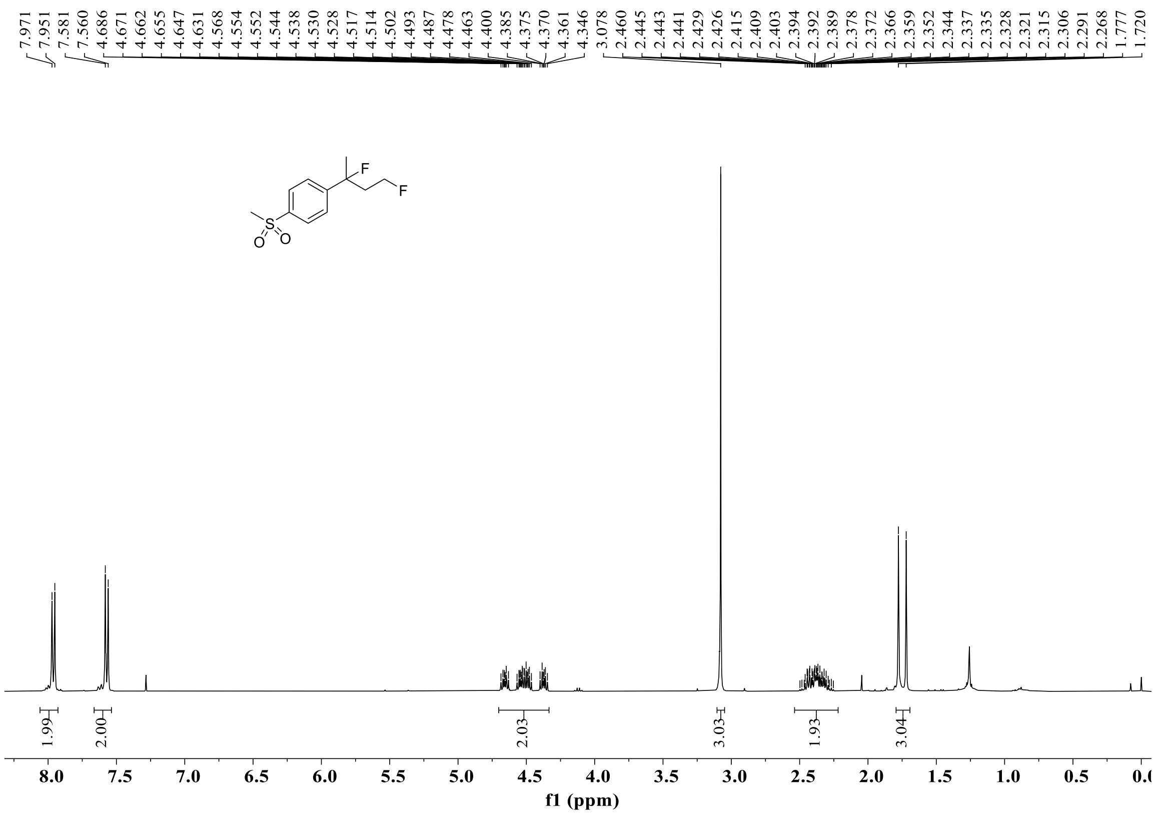

Supplementary Figure 78 ^{1}H NMR spectra of compound **29**

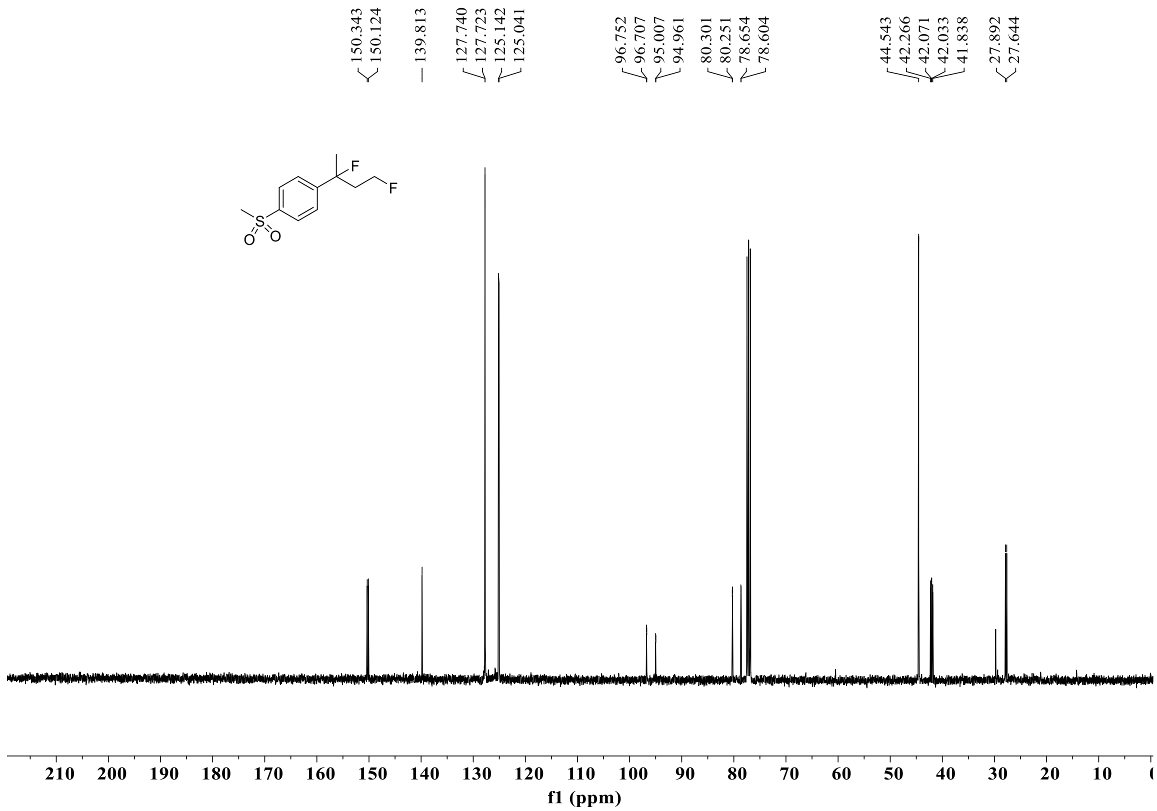

Supplementary Figure 79 ^{13}C NMR spectra of compound **29**

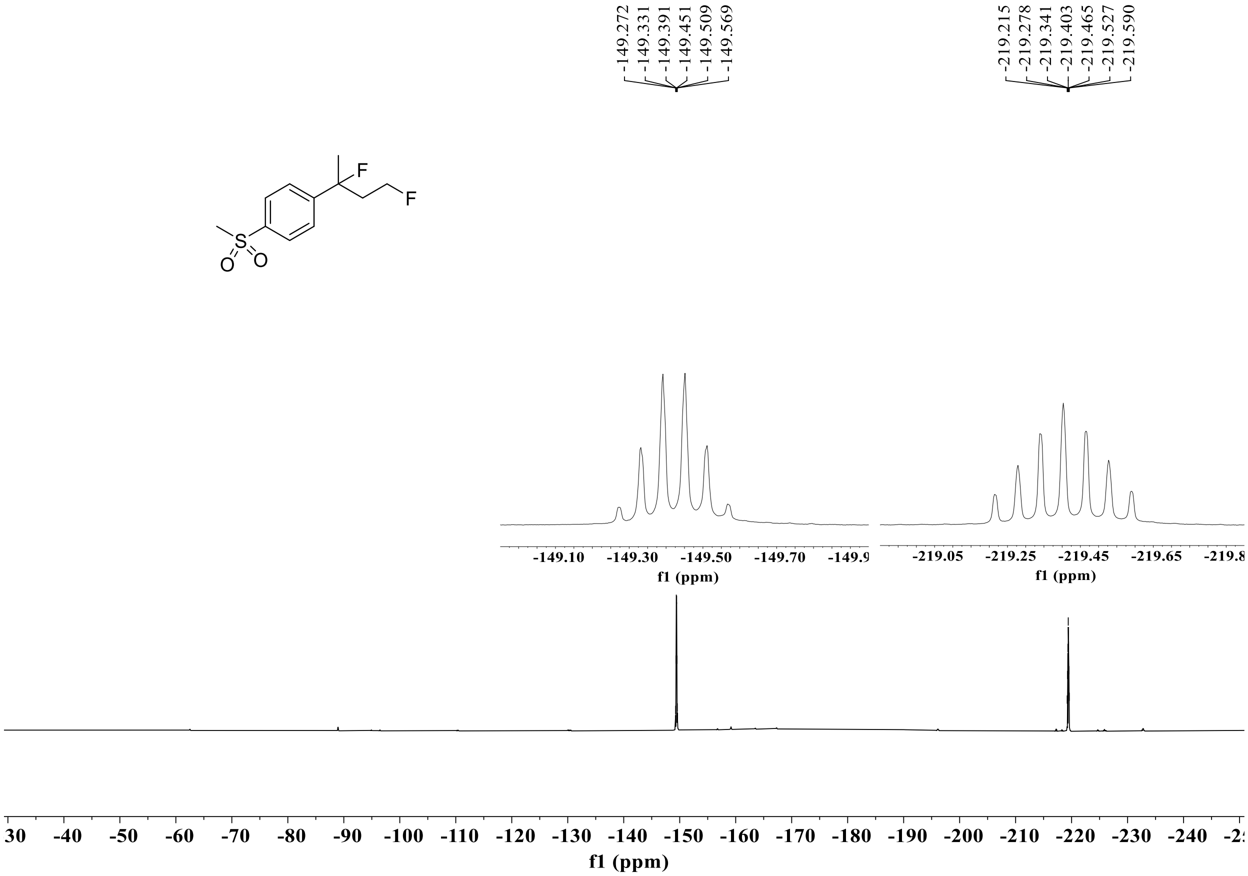

Supplementary Figure 80 ^{19}F NMR spectra of compound **29**

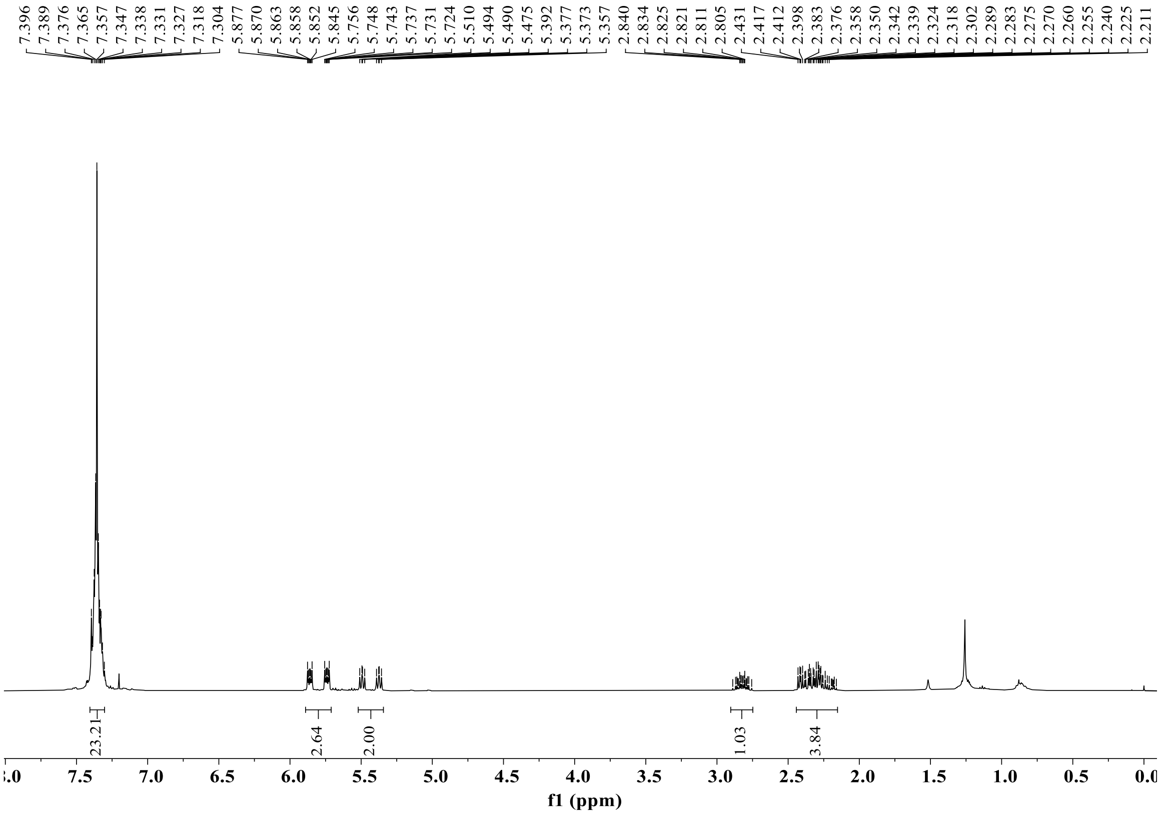

Supplementary Figure 81 ^{1}H NMR spectra of compound **30**

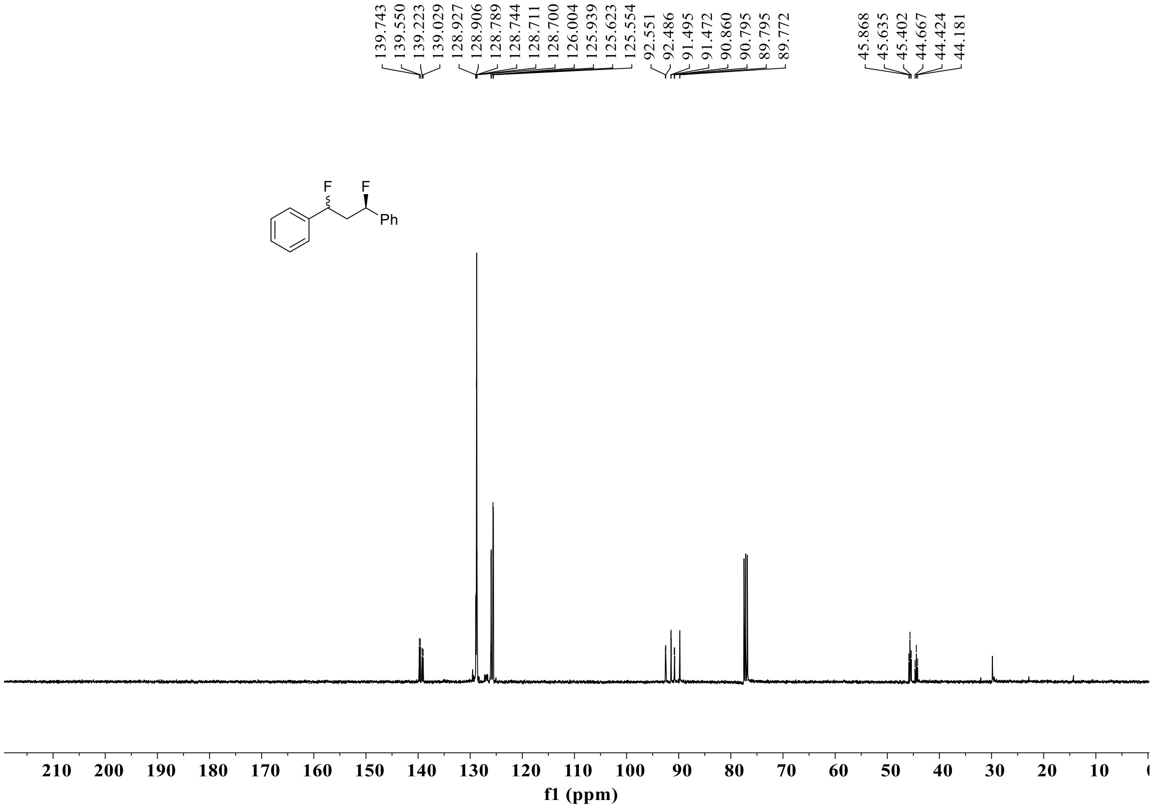

Supplementary Figure 82 ^{13}C NMR spectra of compound **30**

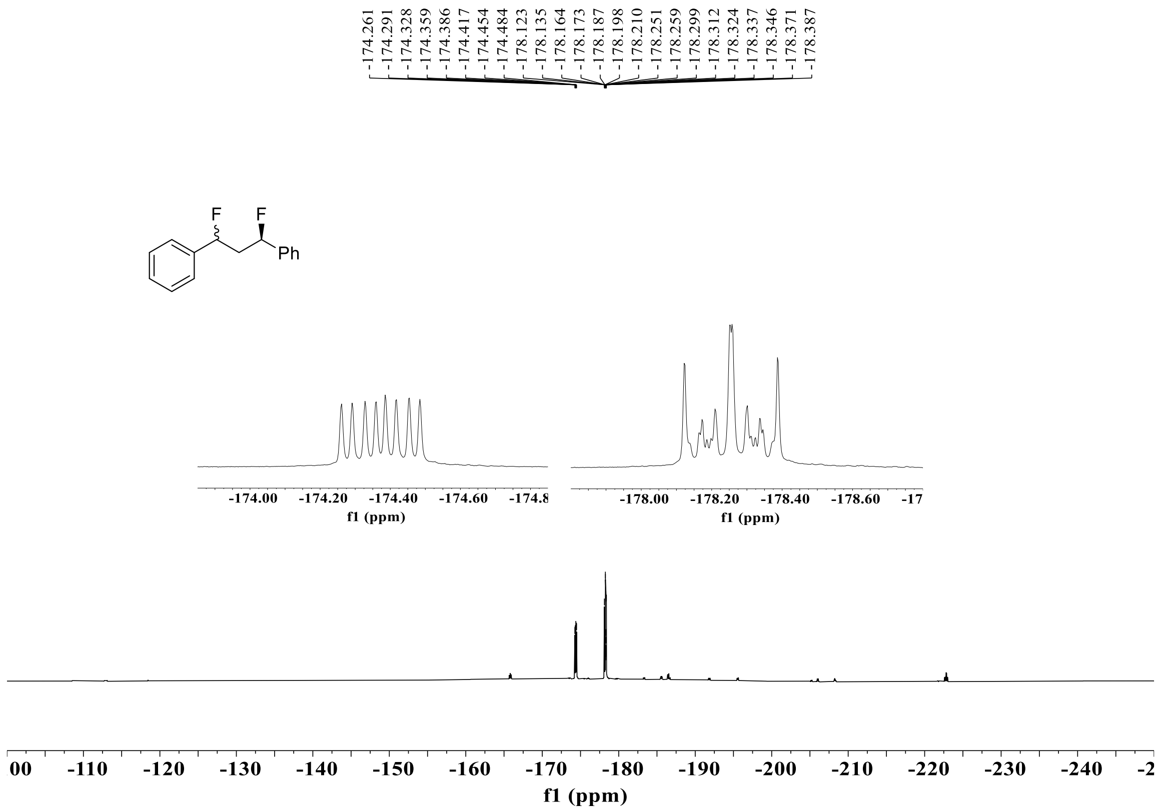

Supplementary Figure 83 ^{19}F NMR spectra of compound **30**

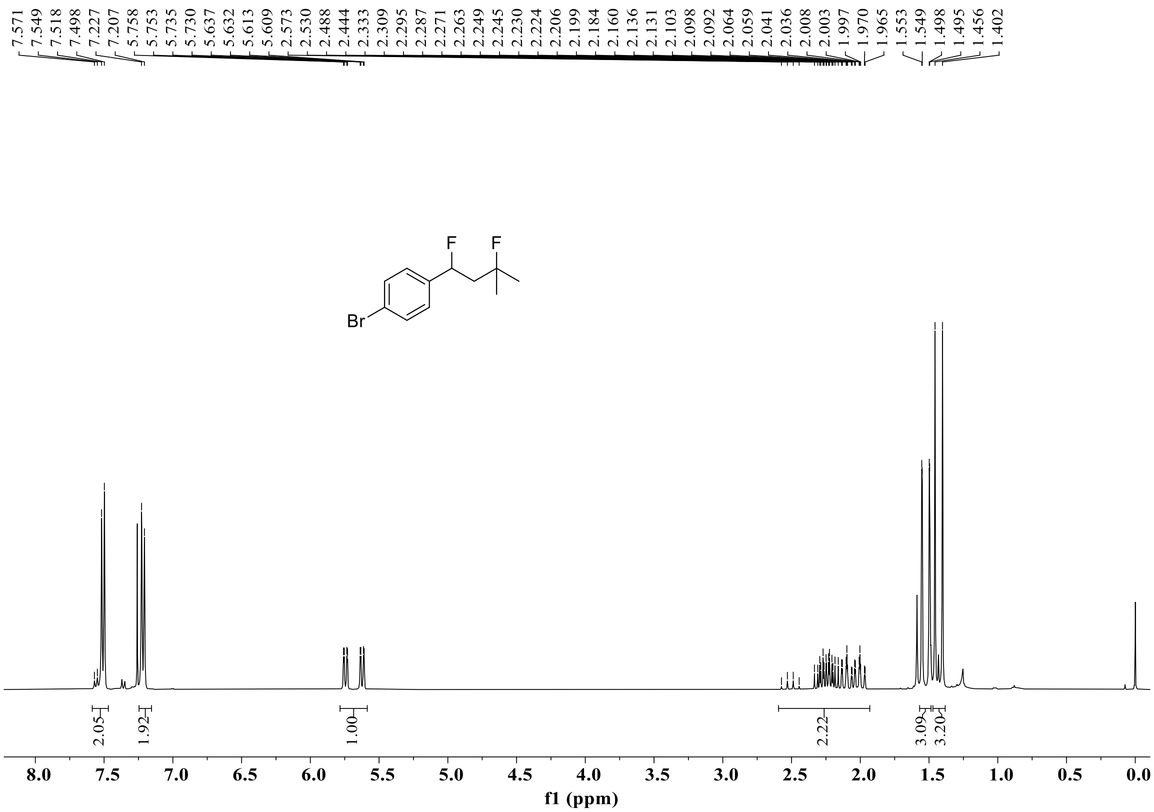

Supplementary Figure 84 ^{1}H NMR spectra of compound **31**

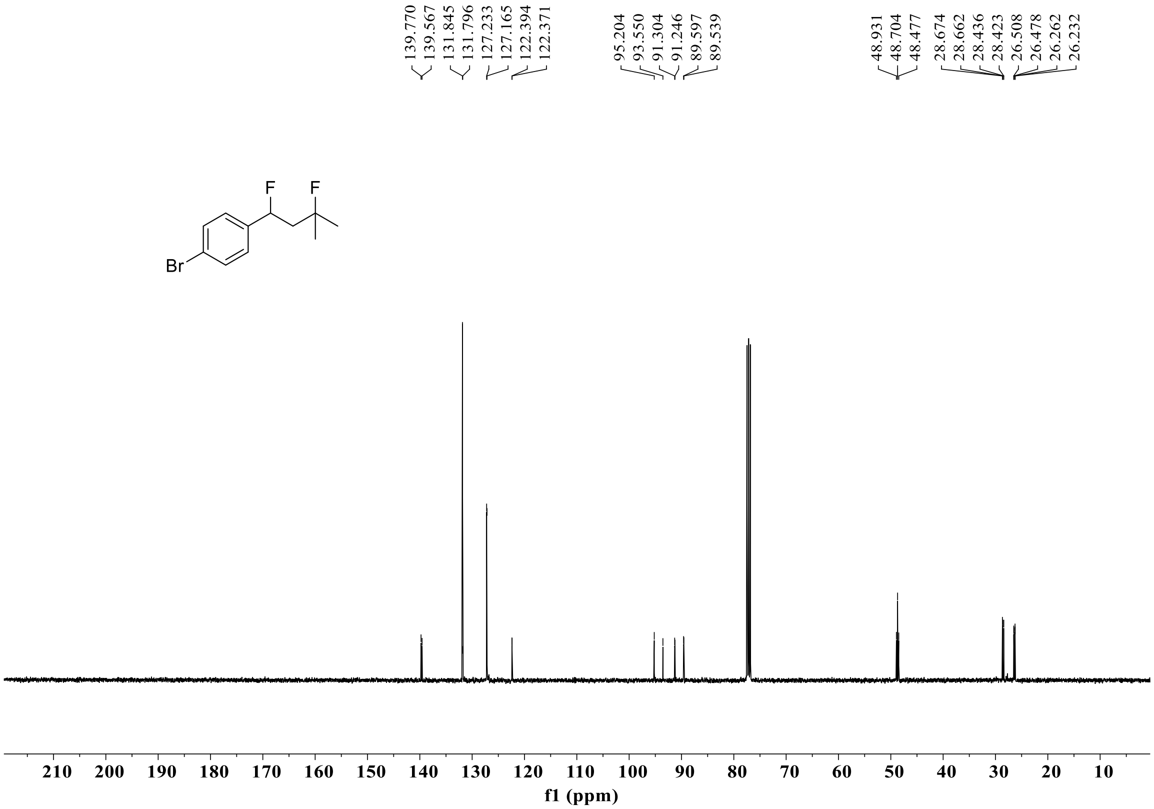

Supplementary Figure 85 ^{13}C NMR spectra of compound **31**

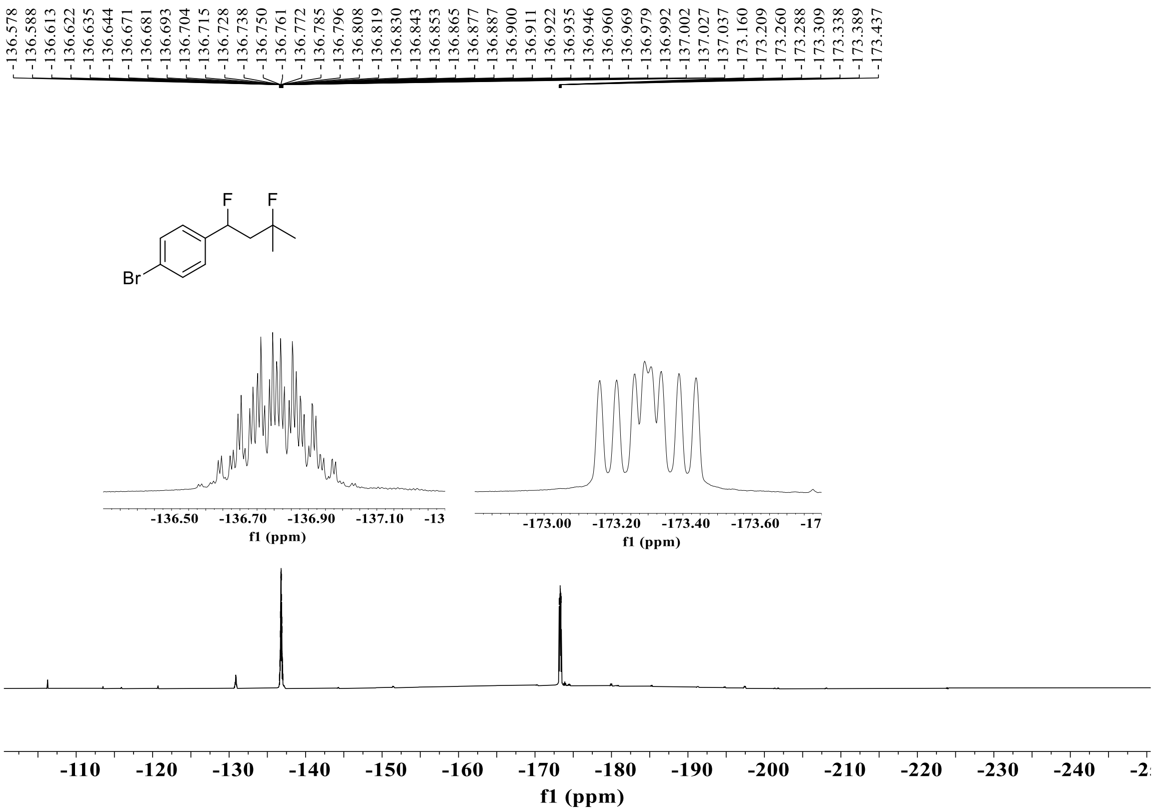

Supplementary Figure 86 ^{19}F NMR spectra of compound **31**

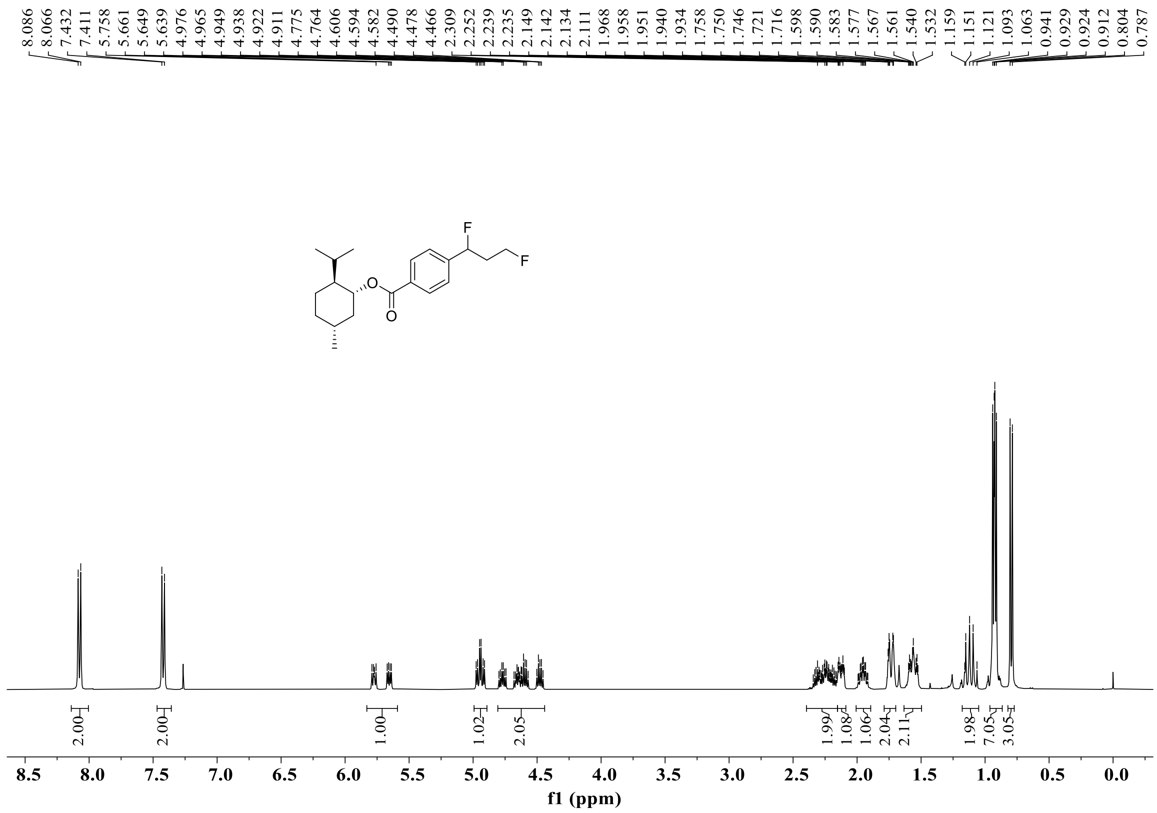

Supplementary Figure 87 ^{1}H NMR spectra of compound **32**

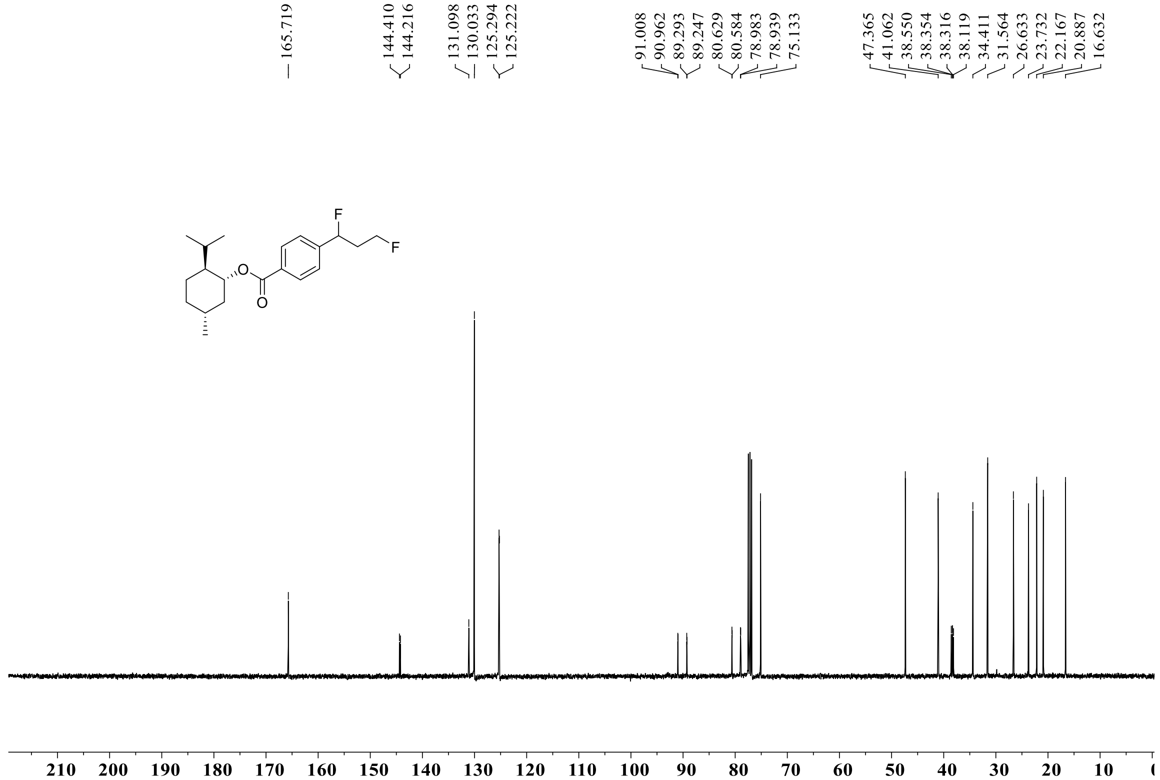

Supplementary Figure 88 ^{13}C NMR spectra of compound **32**

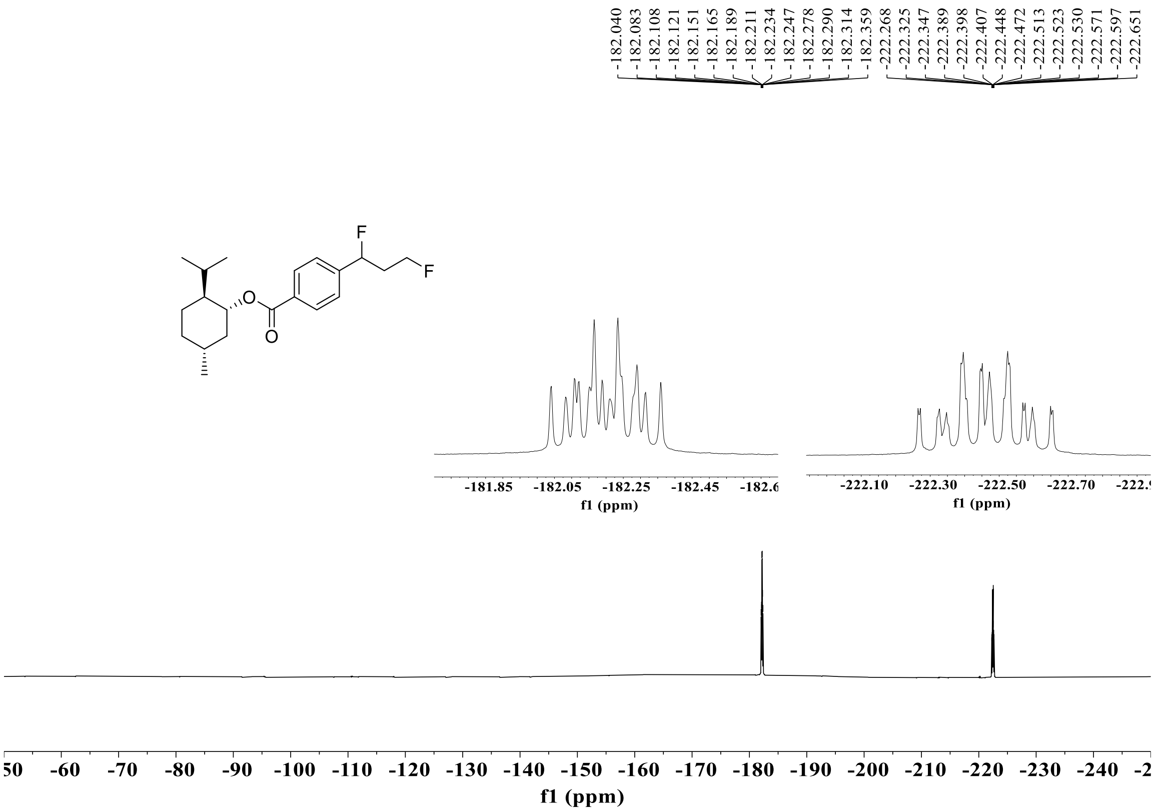

Supplementary Figure 89 ^{19}F NMR spectra of compound **32**

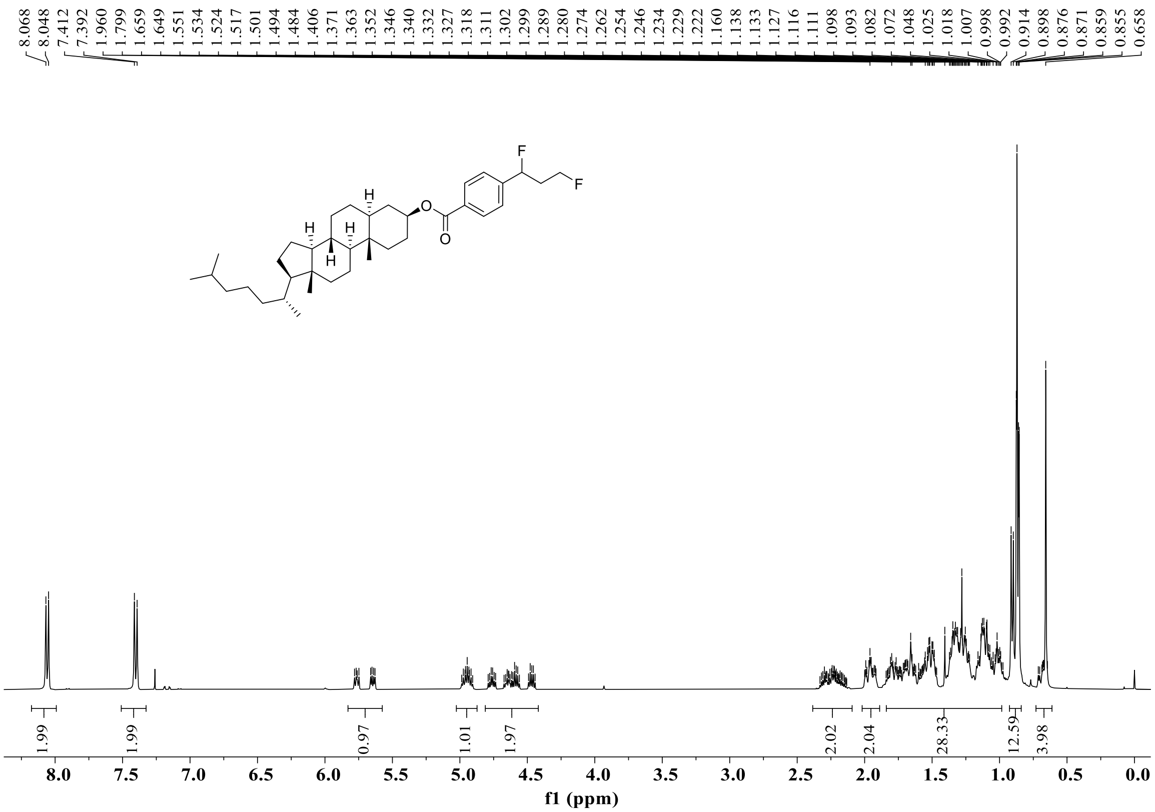

Supplementary Figure 90 ^{1}H NMR spectra of compound **33**

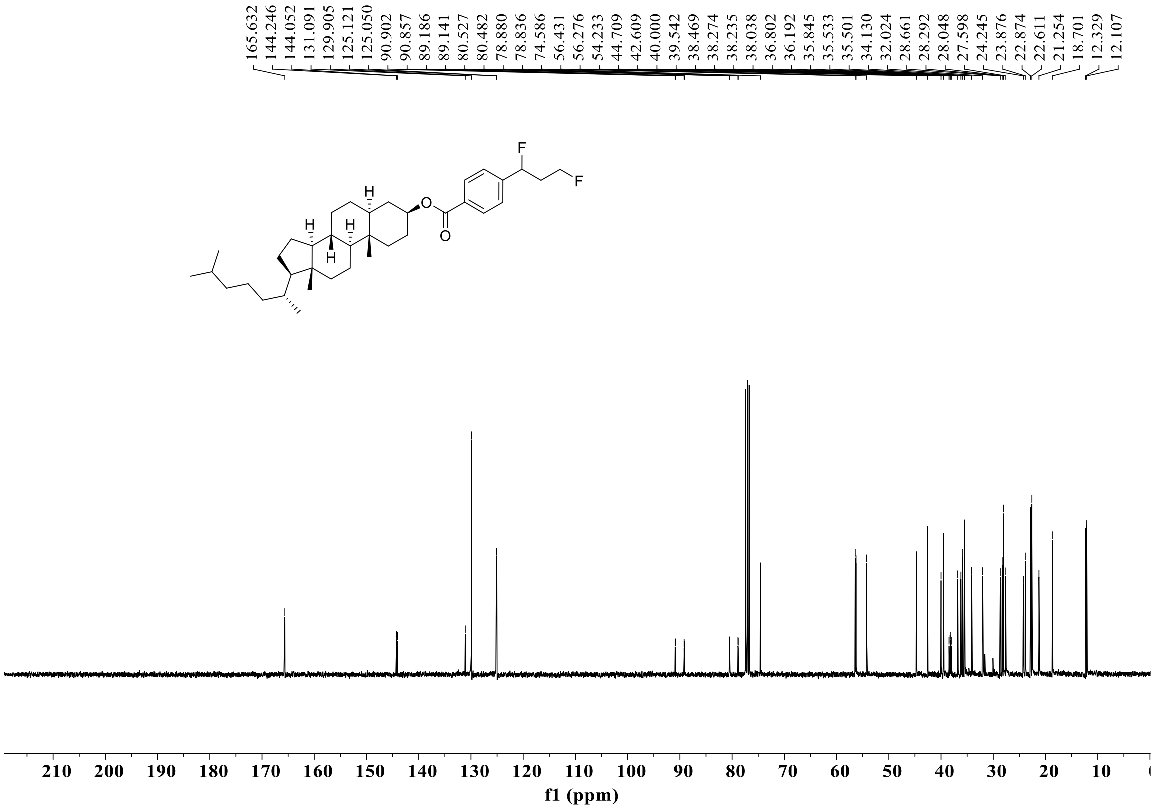

Supplementary Figure 91 ^{13}C NMR spectra of compound **33**

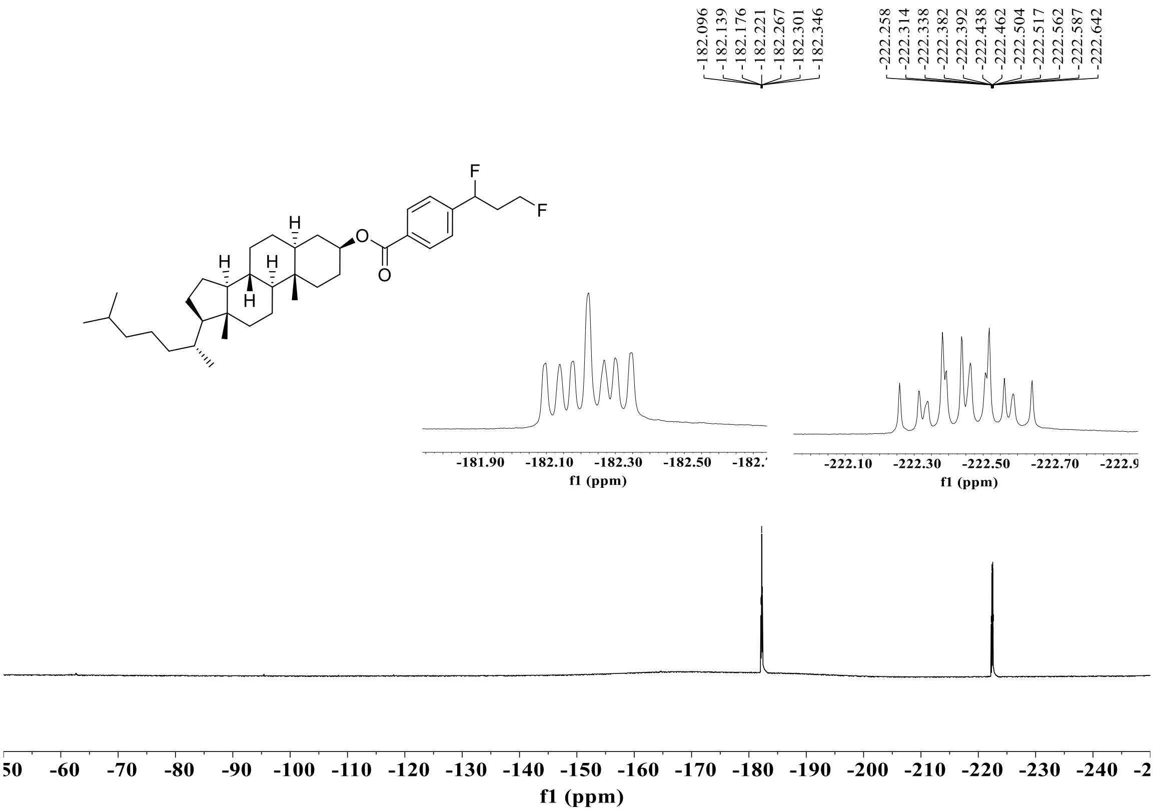

Supplementary Figure 92 ^{19}F NMR spectra of compound **33**

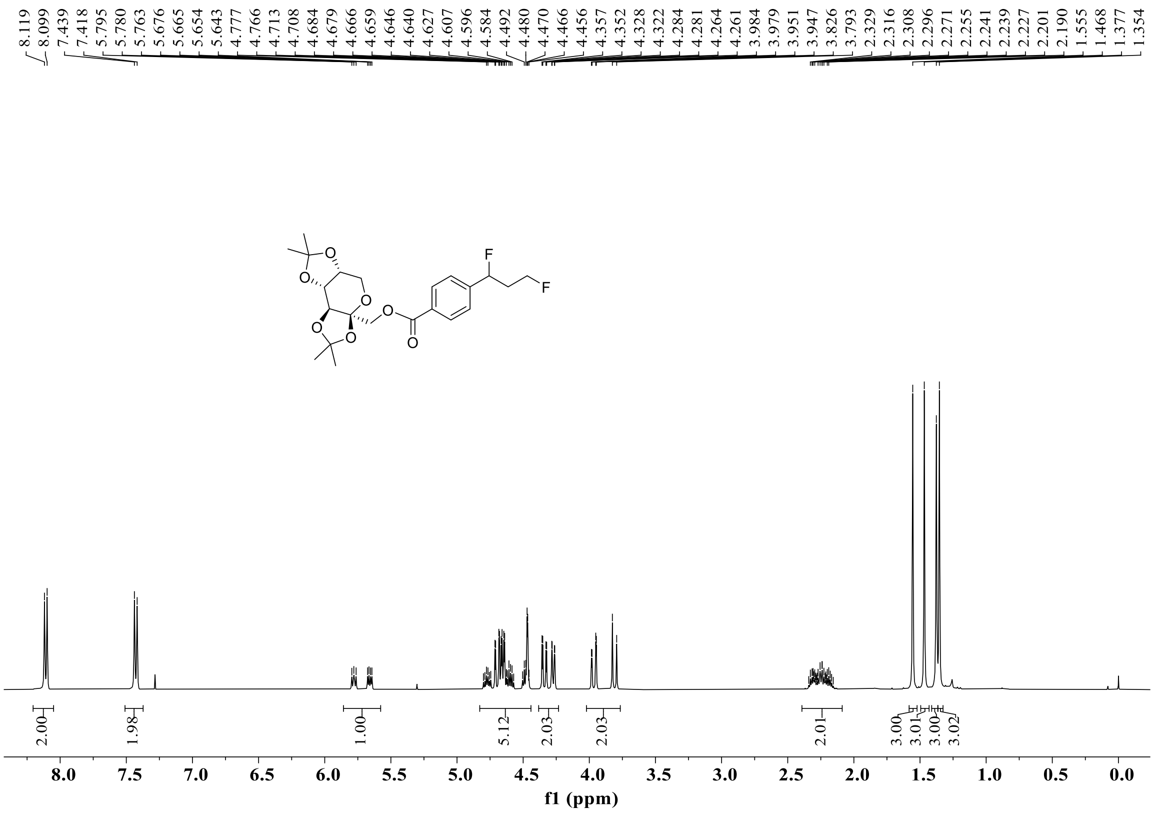

Supplementary Figure 93 ^{1}H NMR spectra of compound **34**

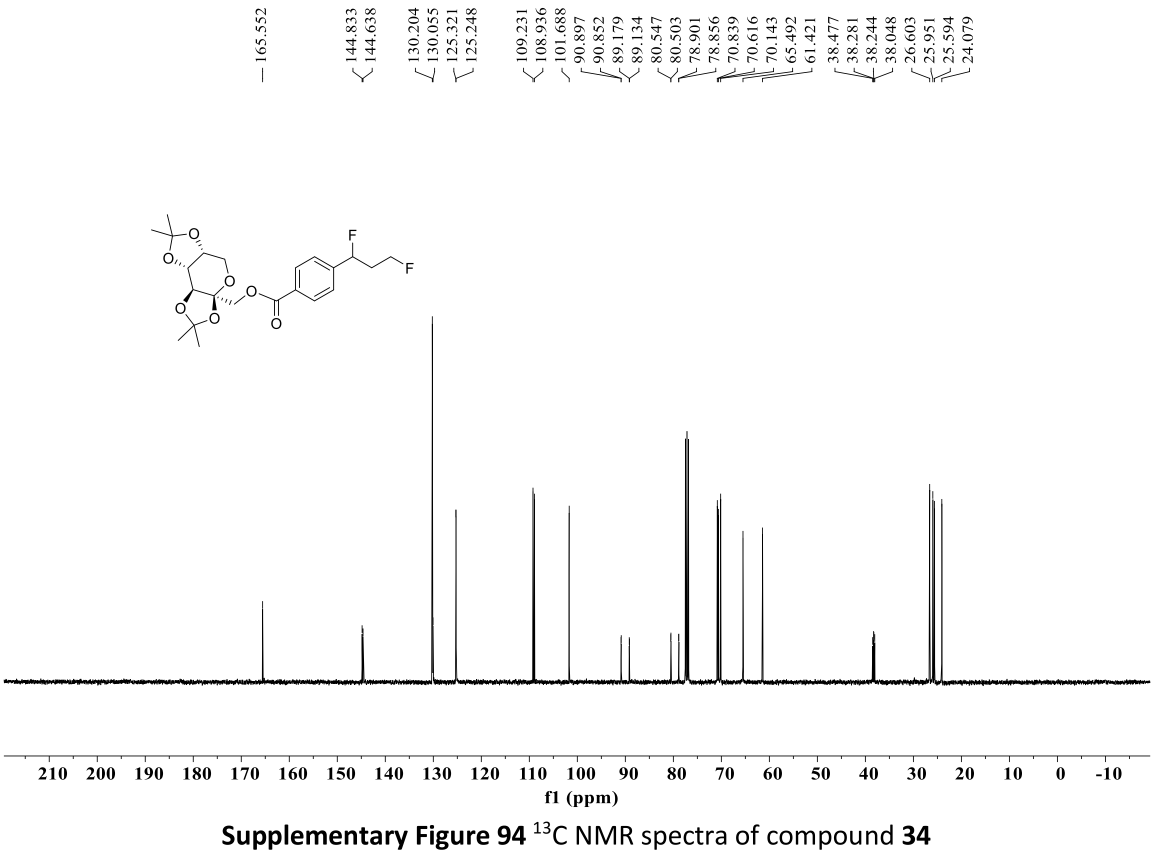

Supplementary Figure 94 ^{13}C NMR spectra of compound **34**

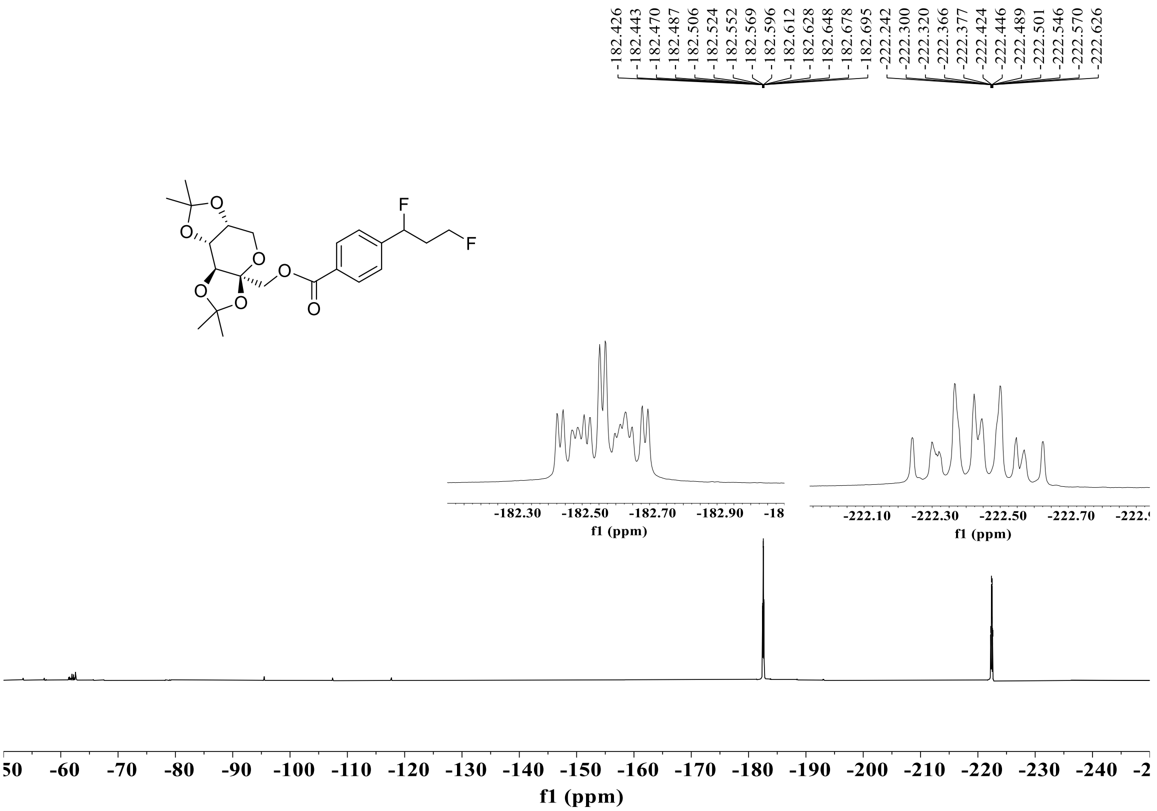

Supplementary Figure 95 ^{19}F NMR spectra of compound **34**

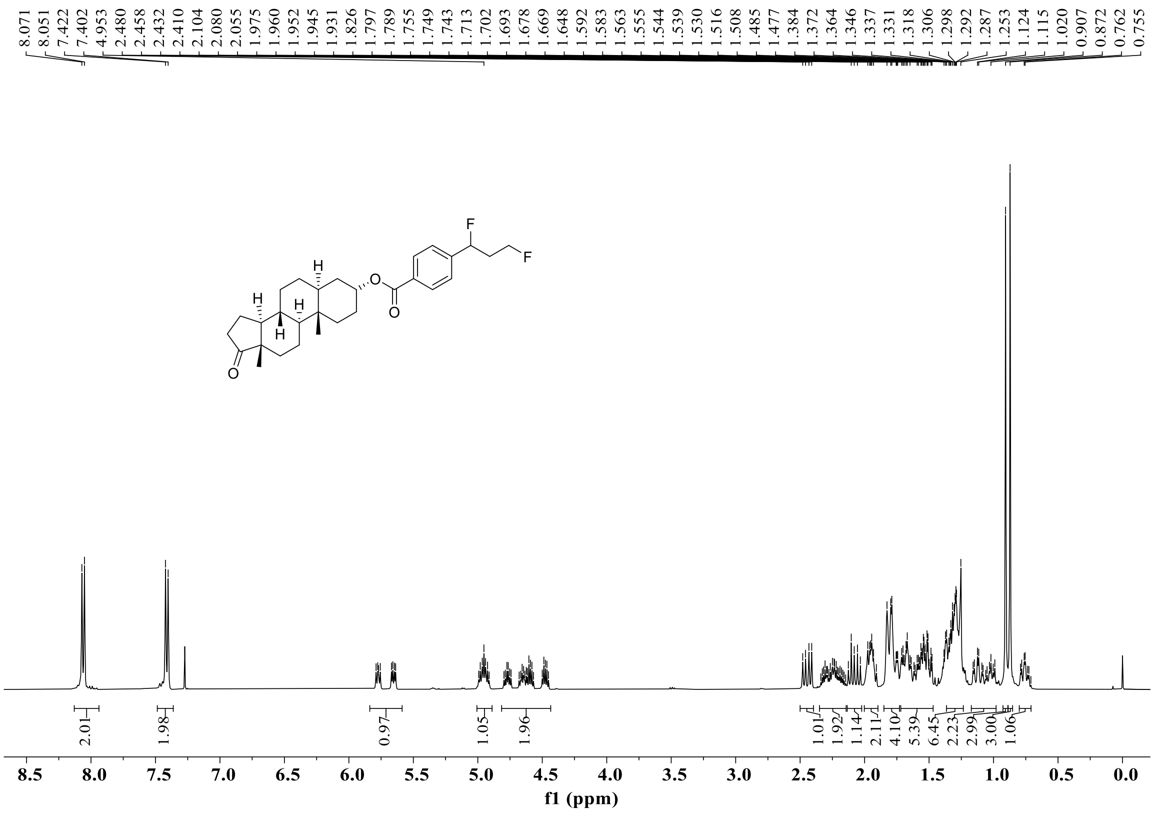

Supplementary Figure 96 ^{1}H NMR spectra of compound **35**

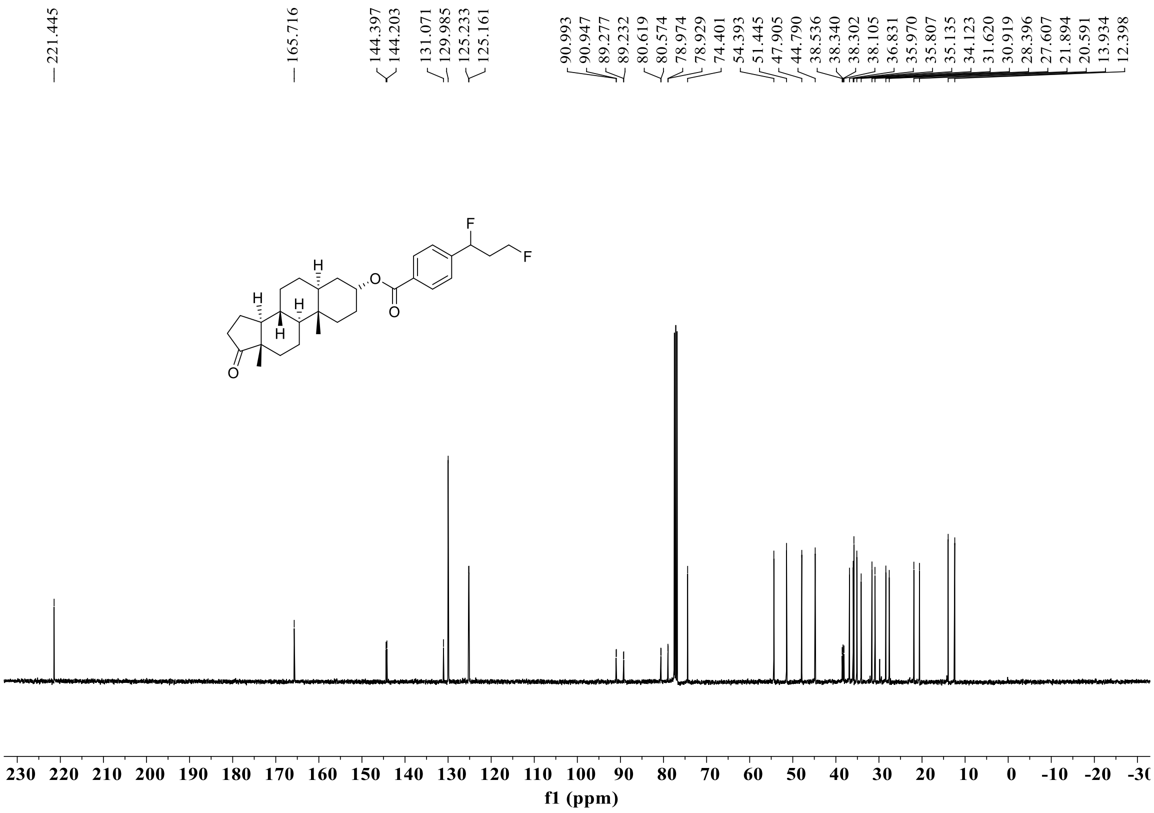

Supplementary Figure 97 ^{13}C NMR spectra of compound **35**

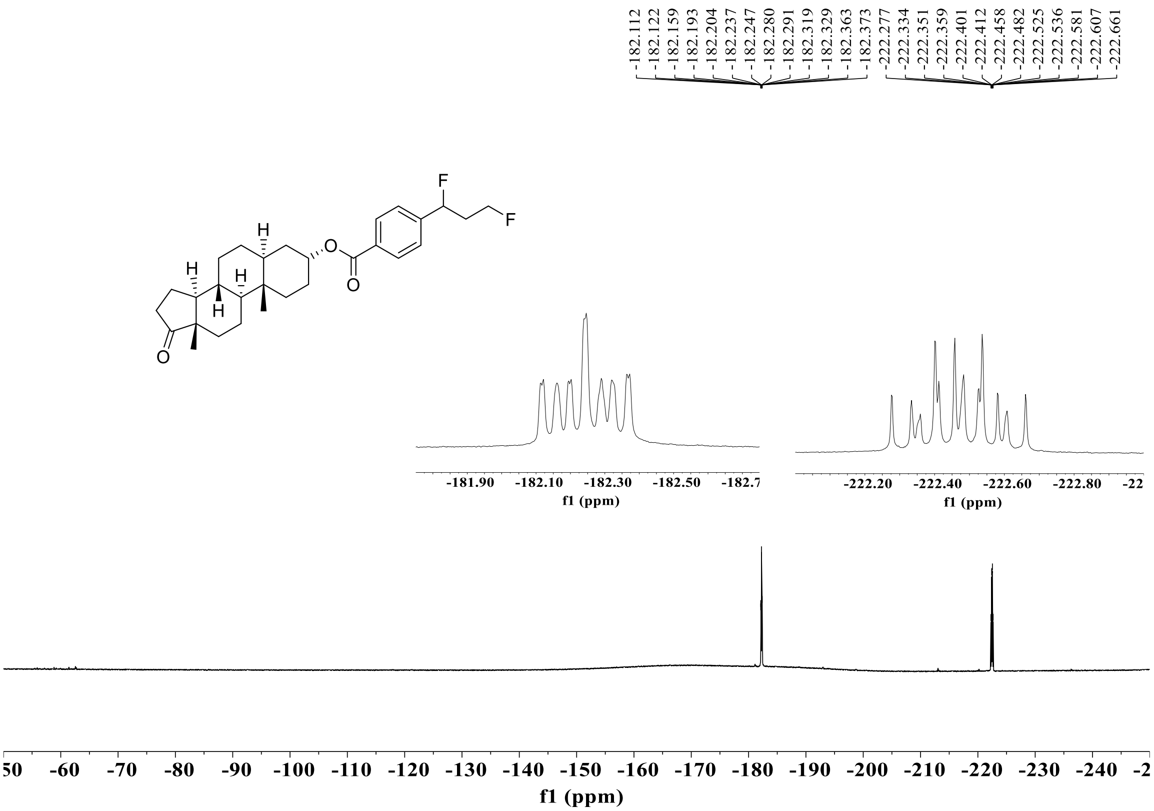

Supplementary Figure 98 ^{19}F NMR spectra of compound **35**

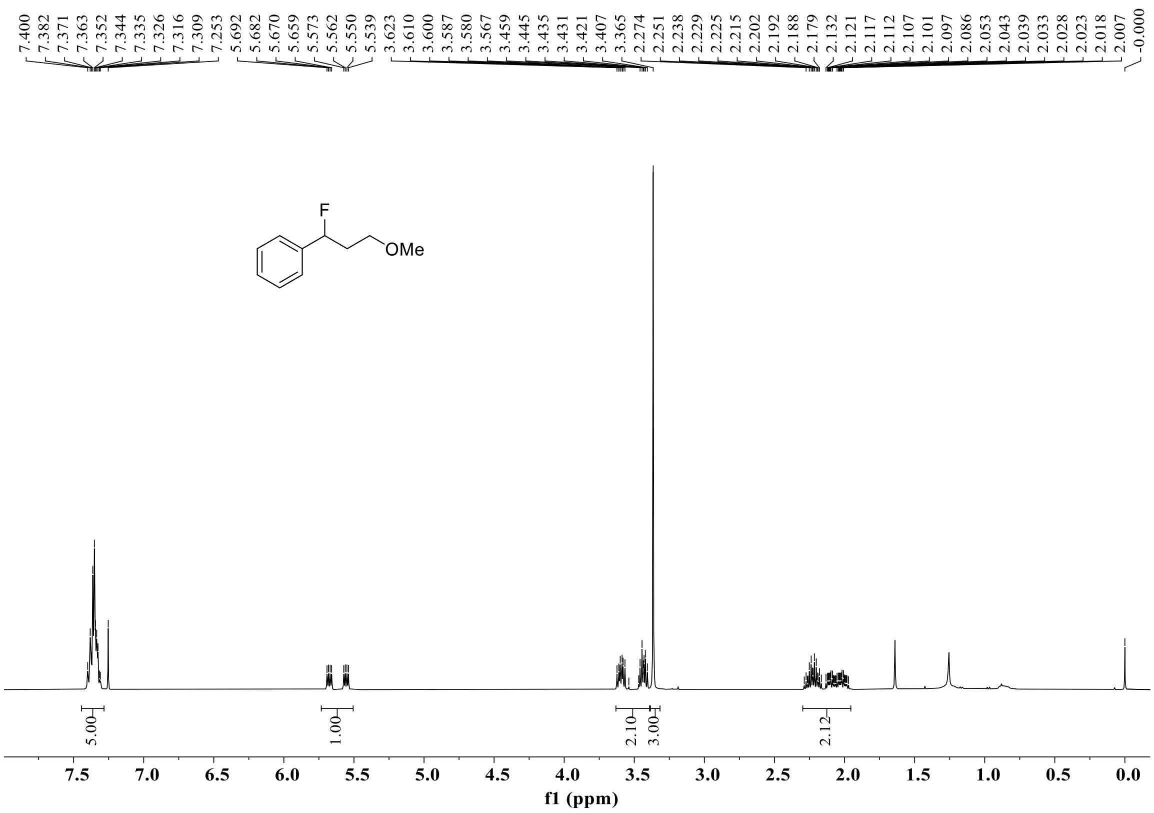

Supplementary Figure 99 ^{1}H NMR spectra of compound **3**

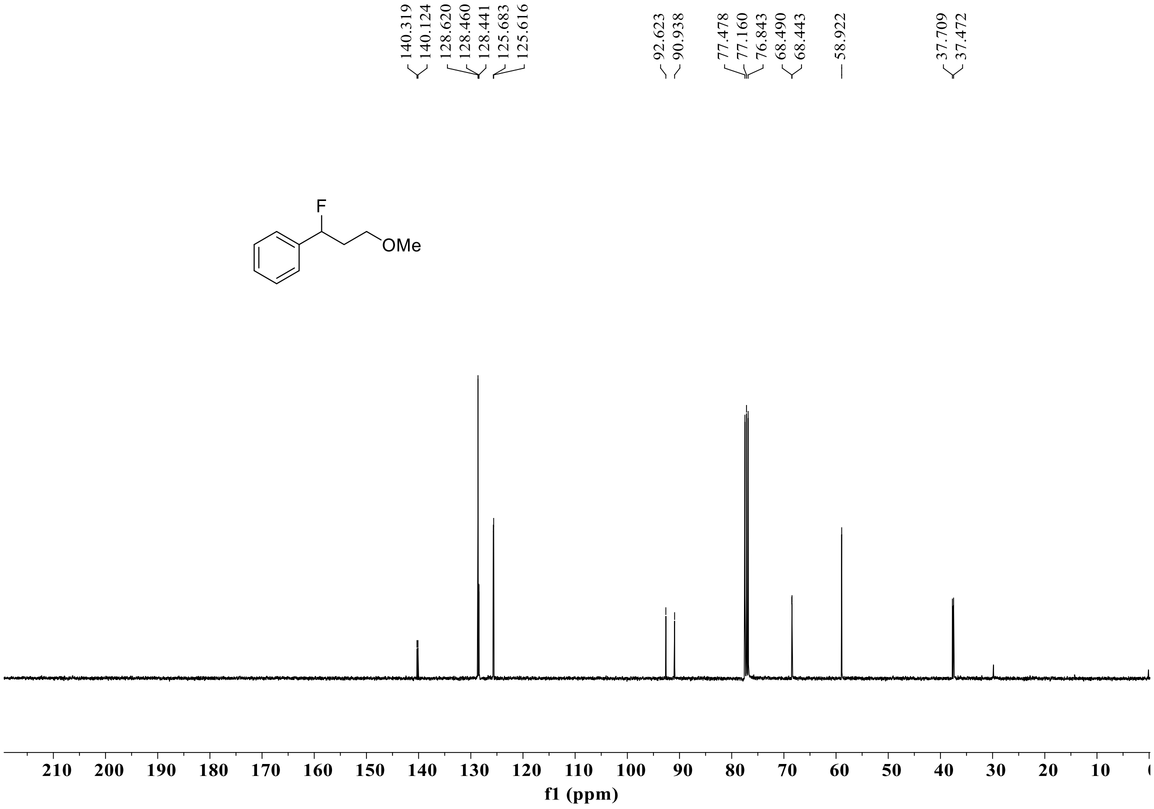

Supplementary Figure 100 ^{13}C NMR spectra of compound **3**

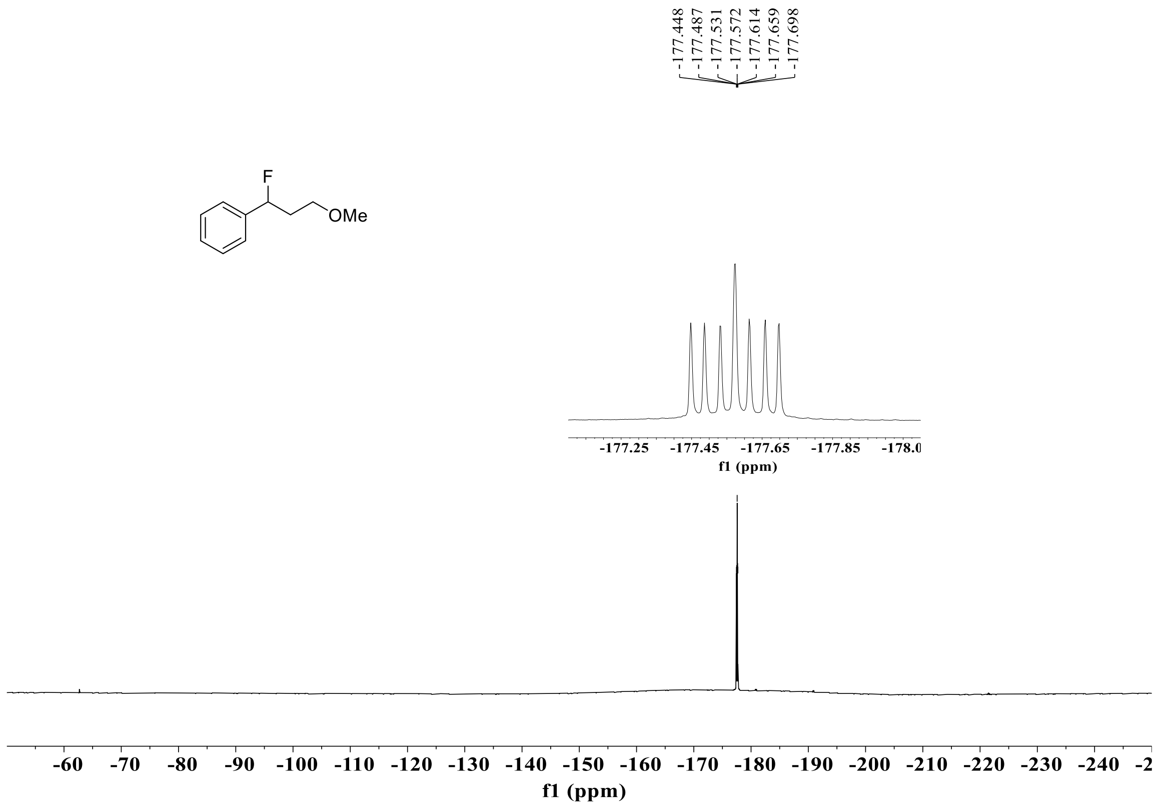

Supplementary Figure 101 ^{19}F NMR spectra of compound **3**

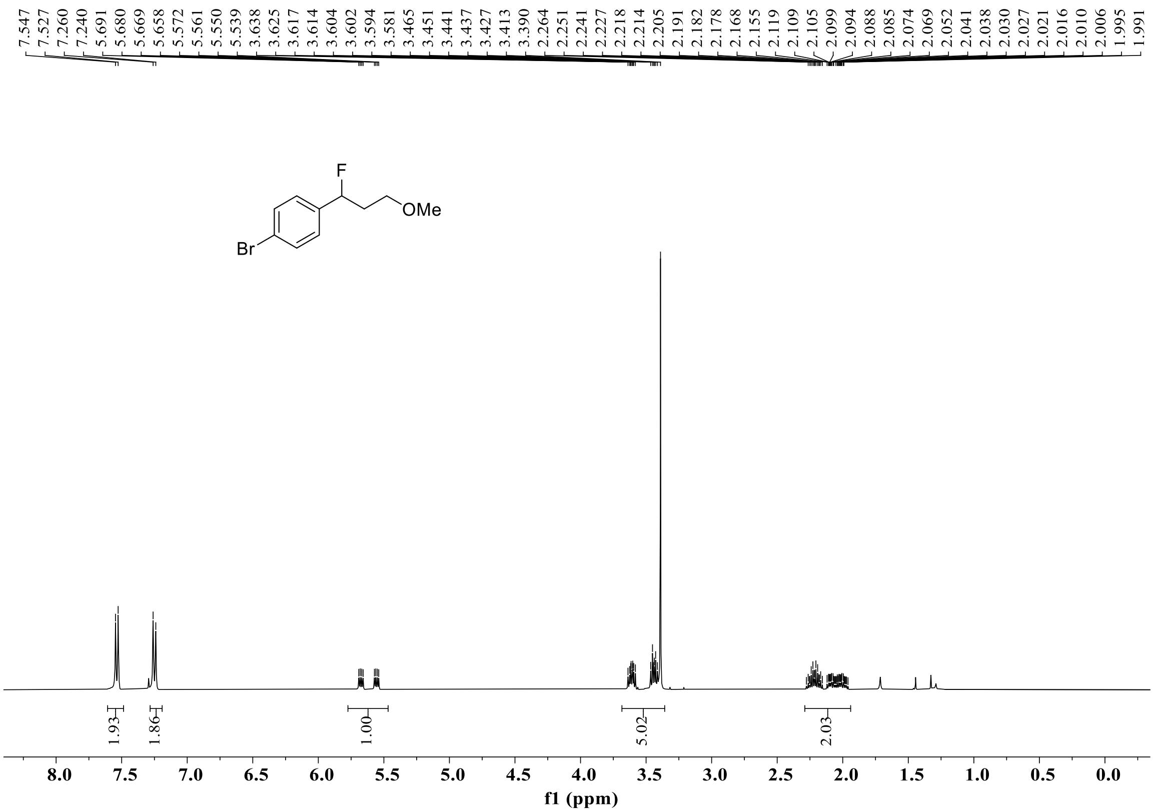

Supplementary Figure 102 ^{1}H NMR spectra of compound **36**

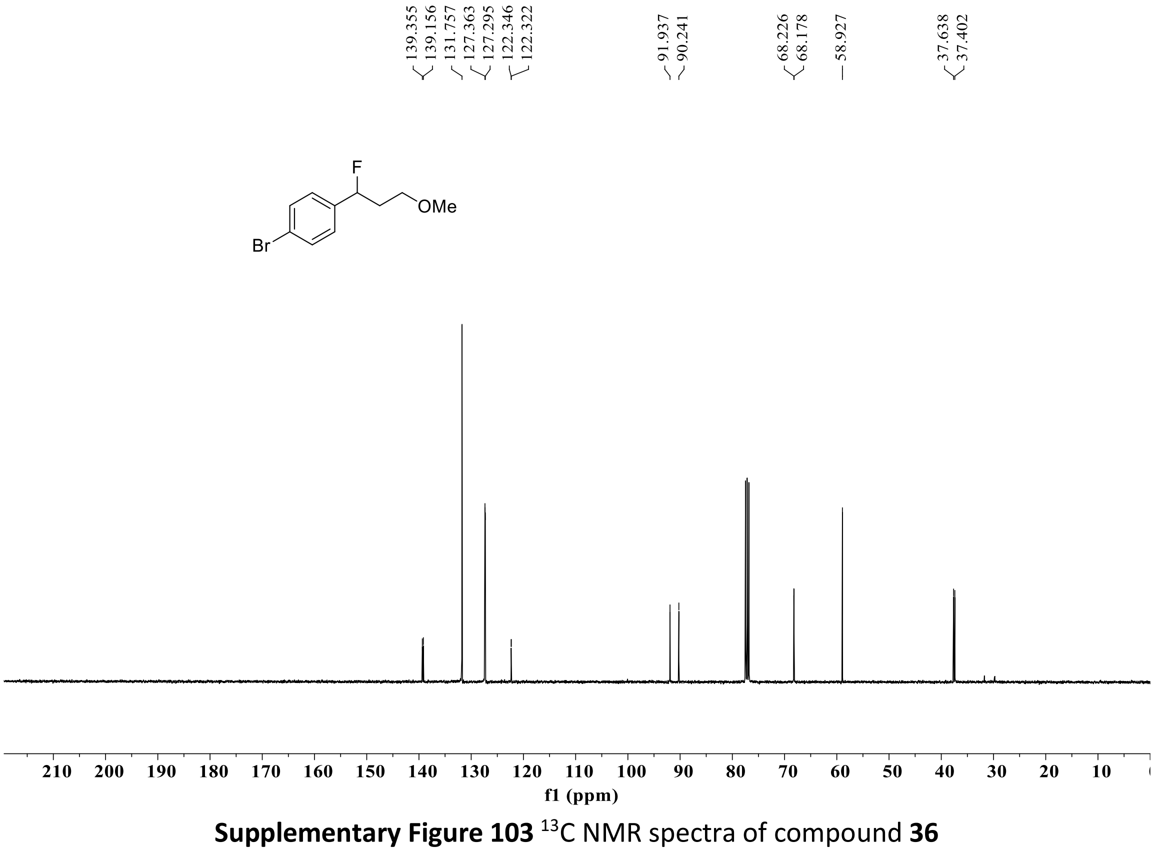

Supplementary Figure 103 ^{13}C NMR spectra of compound **36**

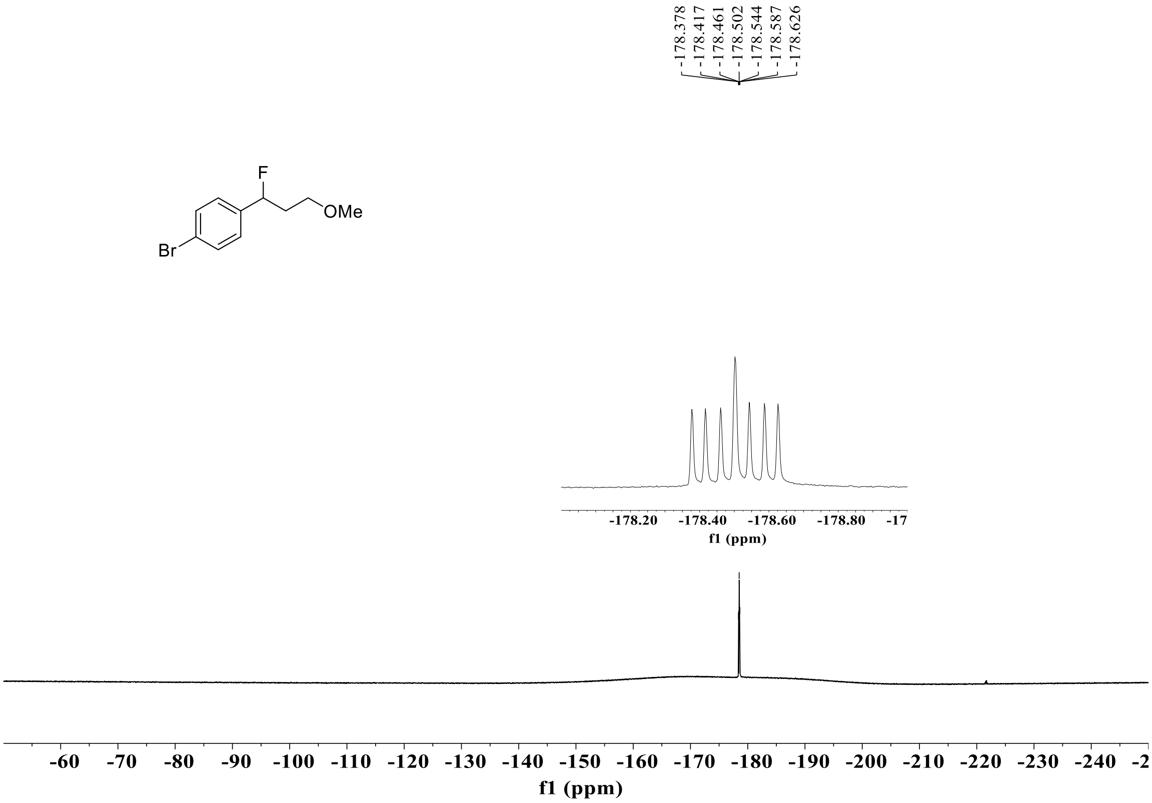

Supplementary Figure 103 ^{19}F NMR spectra of compound **36**

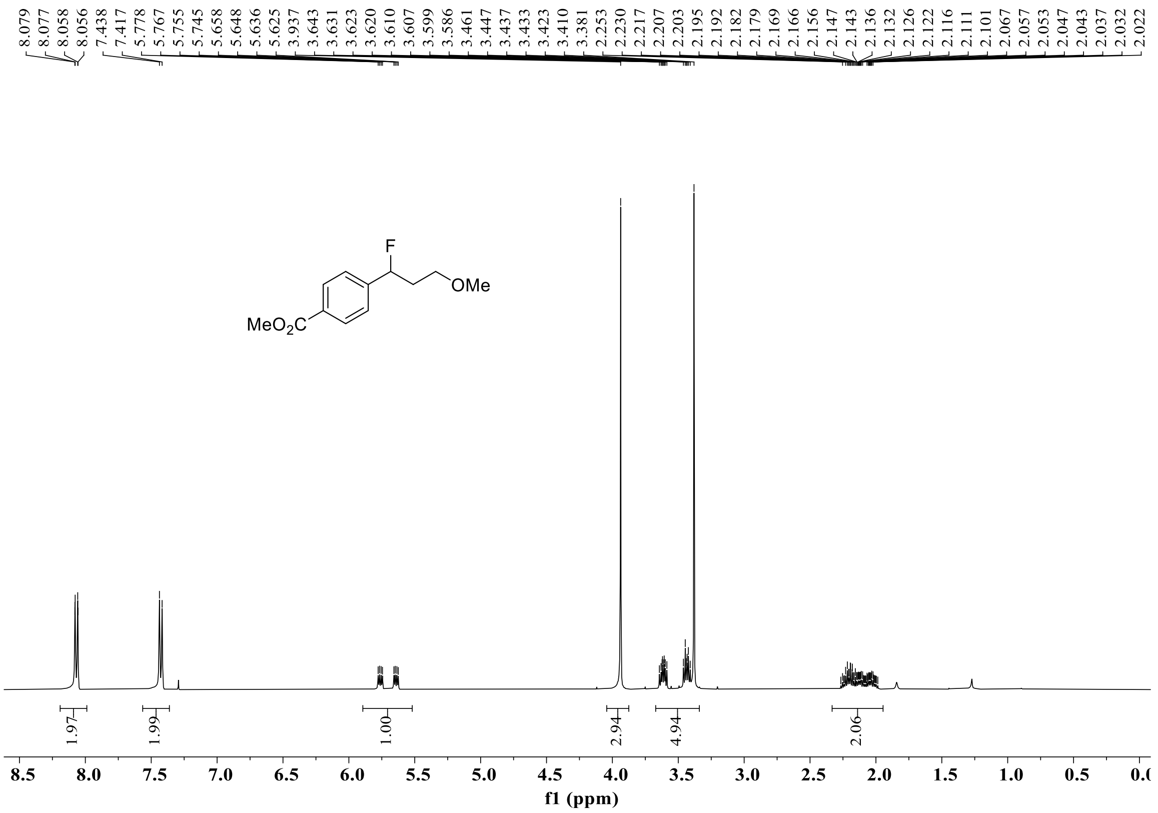

Supplementary Figure 104 ^{1}H NMR spectra of compound **37**

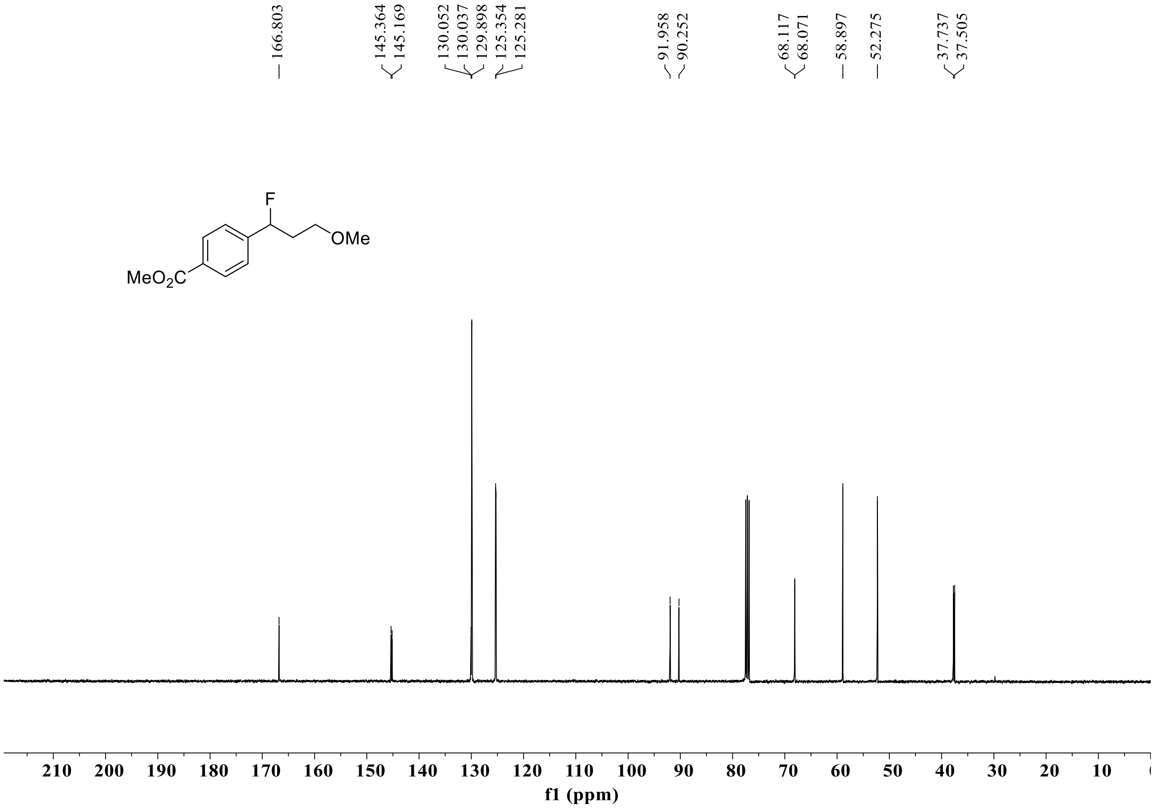

Supplementary Figure 105 ^{13}C NMR spectra of compound **37**

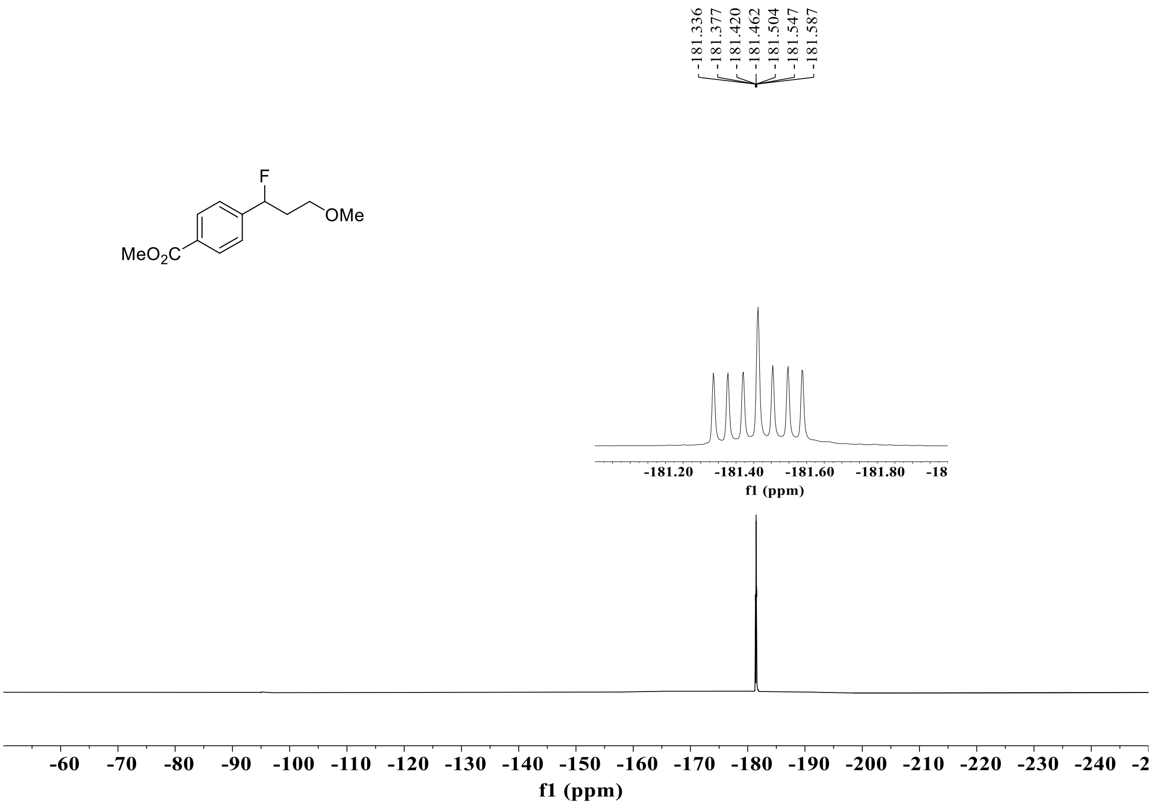

Supplementary Figure 106 ^{19}F NMR spectra of compound **37**

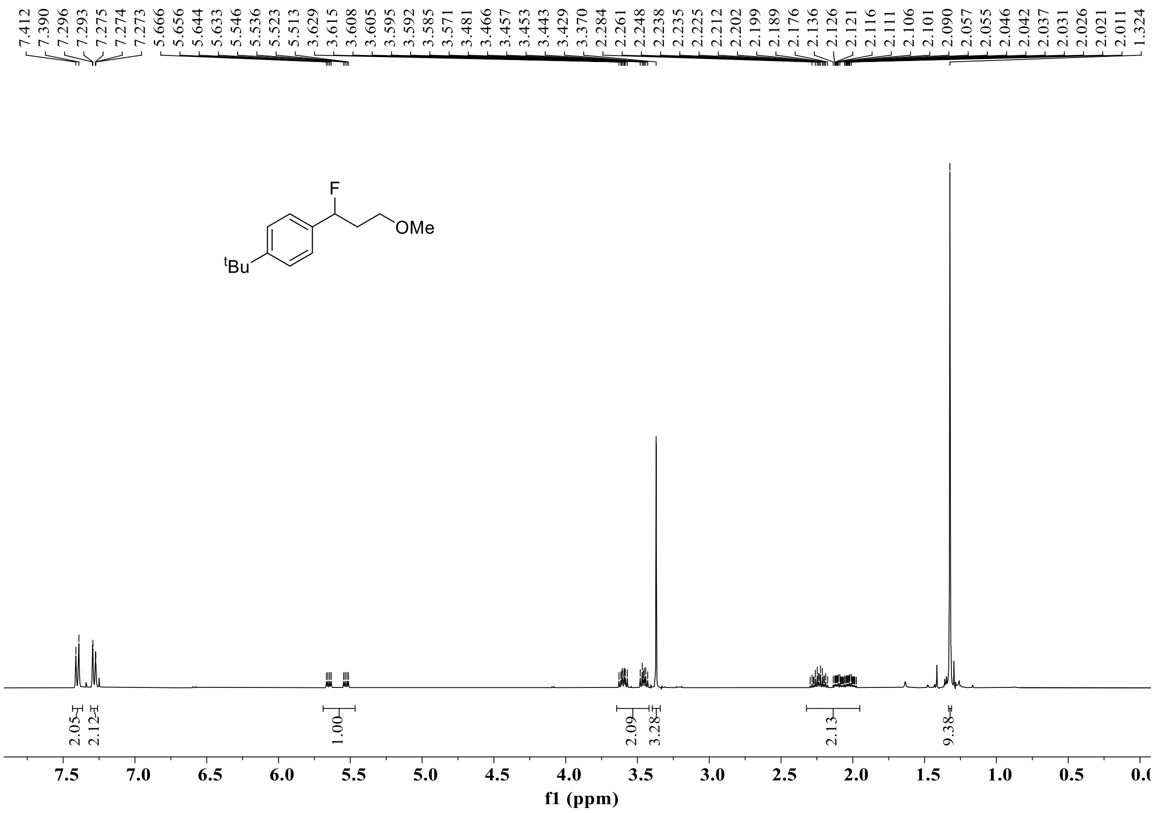

Supplementary Figure 107 ^{1}H NMR spectra of compound **38**

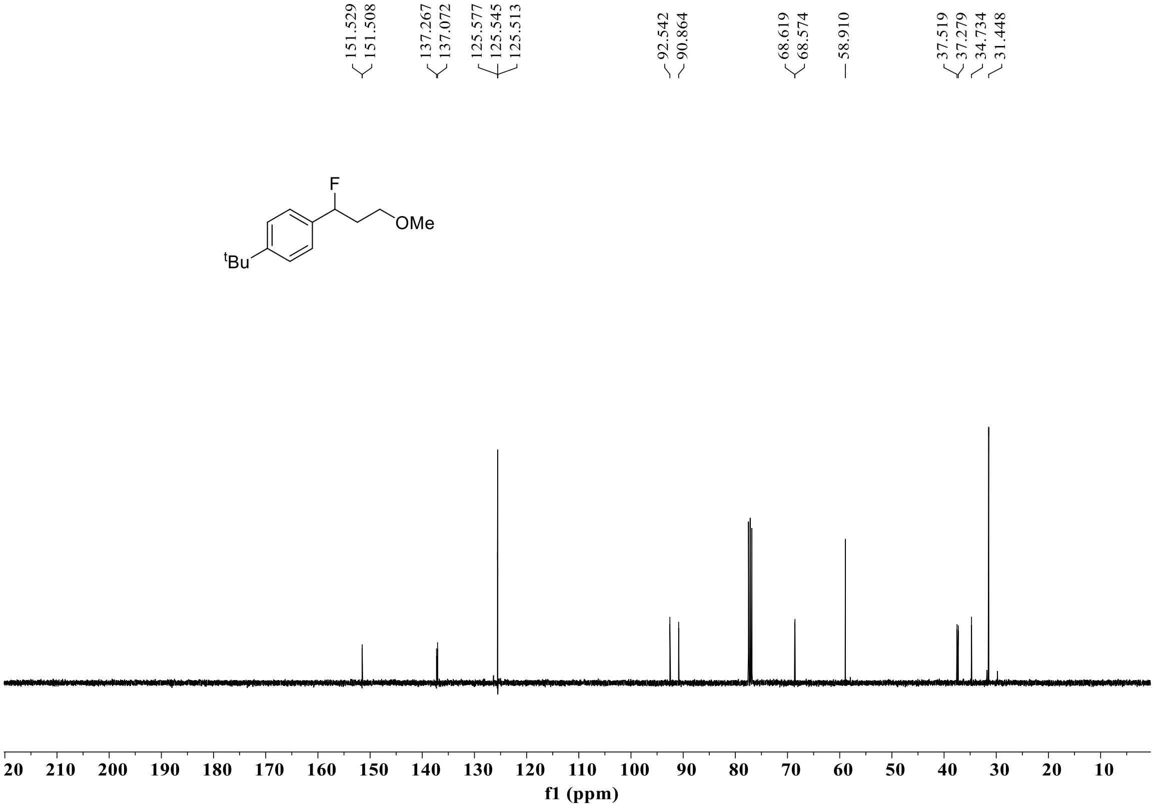

Supplementary Figure 108 ^{13}C NMR spectra of compound **38**

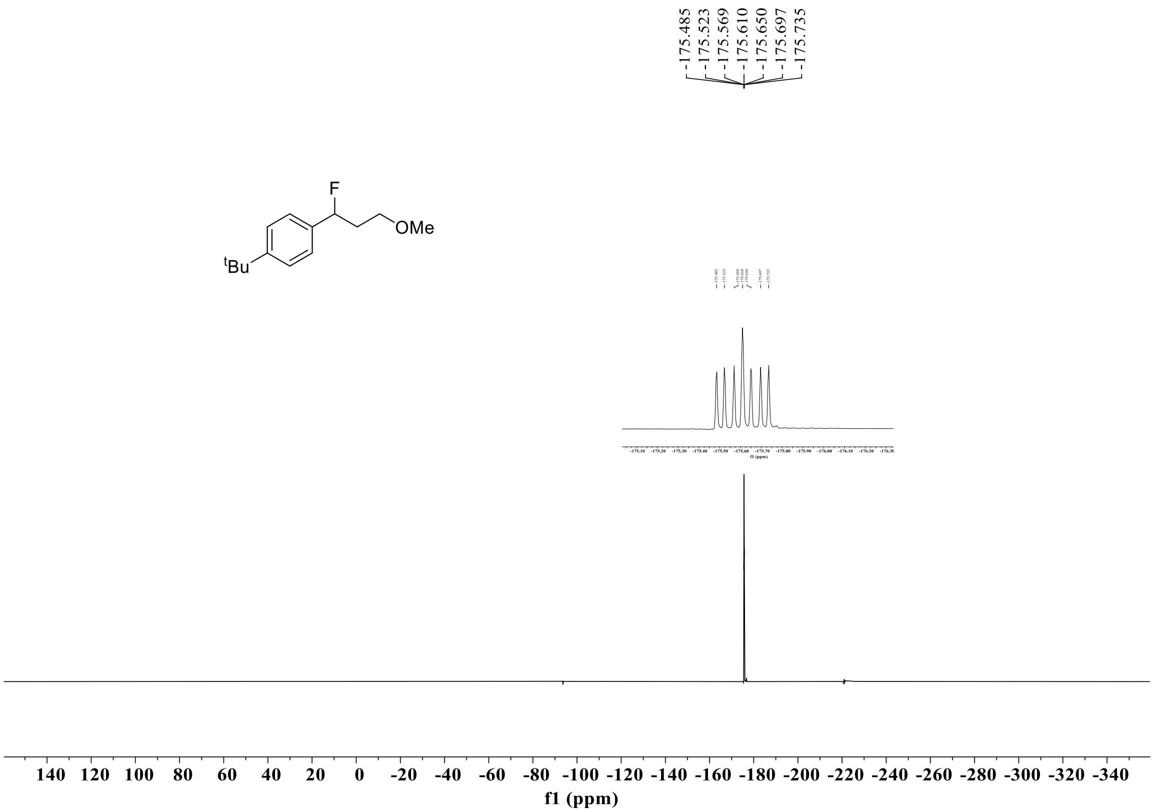

Supplementary Figure 109 ^{19}F NMR spectra of compound **38**

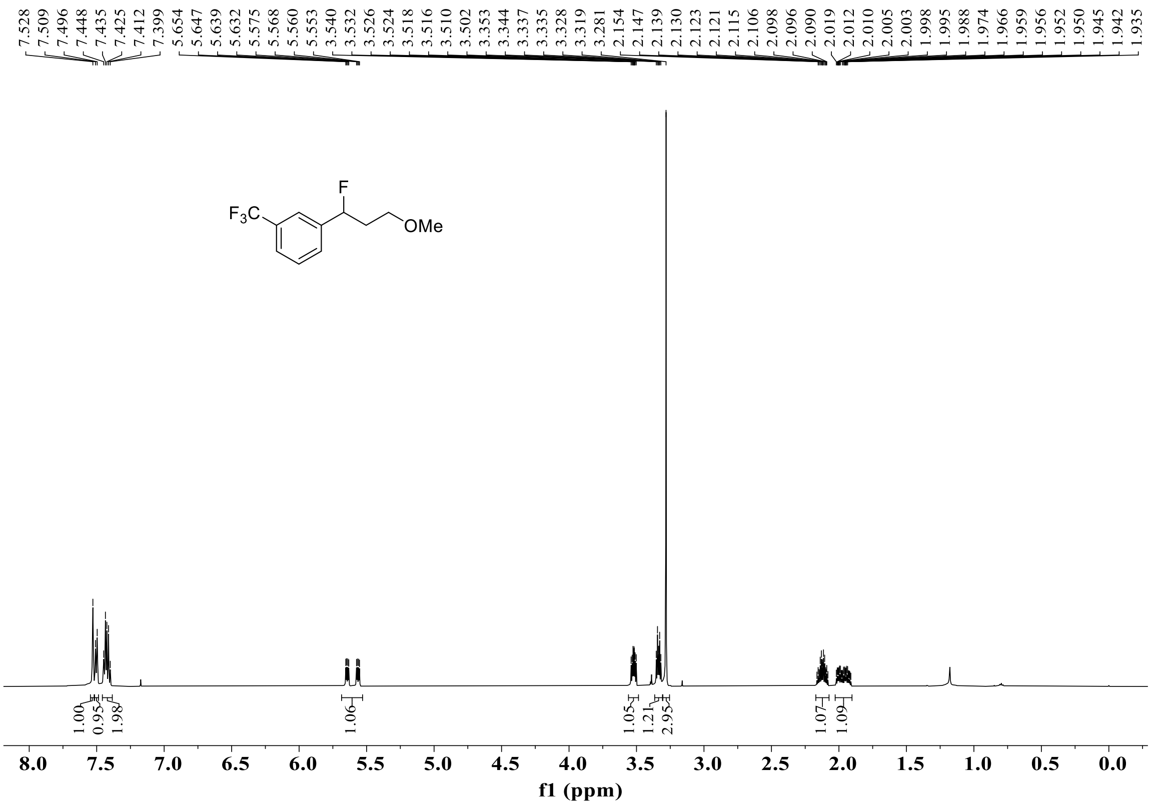

Supplementary Figure 110 ^{1}H NMR spectra of compound **39**

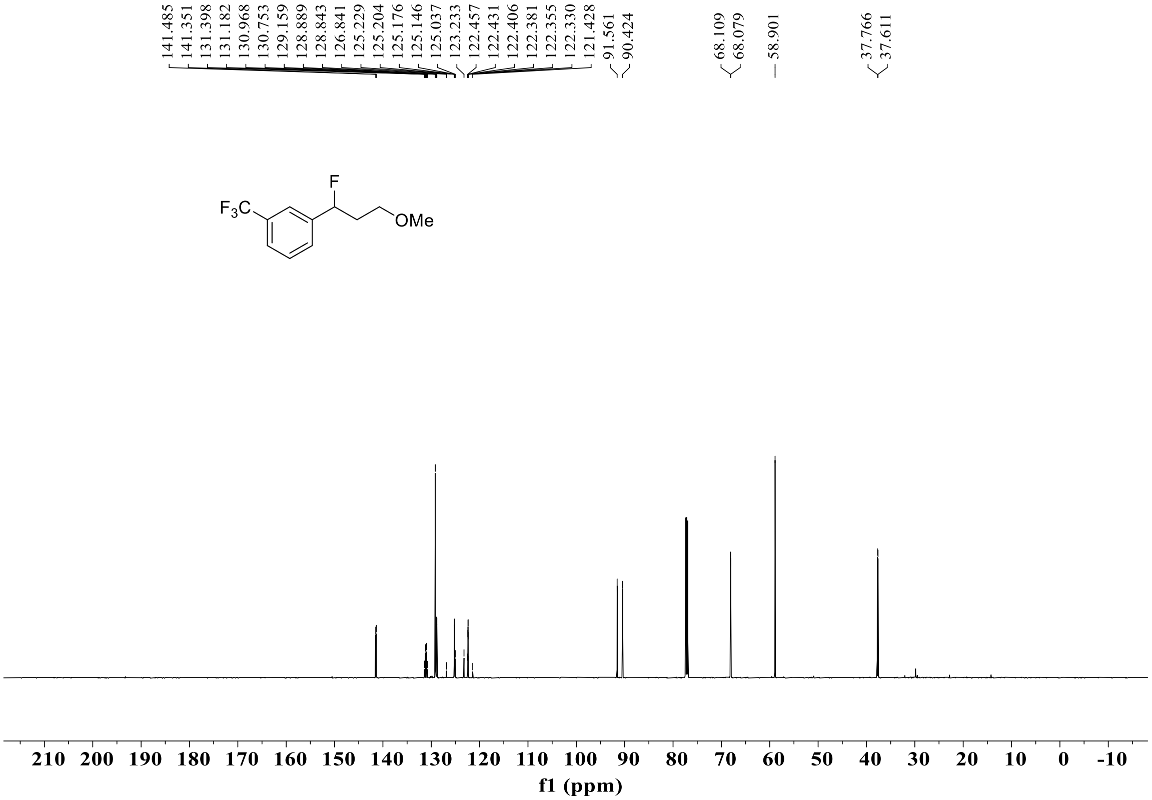

Supplementary Figure 111 ^{13}C NMR spectra of compound **39**

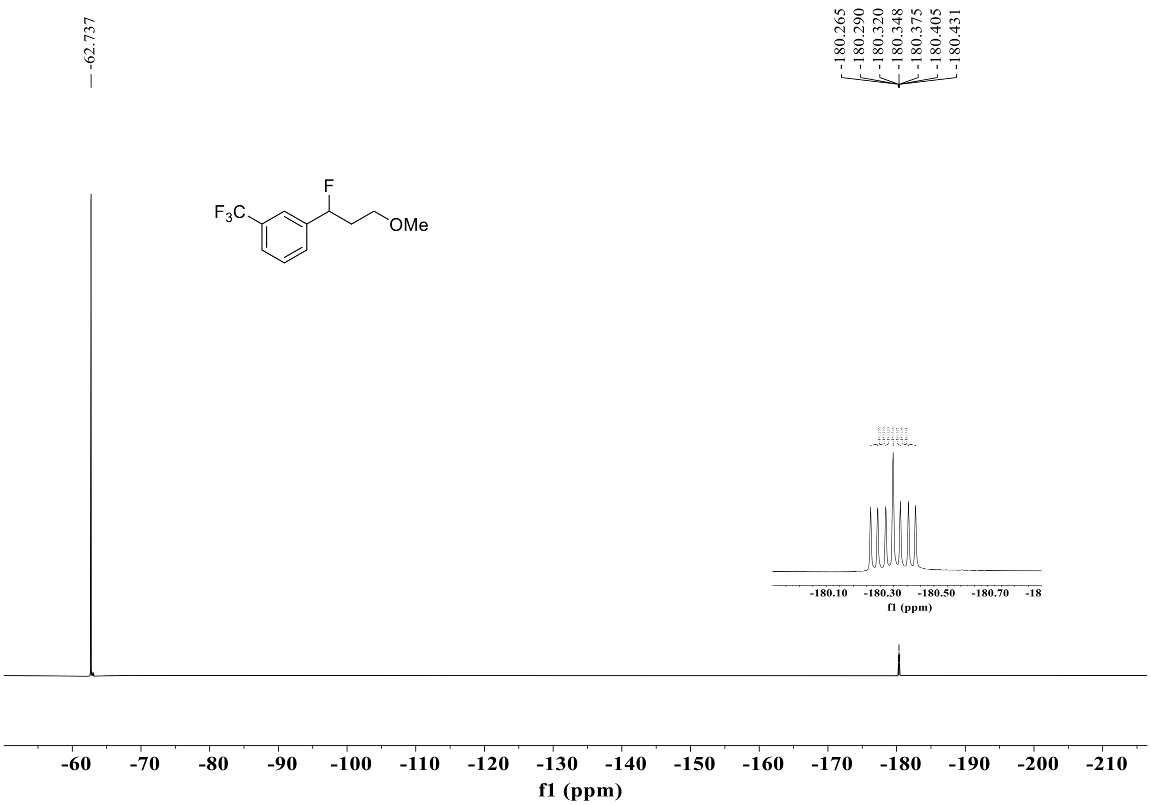

Supplementary Figure 112 ^{19}F NMR spectra of compound **39**

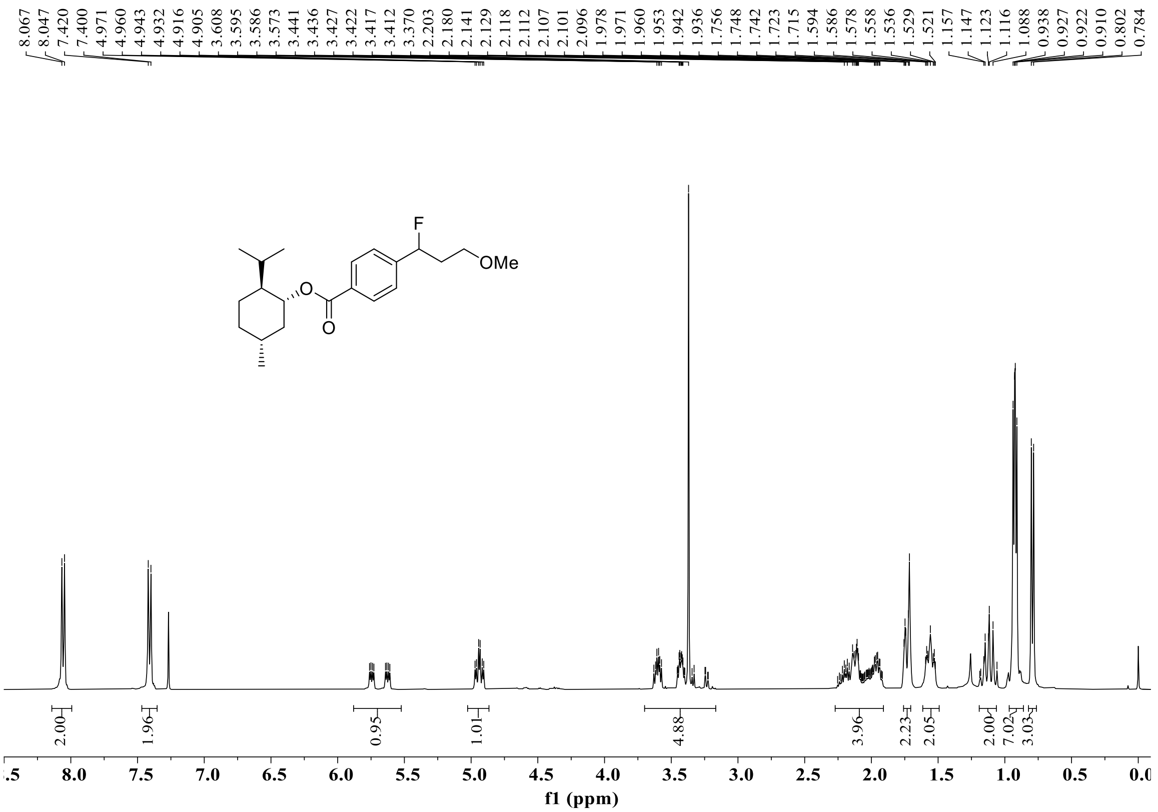

Supplementary Figure 113 ^{1}H NMR spectra of compound **40**

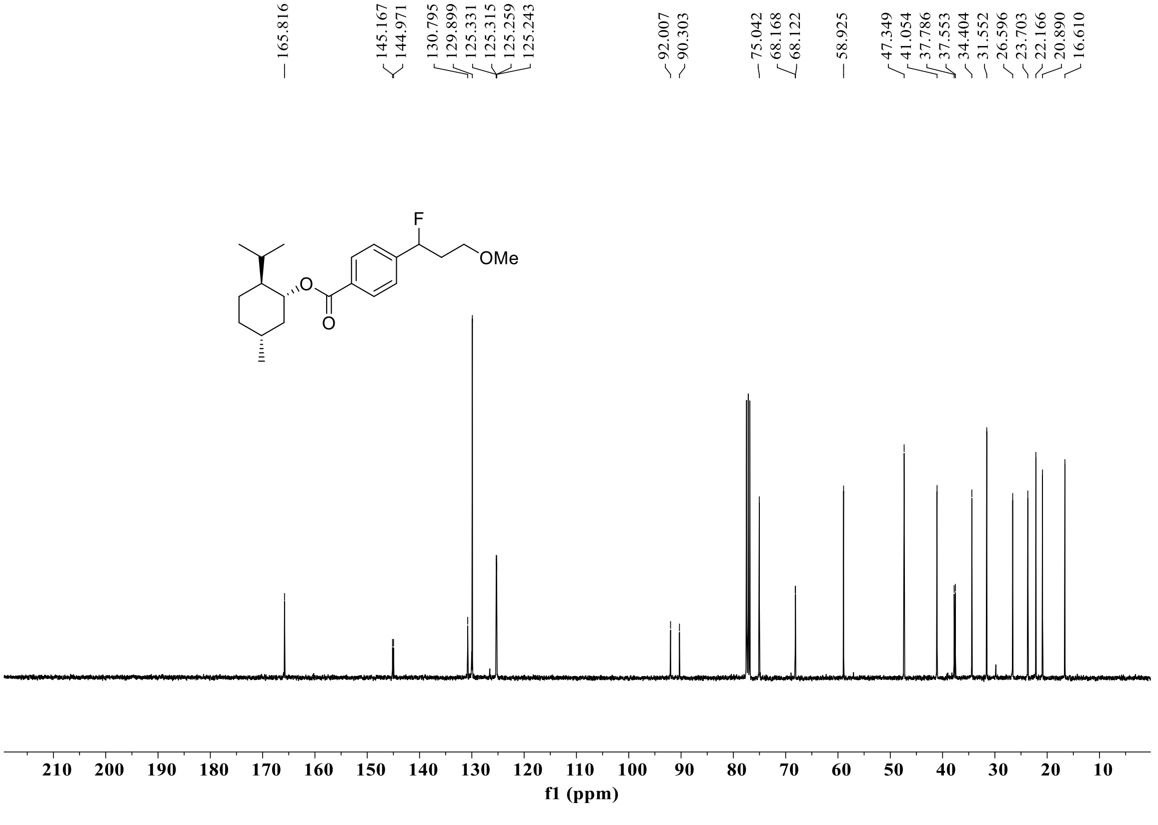

Supplementary Figure 114 ^{13}C NMR spectra of compound **40**

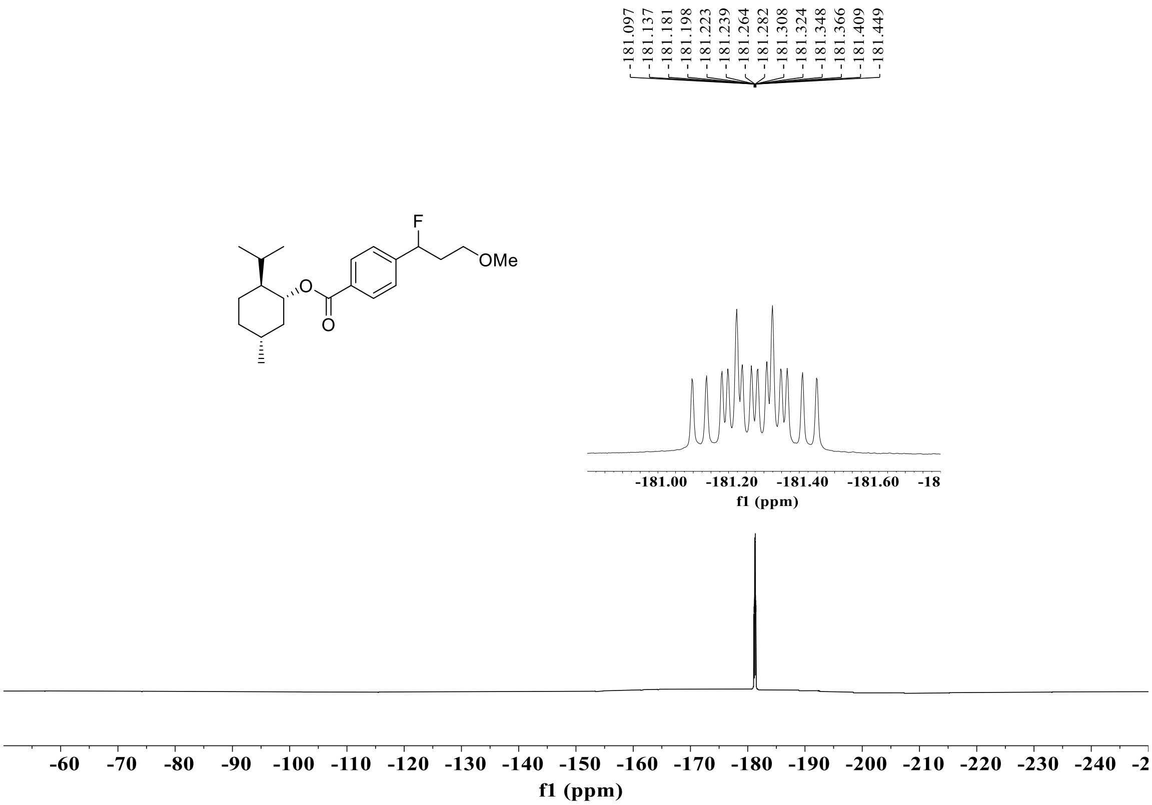

Supplementary Figure 115 ^{19}F NMR spectra of compound **40**

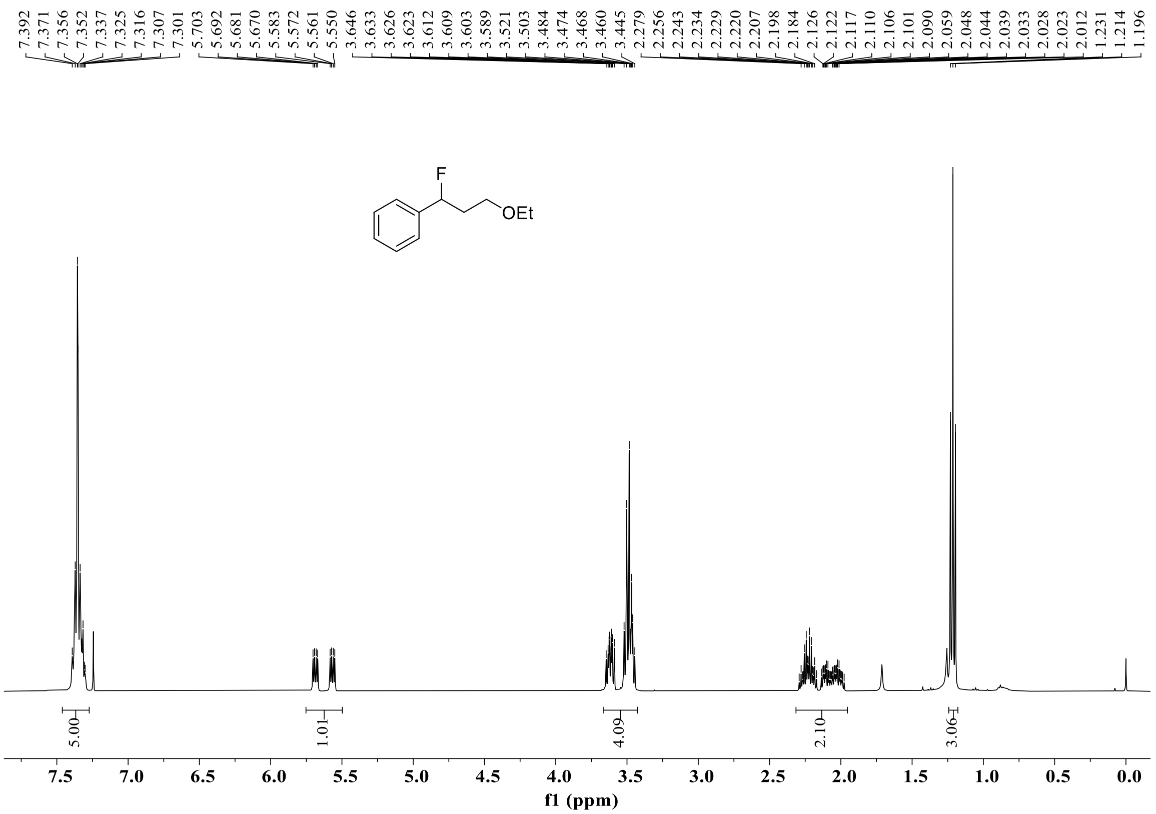

Supplementary Figure 116 ^{1}H NMR spectra of compound **41**

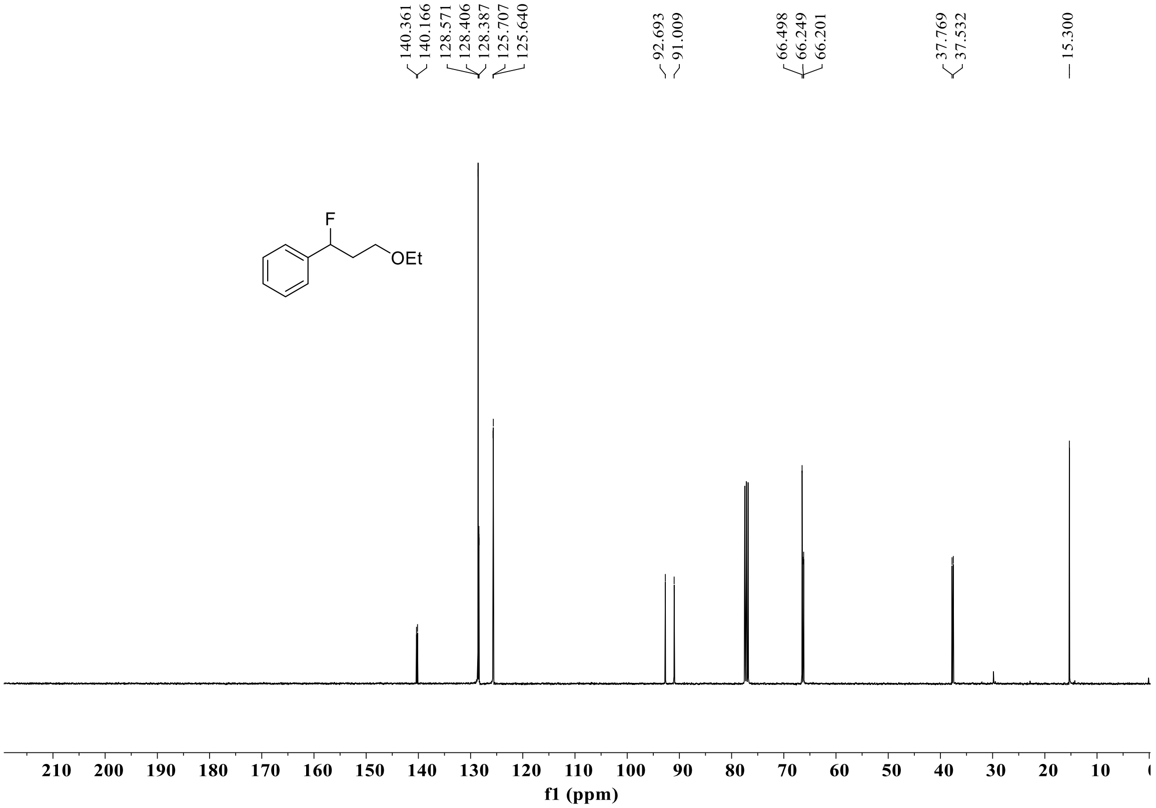

Supplementary Figure 117 ^{13}C NMR spectra of compound **41**

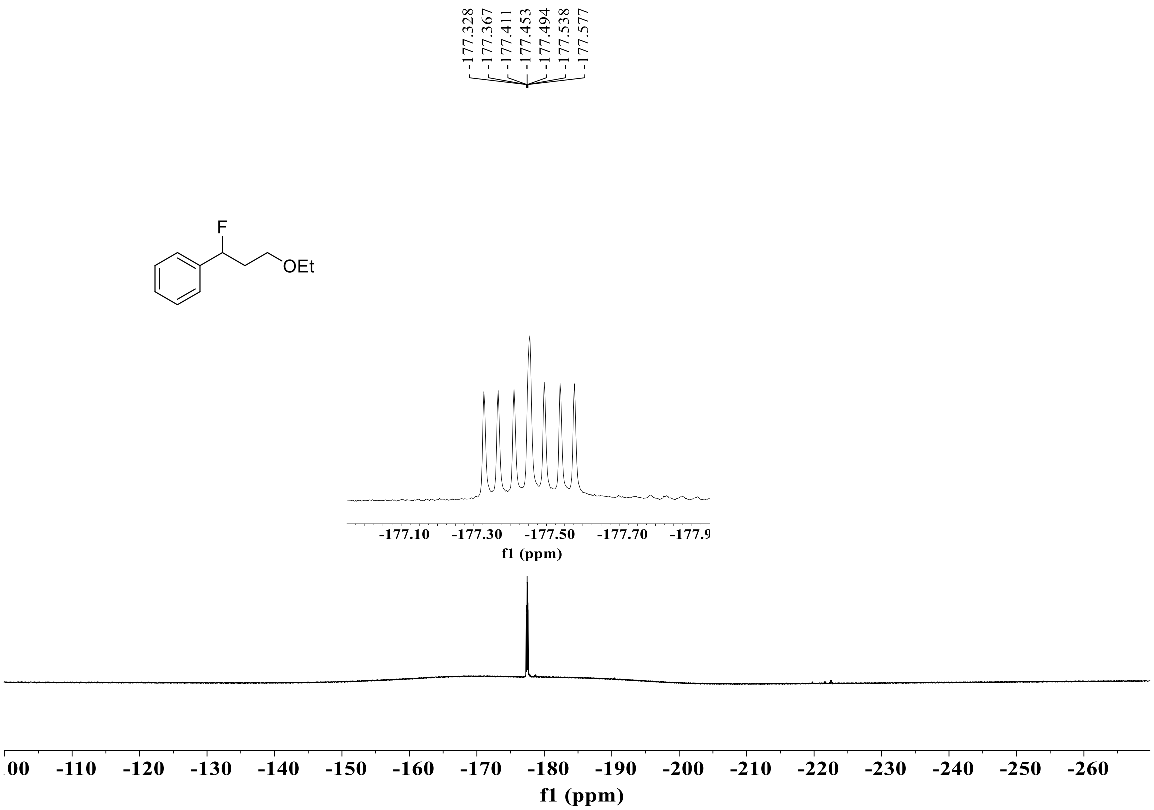

Supplementary Figure 118 ^{19}F NMR spectra of compound **41**

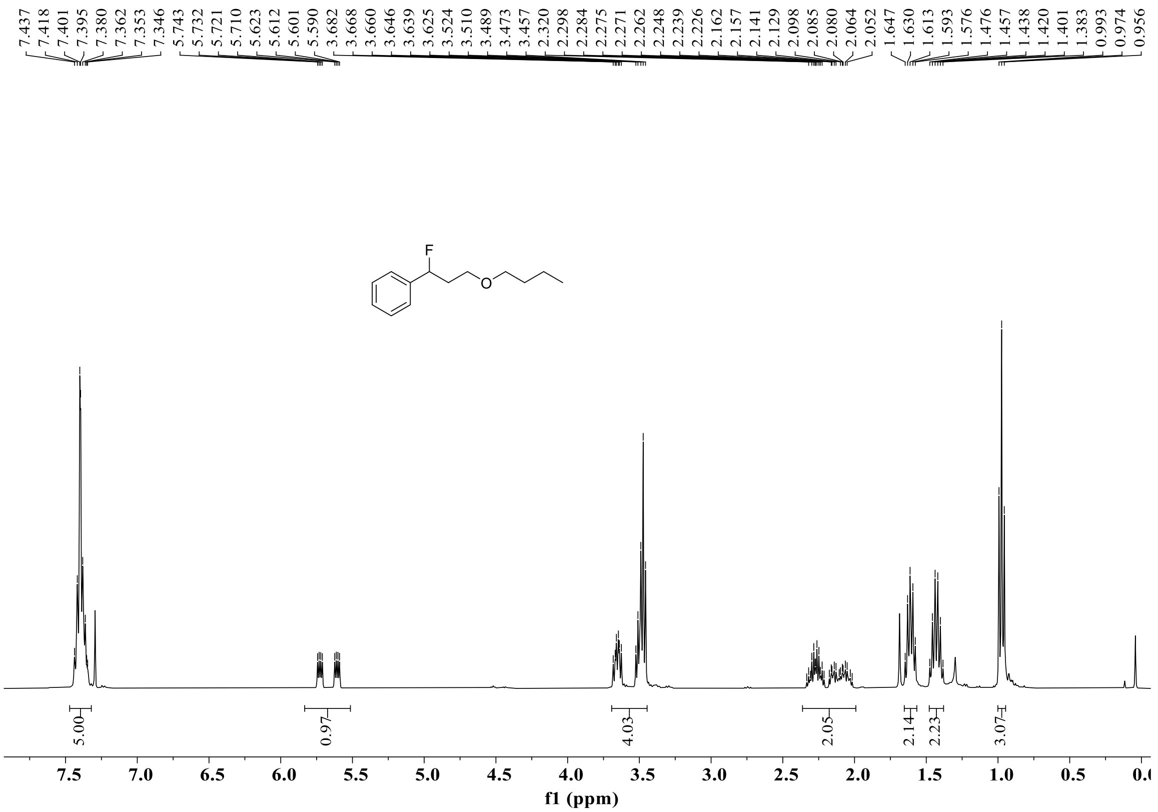

Supplementary Figure 119 ^{1}H NMR spectra of compound **42**

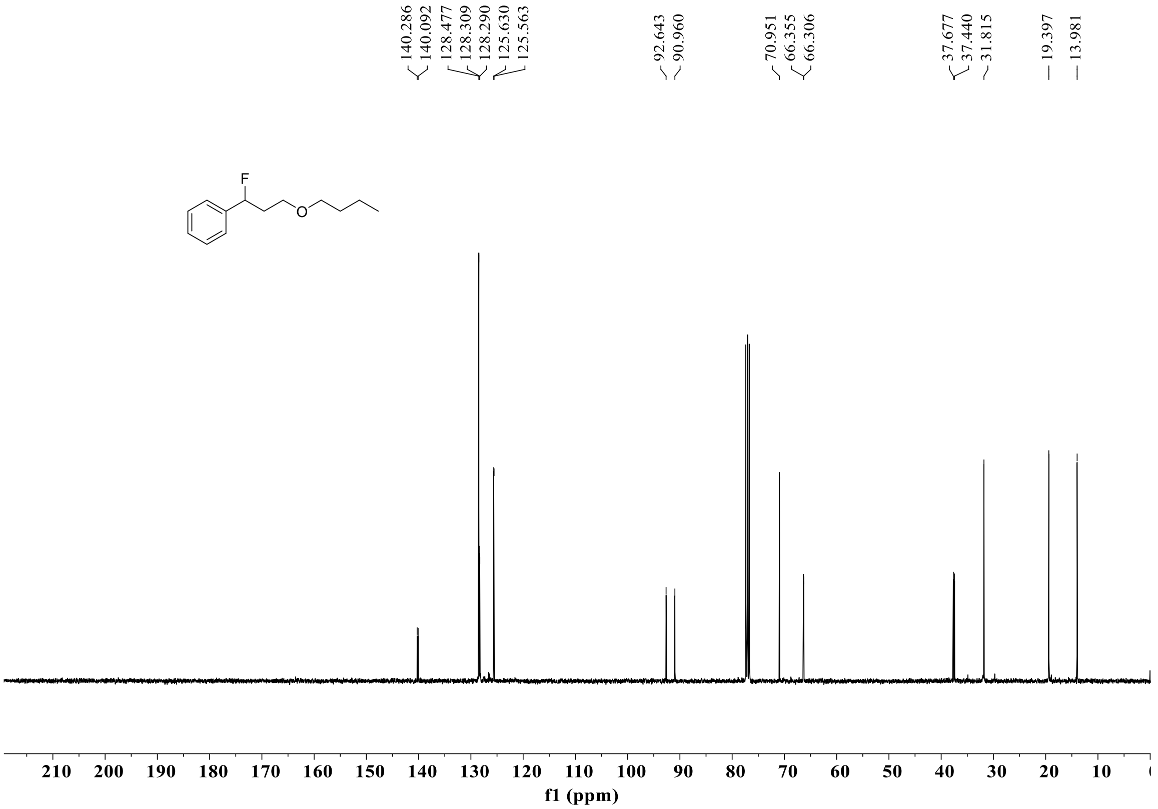

Supplementary Figure 120 ^{13}C NMR spectra of compound **42**

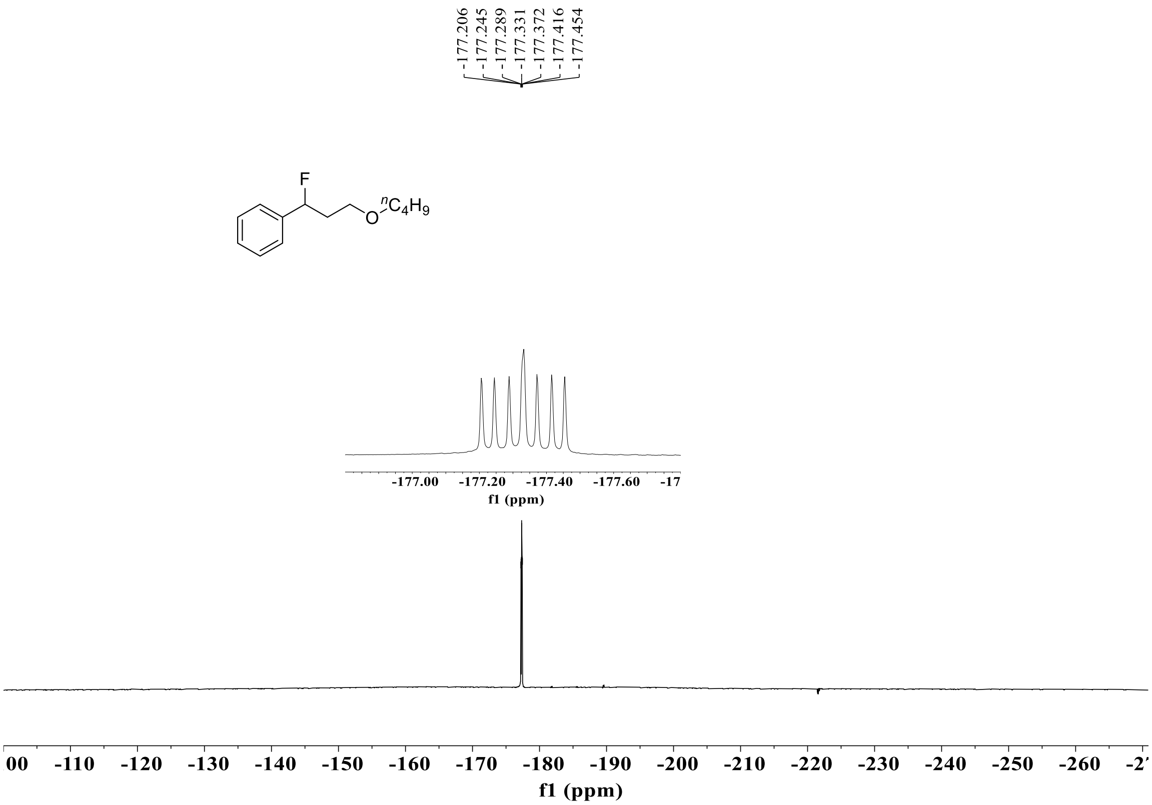

Supplementary Figure 121 ^{19}F NMR spectra of compound **42**

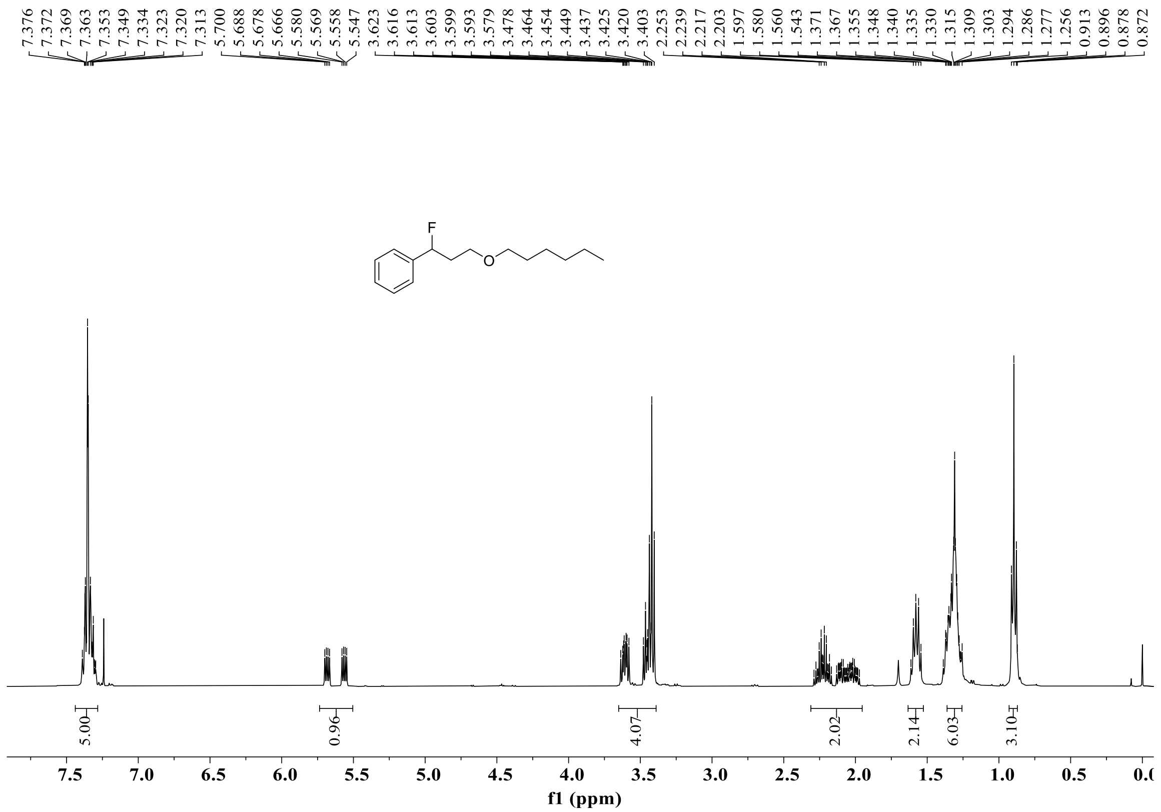

Supplementary Figure 122 ^{1}H NMR spectra of compound **43**

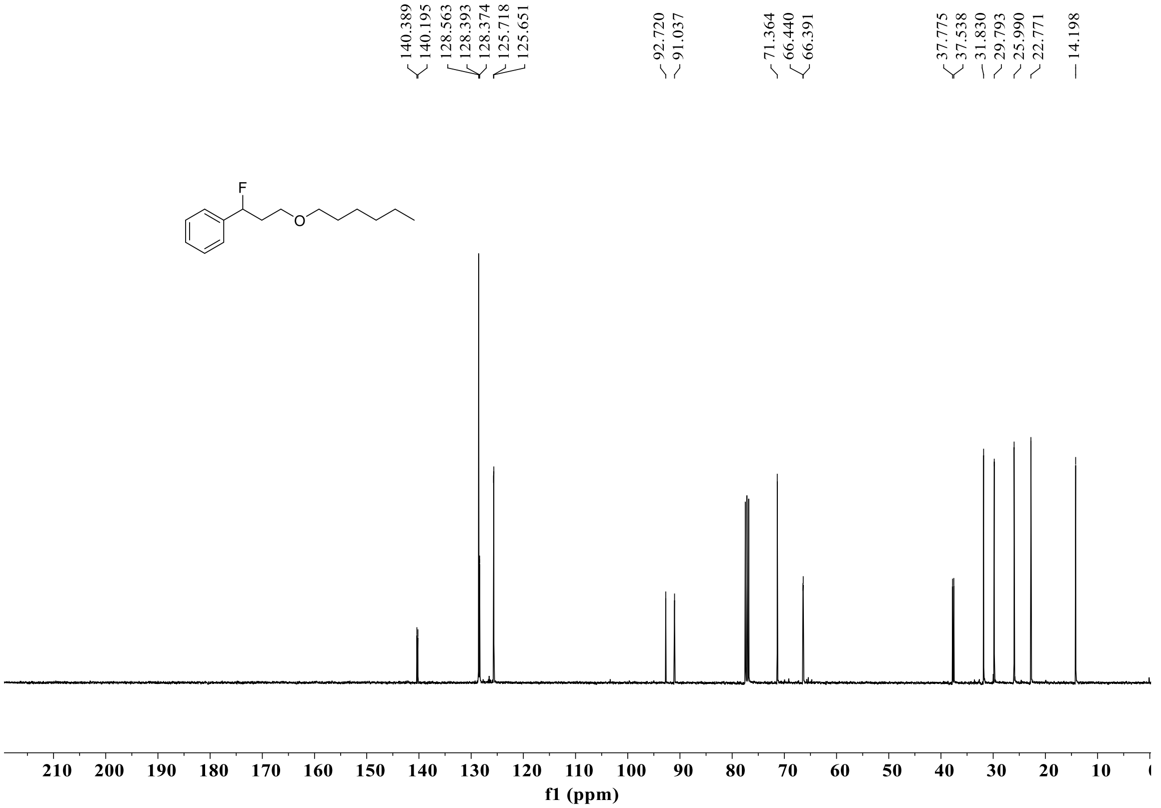

Supplementary Figure 123 ^{13}C NMR spectra of compound **43**

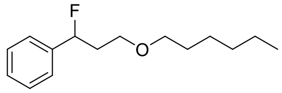

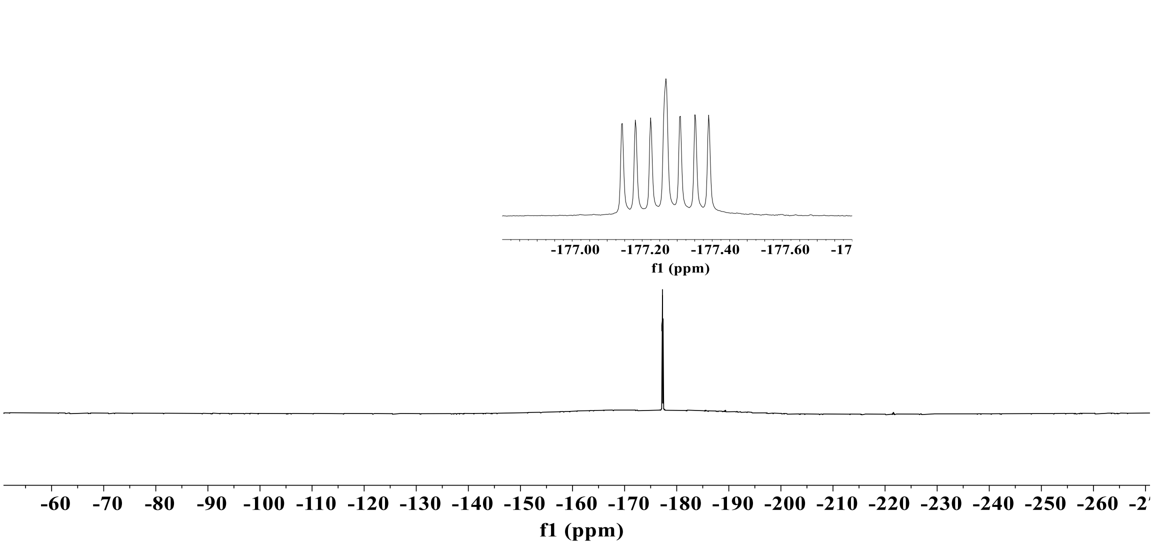

Supplementary Figure 124 ^{19}F NMR spectra of compound **43**

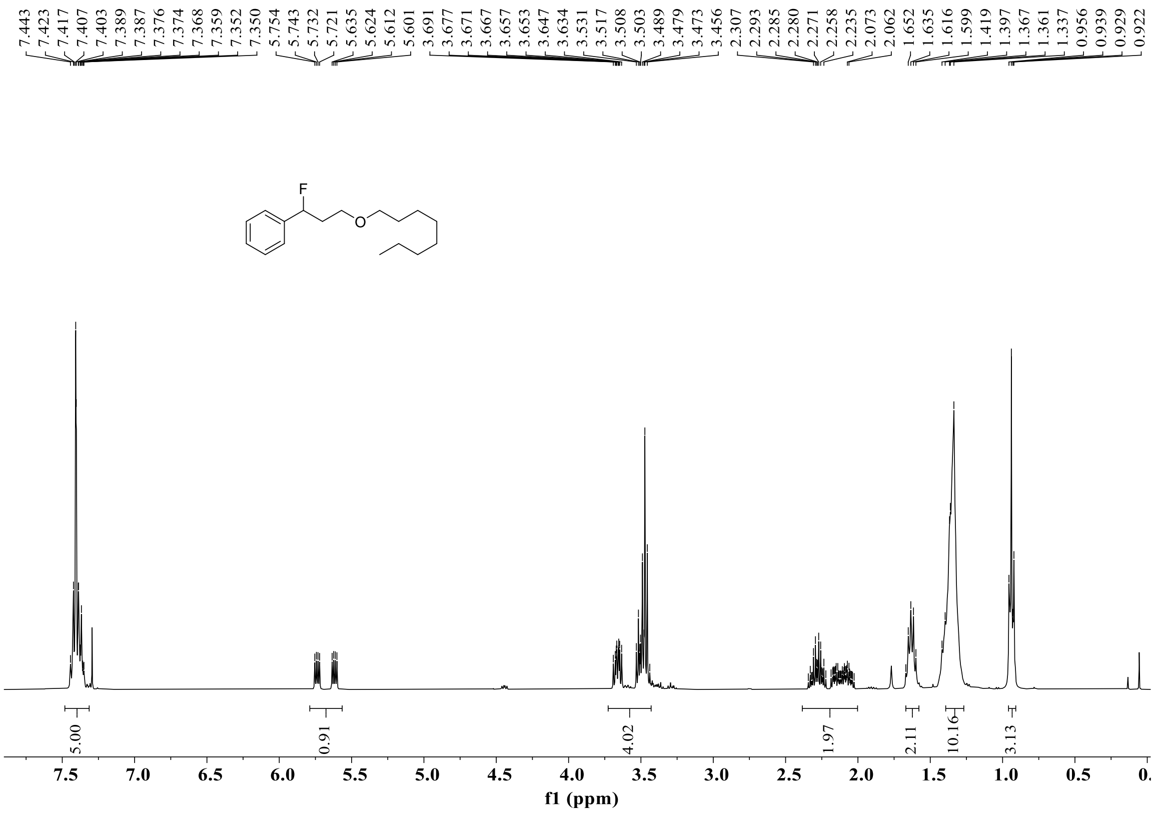

Supplementary Figure 125 ^{1}H NMR spectra of compound **44**

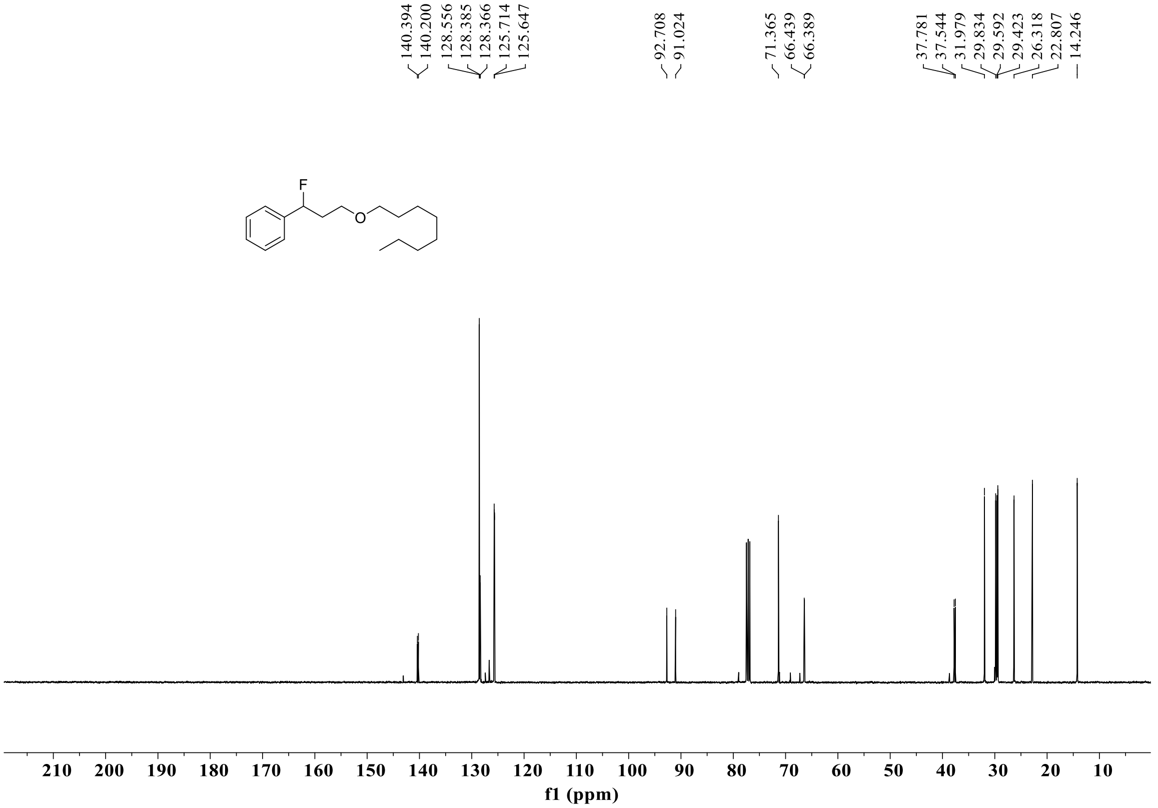

Supplementary Figure 126 ^{13}C NMR spectra of compound **44**

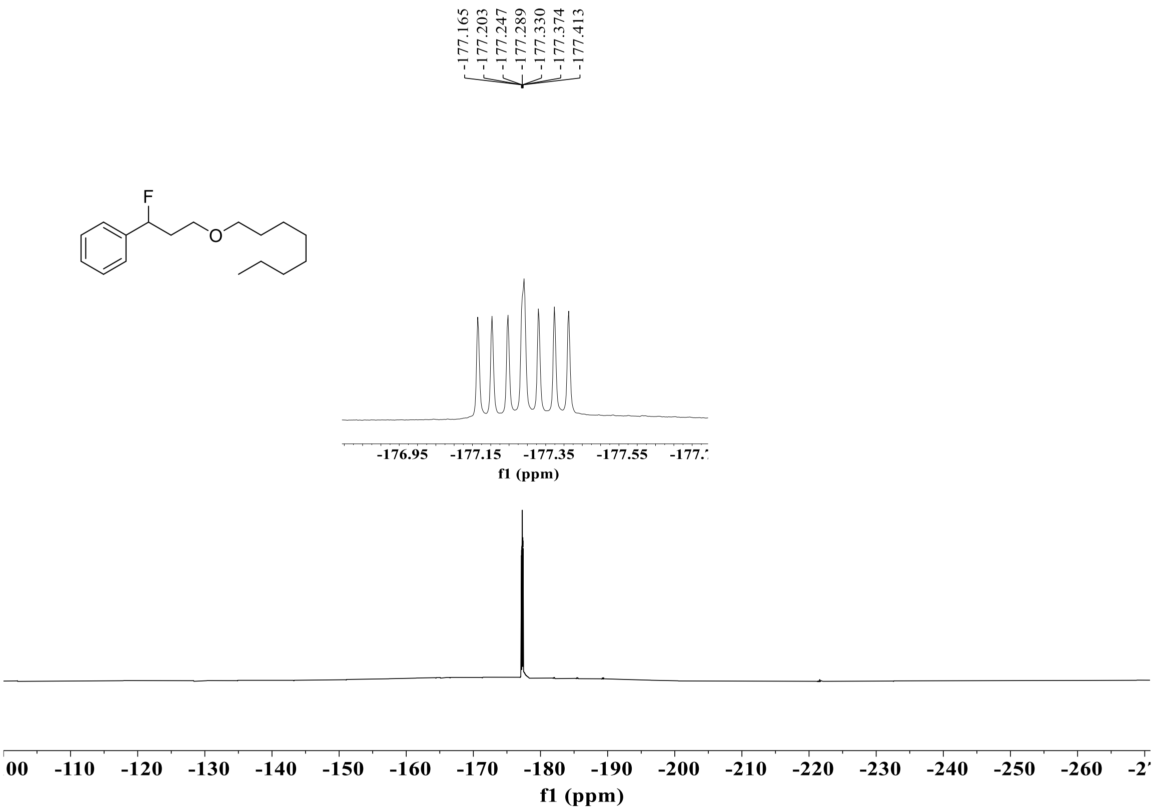

Supplementary Figure 127 ^{19}F NMR spectra of compound **44**

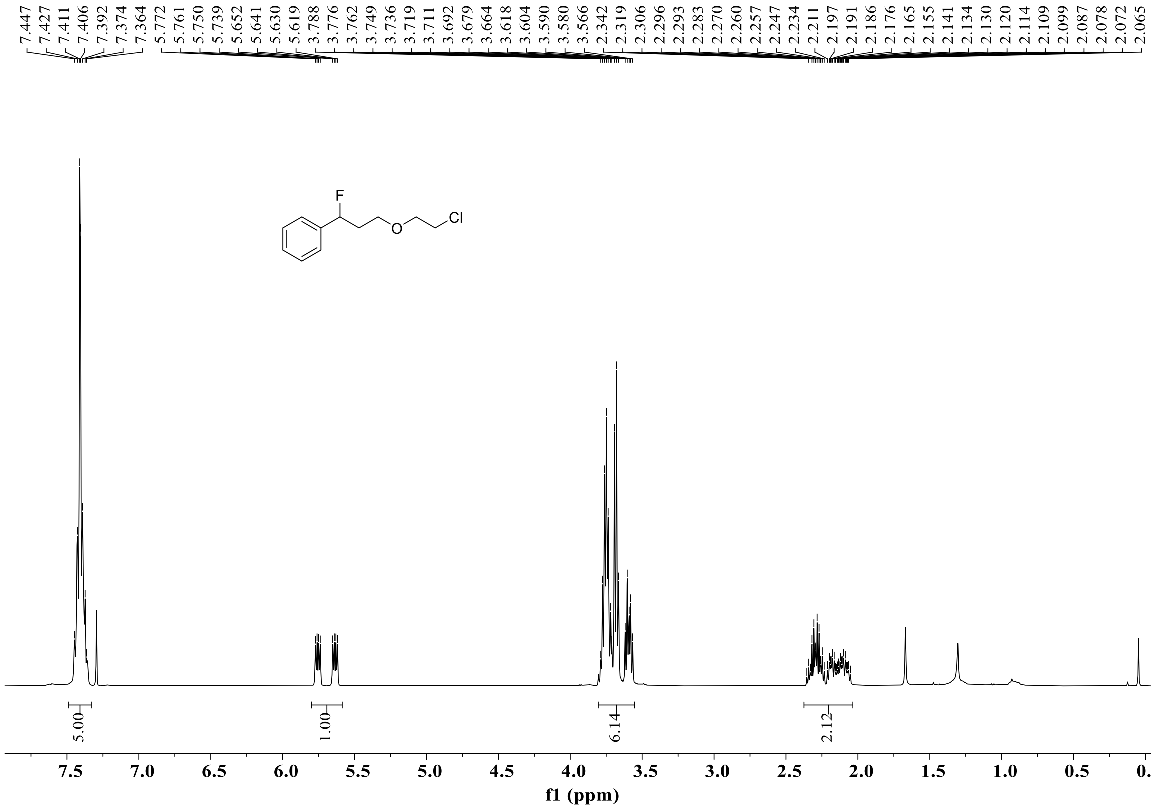

Supplementary Figure 128 ^{1}H NMR spectra of compound **45**

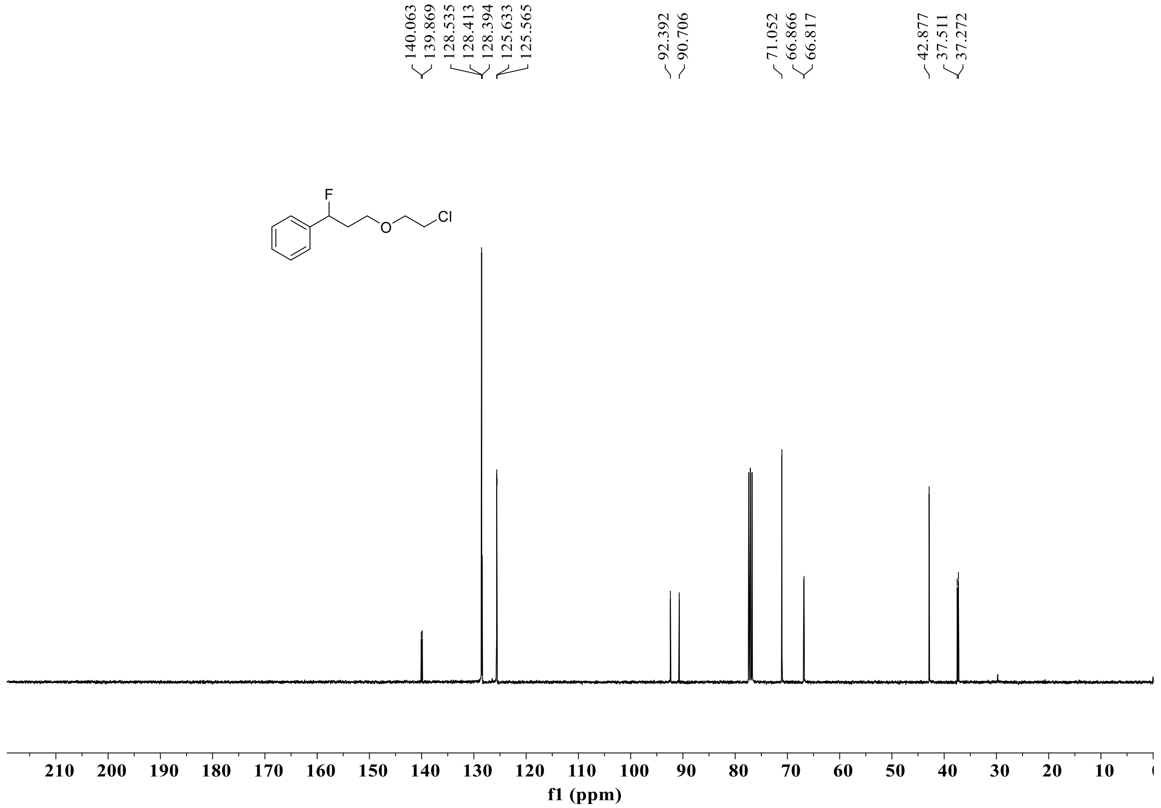

Supplementary Figure 129 ^{13}C NMR spectra of compound **45**

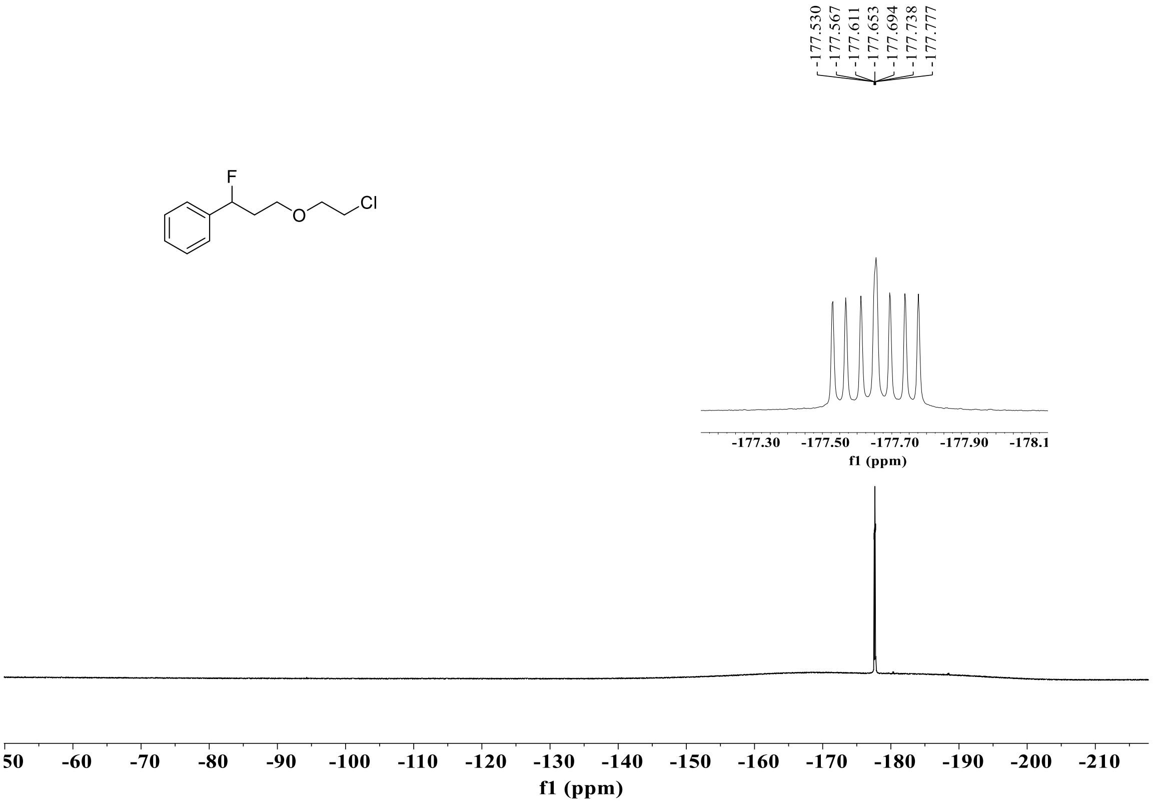

Supplementary Figure 130 ^{19}F NMR spectra of compound **45**

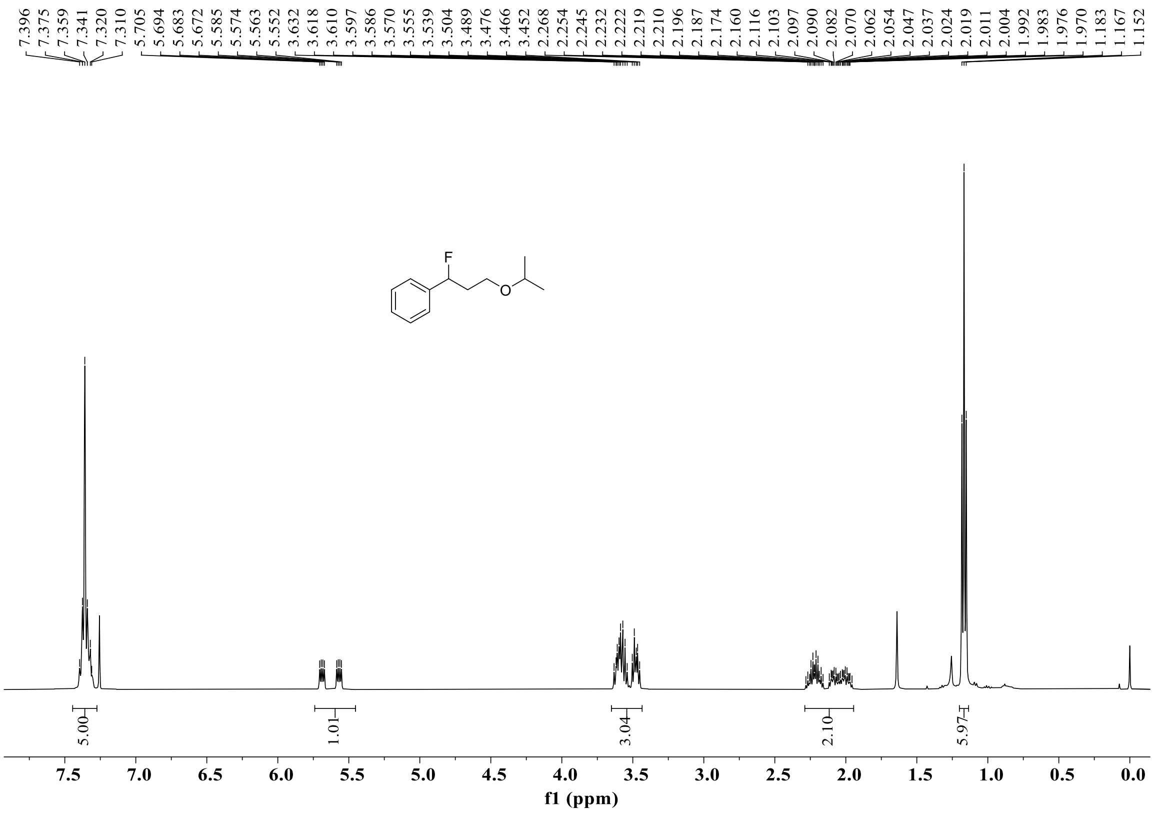

Supplementary Figure 131 ^{1}H NMR spectra of compound **46**

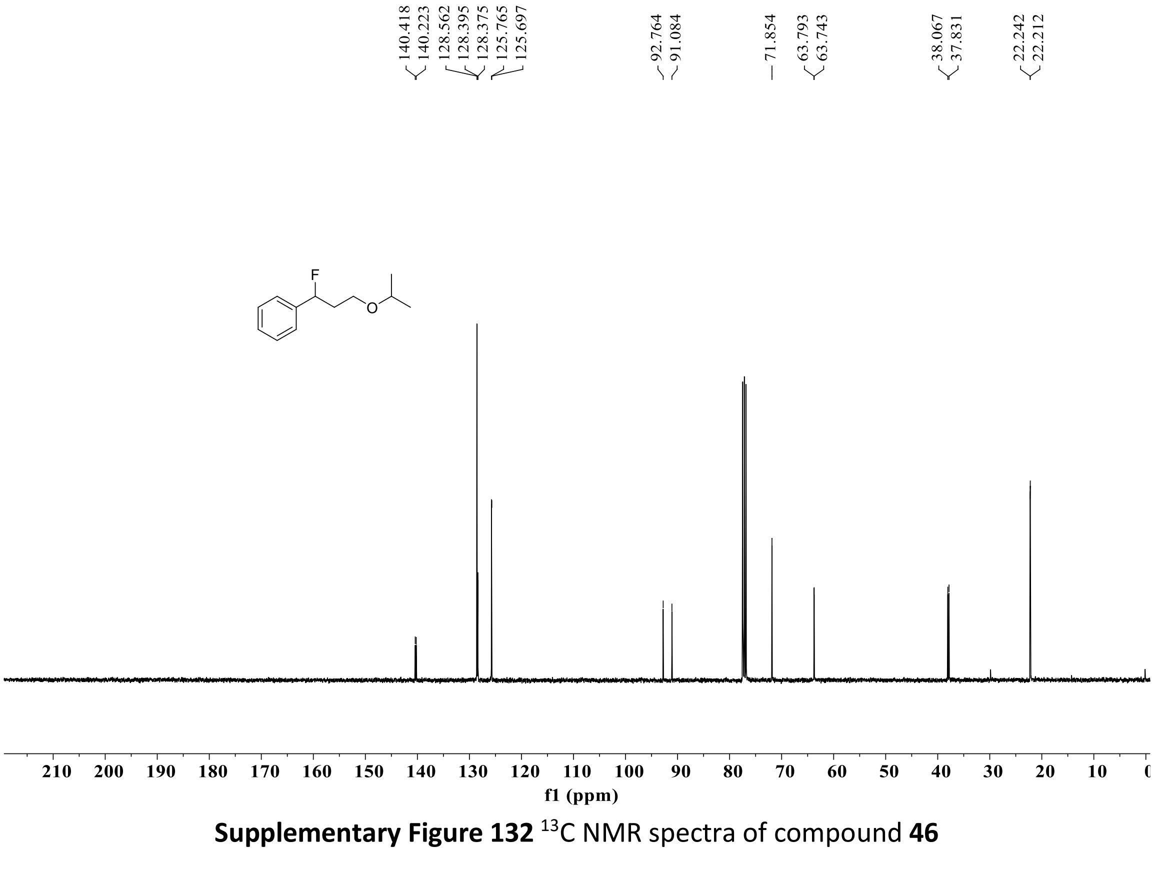

Supplementary Figure 132 ^{13}C NMR spectra of compound **46**

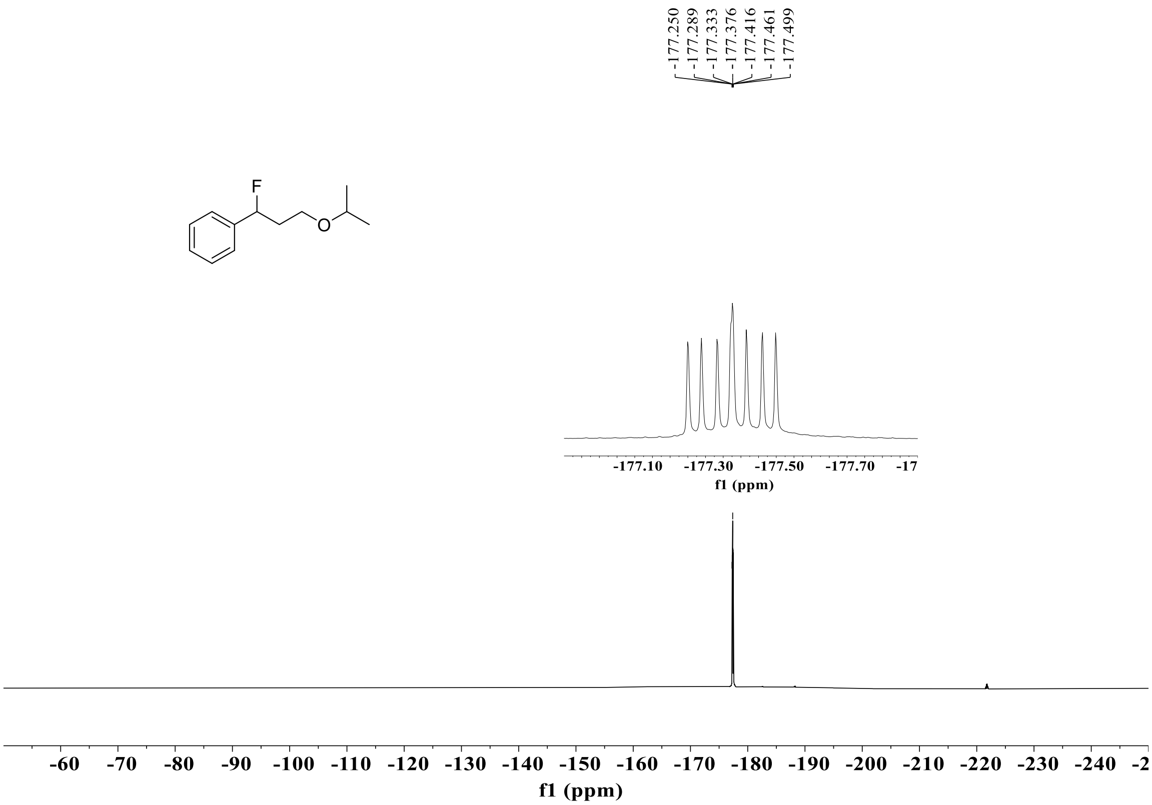

Supplementary Figure 133 ^{19}F NMR spectra of compound **46**

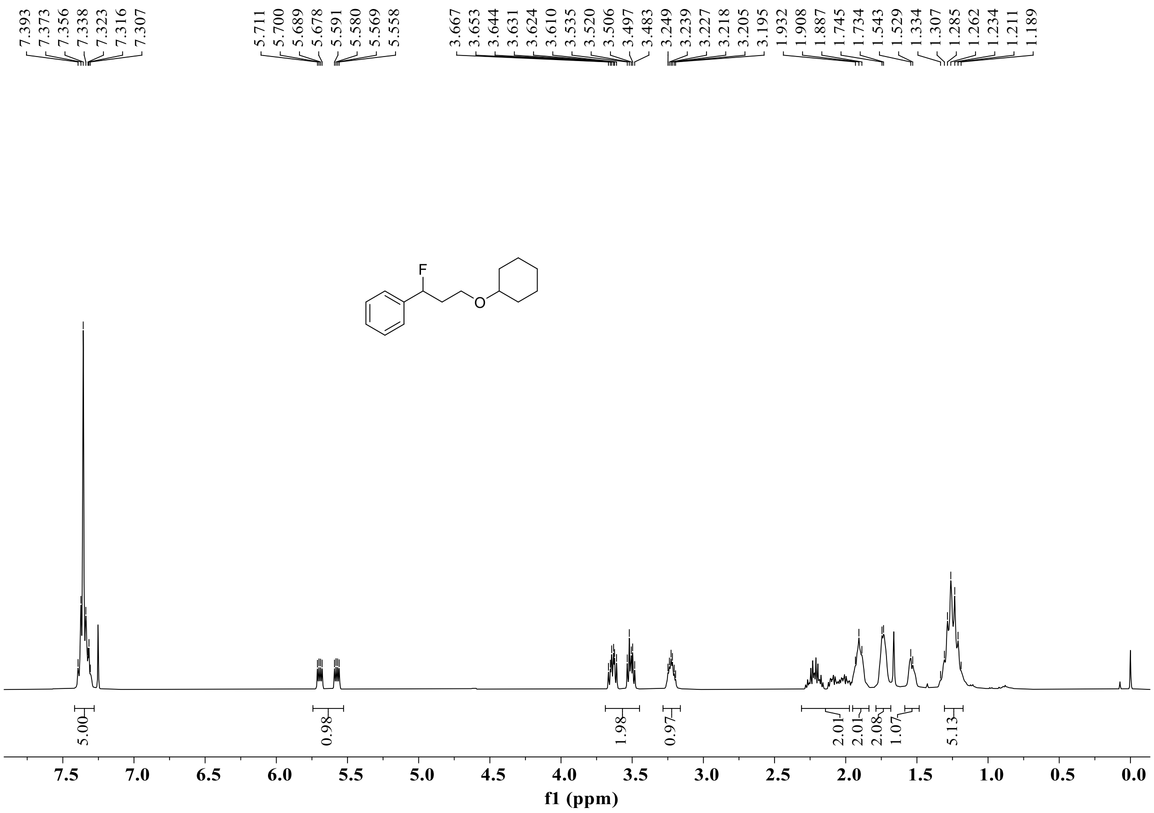

Supplementary Figure 134 ^{1}H NMR spectra of compound **47**

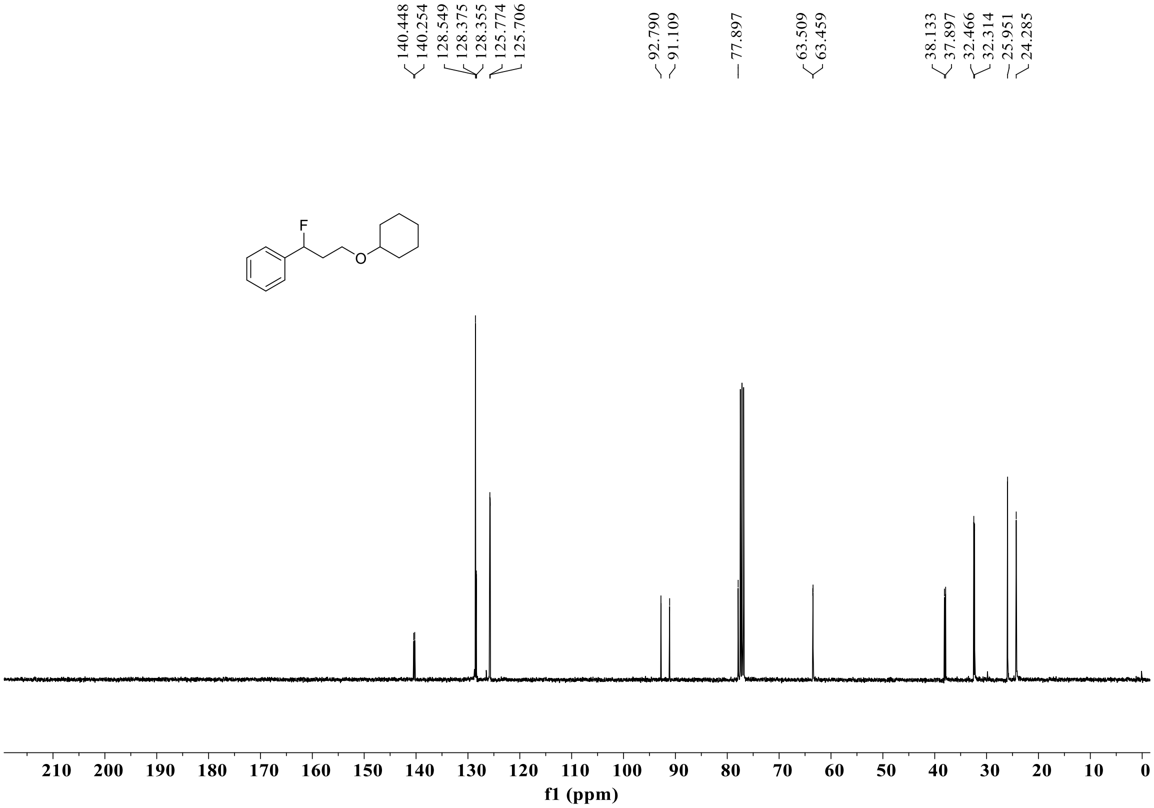

Supplementary Figure 135 ^{13}C NMR spectra of compound **47**

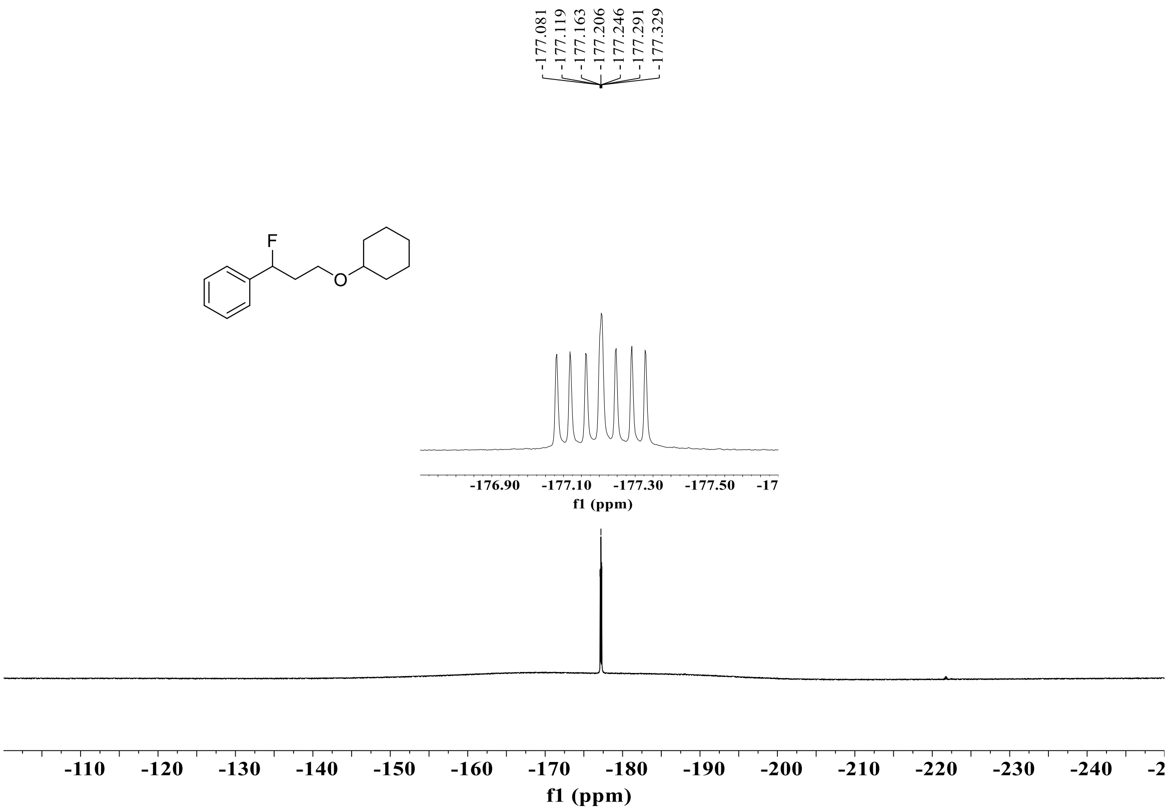

Supplementary Figure 136 ^{19}F NMR spectra of compound **47**

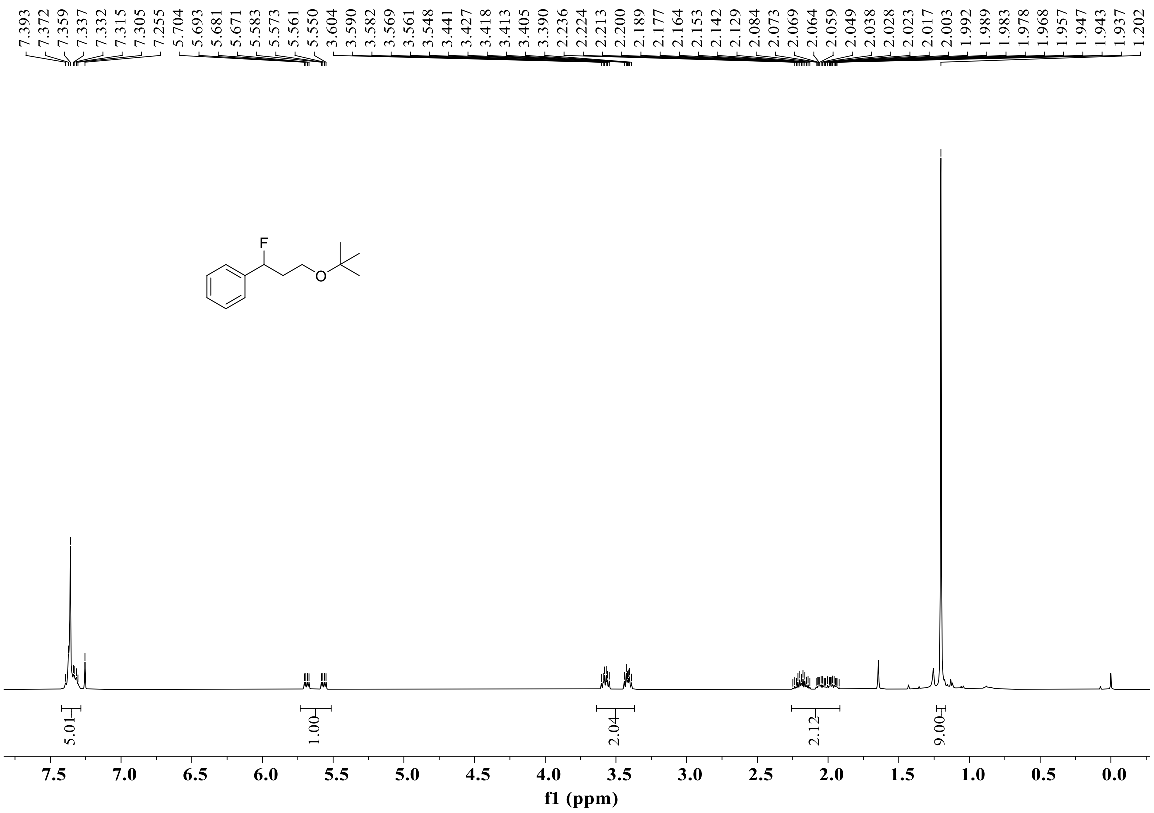

Supplementary Figure 137 ^{1}H NMR spectra of compound **48**

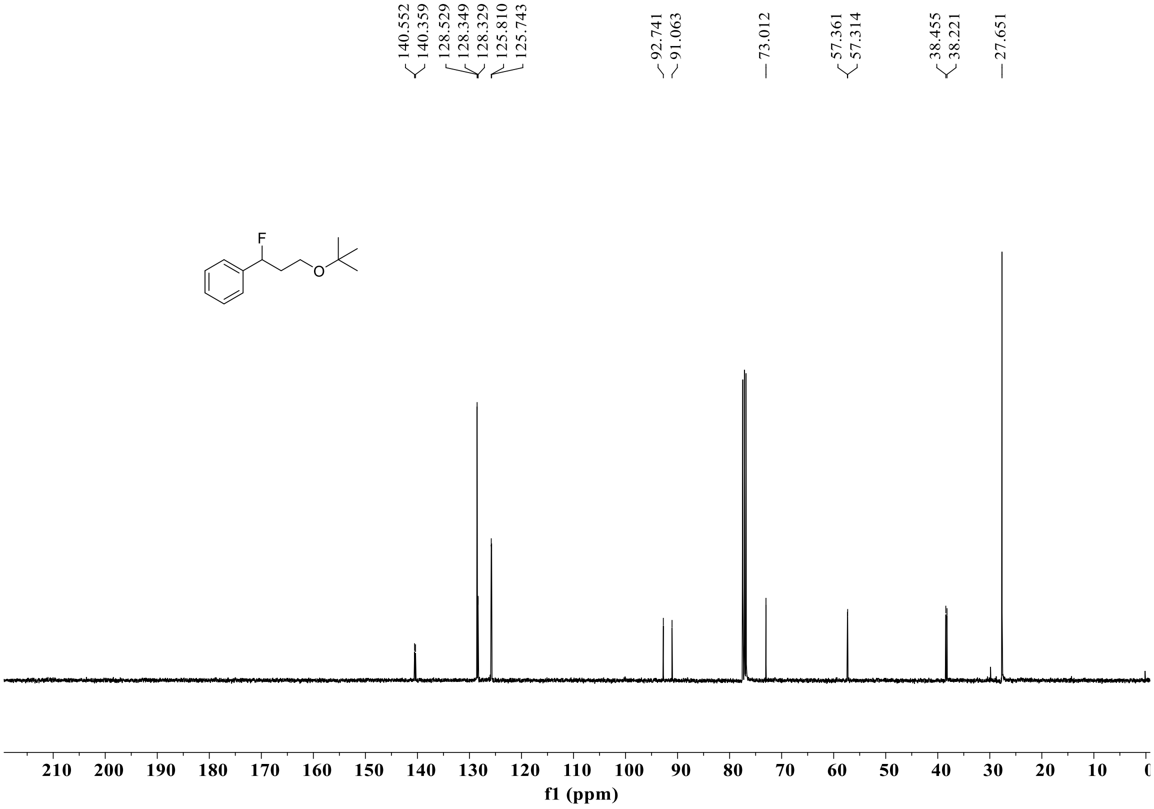

Supplementary Figure 138 ^{13}C NMR spectra of compound **48**

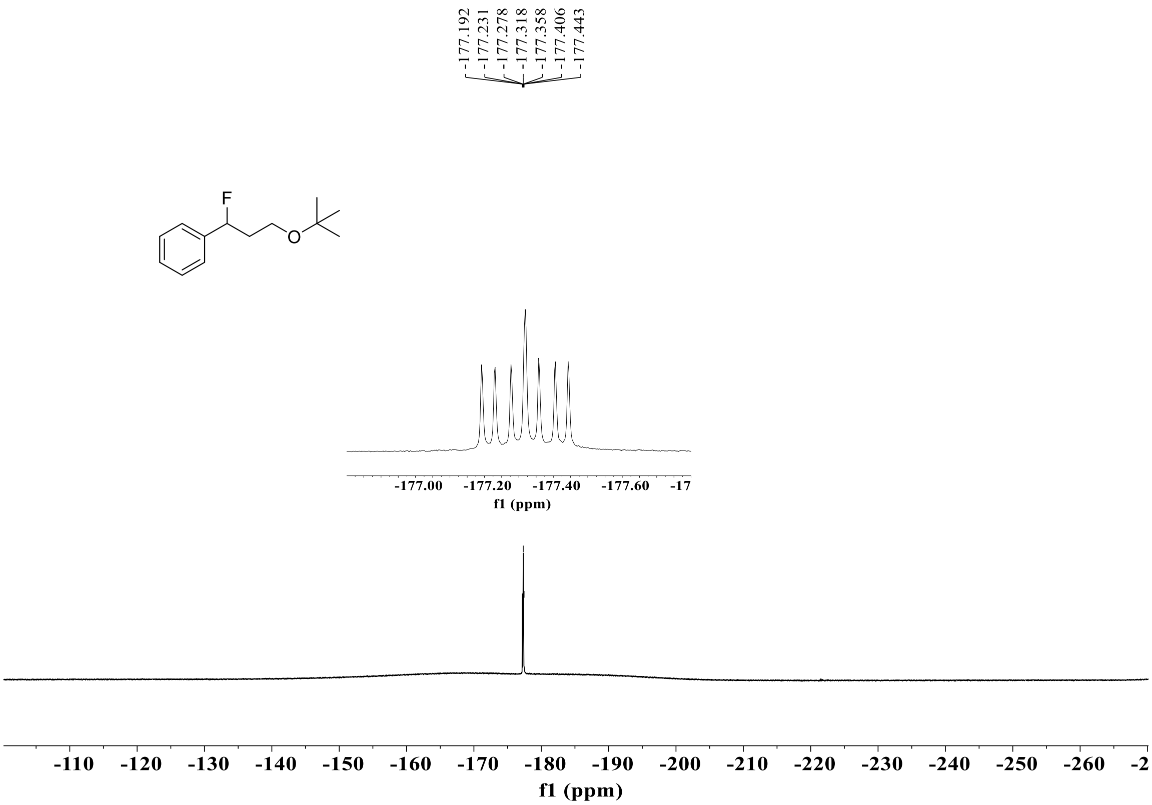

Supplementary Figure 139 ^{19}F NMR spectra of compound **48**

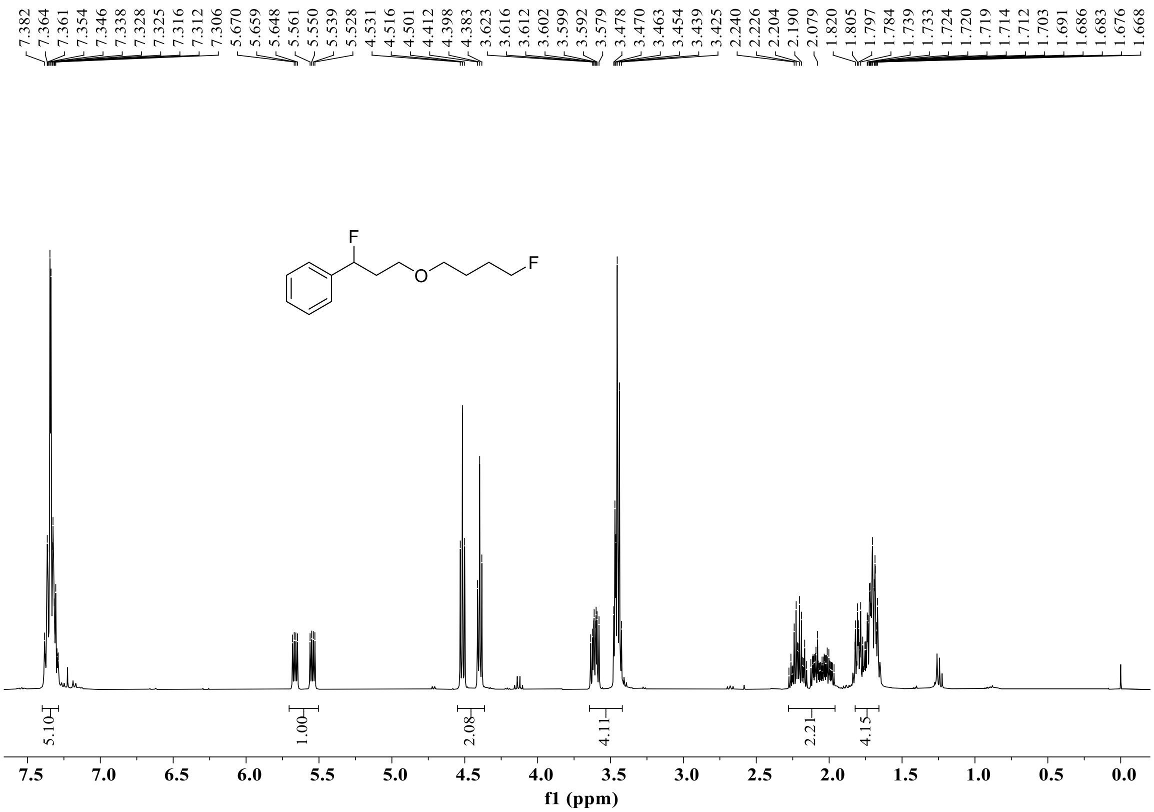

Supplementary Figure 140 ^{1}H NMR spectra of compound **49**

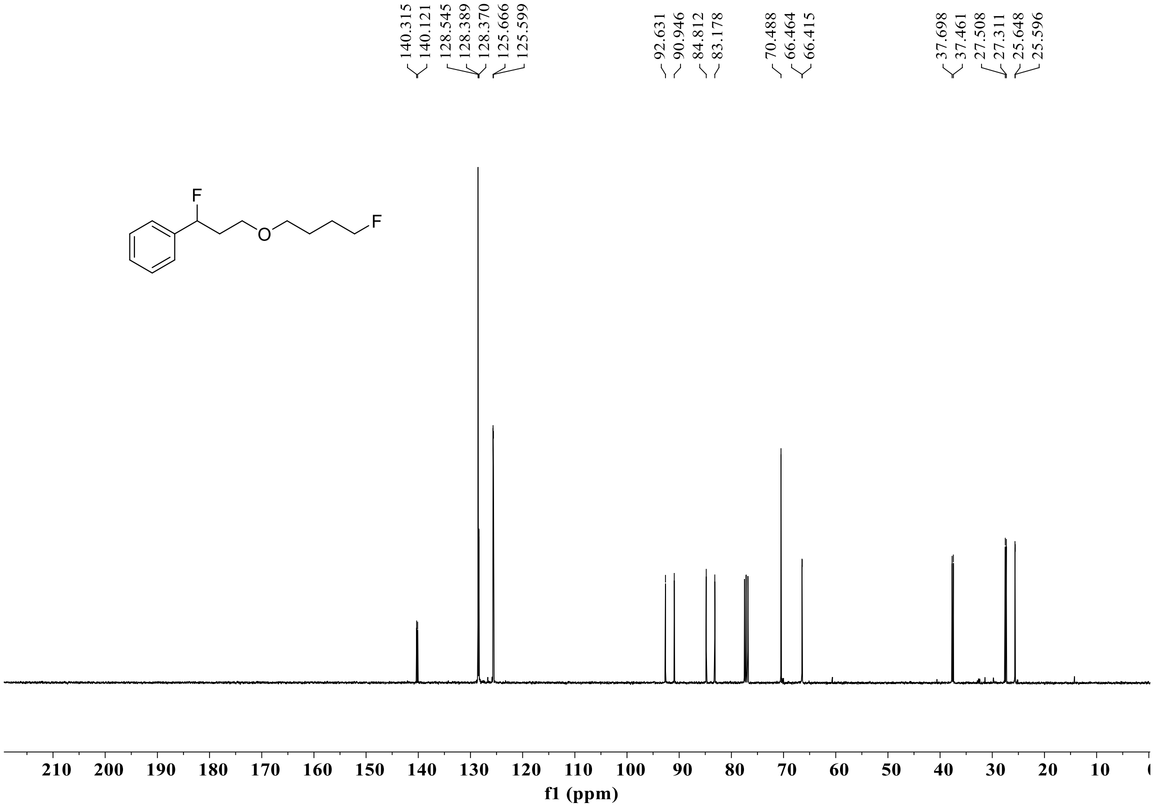

Supplementary Figure 141 ^{13}C NMR spectra of compound **49**

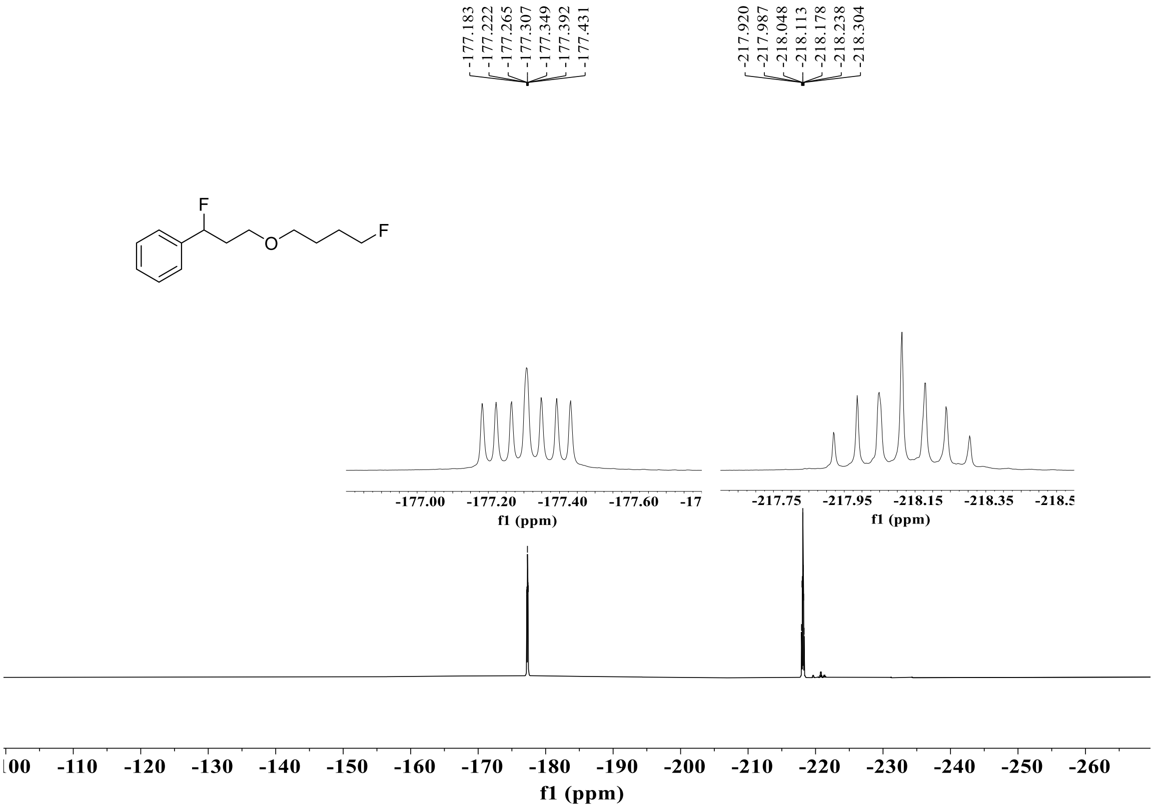

Supplementary Figure 142 ^{19}F NMR spectra of compound **49**

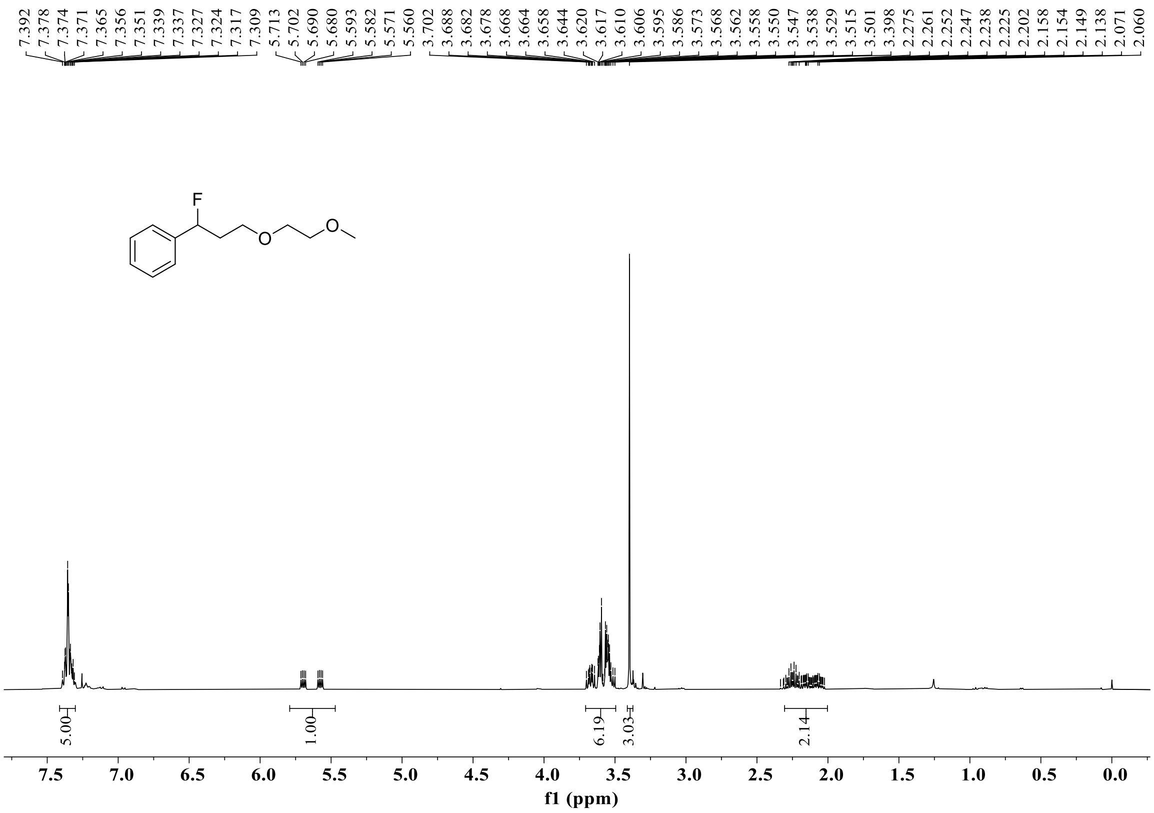

Supplementary Figure 143 ^{1}H NMR spectra of compound **50**

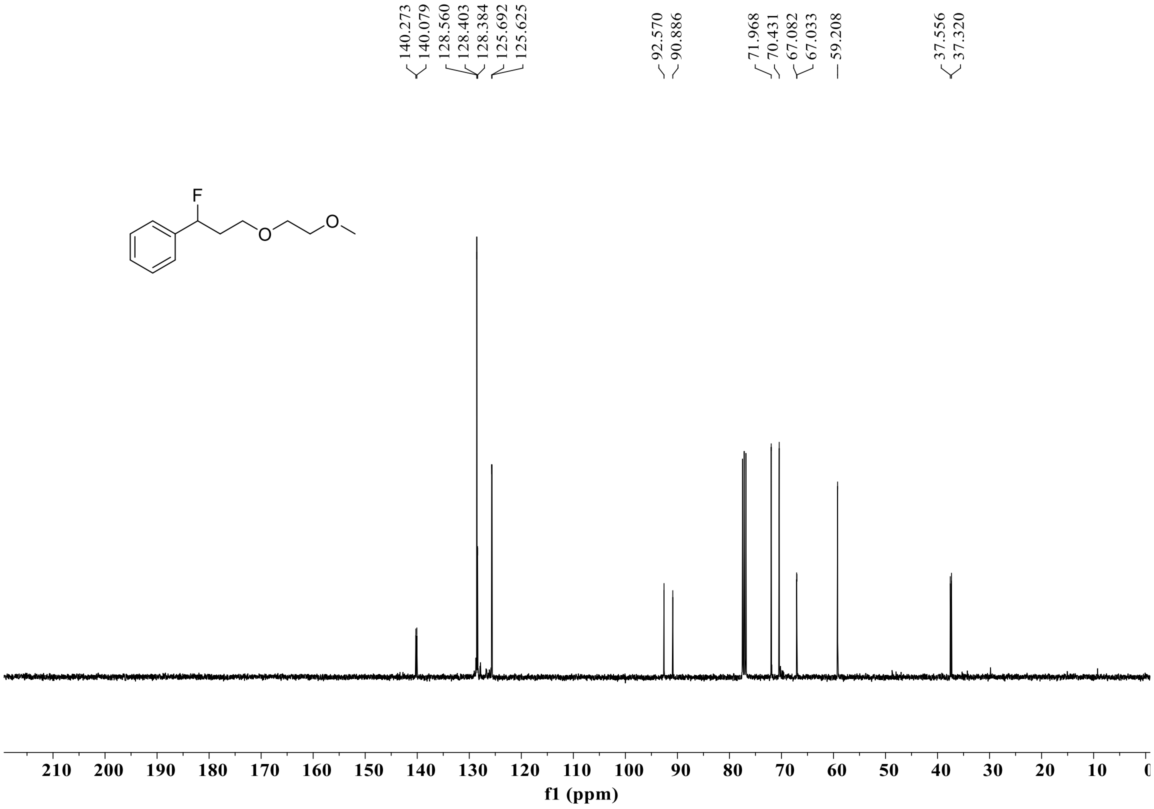

Supplementary Figure 144 ^{13}C NMR spectra of compound **50**

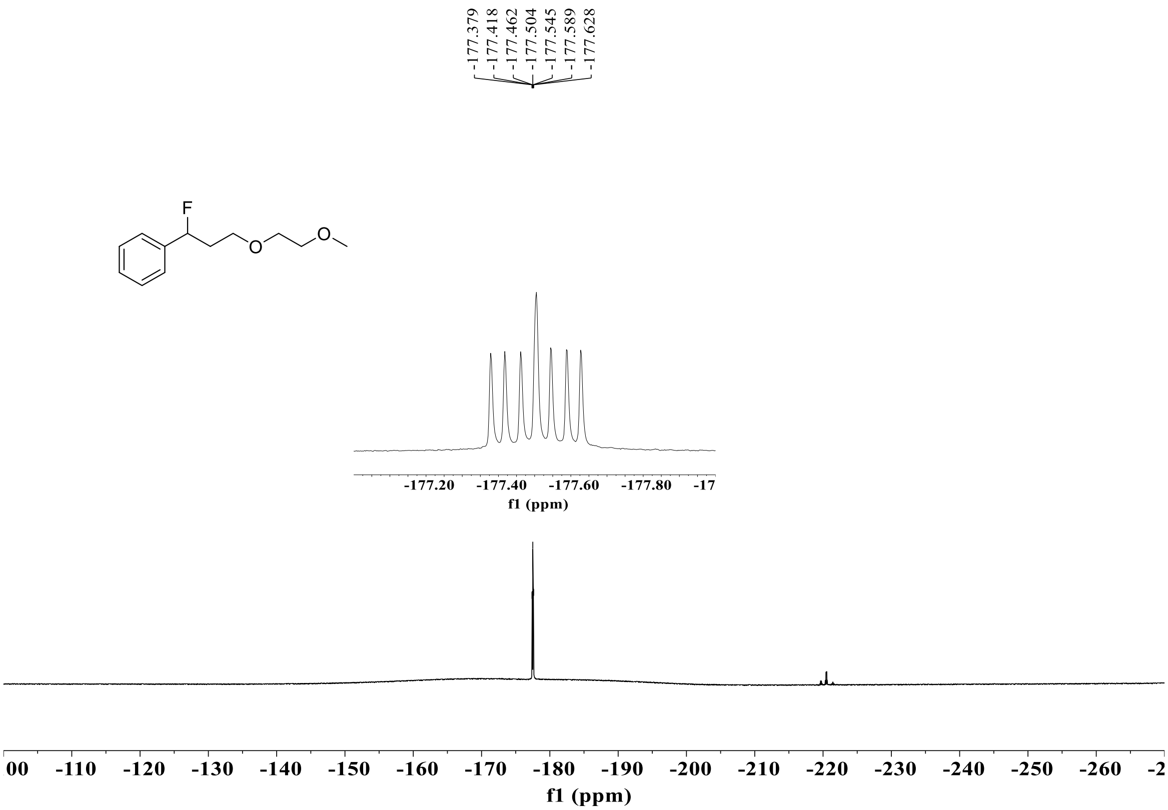

Supplementary Figure 145 ^{19}F NMR spectra of compound **50**

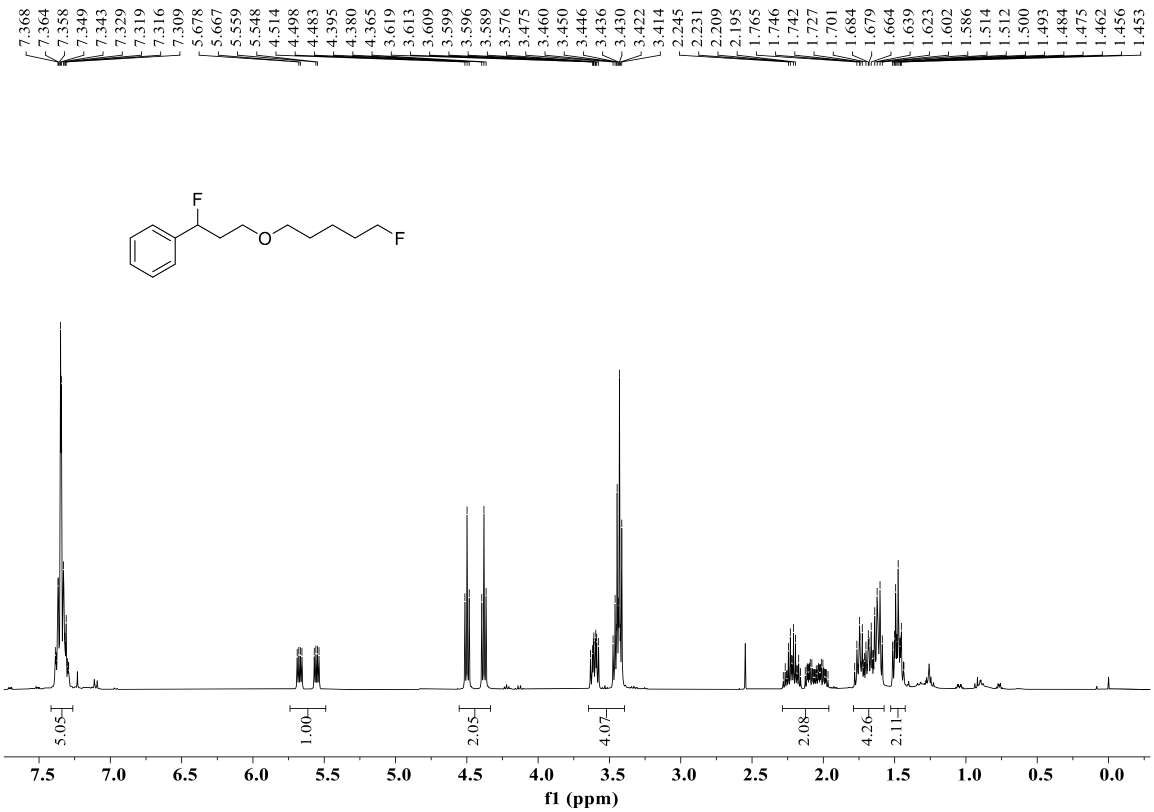

Supplementary Figure 146 ^{1}H NMR spectra of compound **51**

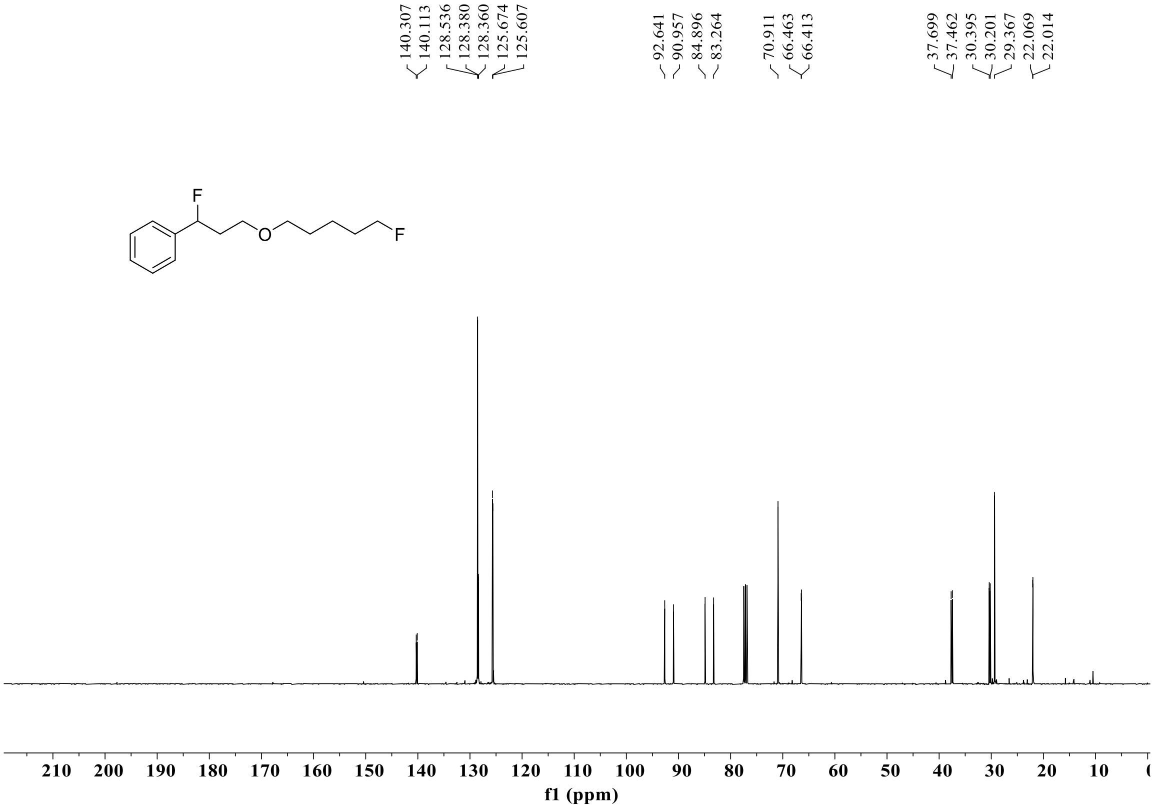

Supplementary Figure 147 ^{13}C NMR spectra of compound **51**

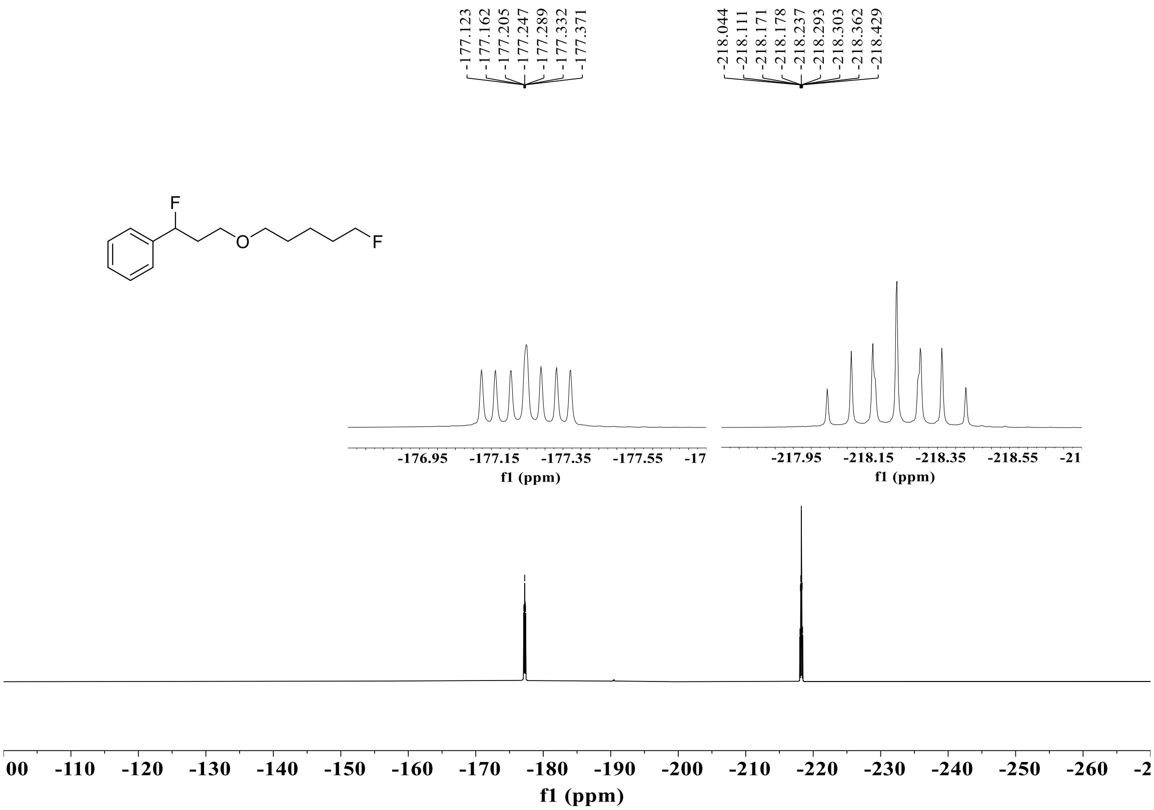

Supplementary Figure 147 ^{19}F NMR spectra of compound **51**

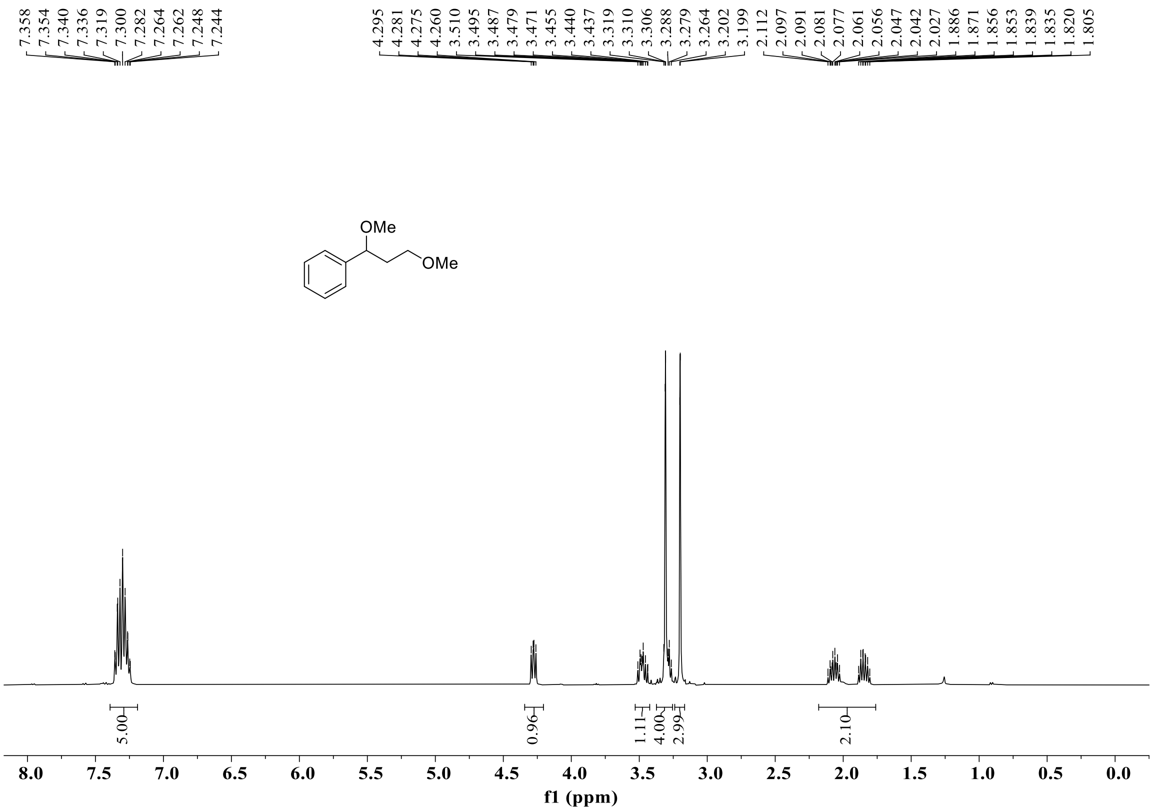

Supplementary Figure 148 ^{1}H NMR spectra of compound **5**

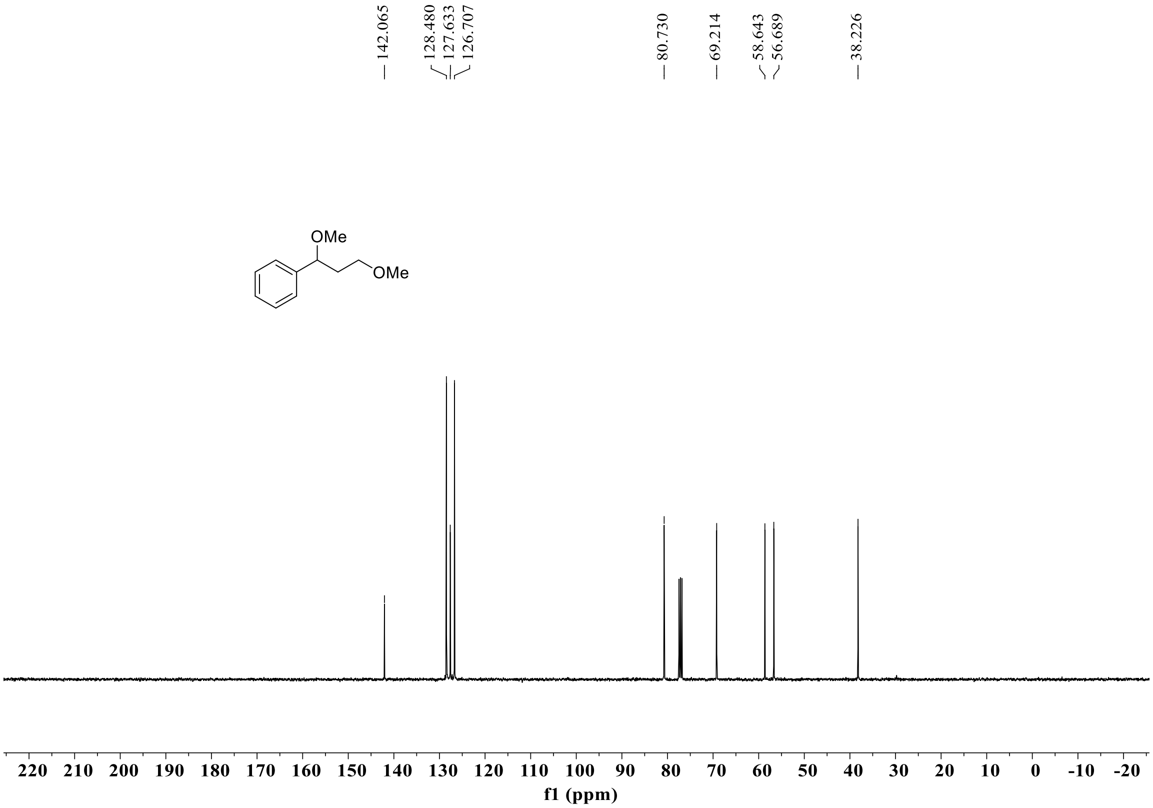

Supplementary Figure 149 ^{13}C NMR spectra of compound **5**

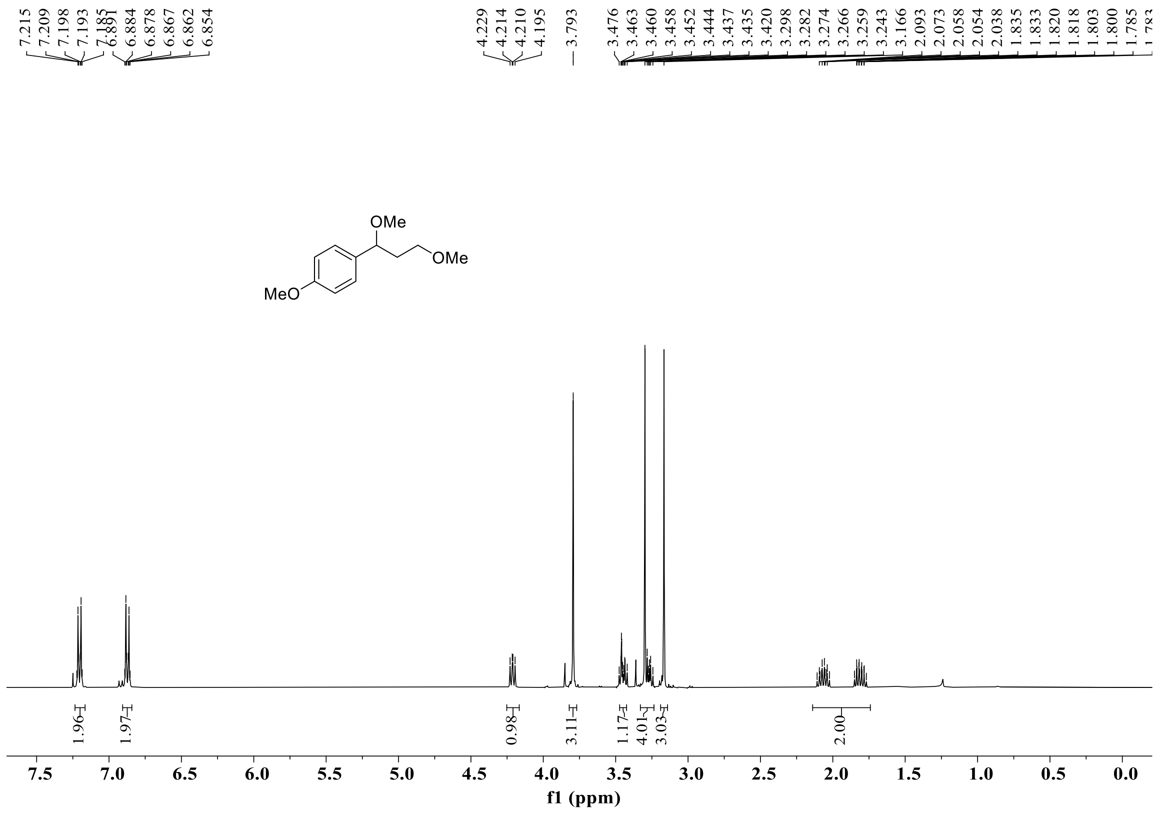

Supplementary Figure 150 ^{1}H NMR spectra of compound **52**

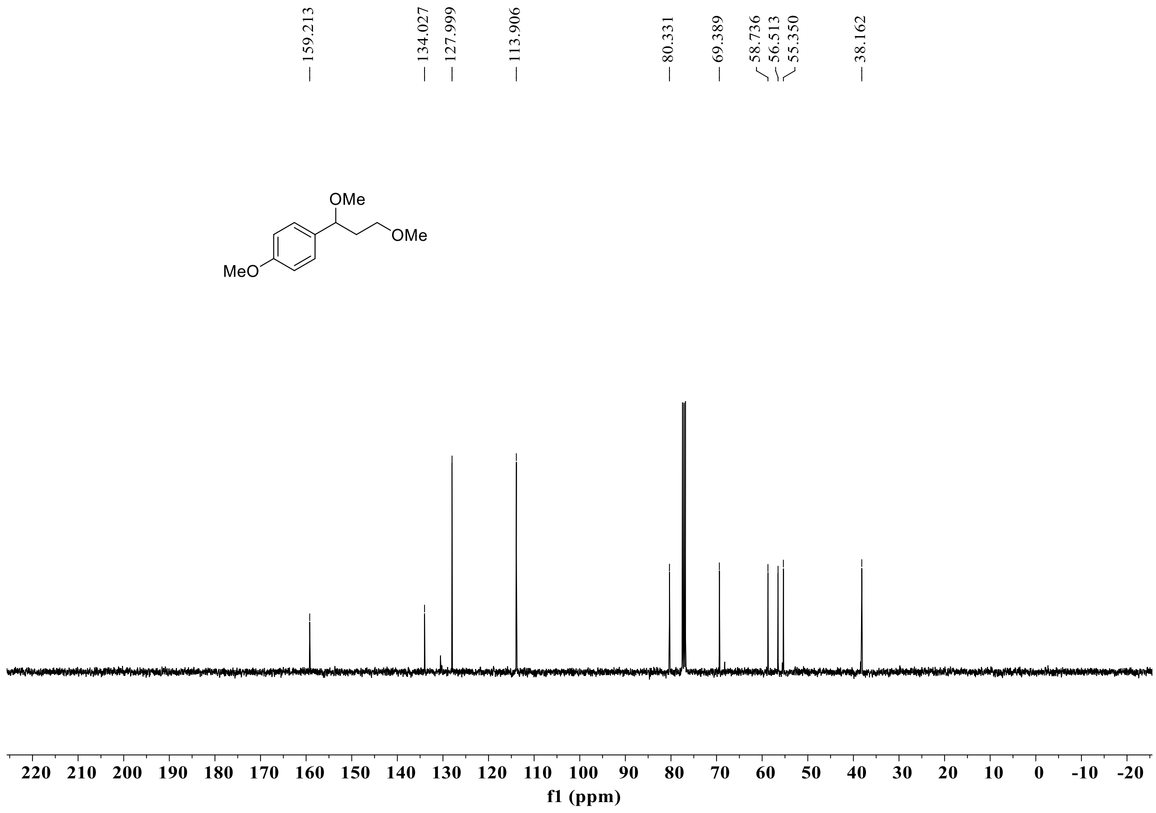

Supplementary Figure 151 ^{13}C NMR spectra of compound **52**

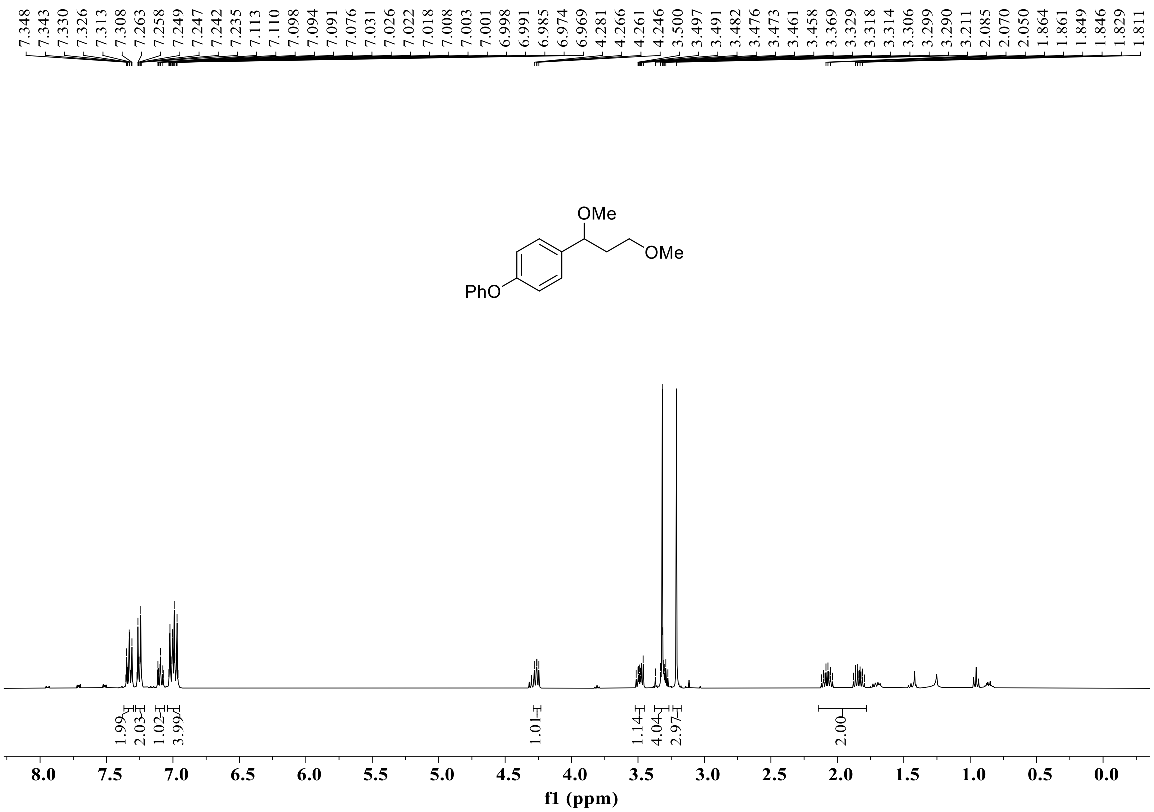

Supplementary Figure 152 ^{1}H NMR spectra of compound **53**

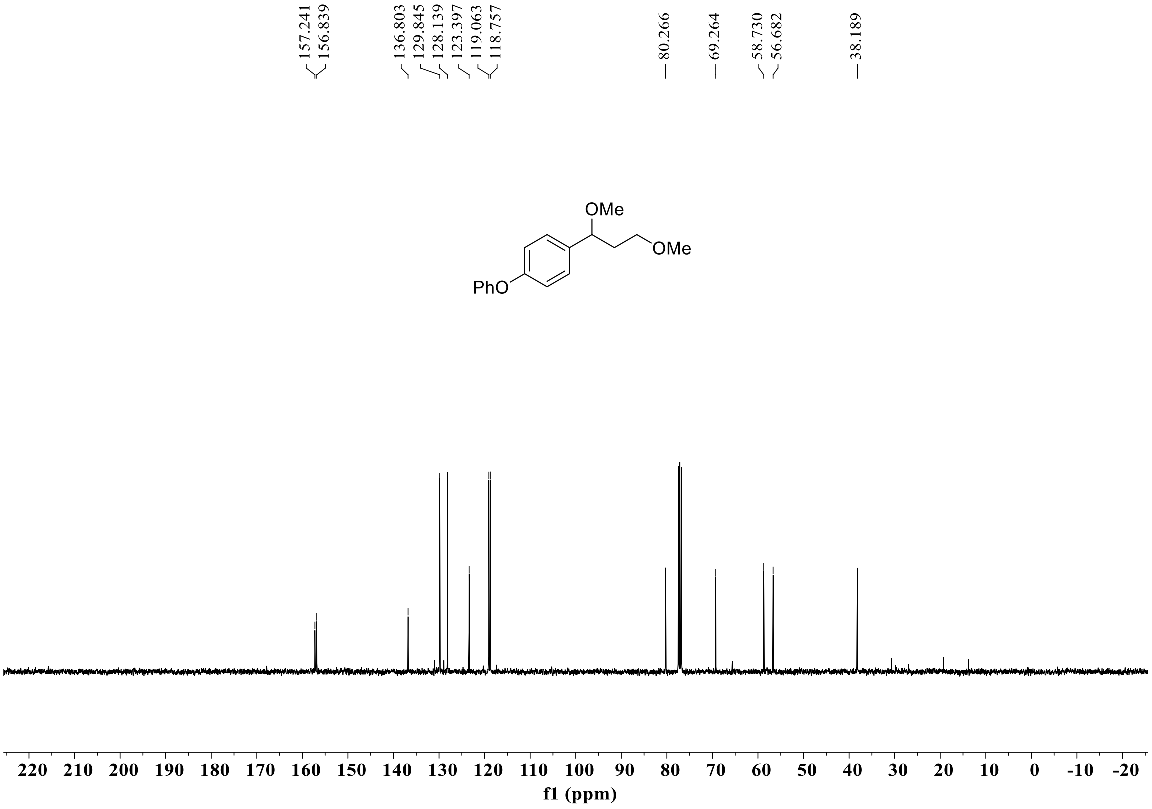

Supplementary Figure 153 ^{13}C NMR spectra of compound **53**

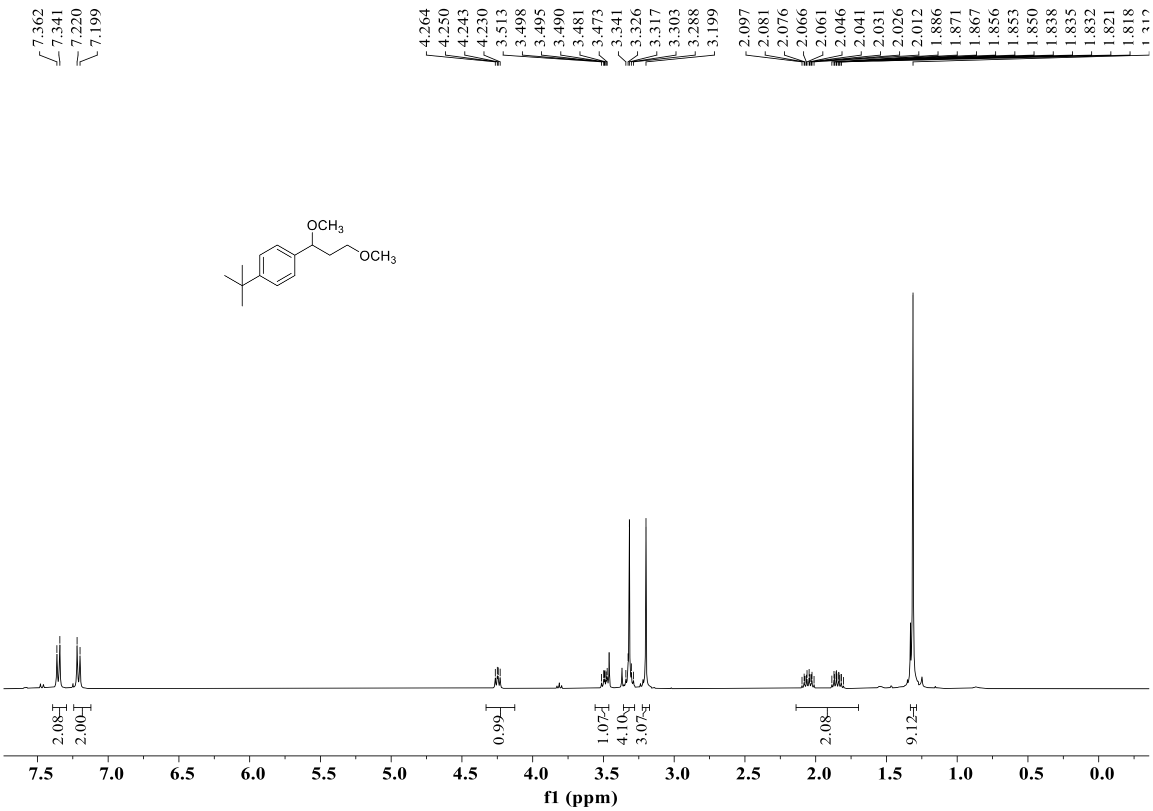

Supplementary Figure 154 ^{1}H NMR spectra of compound **54**

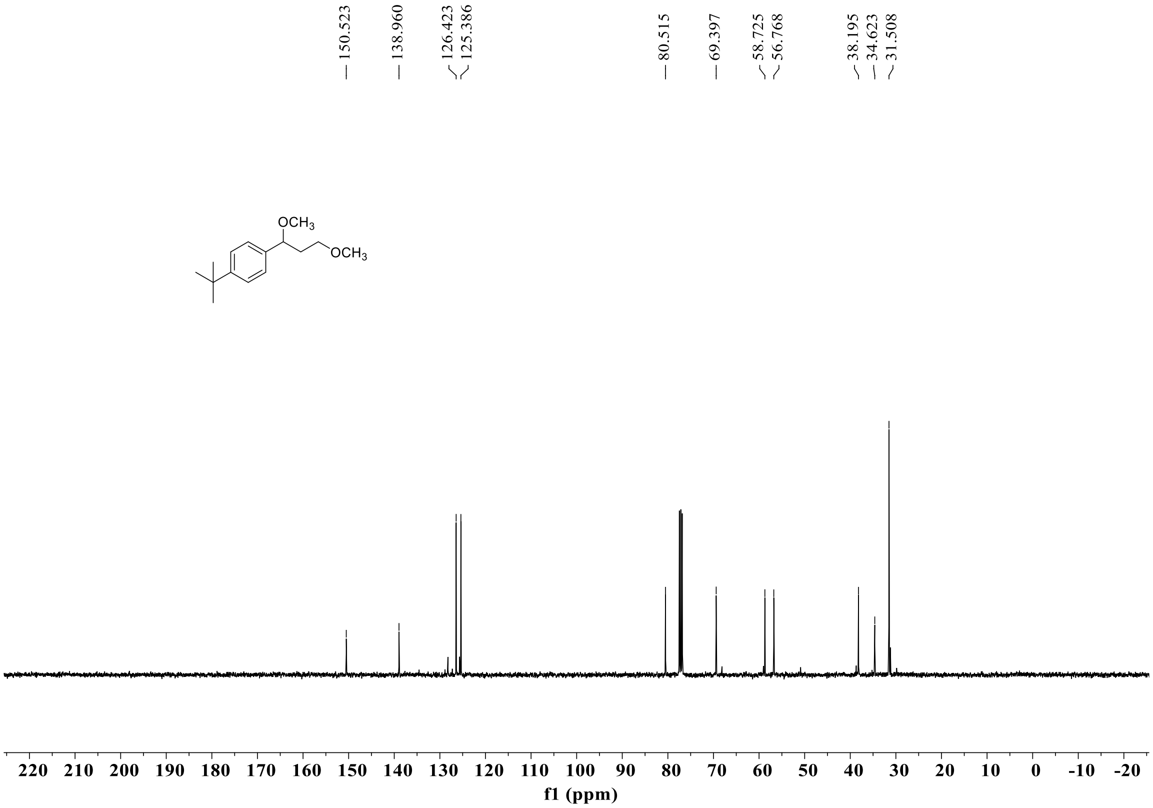

Supplementary Figure 155 ^{13}C NMR spectra of compound **54**

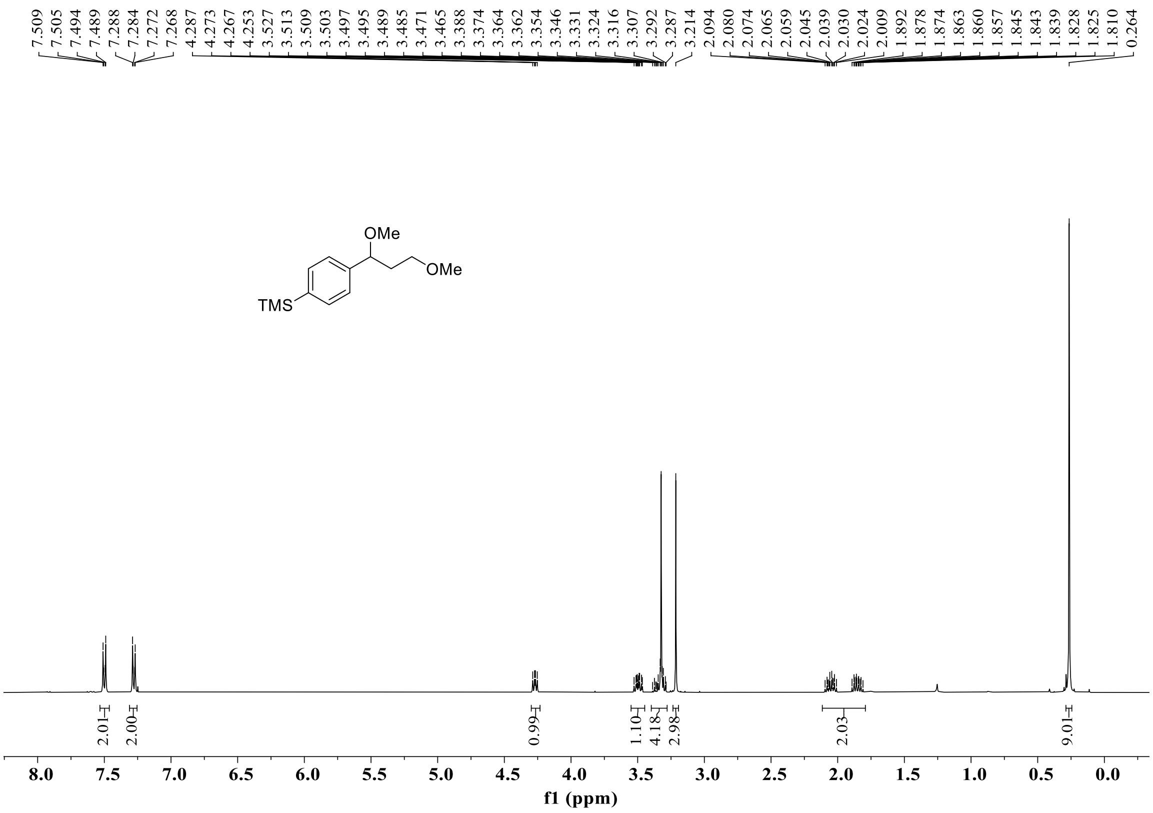

Supplementary Figure 156 ^{1}H NMR spectra of compound **55**

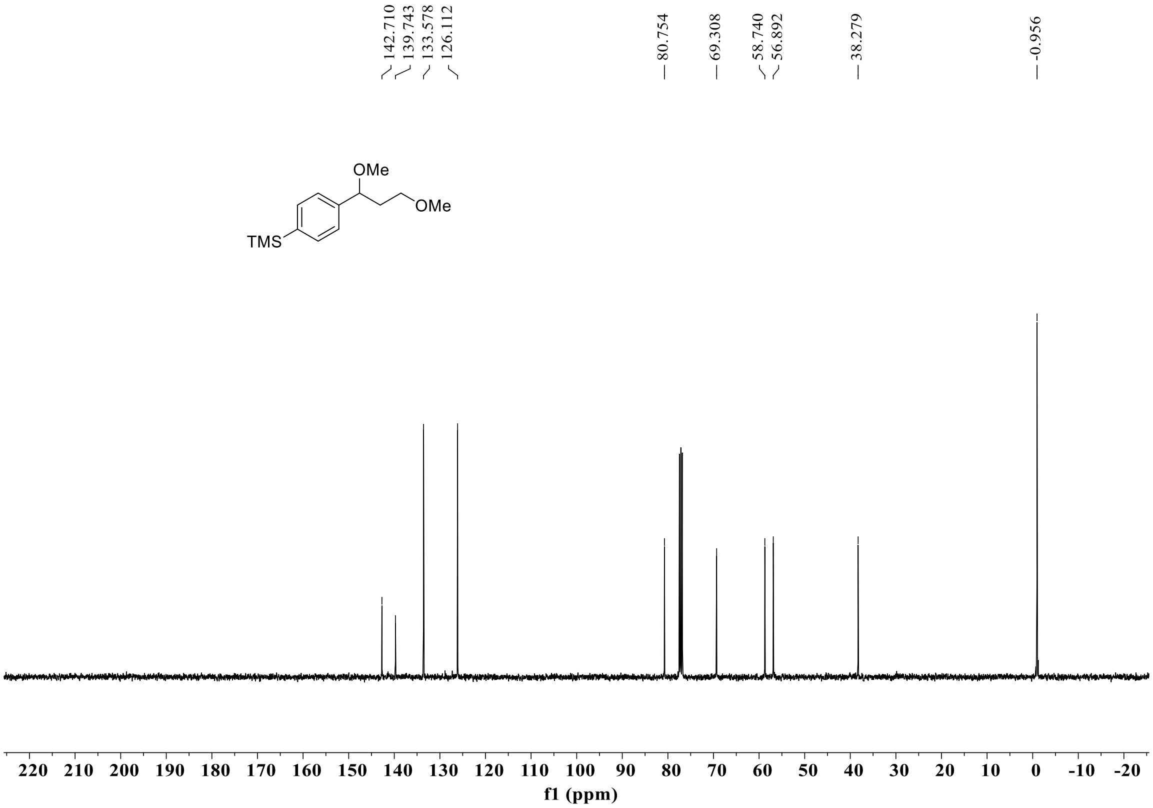

Supplementary Figure 157 ^{13}C NMR spectra of compound **55**

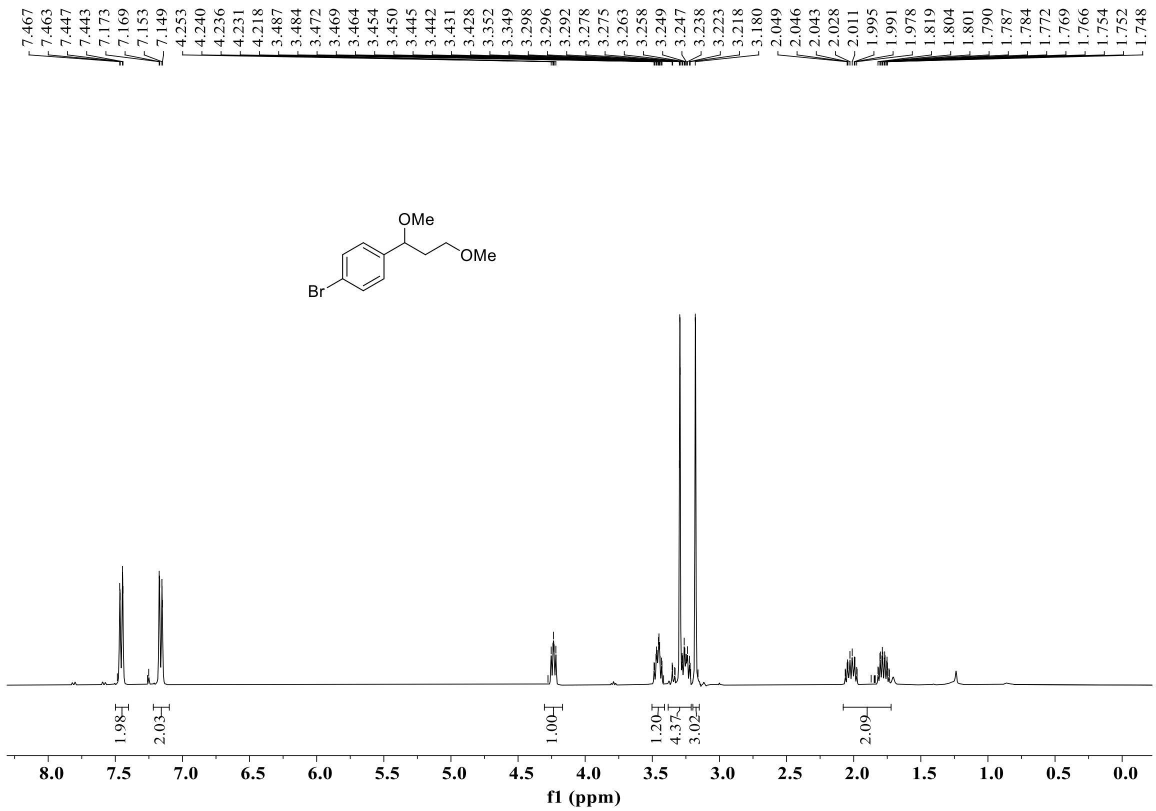

Supplementary Figure 158 ^{1}H NMR spectra of compound **56**

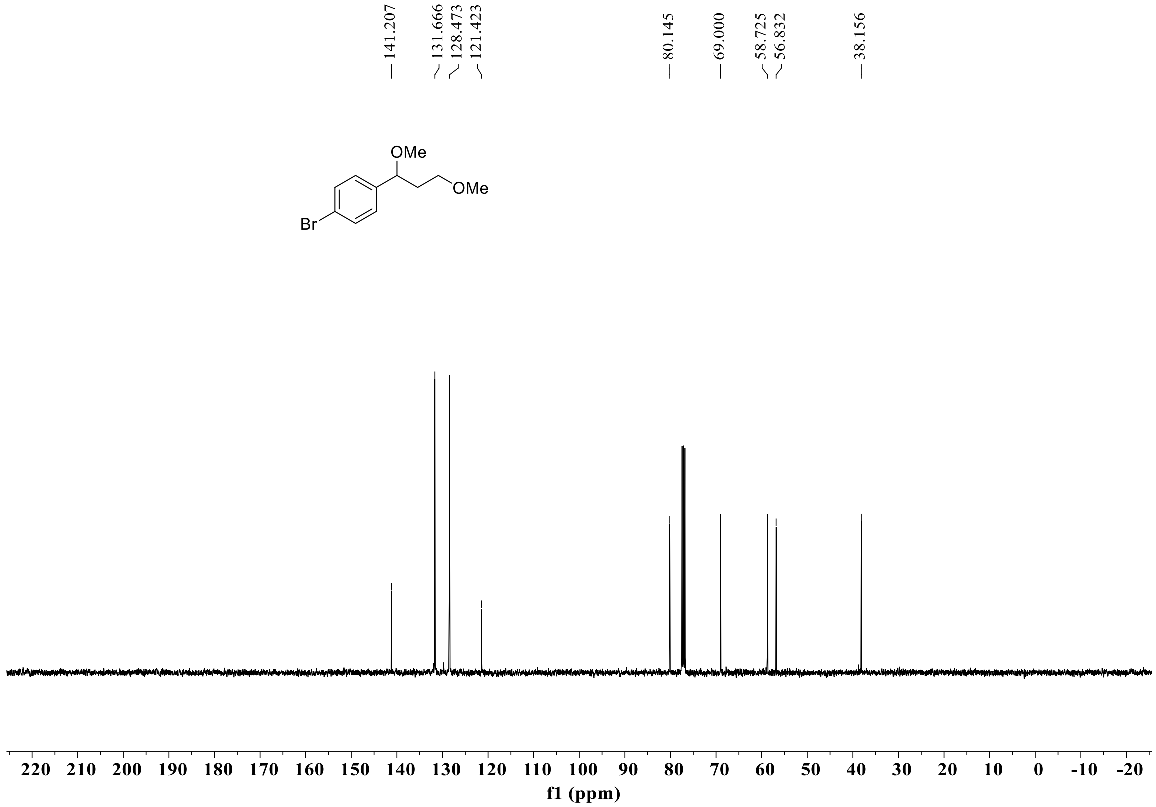

Supplementary Figure 159 ^{13}C NMR spectra of compound **56**

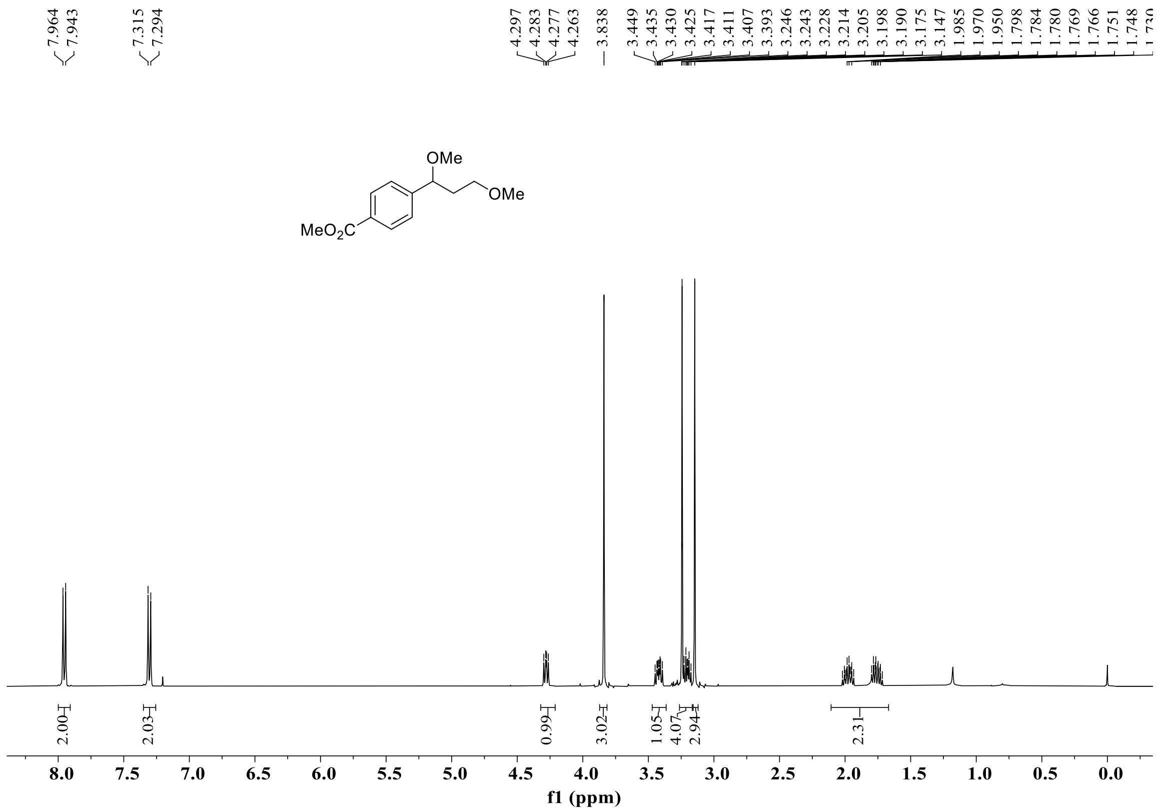

Supplementary Figure 160 ^{1}H NMR spectra of compound **57**

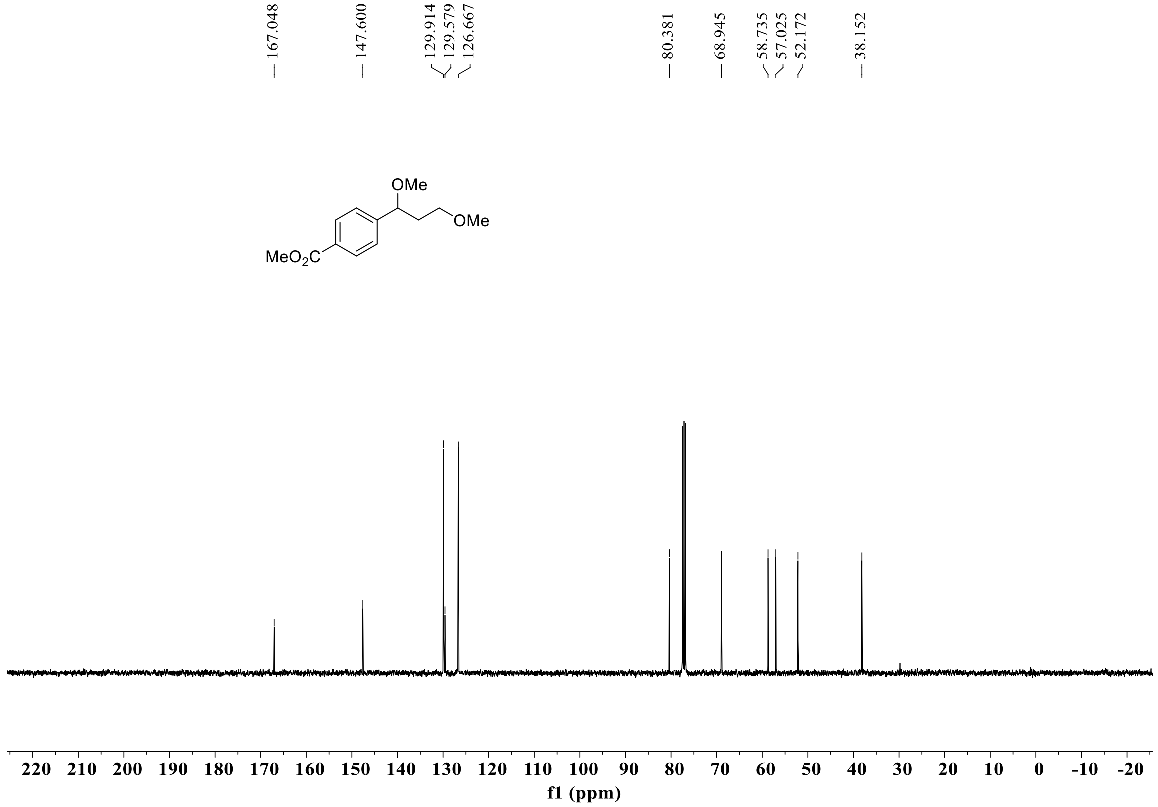

Supplementary Figure 161 ^{13}C NMR spectra of compound **57**

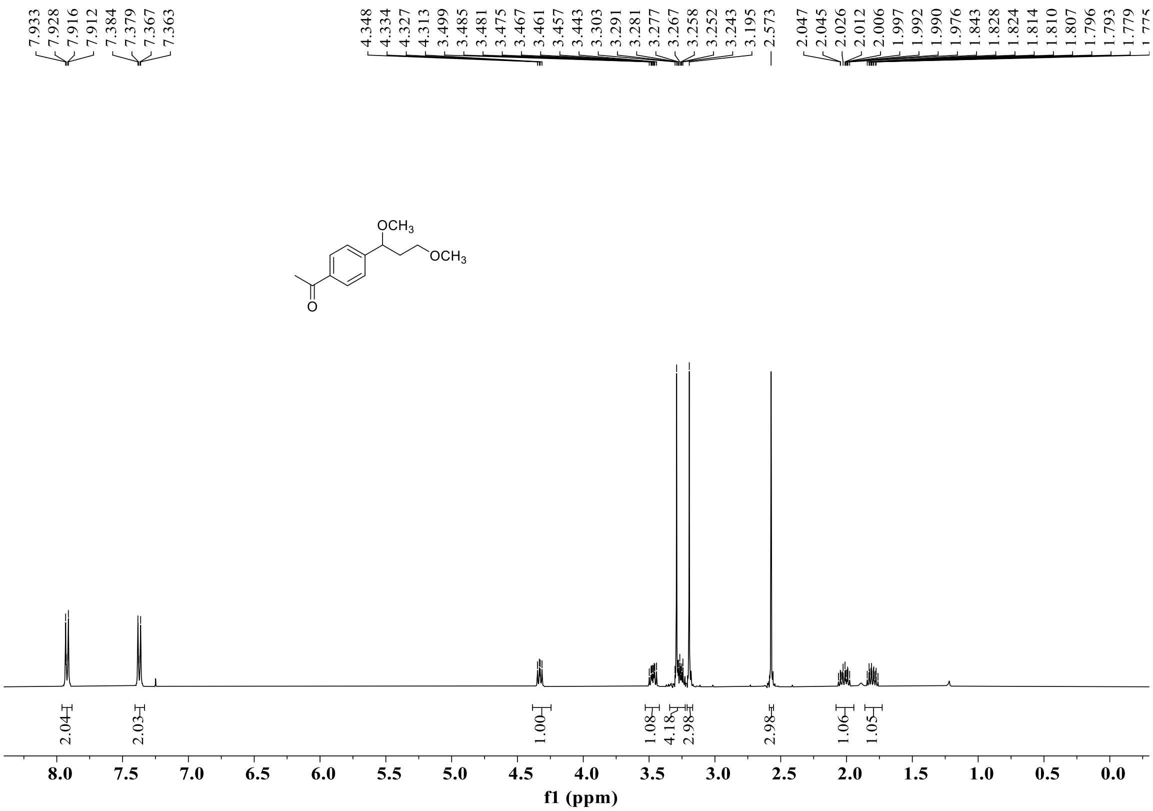

Supplementary Figure 162 ^{1}H NMR spectra of compound **58**

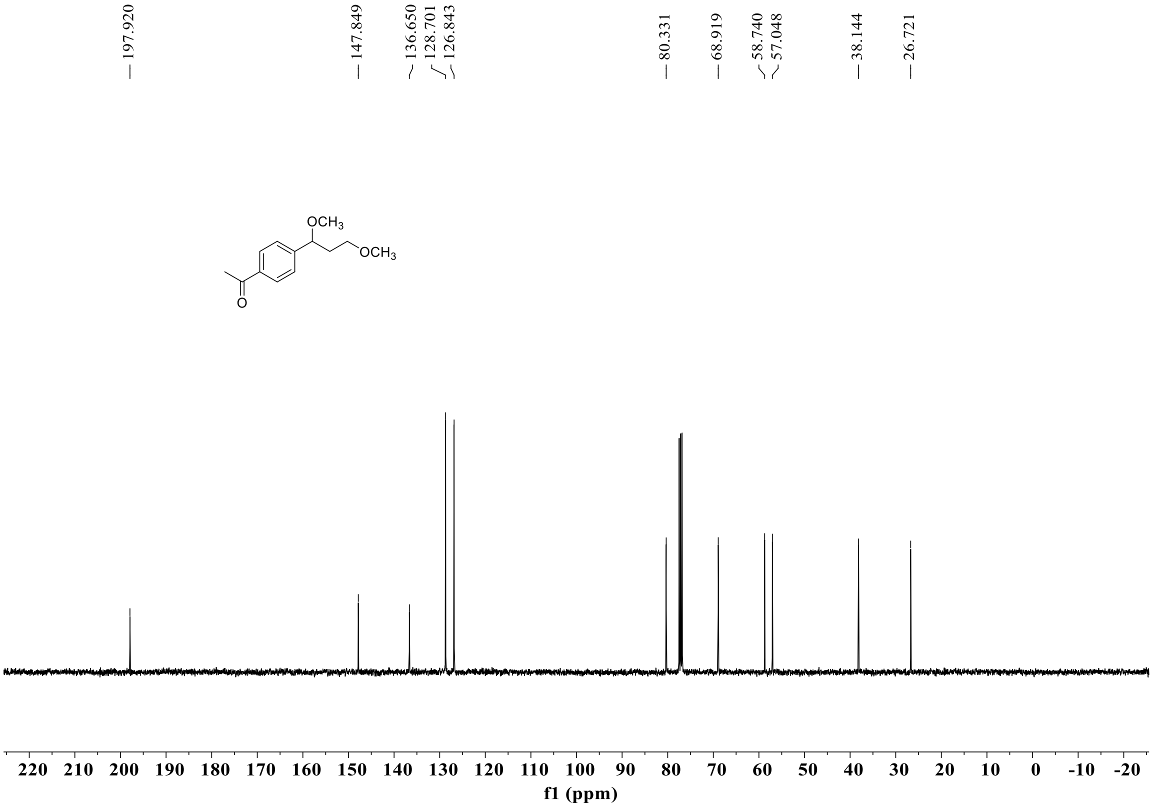

Supplementary Figure 163 ^{13}C NMR spectra of compound **58**

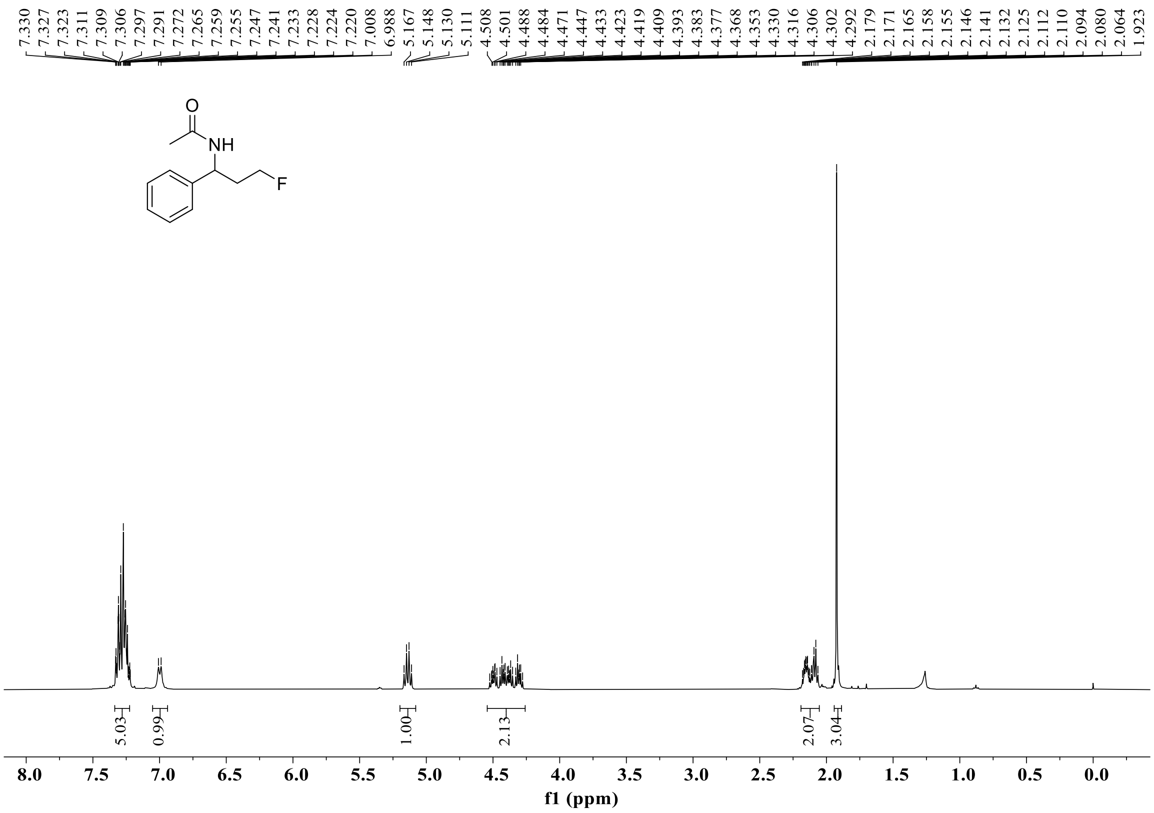

Supplementary Figure 164 ^{1}H NMR spectra of compound **59**

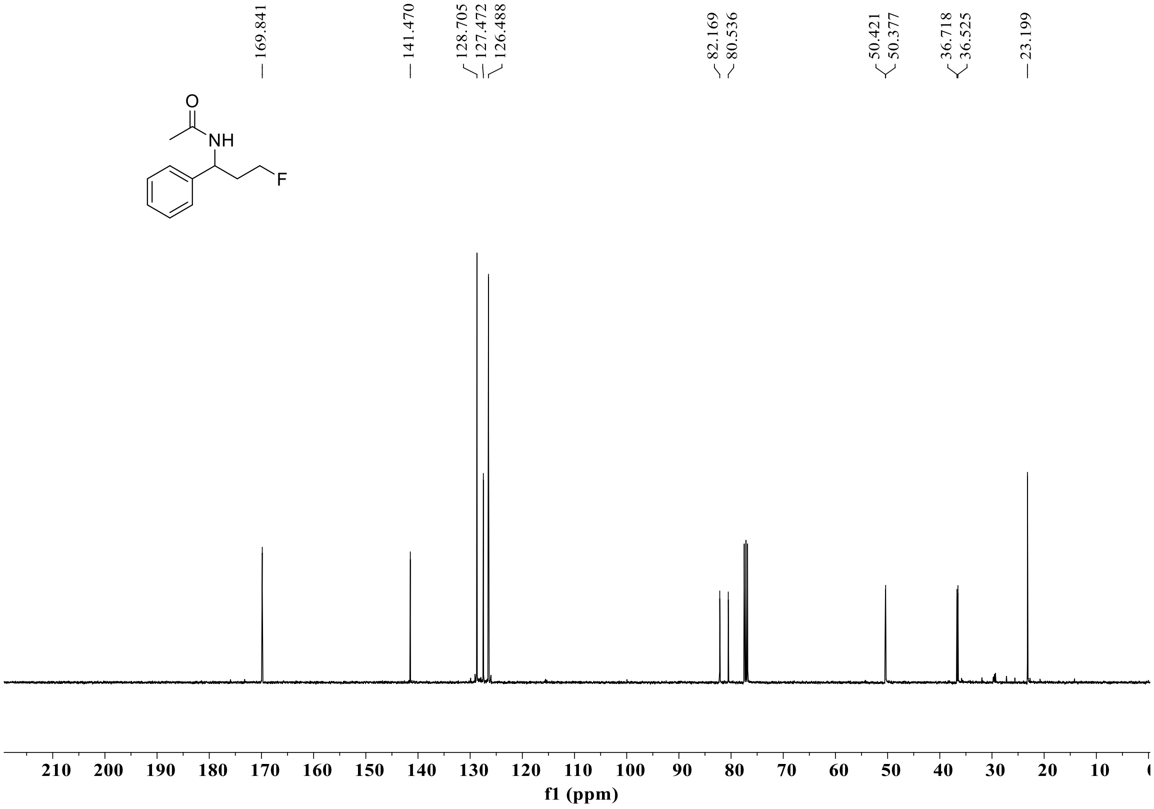

Supplementary Figure 165 ^{13}C NMR spectra of compound **59**

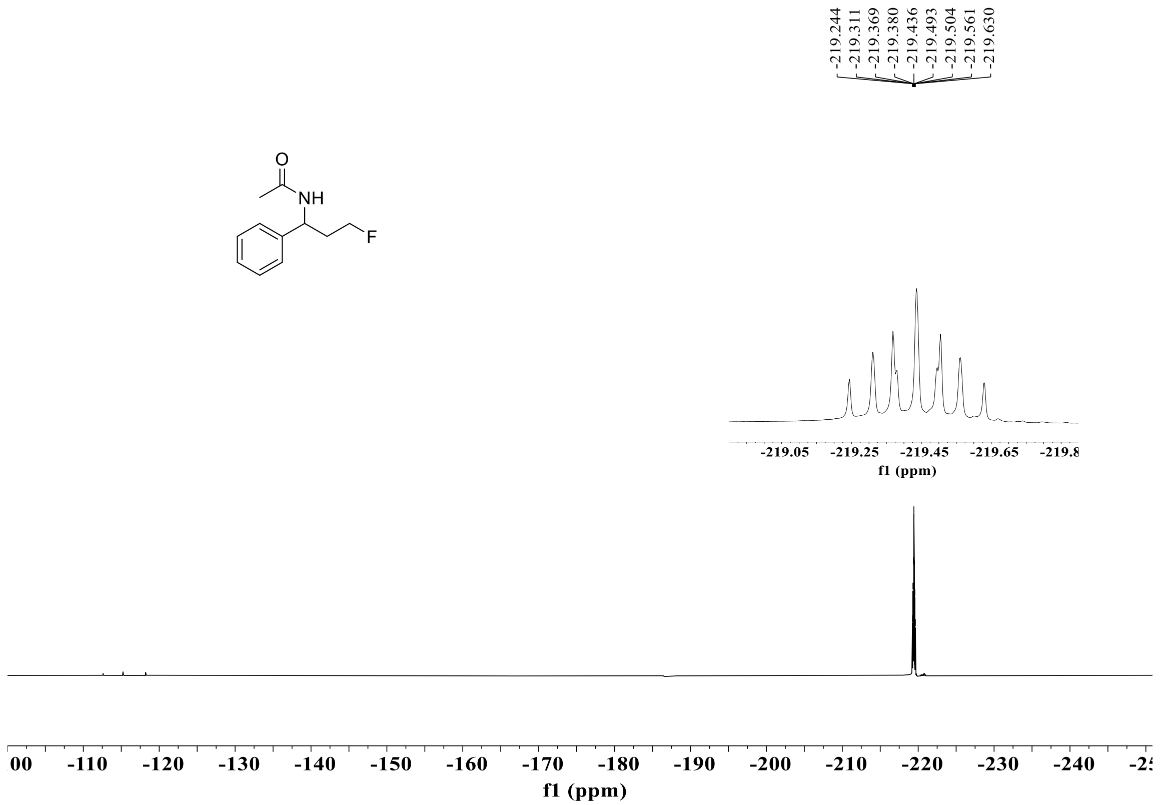

Supplementary Figure 166 ^{19}F NMR spectra of compound **59**

Supplementary References

[1] Gieuw M. H., Ke Z. & Yeung Y.-Y. Lewis Base-Promoted Ring-Opening 1,3-Dioxygenation of Unactivated Cyclopropanes Using a Hypervalent Iodine Reagent. *Angew. Chem. Int. Ed.* **57**, 3782-3786 (2018).

[2] Banik S. M., Mennie K. M. & Jacobsen E. N. Catalytic 1,3-Difunctionalization via Oxidative C–C Bond Activation. *J. Am. Chem. Soc.* **139**, 9152-9155 (2017).

[3] Zhou Y.-Y. & Uyeda C. Reductive Cyclopropanations Catalyzed by Dinuclear Nickel Complexes. *Angew. Chem. Int. Ed.* **55**, 3171-3175 (2016).

[4] Pitts C. R., Ling B., Snyder J. A., Bragg A. E. & Lectka T. Aminofluorination of Cyclopropanes: A Multifold Approach through a Common, Catalytically Generated Intermediate. *J. Am. Chem. Soc.* **138**, 6598-6609 (2016).

[5] Yang S., Wang L., Zhang H., Liu C., Zhang L., Wang X., Zhang G., Li Y. & Zhang Q. Copper-Catalyzed Asymmetric Aminocyanation of Arylcyclopropanes for Synthesis of γ-Amino Nitriles. *ACS Catal.* **9**, 716-721 (2019).

[6] Petzold D., Singh P., Almqvist F. & König B. Visible-Light-Mediated Synthesis of β-Chloro Ketones from Aryl Cyclopropanes. *Angew. Chem. Int. Ed.* **58**, 8577-8580 (2019).

[7] He Z. & Yudin A. K. Palladium-Catalyzed Oxidative Activation of Arylcyclopropanes. *Org. Lett.* **8**, 5829-5832 (2006).

[8] Ilchenko N. O., Hedberg M. & Szabó K. J. Fluorinative ring-opening of cyclopropanes by hypervalent iodine reagents. An efficient method for 1,3-oxyfluorination and 1,3-difluorination. *Chem. Sci.* **8**, 1056-1061 (2017).

[9] Chen C., Feng S. & Chan K. S. Rhodium Porphyrin Catalyzed Regioselective Transfer Hydrogenolysis of C–C σ-Bonds in Cyclopropanes with iPrOH. *Organometallics* **38**, 2582-2589 (2019).

[10] Ge L., Wang D.-X., Xing R., Ma D., Walsh P. J. & Feng C. Photoredox-catalyzed oxo-amination of aryl cyclopropanes. *Nat. Commun.* **10**, 4367 (2019).

[11] Hie L., Baker E. L., Anthony S. M., Desrosiers J.-N., Senanayake C. & Garg N. K. Nickel-Catalyzed Esterification of Aliphatic Amides. *Angew. Chem. Int. Ed.* **55**, 15129-15132 (2016).
